# An atlas of paste fabrics and supplemental paste compositional data from late middle preclassic-period ceramics at the Maya site of Holtun, Guatemala

**DOI:** 10.1016/j.dib.2017.03.024

**Published:** 2017-03-19

**Authors:** Michael G. Callaghan, Daniel Pierce, Brigitte Kovacevich, Michael D. Glascock

**Affiliations:** a University of Central Florida, USA; b Research Reactor, University of Missouri, USA

**Keywords:** Maya, Archeology, Middle Preclassic, Ceramics, Neutron Activation Analysis, Microscopy, Craft production

## Abstract

This data article contains an atlas of paste fabrics and supplemental paste compositional data generated from Late Middle Preclassic-period ceramics at the Maya site of Holtun, Guatemala. The data include maps showing locations of archeological contexts, excavation profiles, photographs and photomicrographs of sherds and paste fabrics, and compositional data produced by Neutron Activation Analysis (NAA) at the Research Reactor, University of Missouri (MURR). The NAA data include a biplot and table of canonical discriminant analyses, Mahalonobis distance calculations, and Euclidian distance searches between the samples.

**Specifications Table**

**Table:** 

Subject area	*Archaeology*
More specific subject area	*Archaeometry*
Type of data	*Maps, tables, charts, photographs, photomicrographs*
How data was acquired	*Digital microscope (Dinolite AMZ750), Neutron Activation Analysis, statistical analysis*
Data format	*Raw and analyzed*
Experimental factors	*Sherds were cleaned, dried, and crushed into powder for NAA*
Experimental features	*Mineralogical and elemental analysis of paste composition*
Data source location	*Archaeological site of Holtun, Department of Peten, Guatemala and MURR*
Data accessibility	*Data is with this article*
Related research article	*2017, Callaghan, Michael G., Daniel Pierce, Brigitte Kovacevich, and Michael D. Glascock. “Chemical Paste Characterization of Late Middle Preclassic-Period Ceramics from Holtun, Guatemala and its Implications for Production and Exchange”. Journal of Archaeological Science Reports* 12:334-345.

**Value of the data**

• Data presented here represent a standard for chemical paste compositional analysis of archeological ceramic material using Neutron Activation Analysis (NAA).
• These data are a benchmark for paste compositional analysis of Late Middle Preclassic-period ceramics in the Maya lowlands.
• These data include the first published atlas of paste fabrics for Middle Preclassic Maya ceramics.
• These data can be compared to compositional data from other Maya sites to identify clay procurement zones, centers of ceramic production, and exchange networks during the Late Middle Preclassic through Postclassic periods.
• These data can be compared to similar data from other world regions in an effort to reconstruct patterns of production and exchange in early states.

## 1 Data

**Fig. 1:**
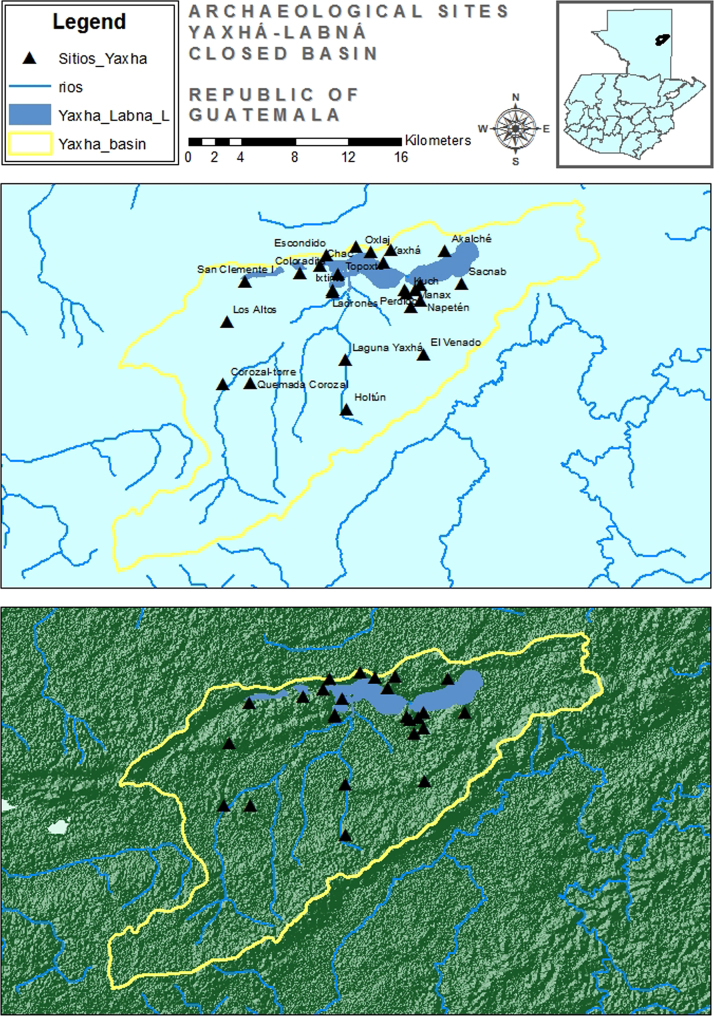
Map of Lake Yaxha Area, Guatemala showing location of the site of Holtun in relation to other sites (map by Rodrigo Guzman).

**Fig. 2:**
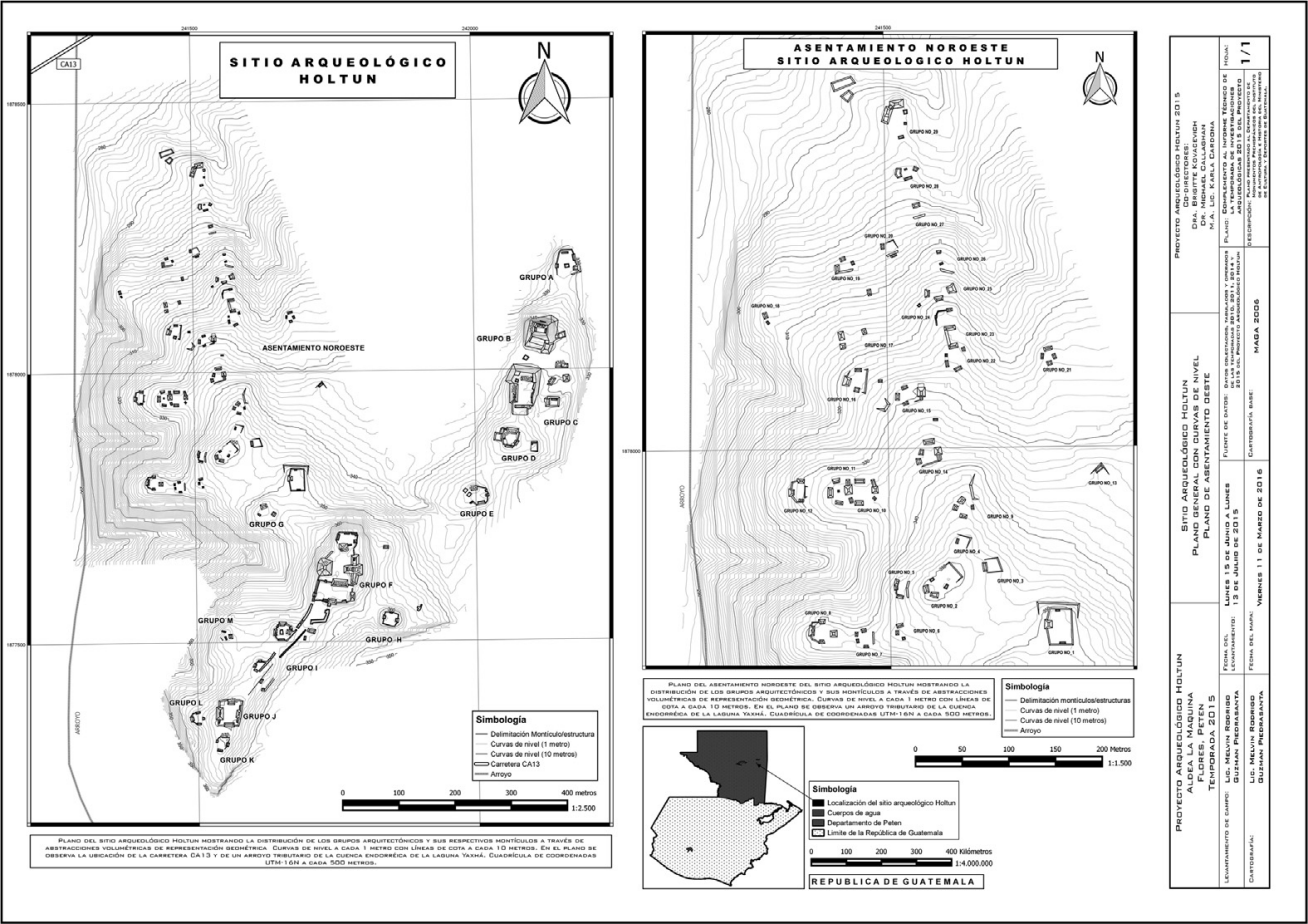
Map of Holtun, Guatemala (map by Rodrigo Guzman).

**Fig. 3:**
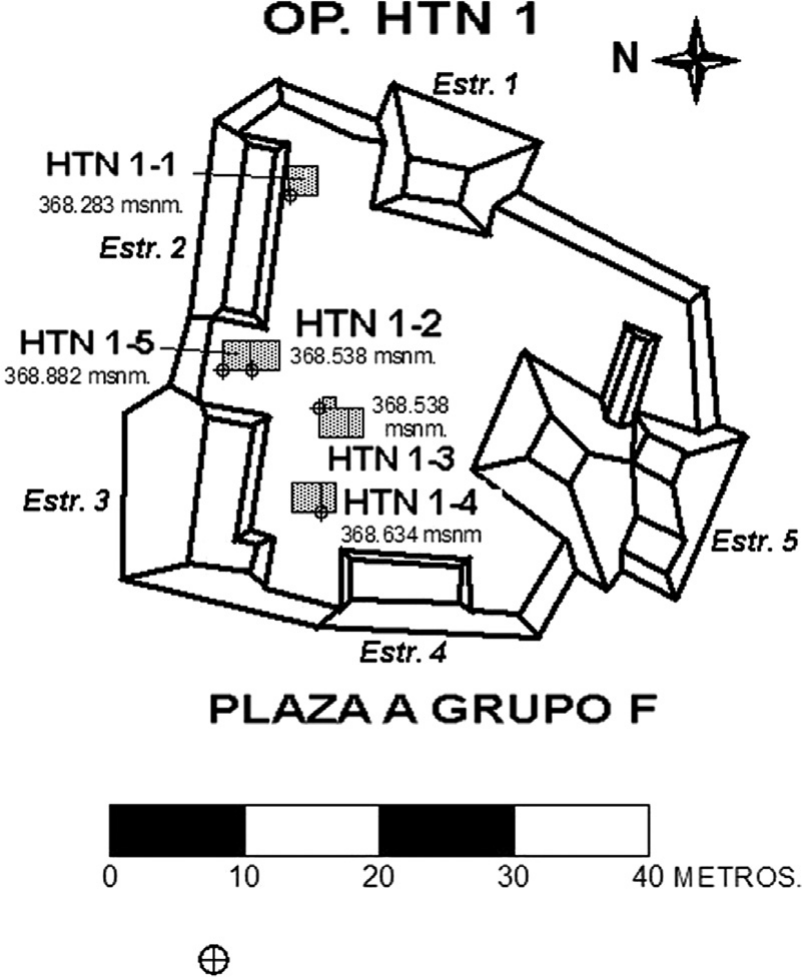
Map of Group F, Patio A with location of excavation units including HTN 1-1 (map by Rodrigo Guzman).

**Fig. 4:**
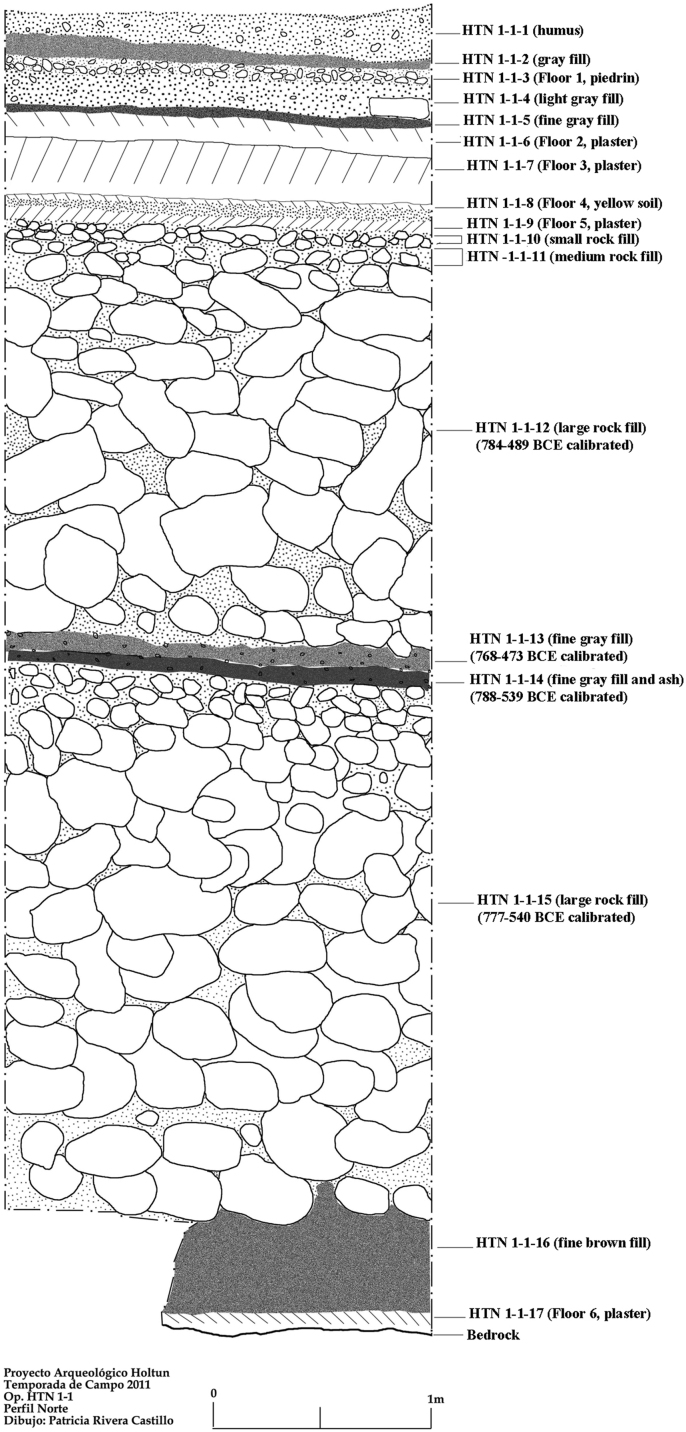
Profile of excavation unit HTN 1-1 (drawing by Patricia Rivera Castillo).

**Fig. 5:**
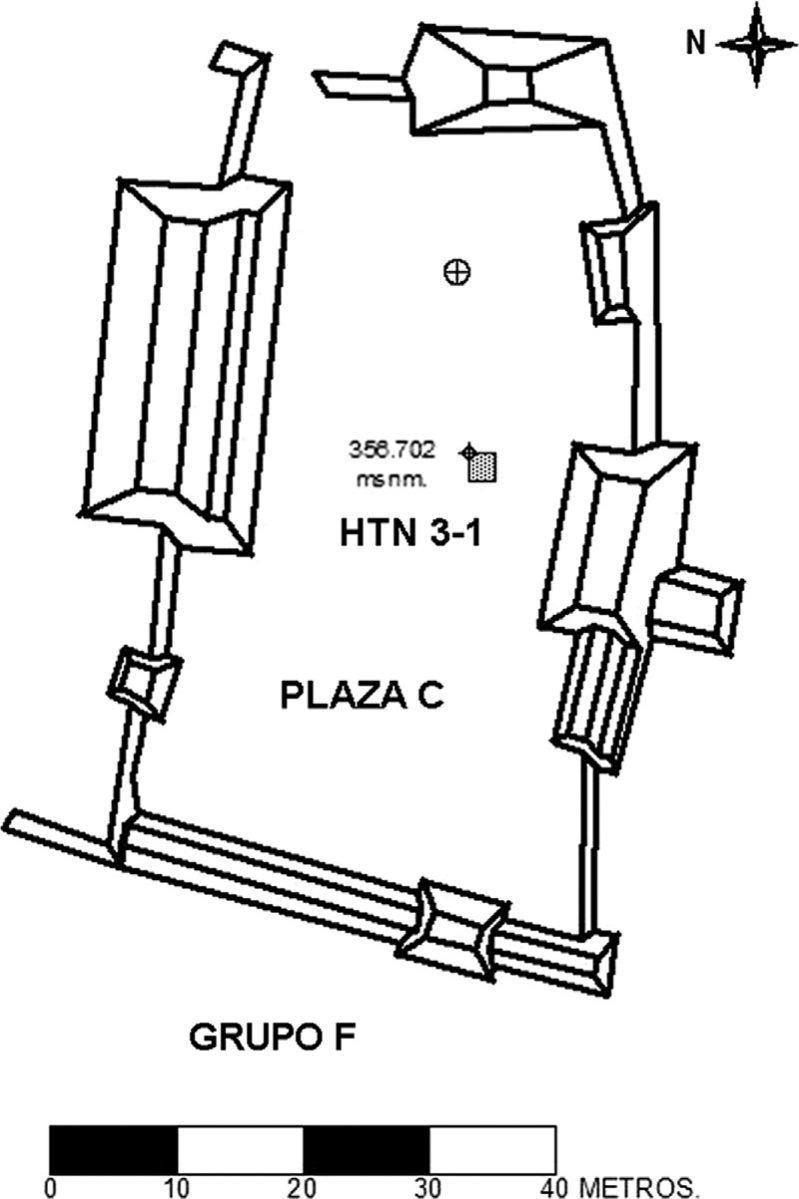
Map of Group F, Patio C with location of excavation units including HTN 3-1 (map by Rodrigo Guzman).

**Fig. 6:**
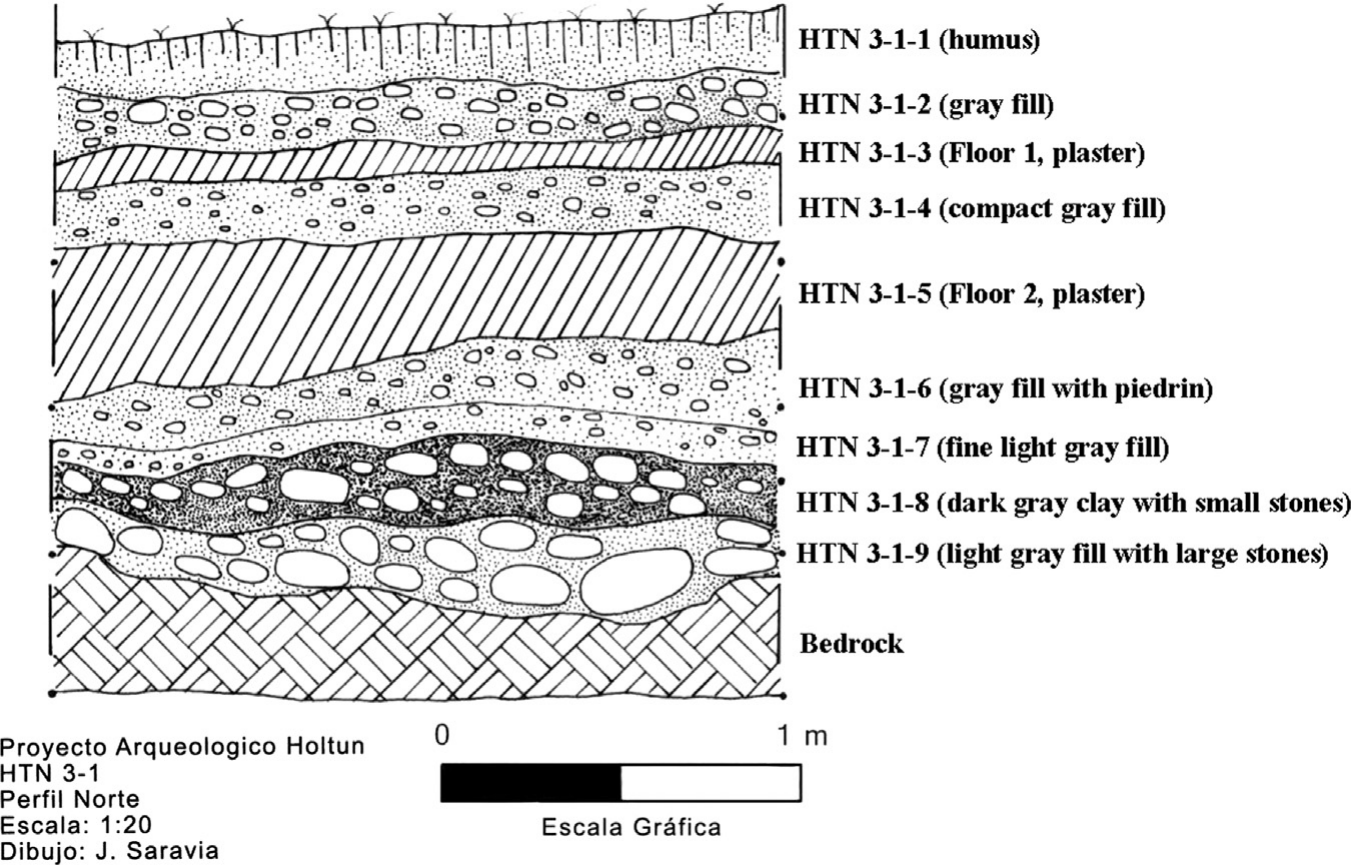
Profile of excavation unit HTN 3-1 (drawing by Juan Saravia).

**Fig. 7:**
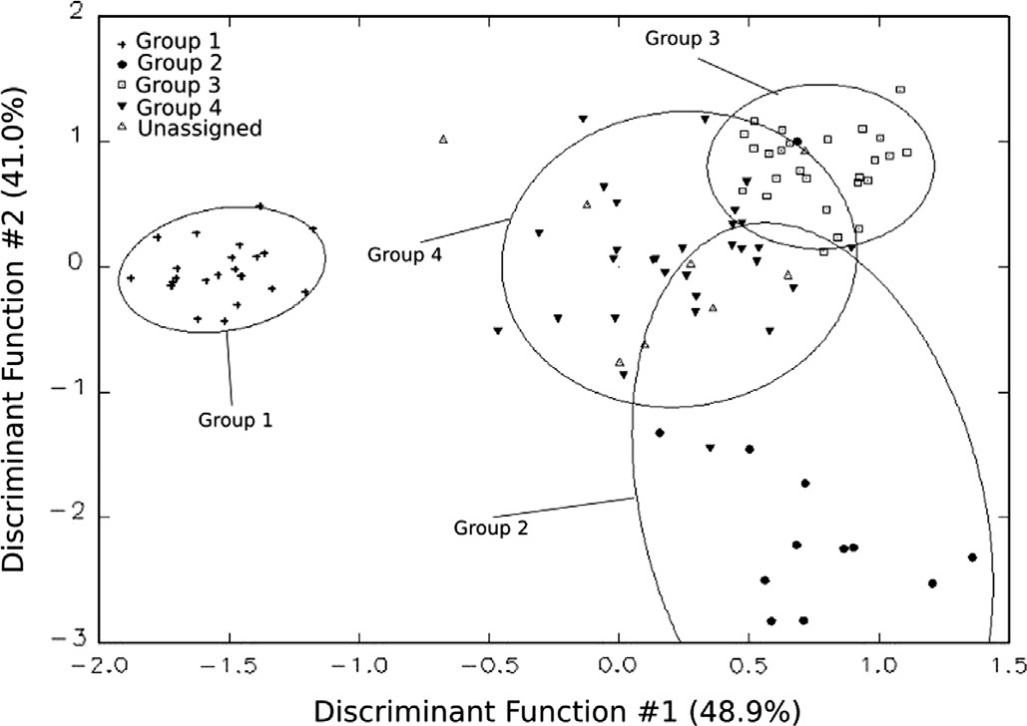
Biplot of Canonical Discriminant Analysis (chart by Daniel Pierce).

**Fig. 8:**
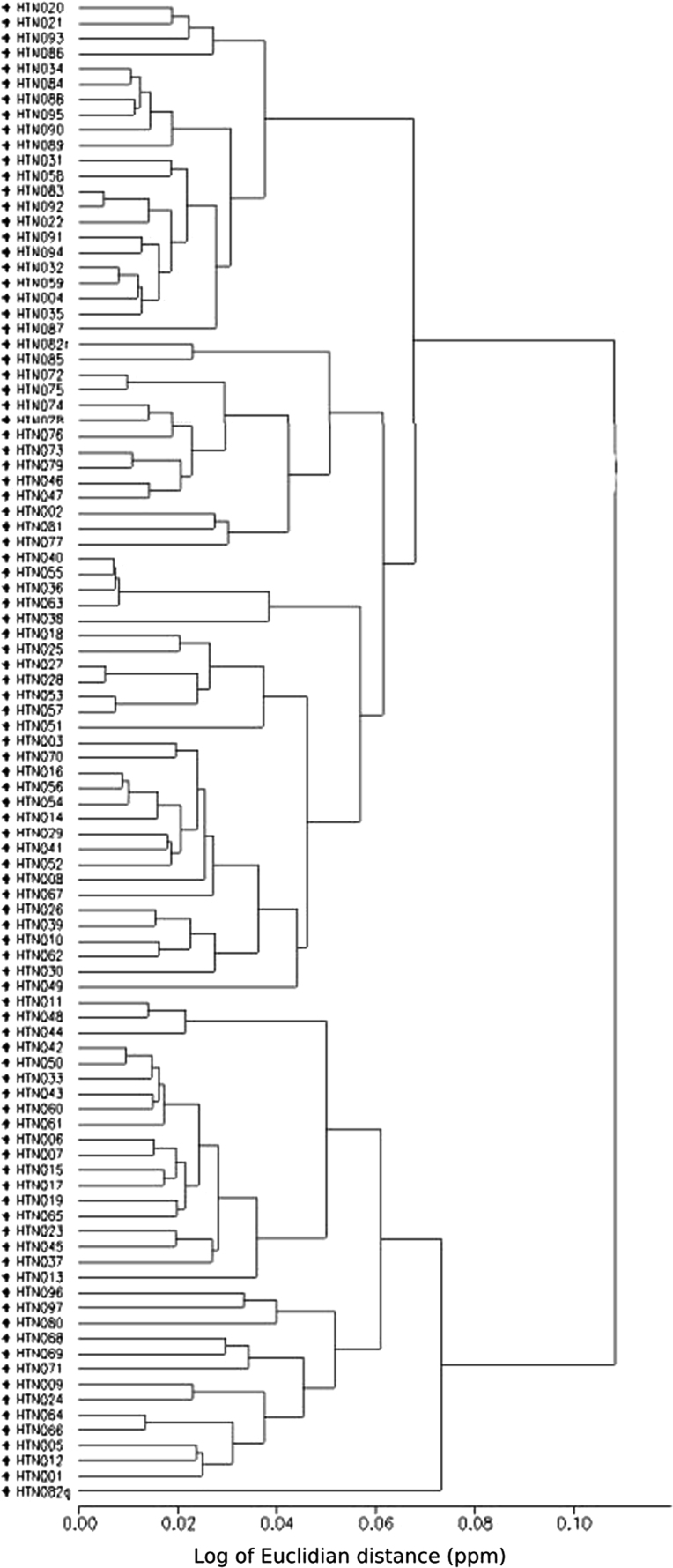
Log of Euclidian distances between samples (chart by Daniel Pierce).

These data include a map showing the location of Holtun in Guatemala and its relation to neighboring sites (Fig. 1), maps of the site showing the location of groups cited in this study (Fig. 2), maps of the locations of excavation units within the patios where samples were found (Fig. 3, Fig. 5), profiles of excavations showing stratigraphy of excavation units where samples were found (Fig. 4, Fig. 6), photographs of sherds and an atlas of paste fabrics with corresponding table of type: varieties (Appendix A), and compositional data produced by Neutron Activation Analysis (NAA) at the Research Reactor, University of Missouri (MURR). The NAA data include a table of Mahalonobis distance calculations of elemental concentrations between samples (Table 1), a chart and table of canonical discriminant analyses of paste groups and elemental concentrations (Fig. 7 and Table 2), and a chart of a log-based Euclidian distance search between the samples (Fig. 8). The data also include results of Chi-Square tests of association between paste chemical composition groups and ceramic attributes including type: variety, group, ware, temper, decoration, and form (Appendix B).

## 2 Experimental design, materials and methods

### 2.1 Study area

The materials for this study consisted of 97 samples of archeological ceramics from eight contexts dating to the Late Middle Preclassic-period at the Maya site of Holtun, Guatemala [6]. The archeological site of Holtun is an intermediate sized civic-ceremonial center with documented occupation beginning in the Late Middle Preclassic through Terminal Classic periods (600 BCE – AD 900) [14], [17], [7], [8], [9]. The site is situated approximately 35 km southwest of Tikal and 12.3 km to the south of Yaxha (Fig. 1). The formal site consists of a monumental epicenter built atop a karstic hill positioned along a roughly northeast-southwest linear axis (Fig. 2). The approximate area of the epicenter is 970×815 m. The epicenter consists of 12 main groups and 86 structures [12]. Holtun, Guatemala has been the focus of investigations since 2010 [14].

### 2.2 Sample

Seven of the eight contexts consisted of sealed stratified deposits located beneath a Late Middle Preclassic-period plaza floor in Group F, Patio A (Fig. 3, Fig. 4). These are sequential layers of fill identified as HTN 1-1-10 through HTN 1-1-16 [14]. Four radiocarbon dates associated with contexts HTN 1-1-12 through HTN 1-1-15 place these layers of deposition between 788 and 473 BCE (calibrated), which is within the established Late Middle Preclassic-period range of 600–300 BCE [4]. The eighth context (HTN 3-1-6) comes from a sealed stratified deposit beneath a Late Middle Preclassic-period plaster floor in Group F, Patio C (Fig. 5, Fig. 6). While no carbon date is associated with this context, stratigraphy and type: variety-mode classification of ceramics in this context indicate HTN 3-1-6 is a sealed, unmixed, Late Middle Preclassic-period deposit

### 2.3 Type: Variety-mode classification and digital photomicrographs

A type: variety-mode classification was performed on all sherds within the eight contexts (see [5], [10]). Next, all samples were photographed with a Canon EOS Rebel DSLR 10 megapixel camera (Appendix A). Pastes were analyzed with a Dinolite AMZ750 digital stereomicroscope with photomicrographs taken at 50x and 250x magnification (Appendix A). Three wares are represented in the sample. Flores Waxy Ware is the slipped serving ware tradition of red, black, and cream colors of the Joventud, Chunhinta, and Pital Groups respectively. Slipped ceramics within Flores Waxy Ware also included two dichrome types: namely, Muxanal Red-on-cream and Tierra Mojada Resist. Forms of slipped ceramics included bowls and jars. Unslipped utilitarian ceramics belong to Uaxactun Unslipped Ware and are classified within the Jocote and Achiotes Groups. Sherds of Mars Orange Paste Ware were also included in the sample. Mars Orange Paste Ware is characterized by fine orange paste with few to no inclusions, or volcanic ash inclusions. This ware appears in bowls, dishes, and jars.

### 2.4 Sample preparation and NAA

Ceramic samples were prepared for NAA using procedures standard at MURR. Fragments of about 1 cm^2^ were removed from each sample and abraded using a silicon carbide burr in order to remove glaze, slip, paint, and adhering soil, thereby reducing the risk of measuring contamination. The samples were washed in deionized water and allowed to dry in the laboratory. Once dry, the individual sherds were ground to powder in an agate mortar to homogenize the samples. Archival samples were retained from each sherd (when possible) for future research.

Two analytical samples were prepared from each source specimen. Portions of approximately 150 mg of powder were weighed into clean high-density polyethylene vials used for short irradiations at MURR. At the same time, 200 mg of each sample was weighed into clean high-purity quartz vials used for long irradiations. Individual sample weights were recorded to the nearest 0.01 mg using an analytical balance. Both vials were sealed prior to irradiation. Along with the unknown samples, Standards made from National Institute of Standards and Technology (NIST) certified standard reference materials of SRM-1633a (coal fly ash) and SRM-688 (basalt rock) were similarly prepared, as were quality control samples (e.g., standards treated as unknowns) of SRM-278 (obsidian rock) and Ohio Red Clay (a standard developed for in-house applications).

Neutron activation analysis of ceramics at MURR, which consists of two irradiations and a total of three gamma counts, constitutes a superset of the procedures used at most other NAA laboratories [11], [15], [16]. As discussed in detail by Glascock [11], a short irradiation is carried out through the pneumatic tube irradiation system. Samples in the polyvials are sequentially irradiated, two at a time, for five seconds by a neutron flux of 8×10^13^ n cm^−2^ s^−1^. The 720-second count yields gamma spectra containing peaks for nine short-lived elements aluminum (Al), barium (Ba), calcium (Ca), dysprosium (Dy), potassium (K), manganese (Mn), sodium (Na), titanium (Ti), and vanadium (V). The samples are encapsulated in quartz vials and are subjected to a 24–hour irradiation at a neutron flux of 5×10^13^ n cm^−2^ s^−1^. This long irradiation is analogous to the single irradiation utilized at most other laboratories. After the long irradiation, samples decay for seven days, and then are counted for 1800 s (the "middle count") on a high-resolution germanium detector coupled to an automatic sample changer. The middle count yields determinations of seven medium half-life elements, namely arsenic (As), lanthanum (La), lutetium (Lu), neodymium (Nd), samarium (Sm), uranium (U), and ytterbium (Yb). After an additional three- or four-week decay, a final count of 8500 s is carried out on each sample. The latter measurement yields the following 17 long half-life elements: cerium (Ce), cobalt (Co), chromium (Cr), cesium (Cs), europium (Eu), iron (Fe), hafnium (Hf), nickel (Ni), rubidium (Rb), antimony (Sb), scandium (Sc), strontium (Sr), tantalum (Ta), terbium (Tb), thorium (Th), zinc (Zn), and zirconium (Zr). The element concentration data from the three measurements are tabulated in parts per million.

### 2.5 Statistical analysis of NAA data

Irradiation and gamma-ray spectroscopy followed procedures established by Glascock [11] and Neff [15], [16]. The interpretation of compositional data obtained from the analysis of archeological materials is discussed in detail elsewhere (e.g., [1], [2], [3], [11], [13], [16]). The approach used to interpret chemical data for pottery involves hierarchical cluster analysis (HCA) and principal component analysis (PCA) to establish initial groupings within the sample (see [6] Fig. 6). After constructing base groups through HCA and PCA, bivariate plots were used to refine groups (see [6] Figs. 7–10 and Table 2). Note, Strontium (Sr) and Nickel (Ni) were removed from all statistical techniques due to the high number of missing values within the dataset. Next, Mahalonobis distance based probabilities were calculated to assess likelihood of group membership (Table 1). A canonical discriminant analysis (CDA) was then conducted using the previously identified groups (Fig. 7 and Table 2). Finally, Euclidian Distance Searches (EDS) were conducted to identify the most chemically similar previously analyzed samples in MURR׳s Mesoamerican NAA database (Fig. 8, also see [6] Table 1).

### 2.6 Chi-square tests of association

Chi-square tests of association were run between paste group and the following variables: type: variety, ceramic group, ceramic ware, temper, vessel form, and decoration (Appendix B).

## Figures and Tables

**Table 1:** Mahalonobis distance calculations for each identified group.

Membership probabilities(%) for samples in Group: unassign					
Probabilities calculated by projecting unknowns against reference Groups.					
*ANID*	*Group 1*	*Group 2*	*Group 3*	*Group 4*	*Best Group*
*HTN002*	*0.000*	*18.076*	*34.374*	*0.344*	*Group 3*
*HTN003*	*0.000*	*4.308*	*87.639*	*0.633*	*Group 3*
*HTN038*	*0.000*	*5.096*	*32.951*	*2.203*	*Group 3*
*HTN068*	*0.000*	*0.138*	*3.548*	*19.799*	*Group 4*
*HTN069*	*0.000*	*0.108*	*3.291*	*25.457*	*Group 4*
*HTN082g*	*0.000*	*0.001*	*0.002*	*7.465*	*Group 4*
*HTN082r*	*0.000*	*3.314*	*99.872*	*0.158*	*Group 3*
*HTN085*	*0.000*	*13.100*	*66.881*	*0.189*	*Group 3*

Membership probabilities(%) for samples in Group: Group 1					

*HTN004*	*72.337*	*2.073*	*0.014*	*0.000*	*Group 1*
*HTN020*	*51.099*	*2.529*	*0.009*	*0.000*	*Group 1*
*HTN021*	*65.876*	*1.894*	*0.008*	*0.000*	*Group 1*
*HTN022*	*34.040*	*2.239*	*0.034*	*0.000*	*Group 1*
*HTN031*	*46.706*	*2.484*	*0.033*	*0.000*	*Group 1*
*HTN032*	*60.626*	*1.875*	*0.012*	*0.000*	*Group 1*
*HTN034*	*70.936*	*1.958*	*0.011*	*0.000*	*Group 1*
*HTN035*	*18.186*	*1.192*	*0.005*	*0.000*	*Group 1*
*HTN058*	*15.092*	*3.861*	*0.092*	*0.000*	*Group 1*
*HTN059*	*72.621*	*2.223*	*0.018*	*0.000*	*Group 1*
*HTN083*	*80.221*	*2.109*	*0.012*	*0.000*	*Group 1*
*HTN084*	*97.824*	*2.549*	*0.015*	*0.000*	*Group 1*
*HTN086*	*7.526*	*1.846*	*0.004*	*0.000*	*Group 1*
*HTN087*	*9.063*	*5.153*	*0.022*	*0.000*	*Group 1*
*HTN088*	*59.296*	*4.184*	*0.040*	*0.000*	*Group 1*
*HTN089*	*22.226*	*4.960*	*0.077*	*0.000*	*Group 1*
*HTN090*	*93.895*	*3.232*	*0.022*	*0.000*	*Group 1*
*HTN091*	*77.800*	*3.206*	*0.016*	*0.000*	*Group 1*
*HTN092*	*56.667*	*1.851*	*0.012*	*0.000*	*Group 1*
*HTN093*	*42.797*	*1.765*	*0.006*	*0.000*	*Group 1*
*HTN094*	*76.728*	*3.781*	*0.026*	*0.000*	*Group 1*
*HTN095*	*61.522*	*4.098*	*0.039*	*0.000*	*Group 1*

Membership probabilities(%) for samples in Group: Group 2					

*HTN030*	*0.000*	*13.599*	*42.838*	*0.003*	*Group 3*
*HTN046*	*0.000*	*76.793*	*2.285*	*0.000*	*Group 2*
*HTN047*	*0.000*	*89.429*	*1.647*	*0.001*	*Group 2*
*HTN072*	*0.000*	*75.981*	*7.595*	*0.001*	*Group 2*
*HTN073*	*0.000*	*51.344*	*0.416*	*0.000*	*Group 2*
*HTN074*	*0.000*	*98.935*	*3.131*	*0.002*	*Group 2*
*HTN075*	*0.000*	*58.209*	*6.176*	*0.000*	*Group 2*
*HTN076*	*0.000*	*55.926*	*0.636*	*0.002*	*Group 2*
*HTN077*	*0.000*	*32.025*	*3.978*	*0.086*	*Group 2*
*HTN078*	*0.000*	*92.504*	*4.946*	*0.007*	*Group 2*
*HTN079*	*0.000*	*43.874*	*0.319*	*0.000*	*Group 2*
*HTN081*	*0.000*	*19.301*	*8.903*	*0.306*	*Group 2*

Membership probabilities(%) for samples in Group: Group 3					

*HTN008*	*0.000*	*0.993*	*56.213*	*5.942*	*Group 3*
*HTN010*	*0.000*	*1.756*	*94.733*	*0.349*	*Group 3*
*HTN014*	*0.000*	*0.444*	*41.650*	*9.379*	*Group 3*
*HTN016*	*0.000*	*0.839*	*59.081*	*4.960*	*Group 3*
*HTN018*	*0.000*	*1.690*	*81.582*	*0.032*	*Group 3*
*HTN025*	*0.000*	*0.709*	*55.327*	*0.016*	*Group 3*
*HTN026*	*0.000*	*0.159*	*24.795*	*2.901*	*Group 3*
*HTN027*	*0.000*	*0.995*	*15.568*	*0.000*	*Group 3*
*HTN028*	*0.000*	*0.883*	*14.346*	*0.000*	*Group 3*
*HTN029*	*0.000*	*2.114*	*77.451*	*1.963*	*Group 3*
*HTN036*	*0.000*	*29.981*	*23.529*	*0.162*	*Group 2*
*HTN039*	*0.000*	*0.974*	*81.168*	*0.443*	*Group 3*
*HTN040*	*0.000*	*27.863*	*22.109*	*0.193*	*Group 2*
*HTN041*	*0.000*	*9.931*	*81.641*	*0.114*	*Group 3*
*HTN049*	*0.000*	*0.320*	*45.197*	*0.828*	*Group 3*
*HTN051*	*0.000*	*10.461*	*6.393*	*0.000*	*Group 2*
*HTN052*	*0.000*	*2.042*	*89.812*	*0.944*	*Group 3*
*HTN053*	*0.000*	*0.793*	*40.838*	*0.002*	*Group 3*
*HTN054*	*0.000*	*0.561*	*52.464*	*5.229*	*Group 3*
*HTN055*	*0.000*	*33.757*	*15.293*	*0.143*	*Group 2*
*HTN056*	*0.000*	*1.225*	*73.307*	*2.539*	*Group 3*
*HTN057*	*0.000*	*0.969*	*45.858*	*0.003*	*Group 3*
*HTN062*	*0.000*	*2.900*	*97.410*	*0.090*	*Group 3*
*HTN063*	*0.000*	*22.397*	*20.604*	*0.292*	*Group 2*
*HTN067*	*0.000*	*4.045*	*95.101*	*0.392*	*Group 3*
*HTN070*	*0.000*	*2.588*	*77.243*	*1.742*	*Group 3*

Membership probabilities(%) for samples in Group: Group 4					

*HTN001*	*0.000*	*0.048*	*2.350*	*60.457*	*Group 4*
*HTN005*	*0.000*	*0.069*	*3.594*	*57.395*	*Group 4*
*HTN006*	*0.000*	*0.004*	*0.053*	*63.313*	*Group 4*
*HTN007*	*0.000*	*0.002*	*0.024*	*47.950*	*Group 4*
*HTN009*	*0.000*	*0.130*	*4.027*	*25.692*	*Group 4*
*HTN011*	*0.000*	*0.000*	*0.001*	*15.006*	*Group 4*
*HTN012*	*0.000*	*0.044*	*2.343*	*69.313*	*Group 4*
*HTN013*	*0.000*	*0.090*	*9.873*	*37.788*	*Group 4*
*HTN015*	*0.000*	*0.004*	*0.083*	*80.786*	*Group 4*
*HTN017*	*0.000*	*0.010*	*0.426*	*88.804*	*Group 4*
*HTN019*	*0.000*	*0.009*	*0.356*	*90.987*	*Group 4*
*HTN023*	*0.000*	*0.043*	*4.253*	*48.542*	*Group 4*
*HTN024*	*0.000*	*0.362*	*12.228*	*23.390*	*Group 4*
*HTN033*	*0.000*	*0.008*	*0.289*	*69.942*	*Group 4*
*HTN037*	*0.000*	*0.011*	*0.484*	*88.236*	*Group 4*
*HTN042*	*0.000*	*0.005*	*0.121*	*54.943*	*Group 4*
*HTN043*	*0.000*	*0.008*	*0.389*	*40.913*	*Group 4*
*HTN044*	*0.000*	*0.001*	*0.003*	*29.583*	*Group 4*
*HTN045*	*0.000*	*0.042*	*4.024*	*52.175*	*Group 4*
*HTN048*	*0.000*	*0.000*	*0.001*	*13.091*	*Group 4*
*HTN050*	*0.000*	*0.003*	*0.067*	*48.924*	*Group 4*
*HTN060*	*0.000*	*0.004*	*0.085*	*42.828*	*Group 4*
*HTN061*	*0.000*	*0.002*	*0.023*	*60.845*	*Group 4*
*HTN064*	*0.000*	*0.265*	*25.529*	*20.622*	*Group 3*
*HTN065*	*0.000*	*0.014*	*0.769*	*77.593*	*Group 4*
*HTN066*	*0.000*	*0.077*	*6.668*	*60.375*	*Group 4*
*HTN071*	*0.000*	*0.043*	*0.829*	*10.618*	*Group 4*
*HTN080*	*0.000*	*0.006*	*0.104*	*33.997*	*Group 4*
*HTN096*	*0.000*	*0.006*	*0.084*	*15.241*	*Group 4*
*HTN097*	*0.000*	*0.035*	*1.192*	*38.473*	*Group 4*

**Table 2:** Canonical Discriminant Analysis: Canonical Discriminant Analysis of four identified source groups in the Holtun sample.

Element	CD1	CD2	CD3
	82.04534	12.38223	5.57243
Sm	−2.41498	0.164417	−0.88161
Eu	1.76103	−0.43321	0.583455
Al	0.977728	0.331017	−0.84942
Hf	−0.69086	−0.43159	−0.87062
Ti	0.804131	0.773795	0.256648
Sc	−0.60128	−0.38501	0.561172
Lu	0.652725	0.177529	0.546106
Dy	−0.80766	−0.19096	0.190131
La	0.701471	0.473308	−0.06208
Th	0.380362	0.137782	0.704084
Fe	−0.50274	0.206254	−0.38915
Sb	−0.05235	−0.54797	−0.29008
Yb	−0.23533	0.274016	−0.43433
Ca	0.481173	0.253661	−0.0568
Zn	−0.41997	0.175993	0.199425
Nd	0.171687	−0.0996	0.207373
Cs	−0.17353	0.020681	−0.18374
Tb	−0.13697	−0.17429	−0.11571
As	0.049685	0.084167	0.1929
Cr	0.113298	−0.12899	0.120426
U	−0.10773	0.173209	0.020955
Ta	0.117935	−0.05244	0.151552
Na	0.122198	0.081784	−0.13194
Mn	0.183093	−0.04356	0.052349
Zr	−0.13084	0.042307	0.127145
V	−0.06074	0.134068	−0.11214
Ce	0.09062	−0.13003	−0.01066
Co	0.002246	0.123474	−0.01544
K	−0.05889	0.072121	0.078588
Ba	−0.01922	−0.00726	−0.06326
Rb	−0.04245	−0.02099	−0.01691
		Wilk׳s lambda:	0.000538
		Approx. F:	20.59806
		*p*‐value:	4.98E‐59
