# Supplementary material for: An atlas of paste fabrics and supplemental paste compositional data from late middle preclassic-period ceramics at the Maya site of Holtun, Guatemala
Source: Data Brief. 2017 Mar 19;12:55–67. doi: 10.1016/j.dib.2017.03.024 (PMC5376253; doi:10.1016/j.dib.2017.03.024)
Supplement: Supplementary file 2 — Supplementary material [file mmc2.docx]

**List of Figures**

Figure 1 Map of Lake Yaxha Area, Guatemala showing location of the site of Holtun in relation to other sites (map by Rodrigo Guzman)

Figure 2 Map of Holtun, Guatemala (map by Rodrigo Guzman)

Figure 3 Map of Group F, Patio A with location of excavation units including HTN 1-1 (map by Rodrigo Guzman)

Figure 4 Profile of excavation unit HTN 1-1 (drawing by Patricia Rivera Castillo)

Figure 5 Map of Group F, Patio C with location of excavation units including HTN 3-3 (map by Rodrigo Guzman)

Figure 6 Profile of excavation unit HTN 3-1 (drawing by Juan Saravia)

Figure 7 Biplot of Canonical Discriminant Analysis (chart by Daniel Pierce)

Figure 8 Log of Euclidian distances between samples (chart by Daniel Pierce)

**List of Tables**

Table 1 Mahalanobis Distance Calculations of samples run using NAA

Table 2 Canonical Discriminant Analysis of four identified source groups in the Holtun sample

**Appendix A**

Table A-1 List of samples with context numbers, wares, type: variety, temper, and form

Figures 1-97 Photos of sherds and micrographs of paste fabrics at 50x and 250x magnification of samples HTN 1 through HTN 97 used in this study (photos by Whitney Goodwin)

**Appendix B**

Table B-1 Chi-square Tests of Association between paste groups and other variables
