# Supplementary figures and images for: An atlas of paste fabrics and supplemental paste compositional data from late middle preclassic-period ceramics at the Maya site of Holtun, Guatemala (part 2 of 2)
Source: Data Brief. 2017 Mar 19;12:55–67. doi: 10.1016/j.dib.2017.03.024 (PMC5376253; doi:10.1016/j.dib.2017.03.024)

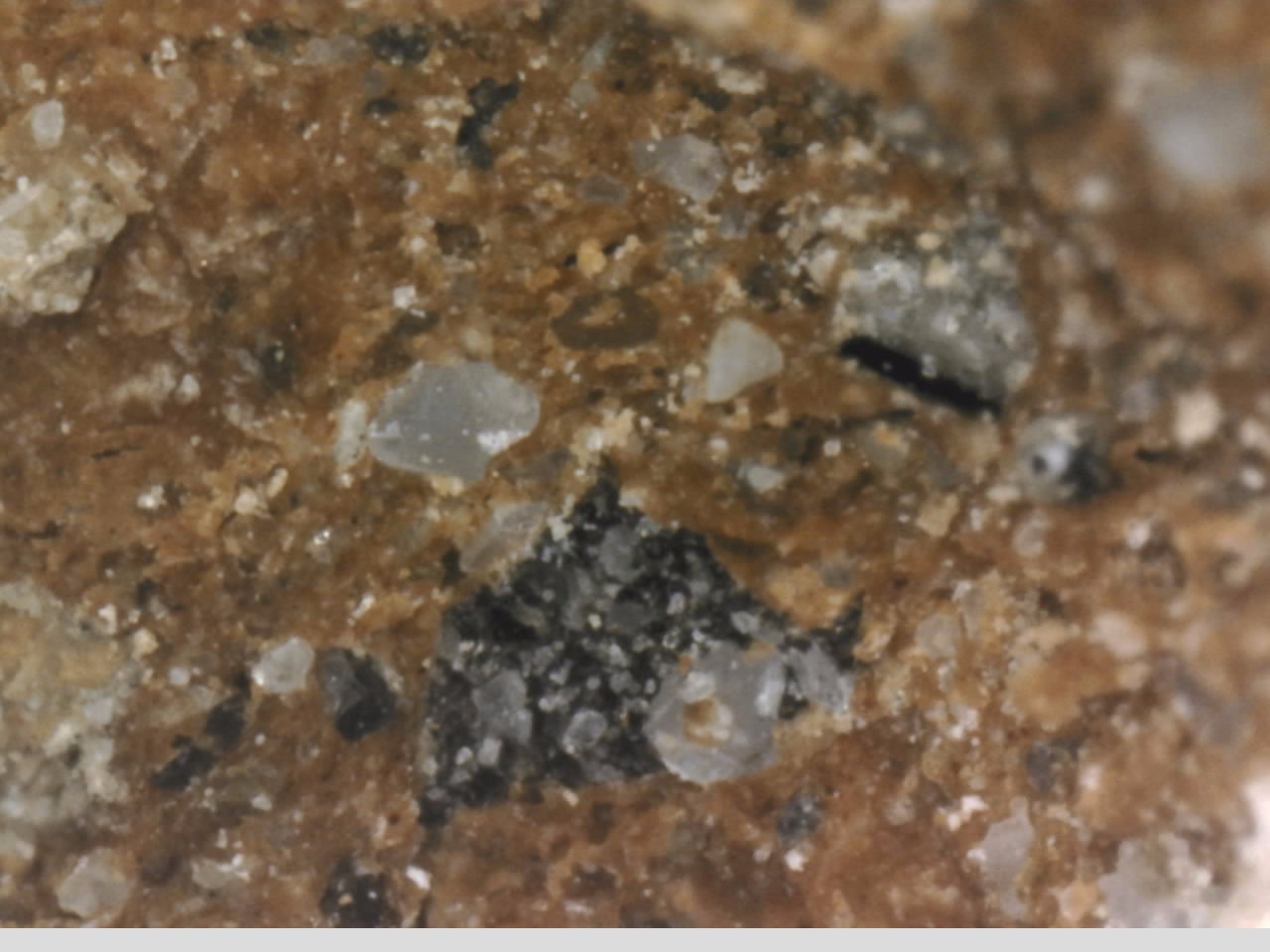

Supplement: Supplementary file 3 — Supplementary material [file mmc3.zip › Appendix A/HTN 40/HTN 40-250m-2.jpg]

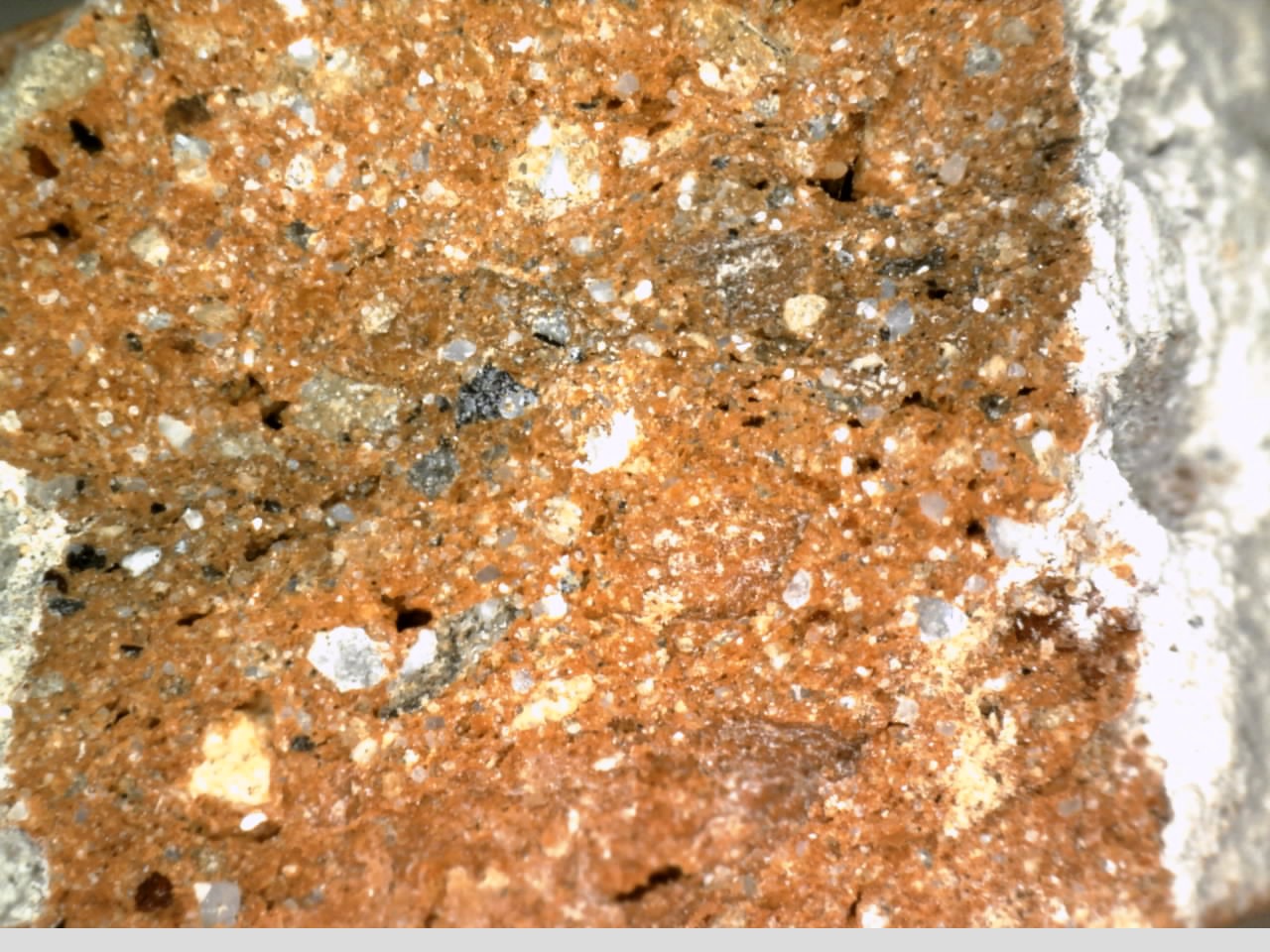

Supplement: Supplementary file 3 — Supplementary material [file mmc3.zip › Appendix A/HTN 40/HTN 40-50m-2.jpg]

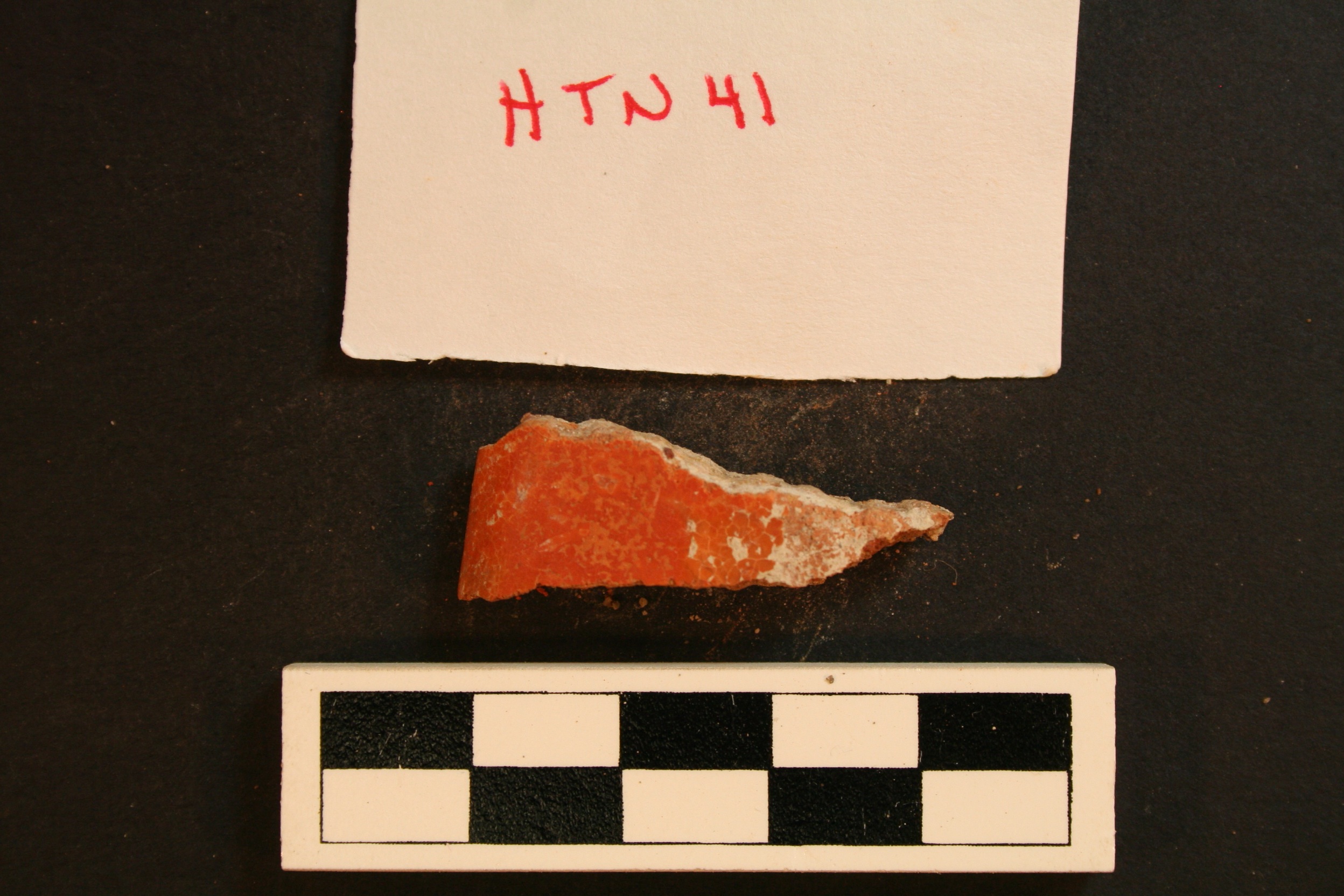

Supplement: Supplementary file 3 — Supplementary material [file mmc3.zip › Appendix A/HTN 41/41a.JPG]

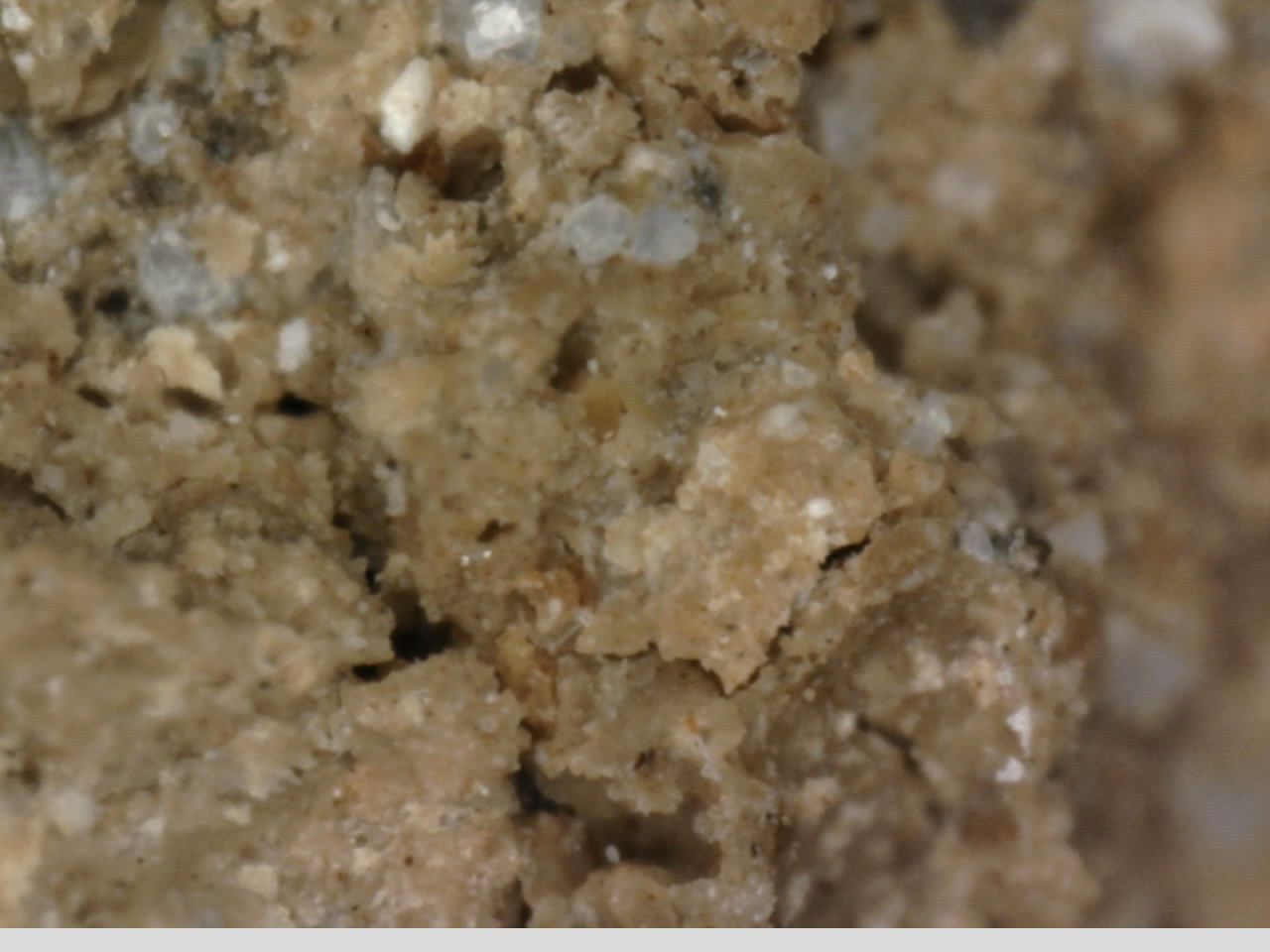

Supplement: Supplementary file 3 — Supplementary material [file mmc3.zip › Appendix A/HTN 41/HTN 41-250m-2.jpg]

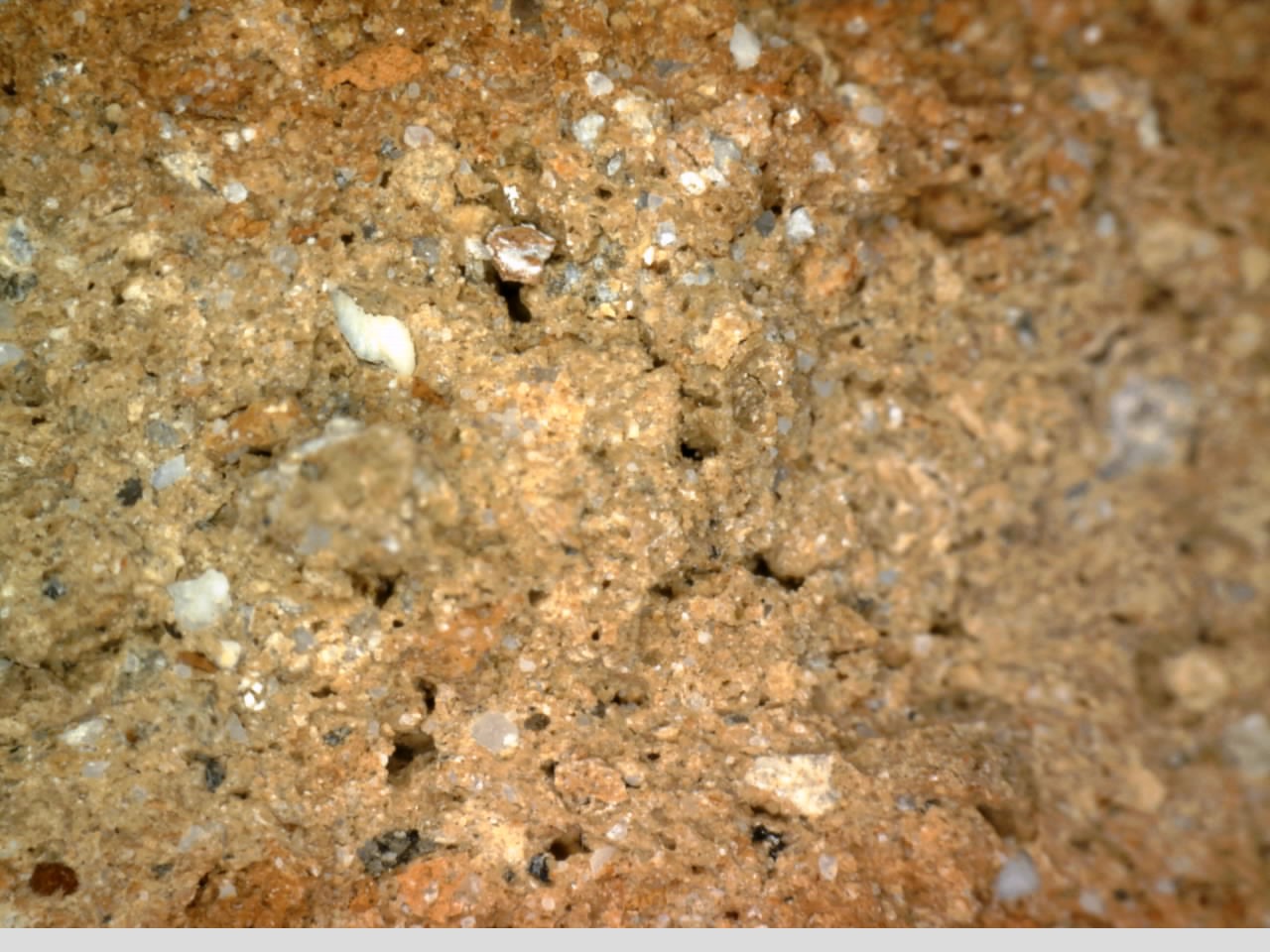

Supplement: Supplementary file 3 — Supplementary material [file mmc3.zip › Appendix A/HTN 41/HTN 41-50m-2.jpg]

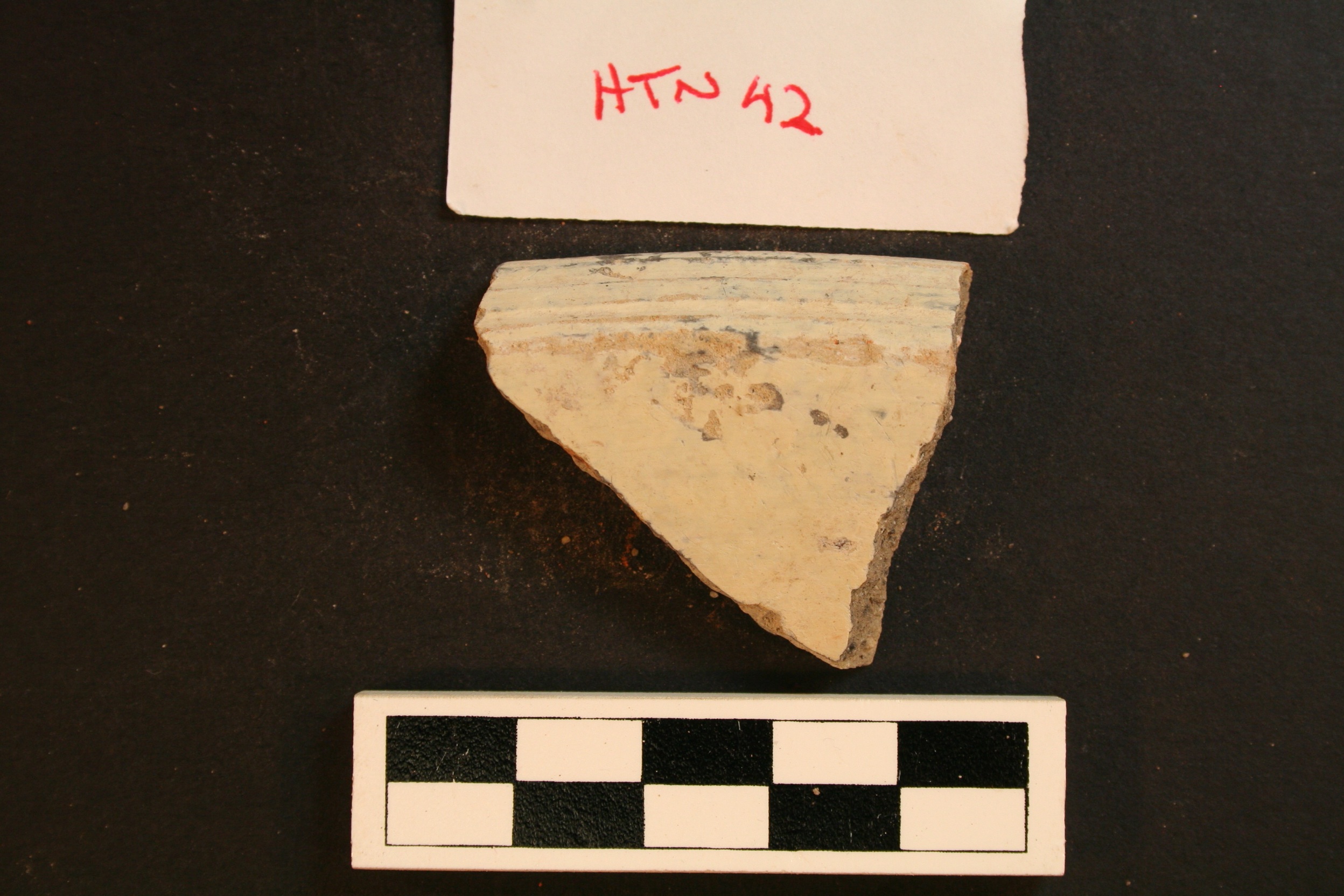

Supplement: Supplementary file 3 — Supplementary material [file mmc3.zip › Appendix A/HTN 42/42a.JPG]

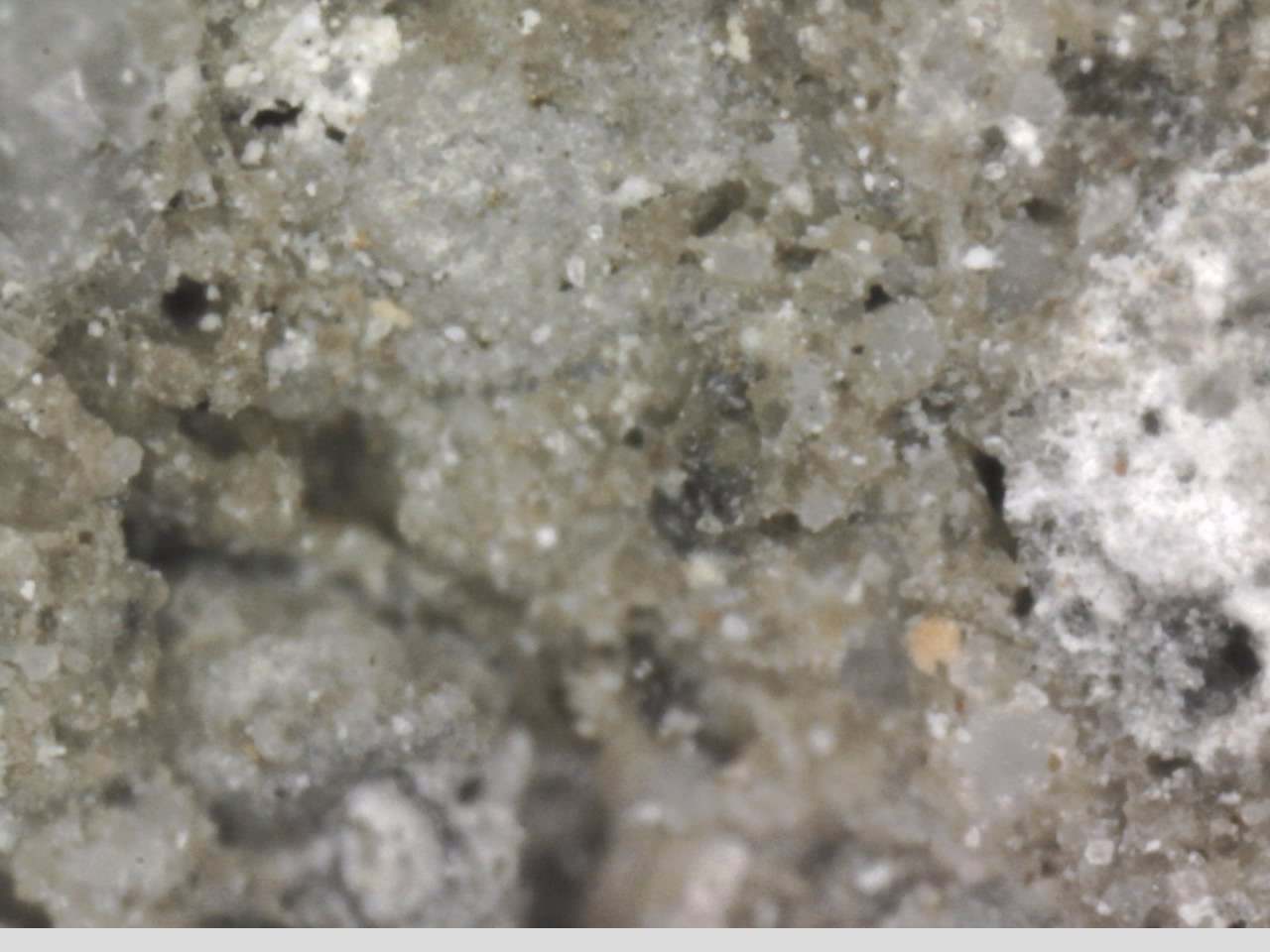

Supplement: Supplementary file 3 — Supplementary material [file mmc3.zip › Appendix A/HTN 42/HTN 42-250m-8.jpg]

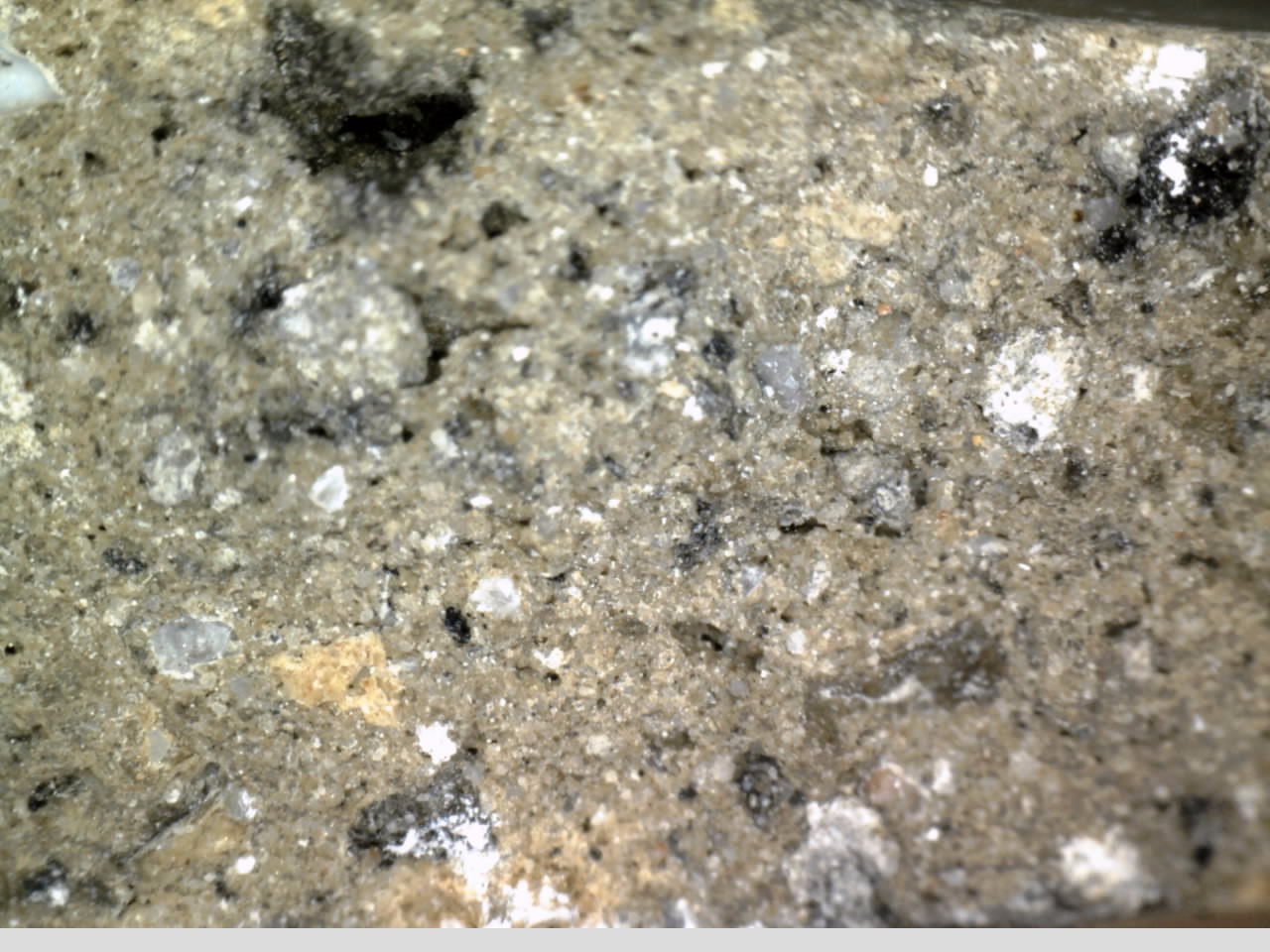

Supplement: Supplementary file 3 — Supplementary material [file mmc3.zip › Appendix A/HTN 42/HTN 42-50m-3.jpg]

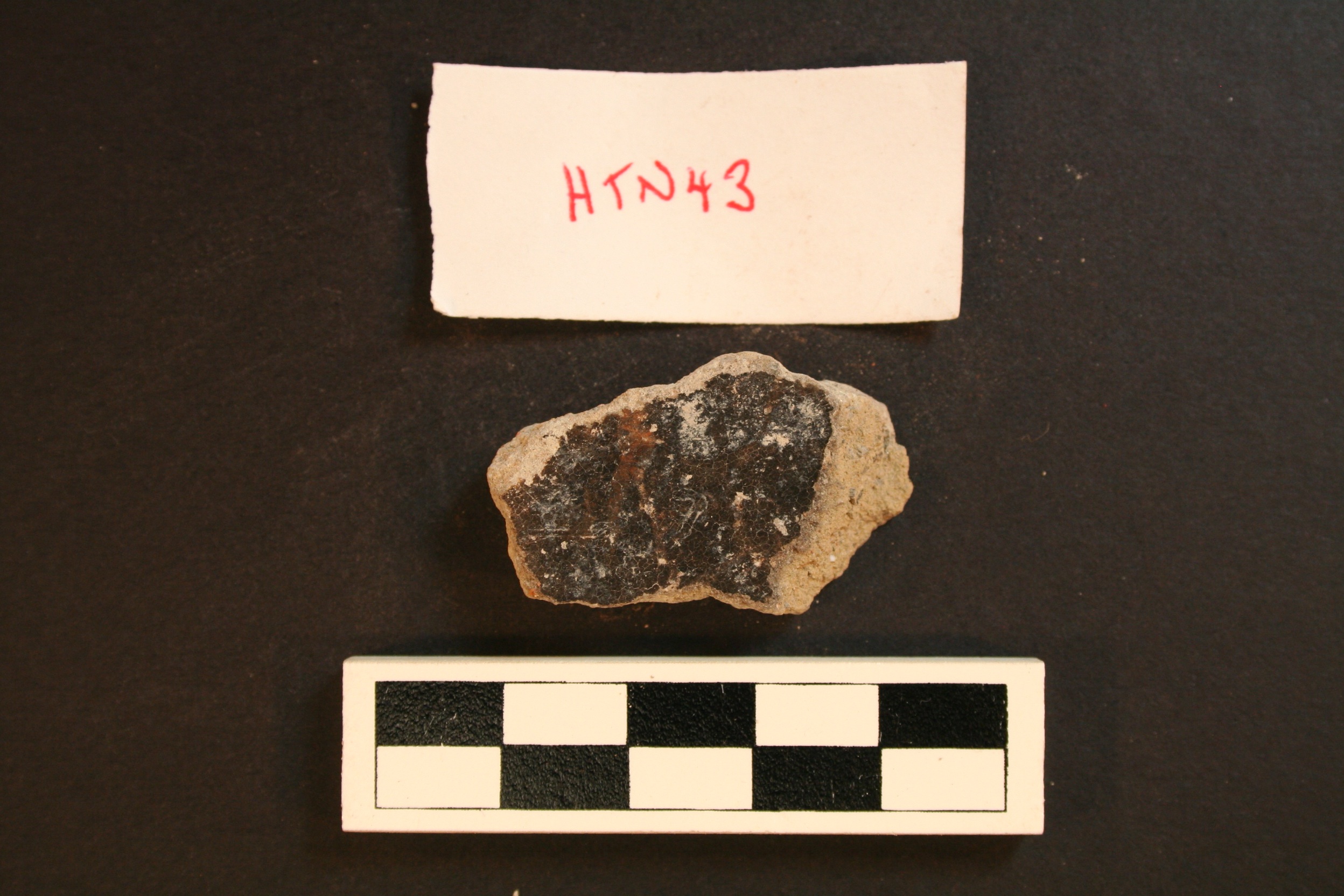

Supplement: Supplementary file 3 — Supplementary material [file mmc3.zip › Appendix A/HTN 43/43a.JPG]

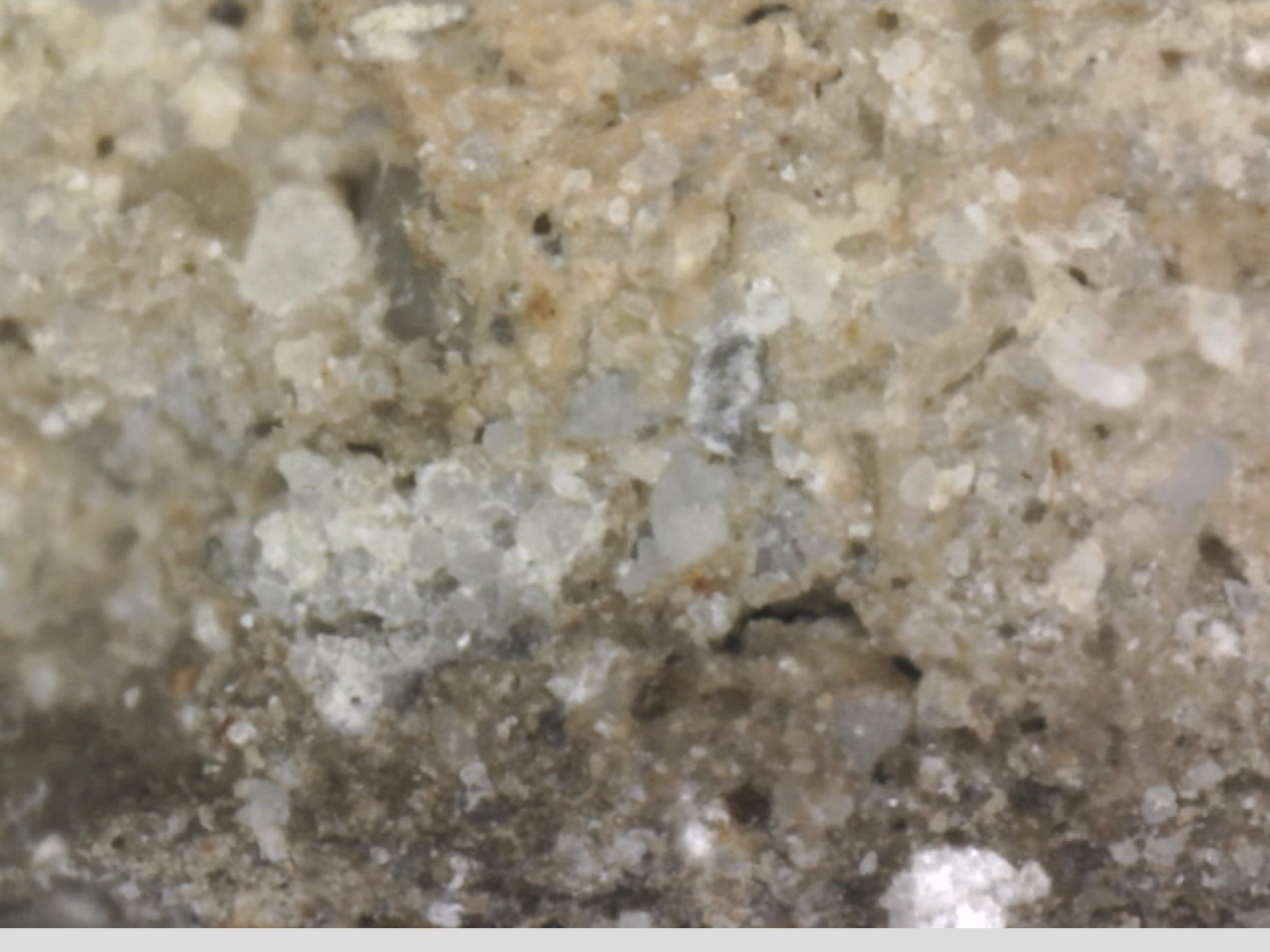

Supplement: Supplementary file 3 — Supplementary material [file mmc3.zip › Appendix A/HTN 43/HTN 43-250m-5.jpg]

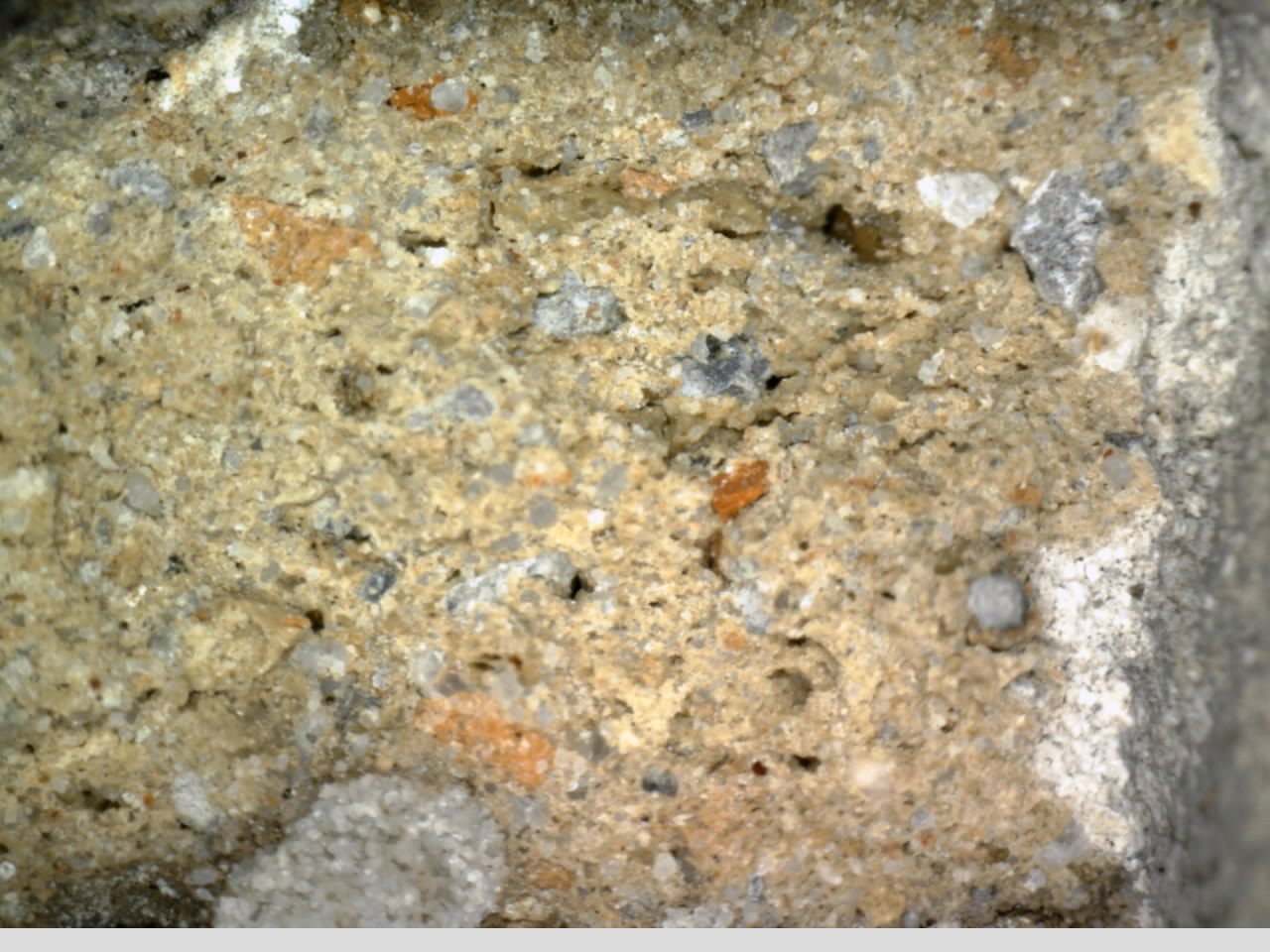

Supplement: Supplementary file 3 — Supplementary material [file mmc3.zip › Appendix A/HTN 43/HTN 43-50m-4.jpg]

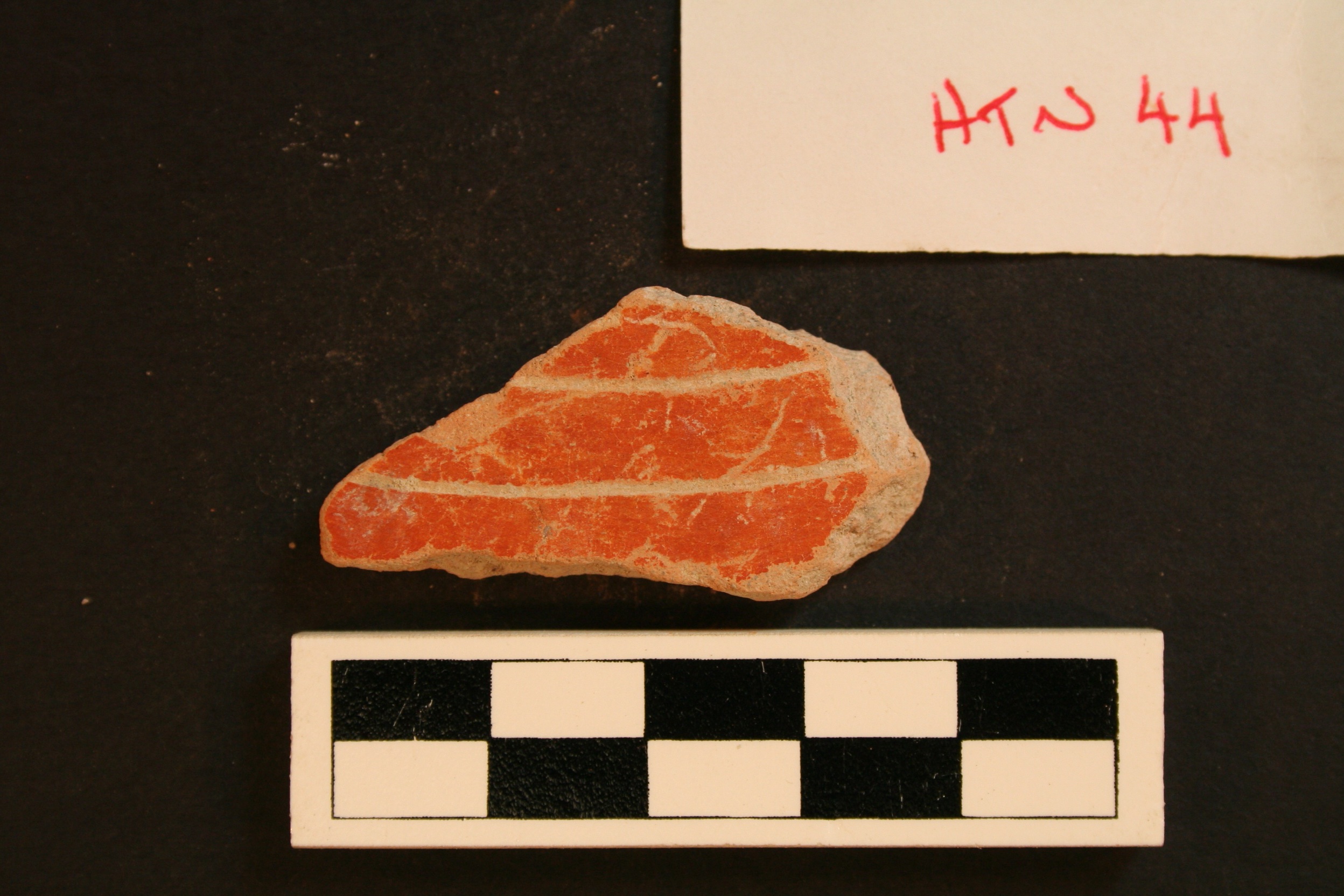

Supplement: Supplementary file 3 — Supplementary material [file mmc3.zip › Appendix A/HTN 44/44a.JPG]

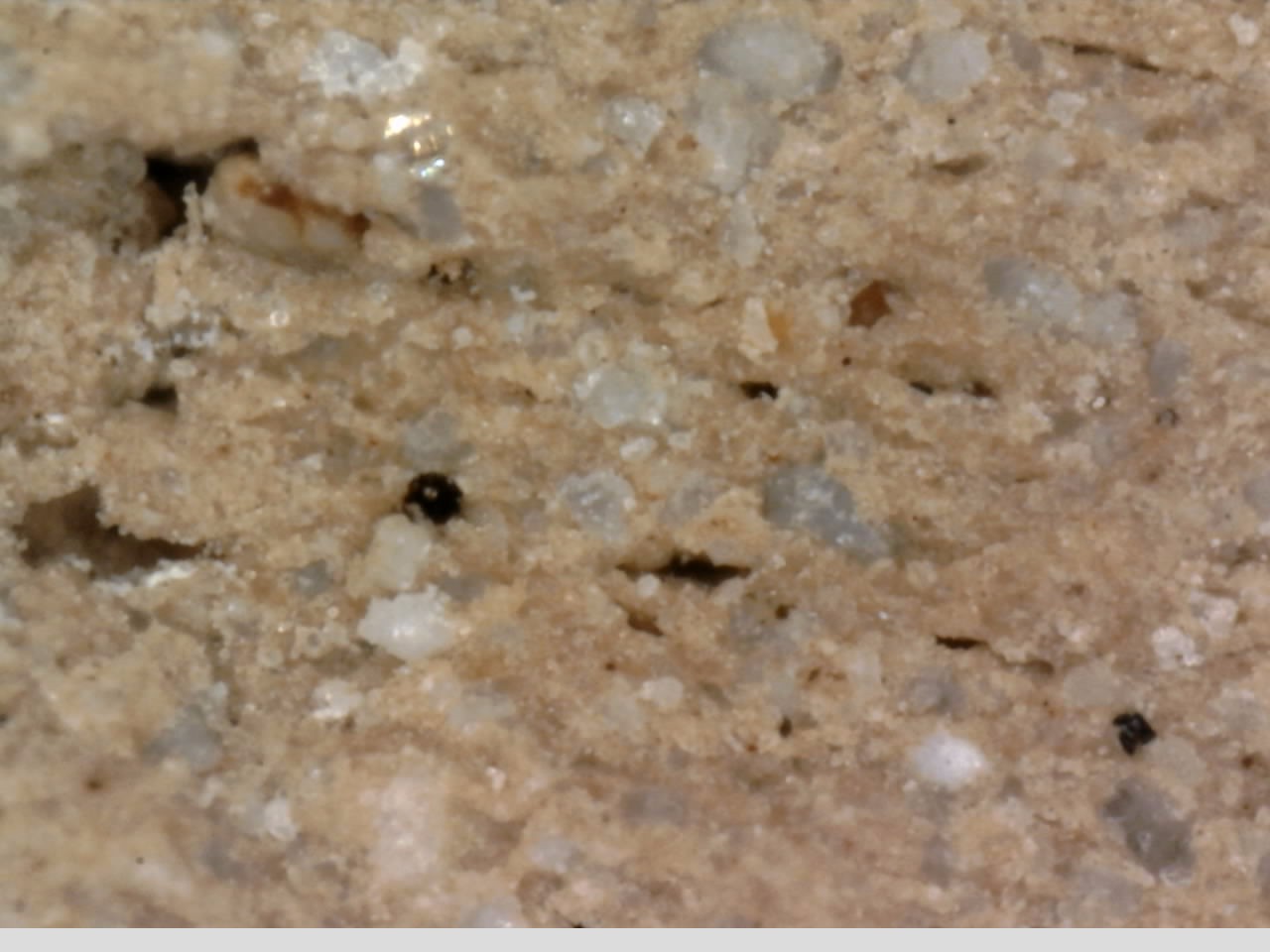

Supplement: Supplementary file 3 — Supplementary material [file mmc3.zip › Appendix A/HTN 44/HTN 44-250m-0.jpg]

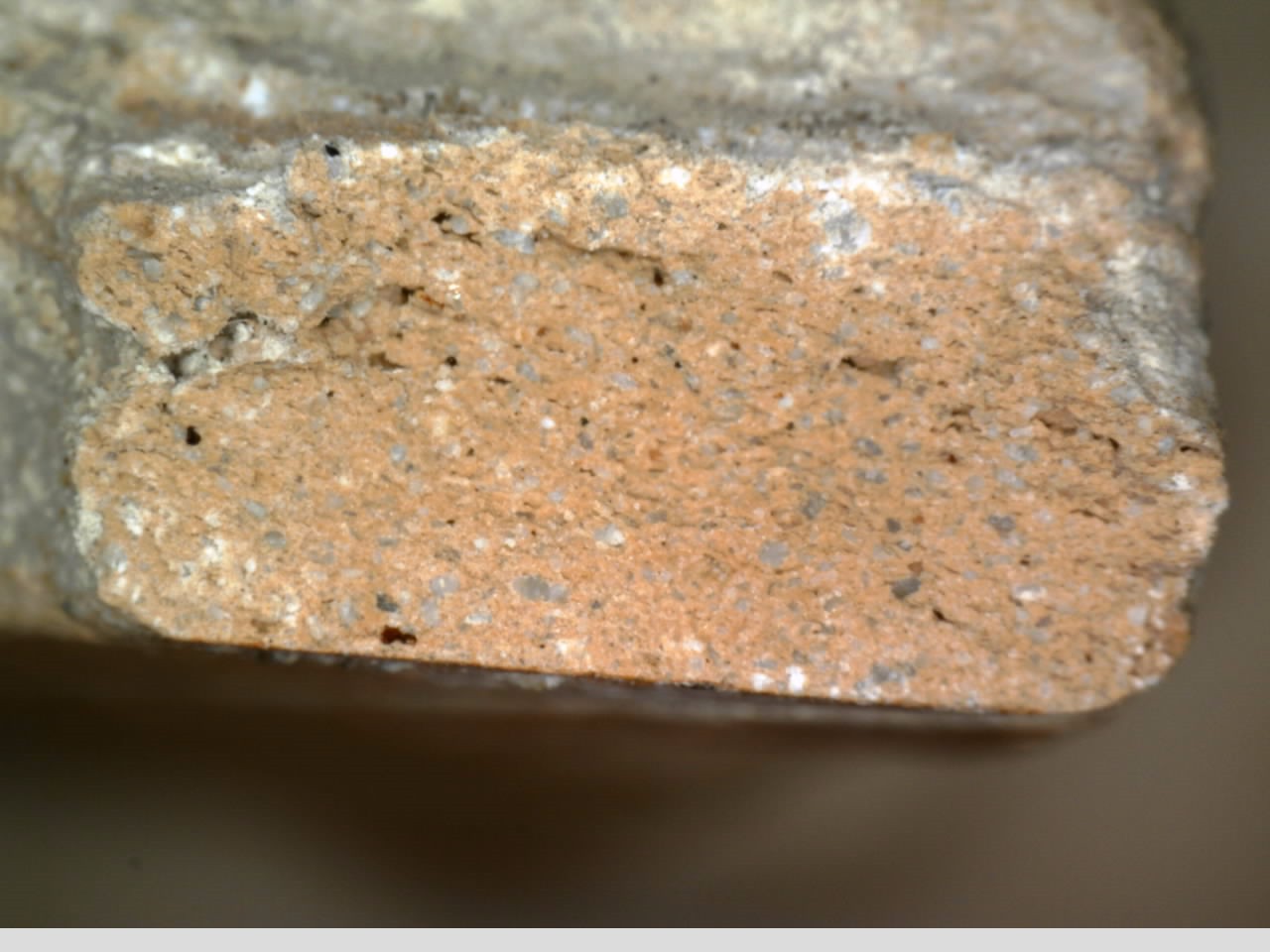

Supplement: Supplementary file 3 — Supplementary material [file mmc3.zip › Appendix A/HTN 44/HTN 44-50m-2.jpg]

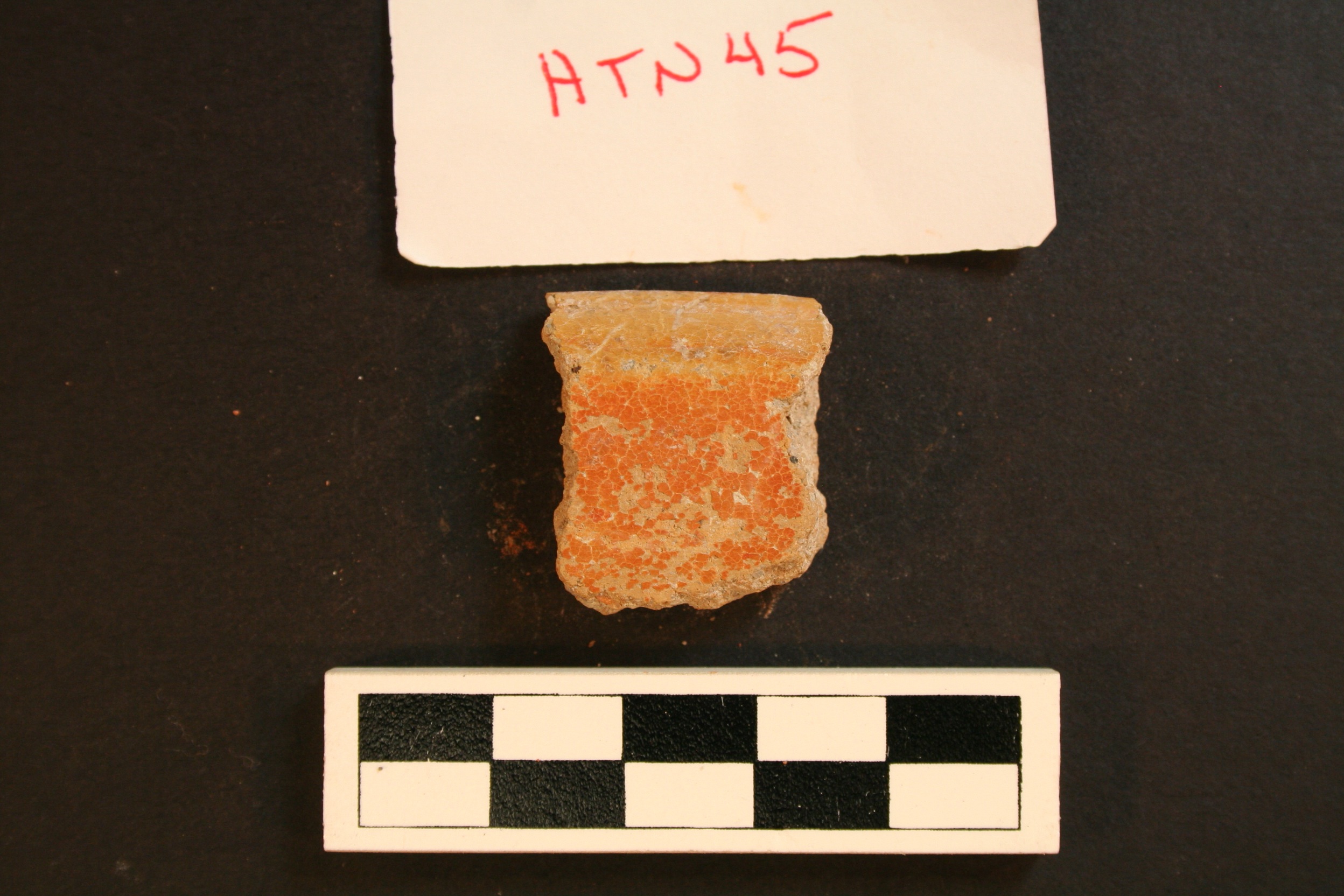

Supplement: Supplementary file 3 — Supplementary material [file mmc3.zip › Appendix A/HTN 45/45a.JPG]

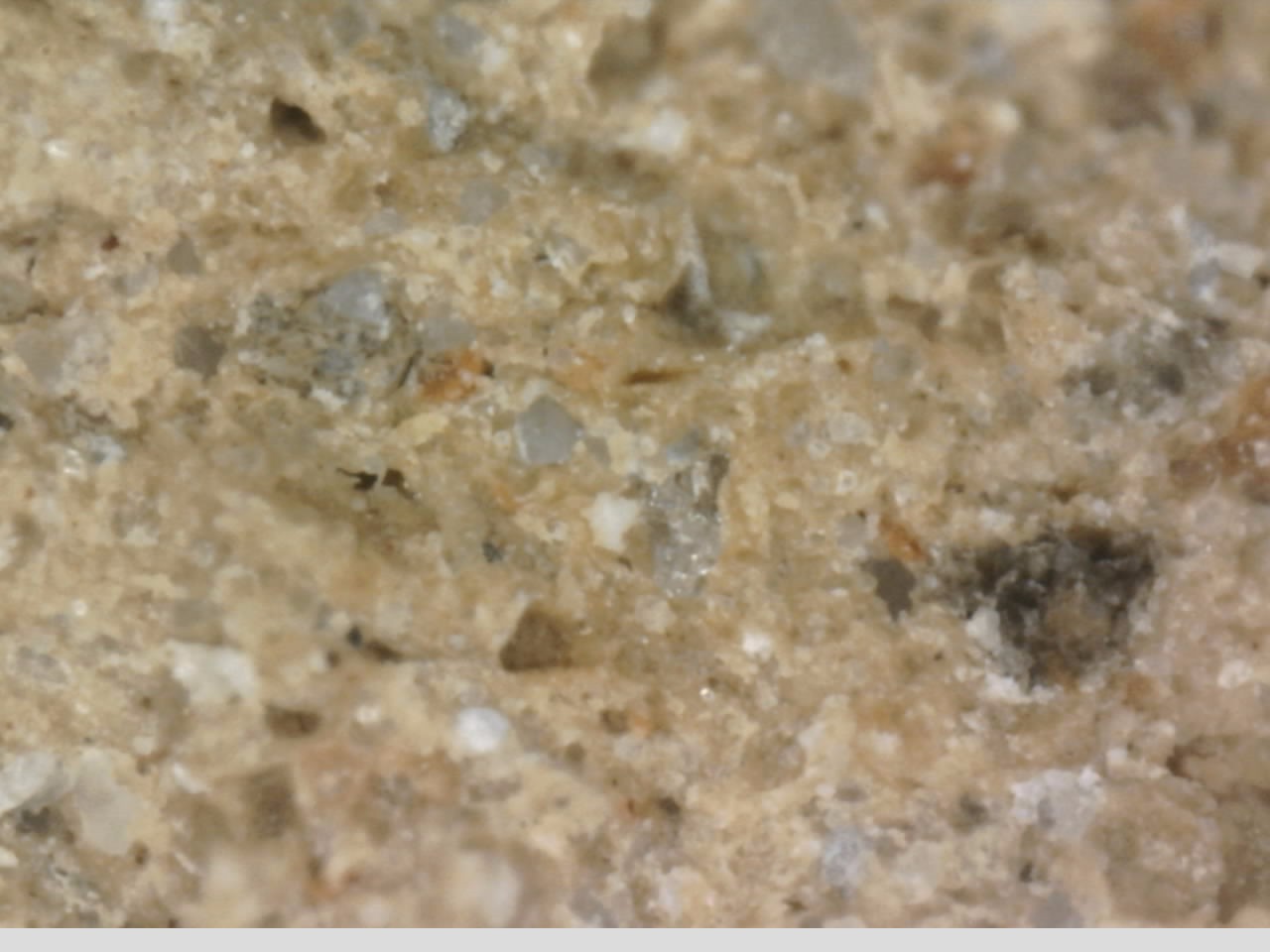

Supplement: Supplementary file 3 — Supplementary material [file mmc3.zip › Appendix A/HTN 45/HTN 45-250m-4.jpg]

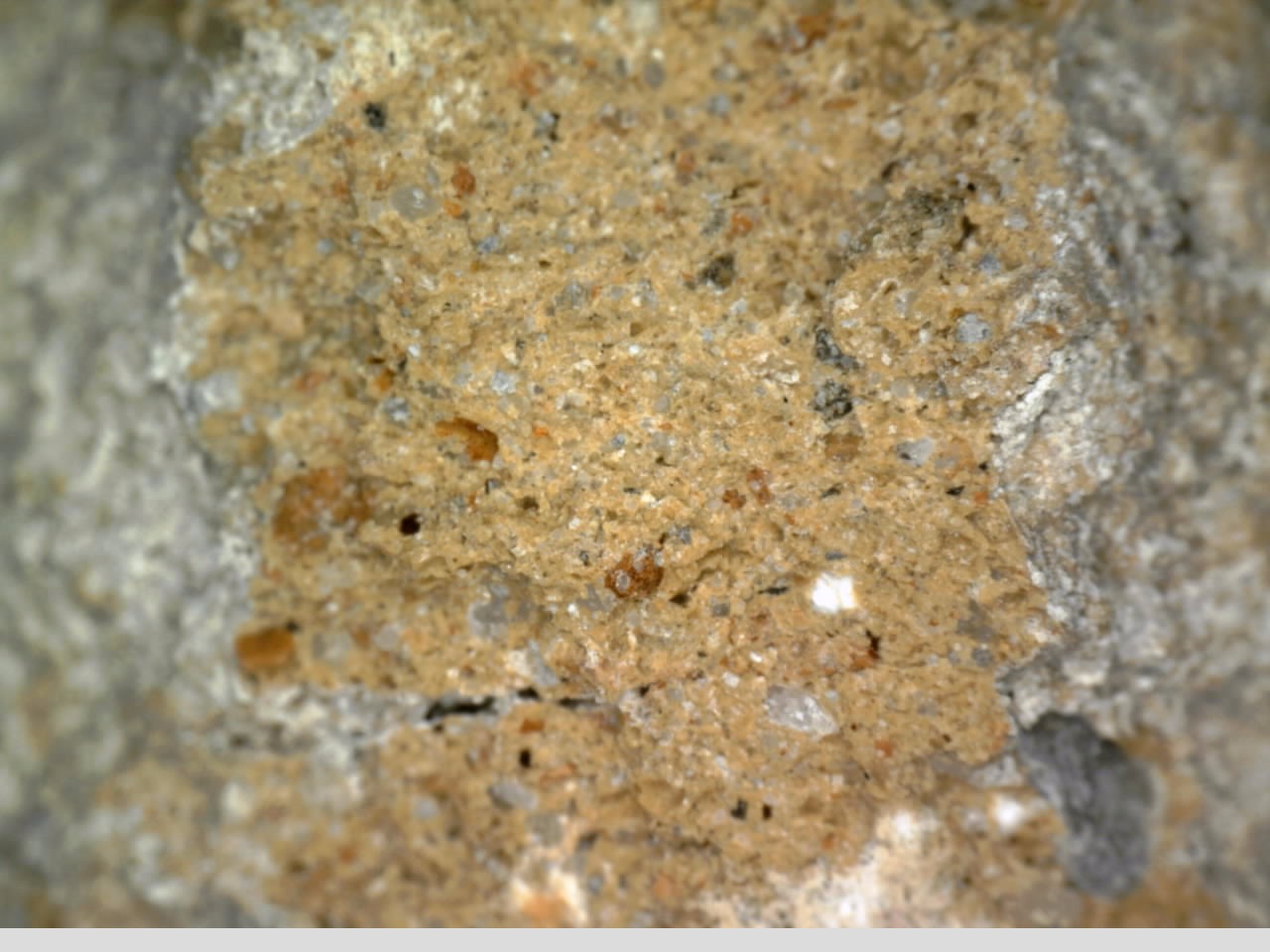

Supplement: Supplementary file 3 — Supplementary material [file mmc3.zip › Appendix A/HTN 45/HTN 45-50m-2.jpg]

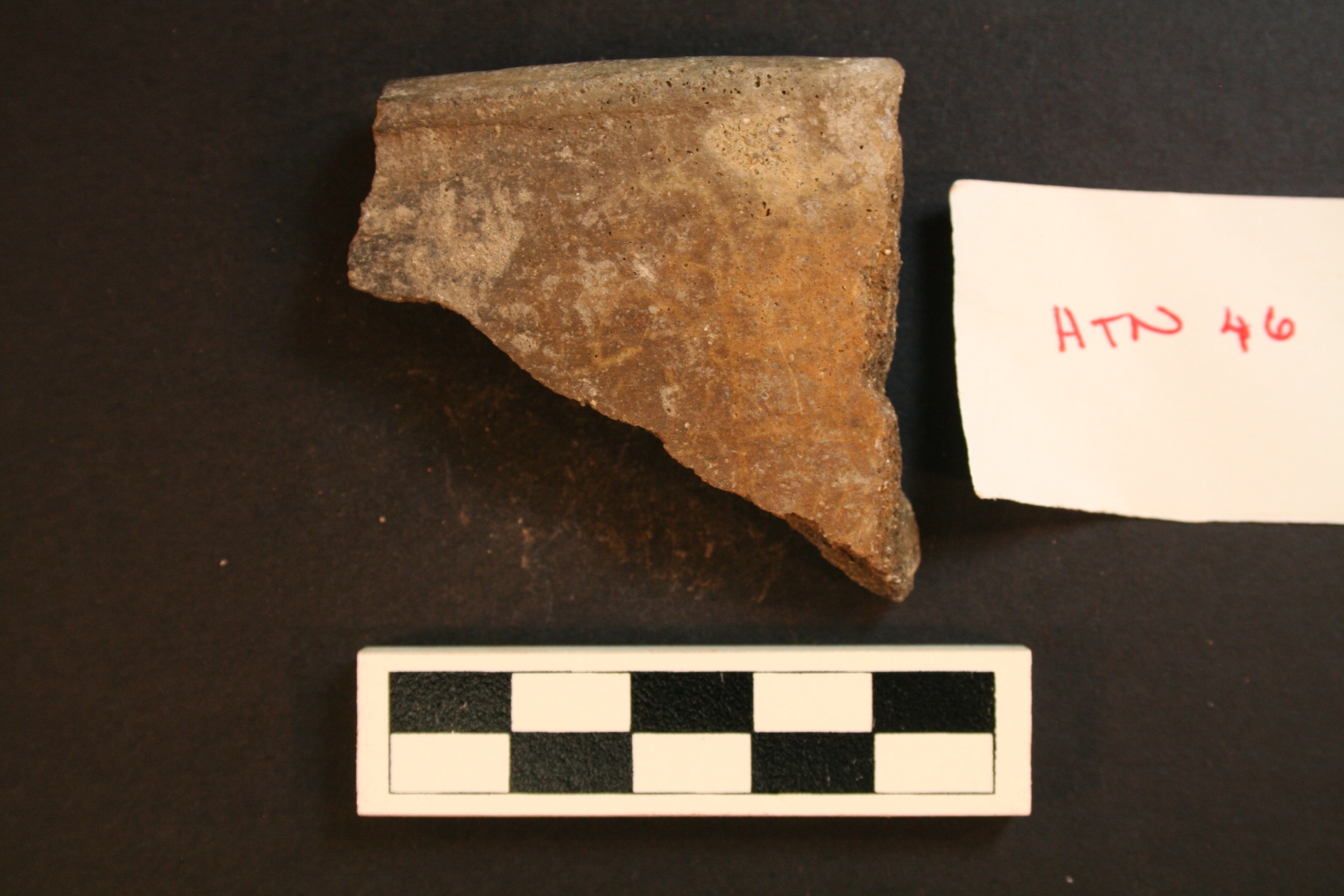

Supplement: Supplementary file 3 — Supplementary material [file mmc3.zip › Appendix A/HTN 46/46a.JPG]

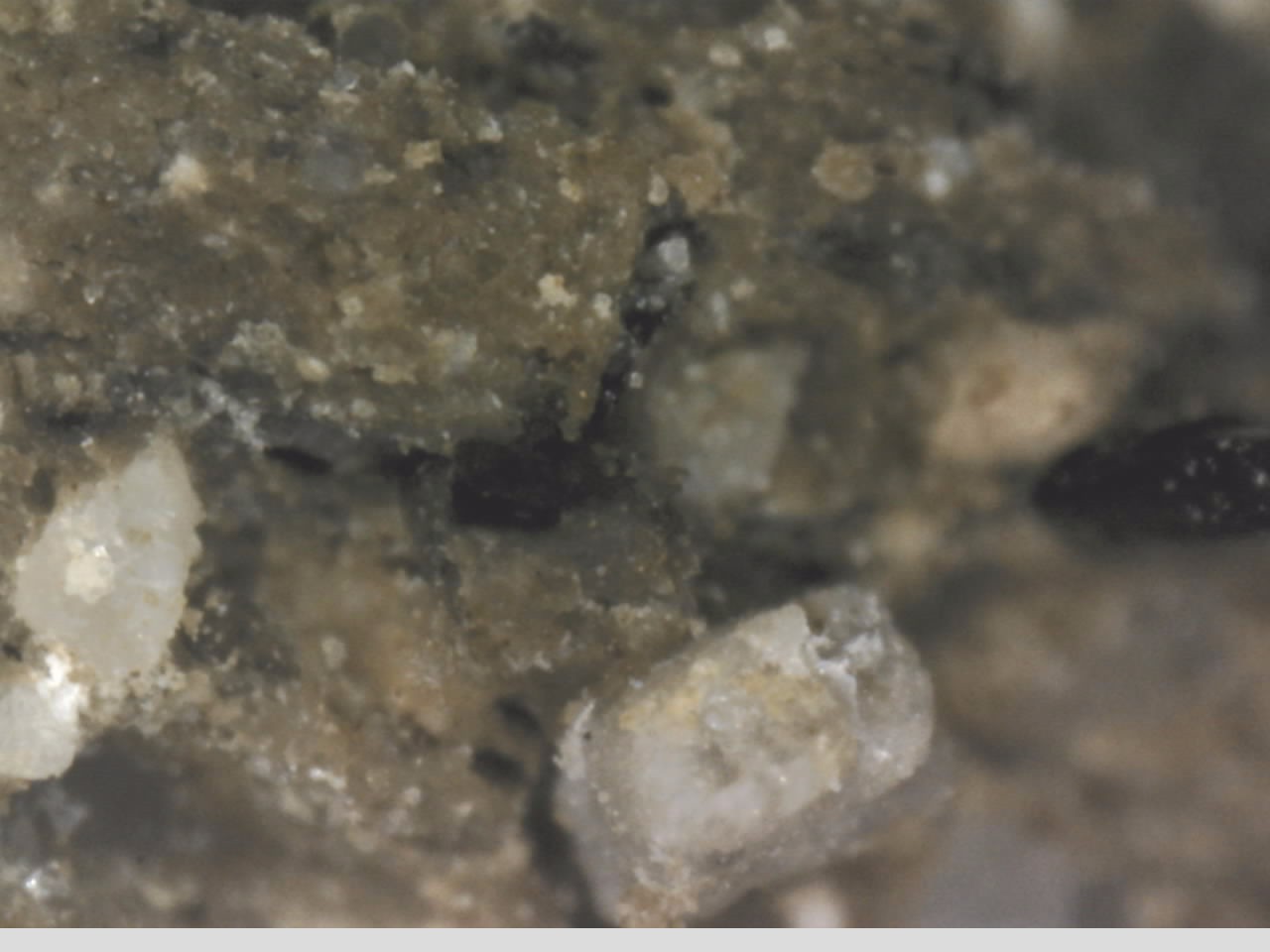

Supplement: Supplementary file 3 — Supplementary material [file mmc3.zip › Appendix A/HTN 46/HTN 46-250m-1.jpg]

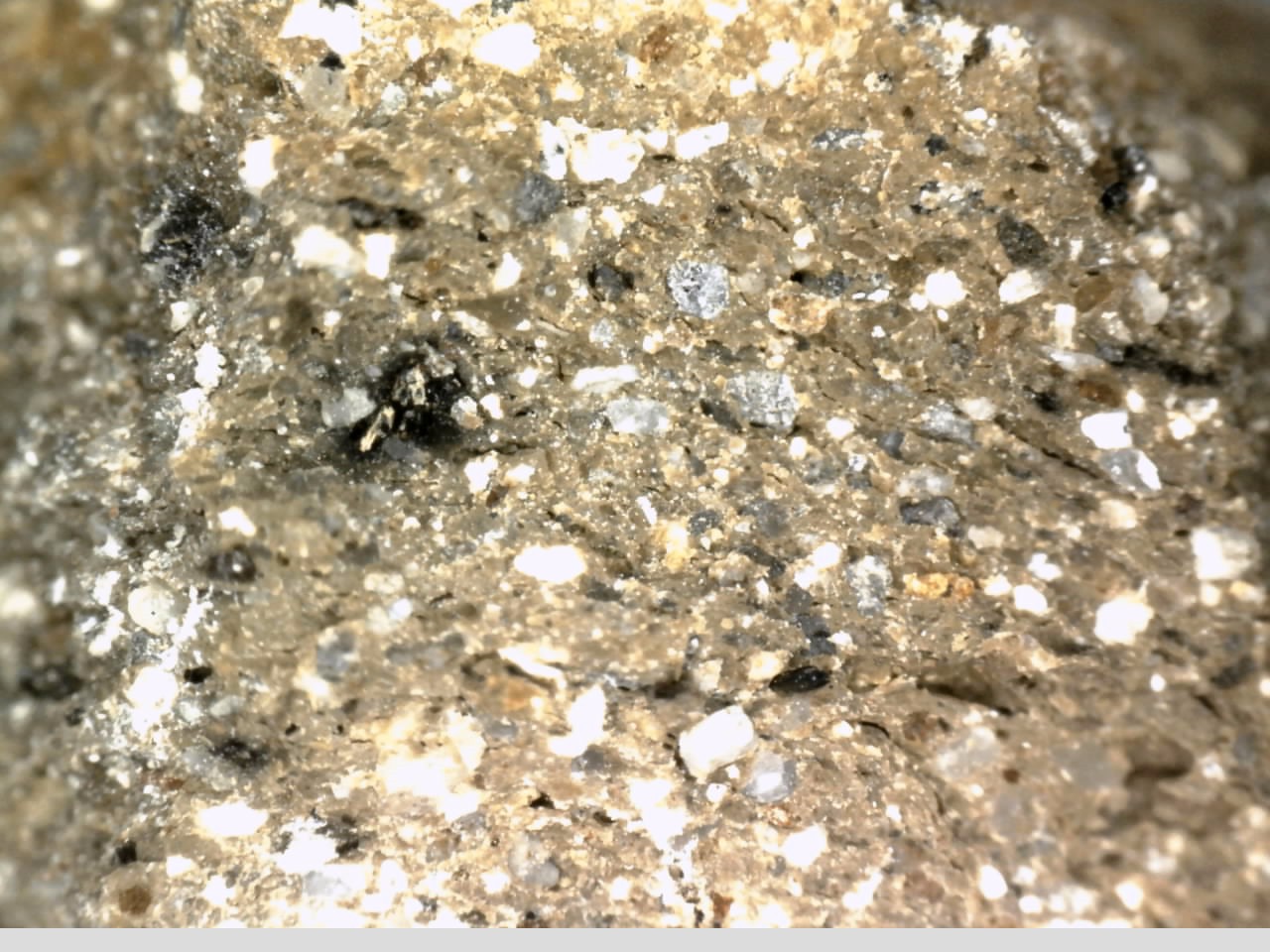

Supplement: Supplementary file 3 — Supplementary material [file mmc3.zip › Appendix A/HTN 46/HTN 46-50m-1.jpg]

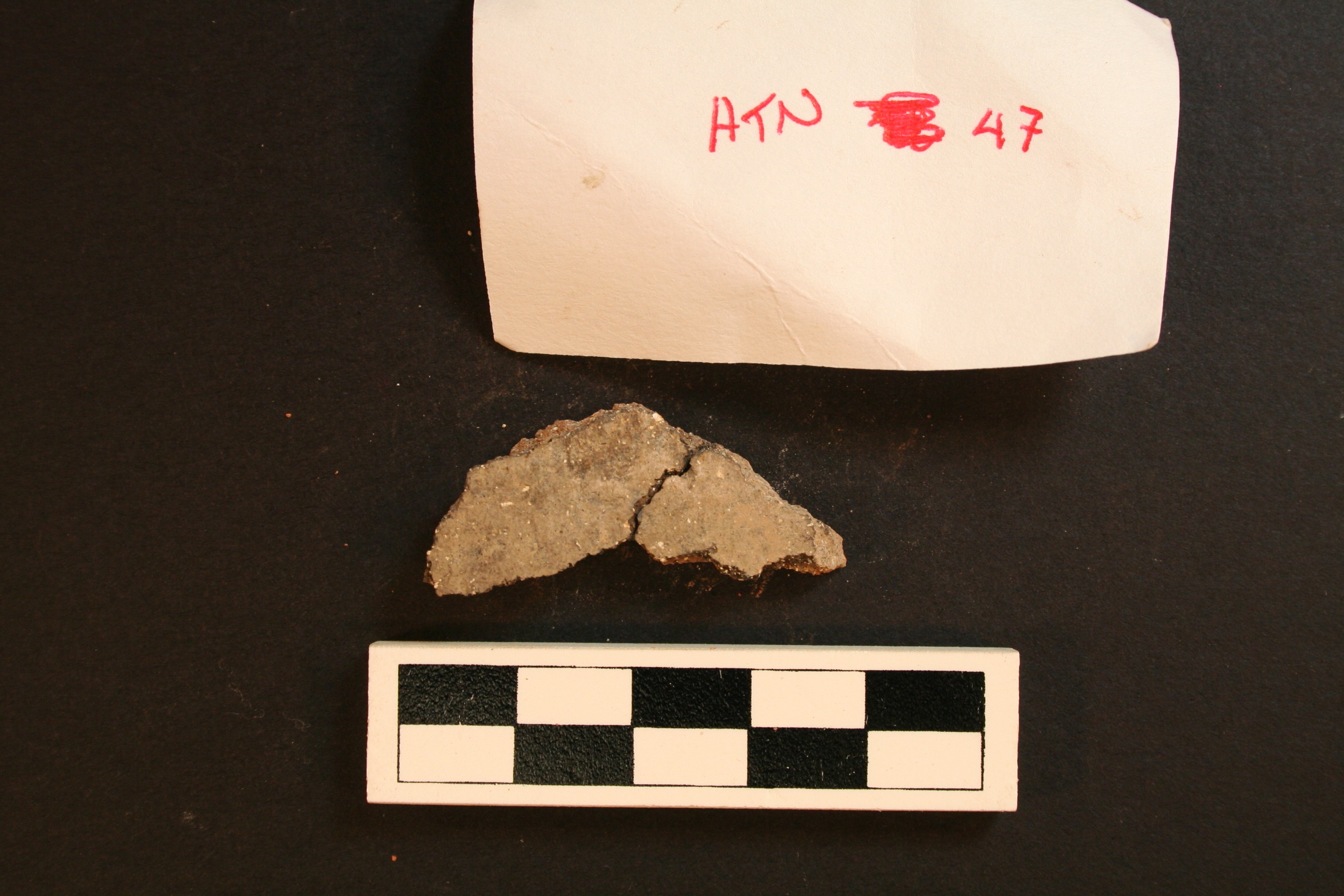

Supplement: Supplementary file 3 — Supplementary material [file mmc3.zip › Appendix A/HTN 47/47a.JPG]

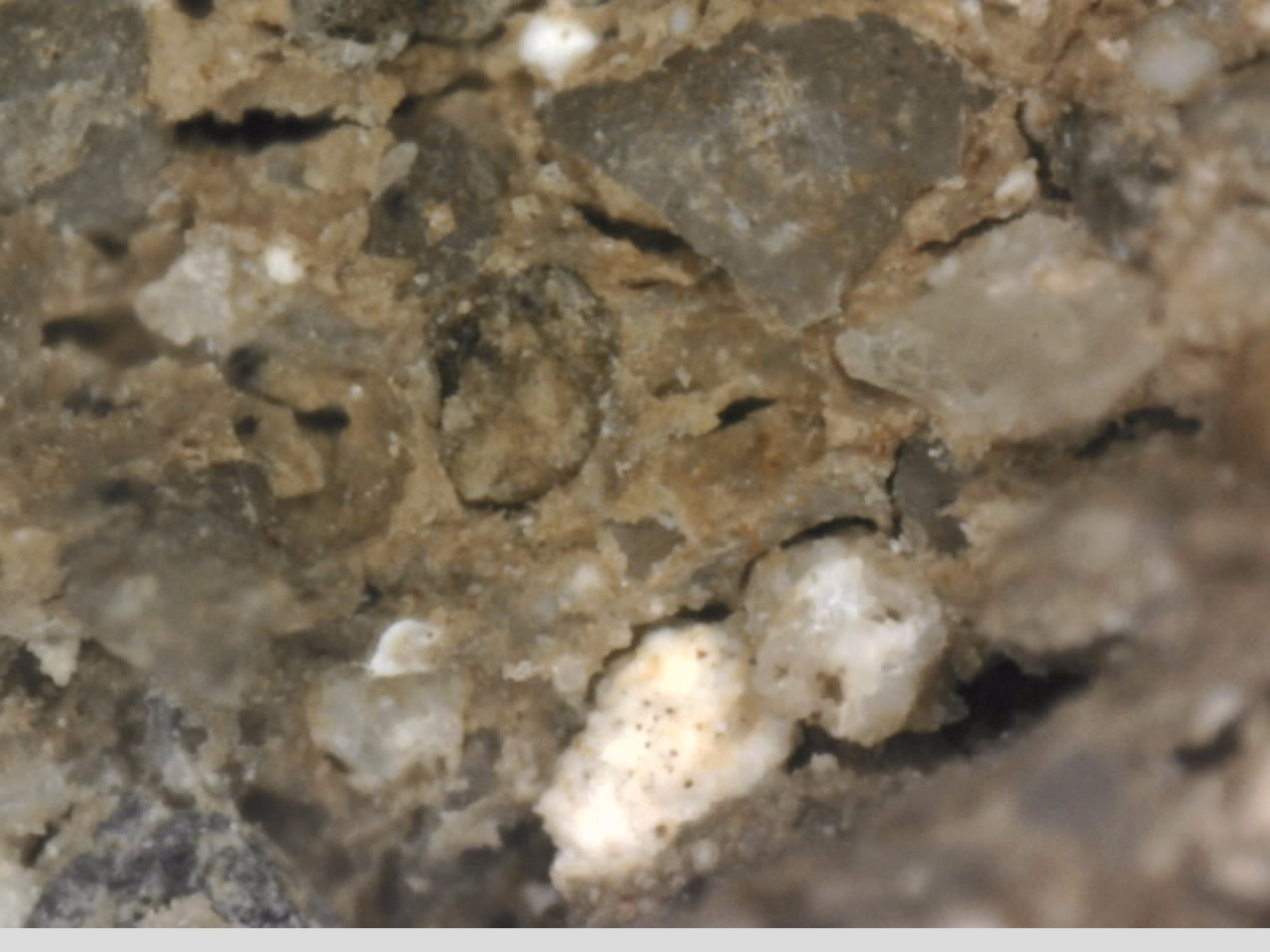

Supplement: Supplementary file 3 — Supplementary material [file mmc3.zip › Appendix A/HTN 47/HTN 47-250m-0.jpg]

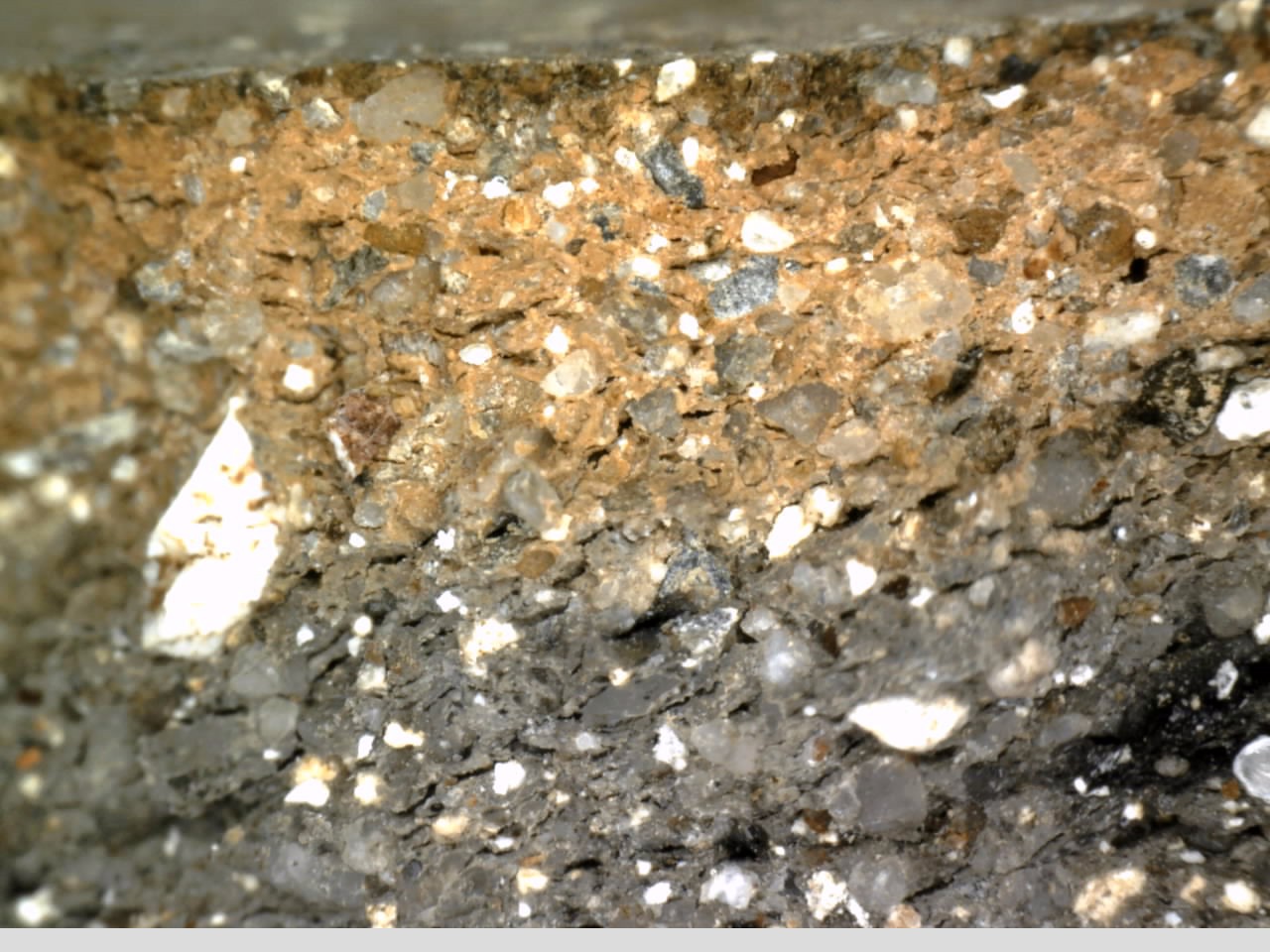

Supplement: Supplementary file 3 — Supplementary material [file mmc3.zip › Appendix A/HTN 47/HTN 47-50m-1.jpg]

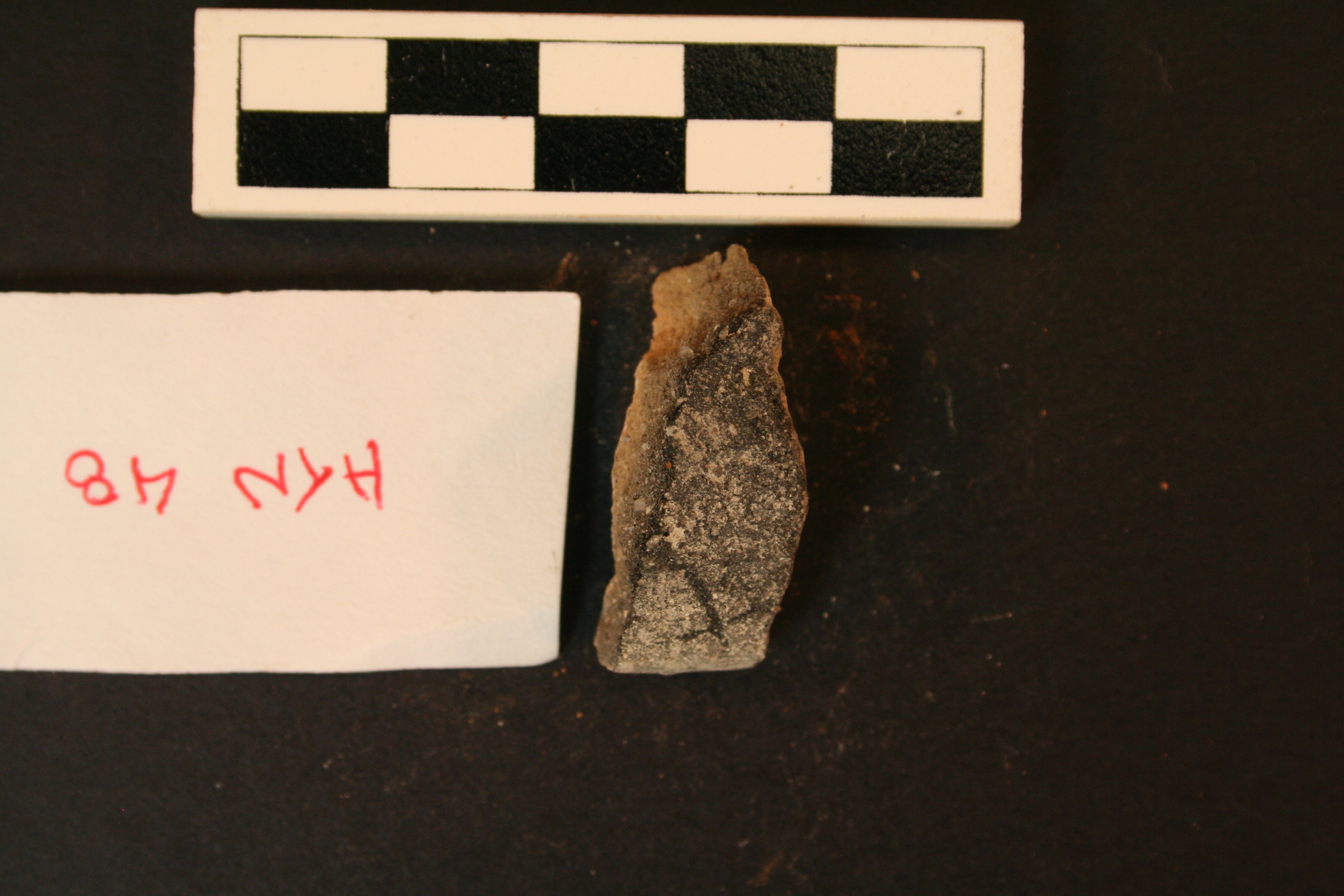

Supplement: Supplementary file 3 — Supplementary material [file mmc3.zip › Appendix A/HTN 48/48a.JPG]

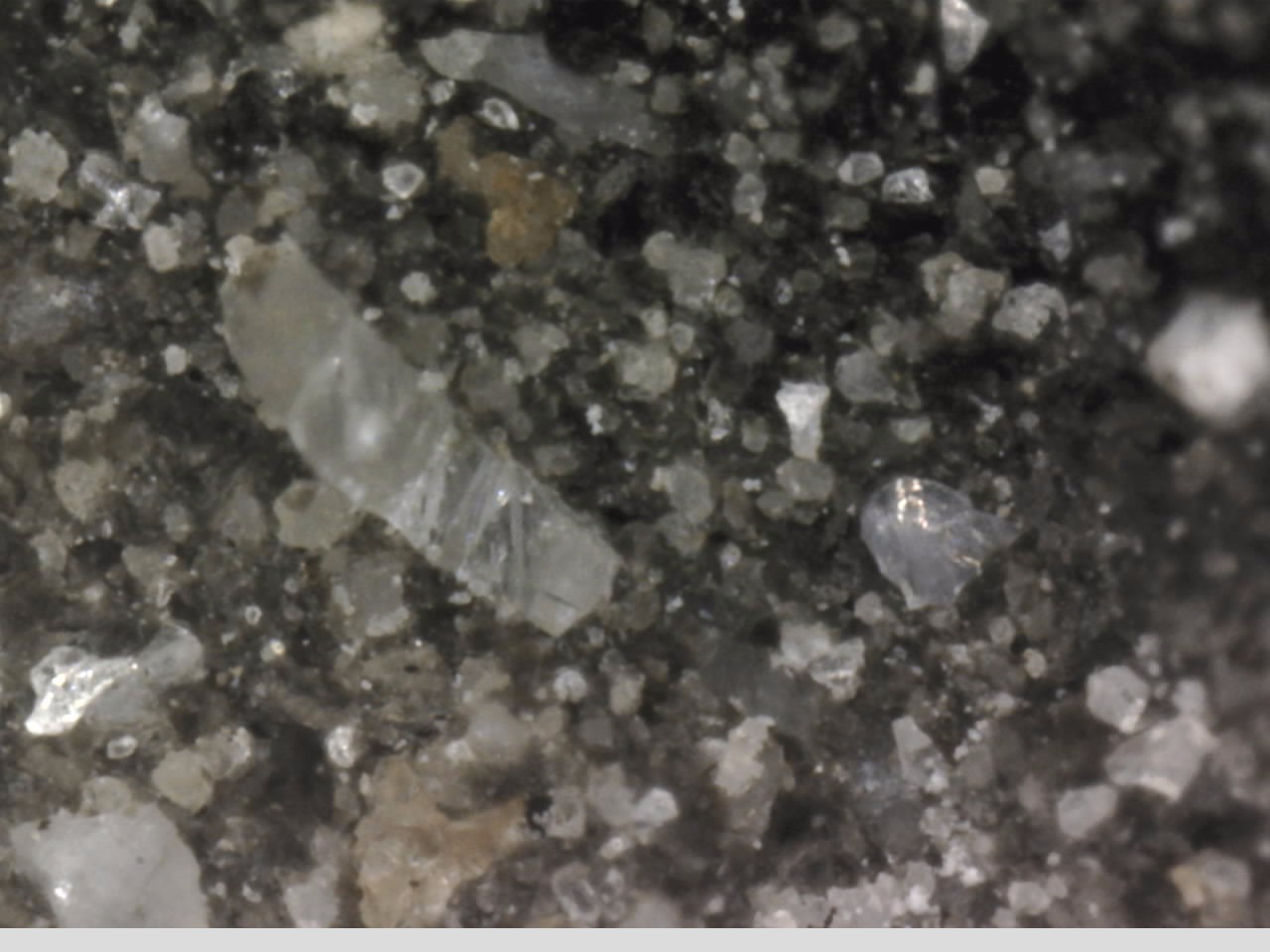

Supplement: Supplementary file 3 — Supplementary material [file mmc3.zip › Appendix A/HTN 48/HTN 48-250m-0.jpg]

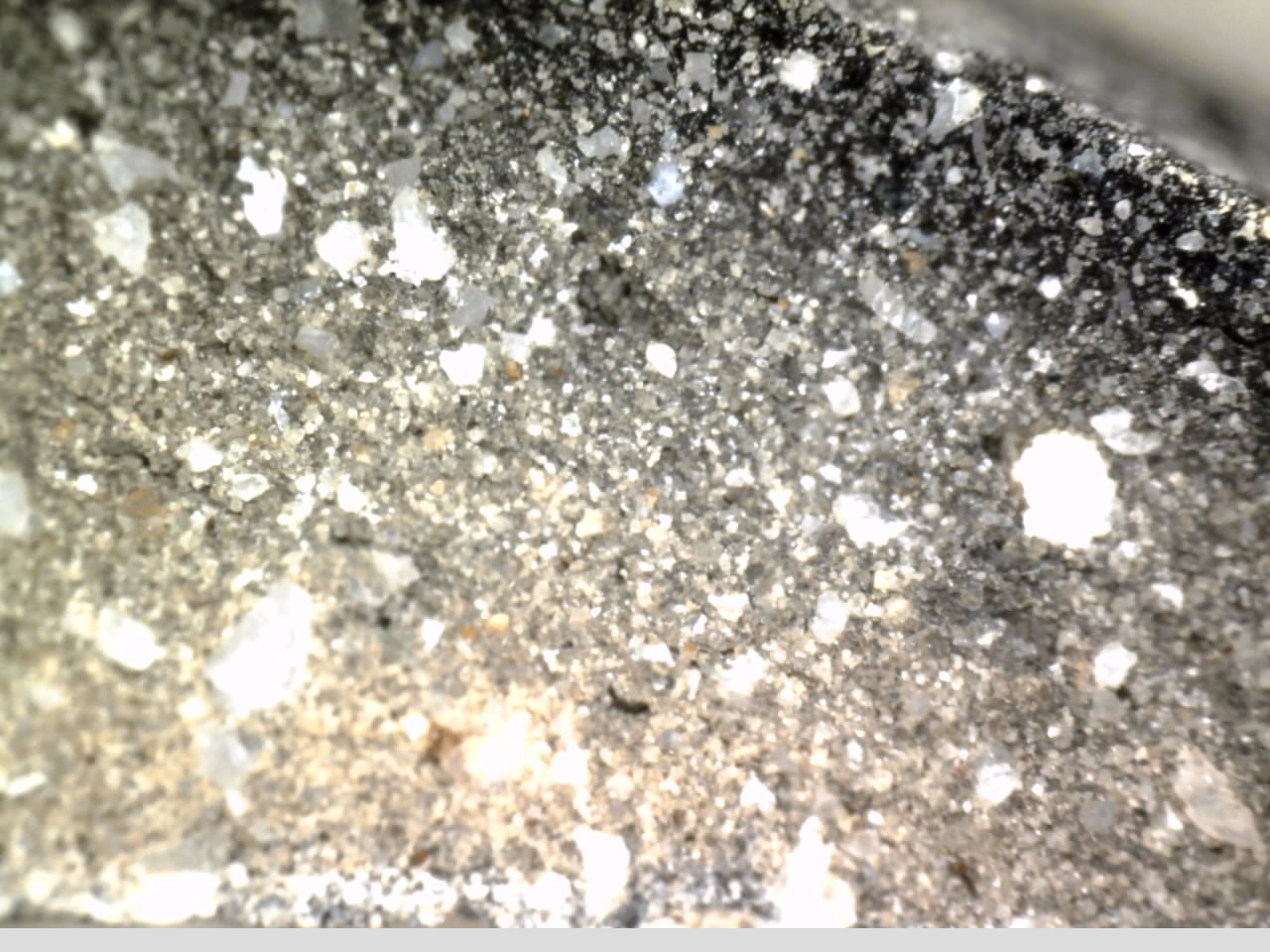

Supplement: Supplementary file 3 — Supplementary material [file mmc3.zip › Appendix A/HTN 48/HTN 48-50m-4.jpg]

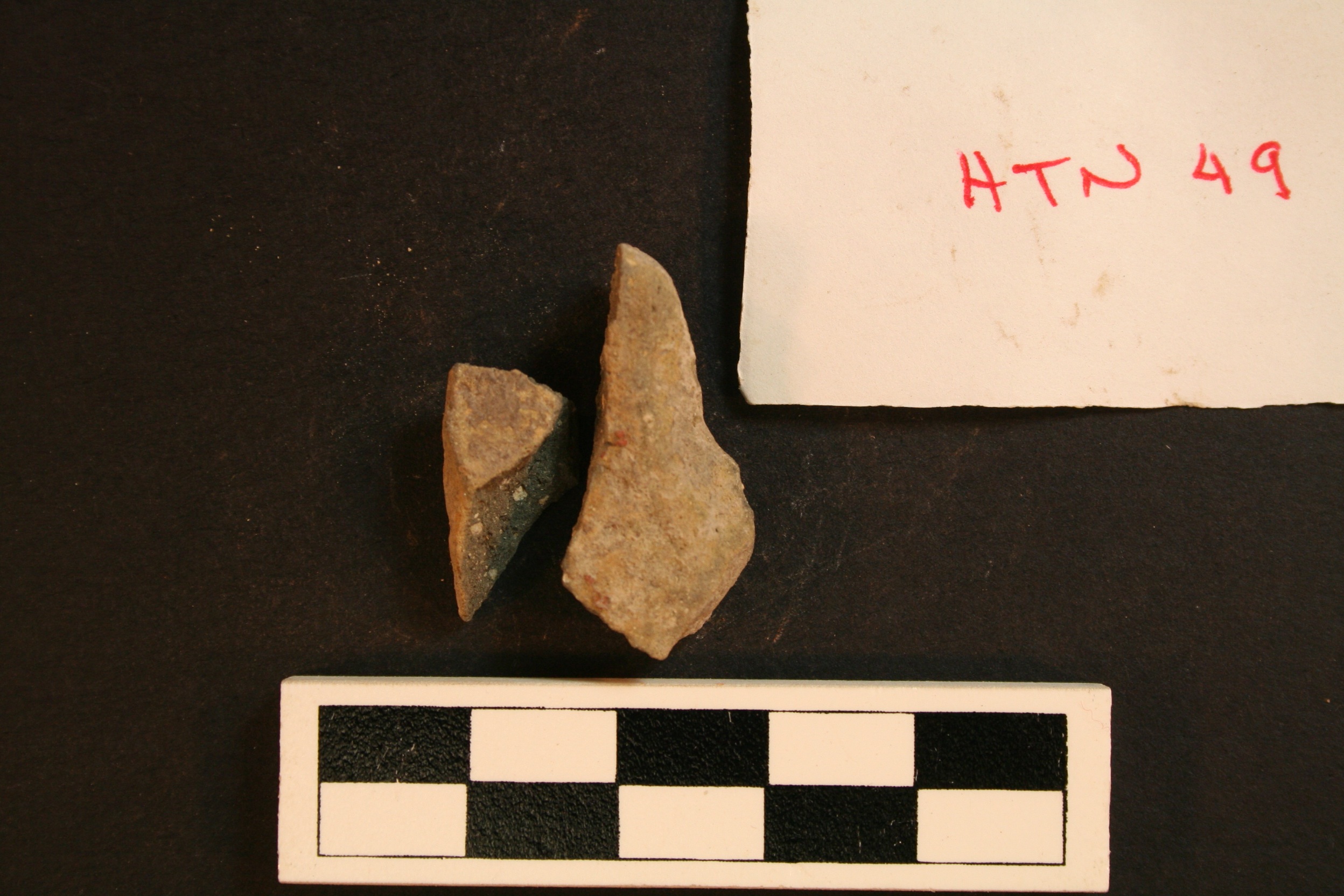

Supplement: Supplementary file 3 — Supplementary material [file mmc3.zip › Appendix A/HTN 49/49a.JPG]

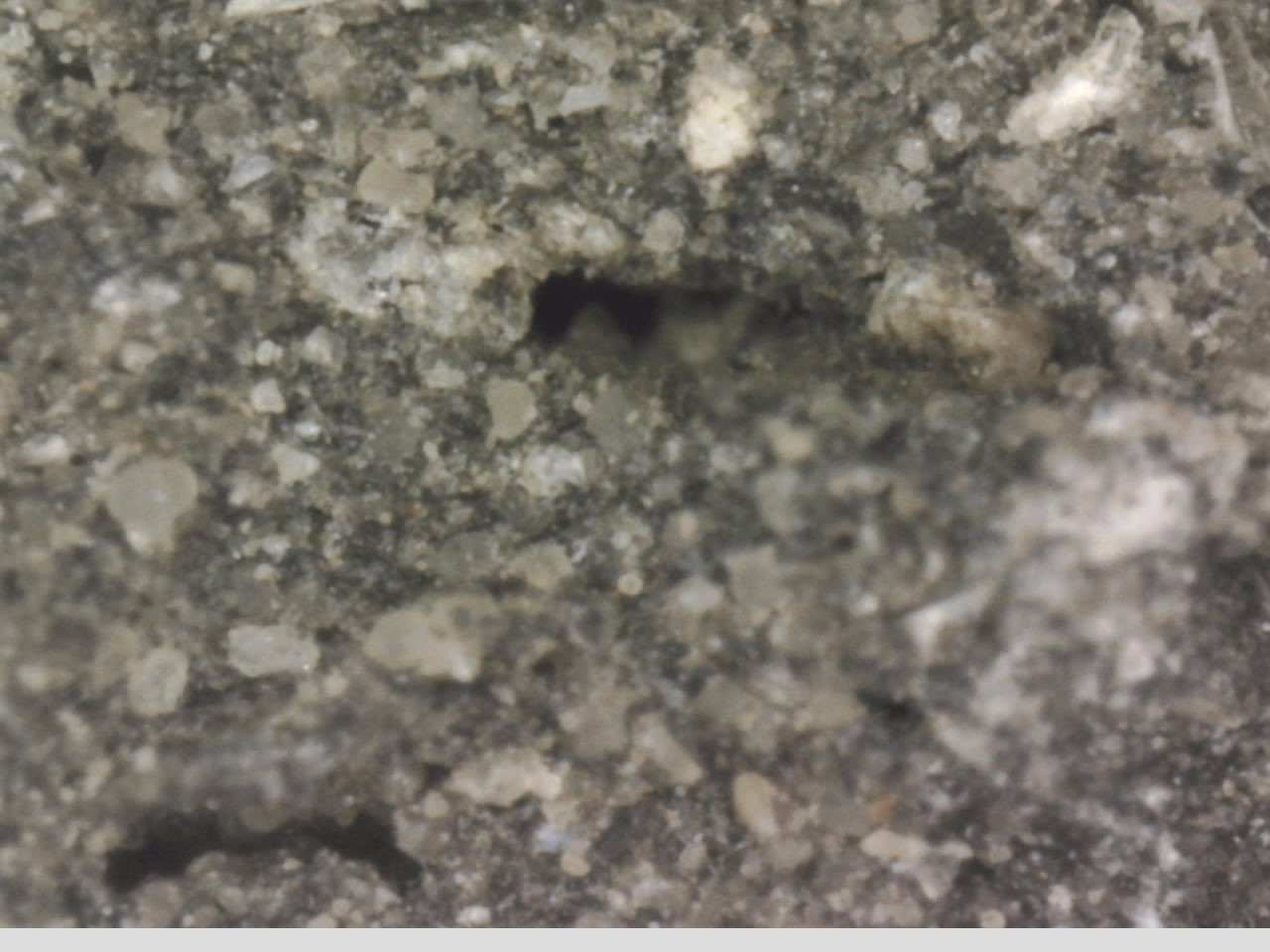

Supplement: Supplementary file 3 — Supplementary material [file mmc3.zip › Appendix A/HTN 49/HTN 49-250m-3.jpg]

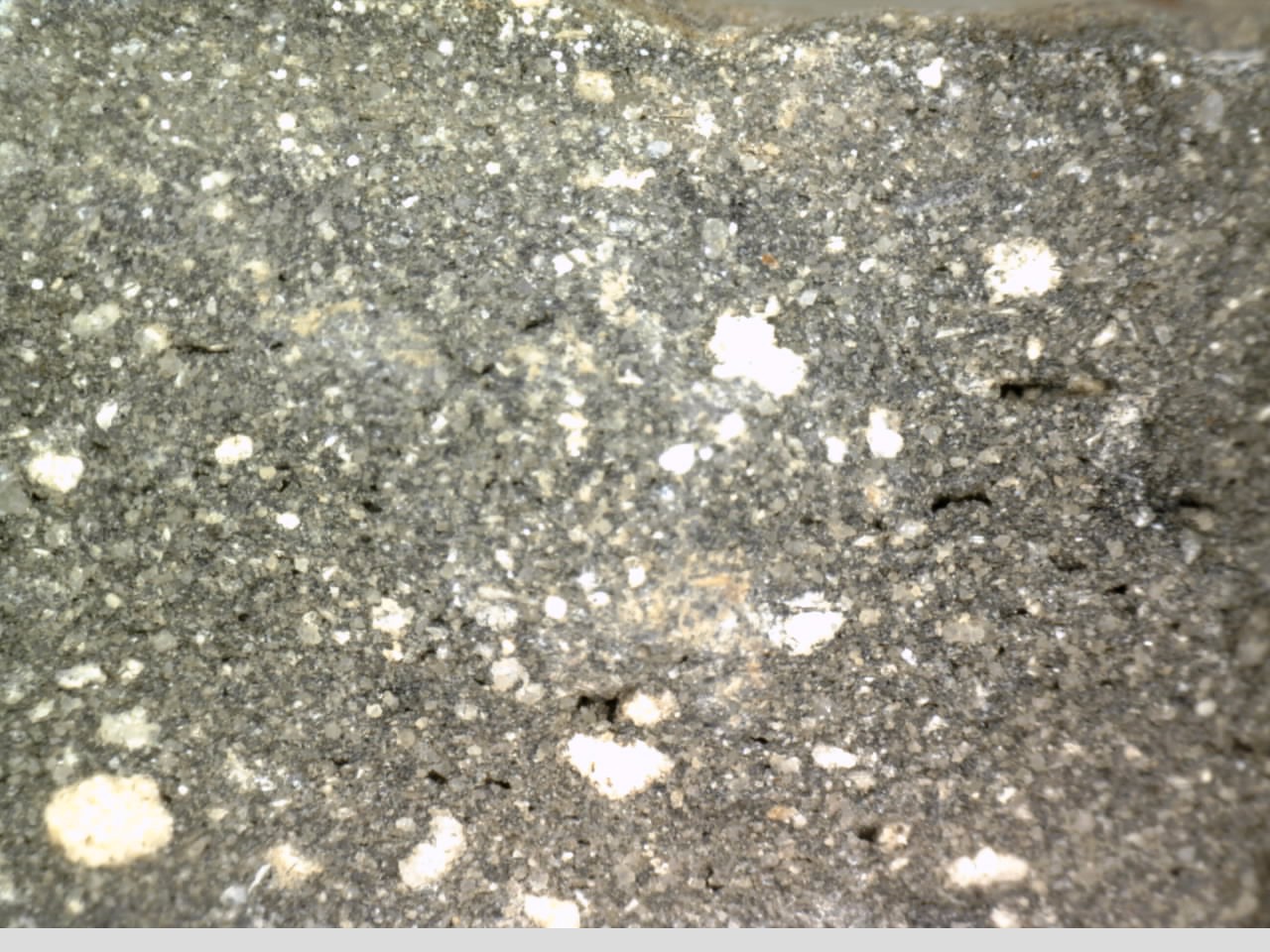

Supplement: Supplementary file 3 — Supplementary material [file mmc3.zip › Appendix A/HTN 49/HTN 49-50m-4.jpg]

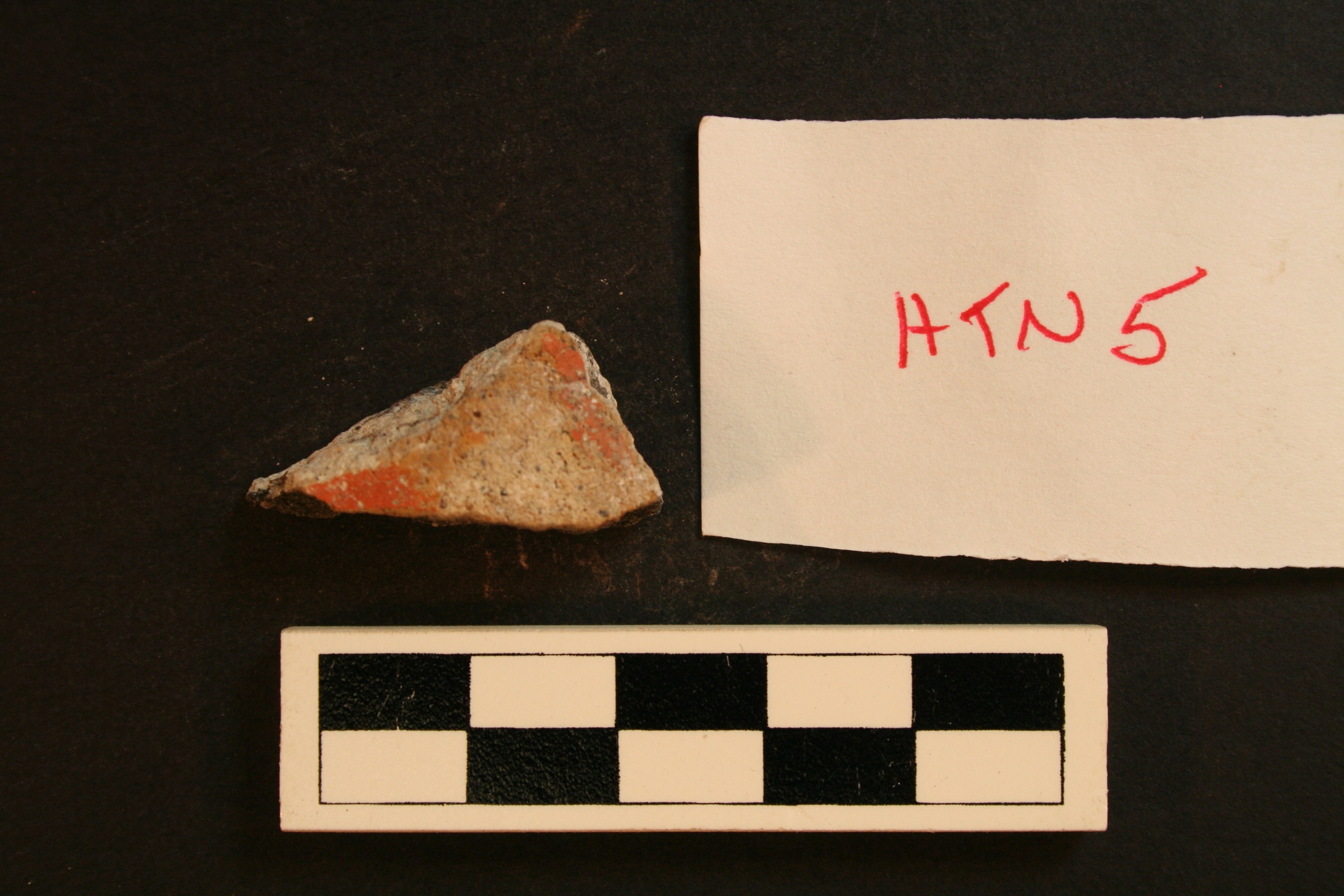

Supplement: Supplementary file 3 — Supplementary material [file mmc3.zip › Appendix A/HTN 5/5a.JPG]

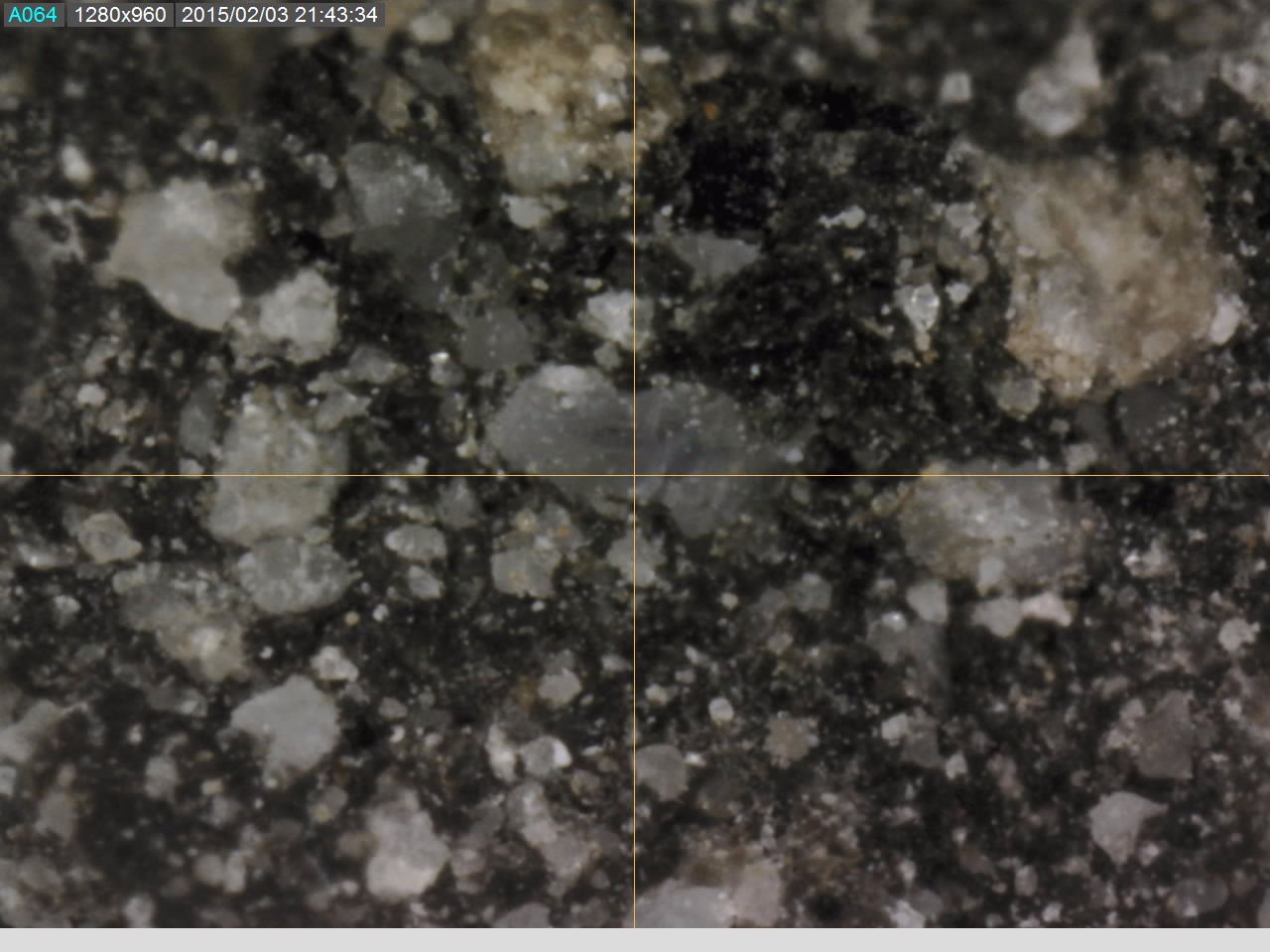

Supplement: Supplementary file 3 — Supplementary material [file mmc3.zip › Appendix A/HTN 5/HTN 5-250m-1.jpg]

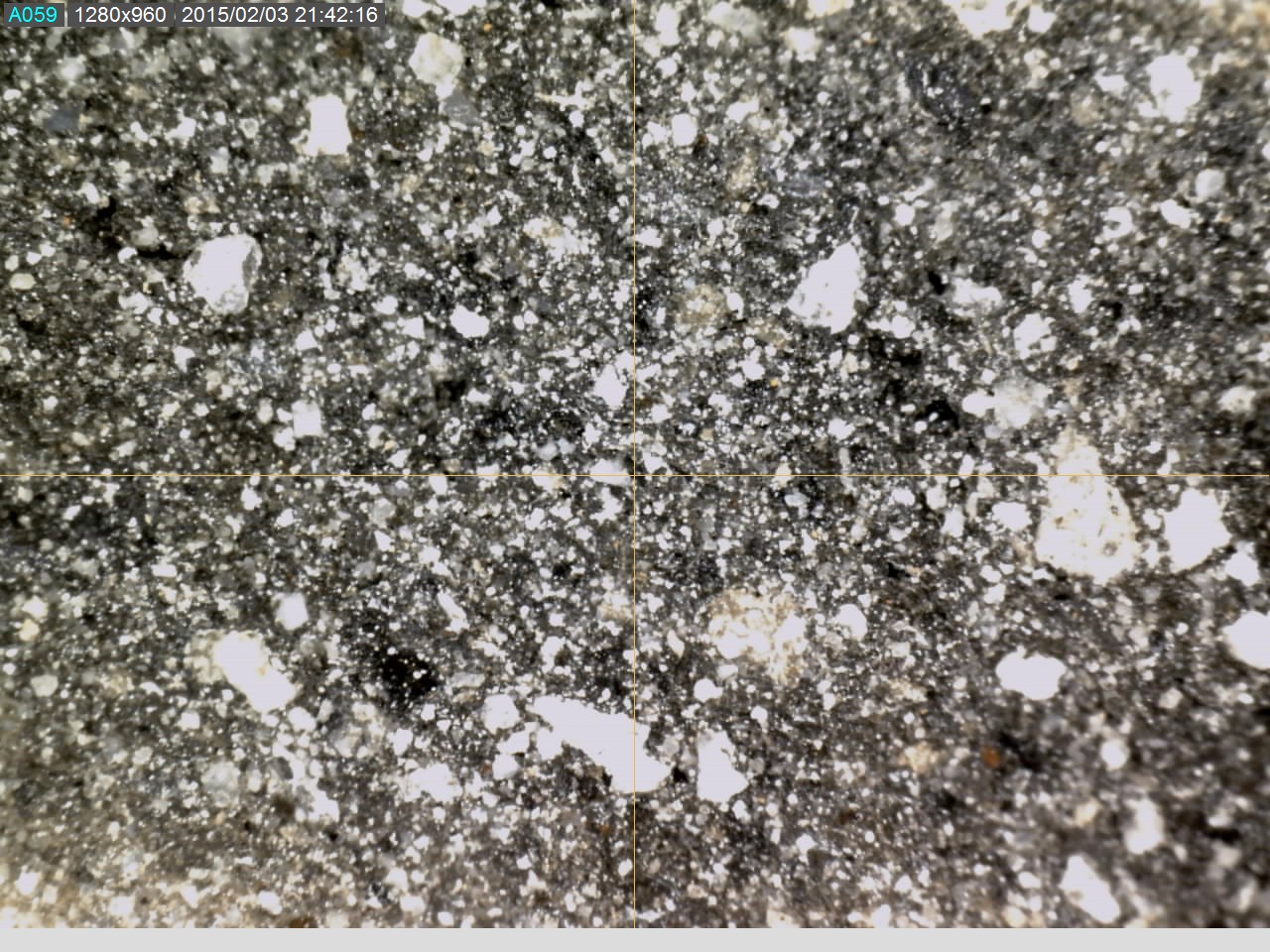

Supplement: Supplementary file 3 — Supplementary material [file mmc3.zip › Appendix A/HTN 5/HTN 5-50m-2.jpg]

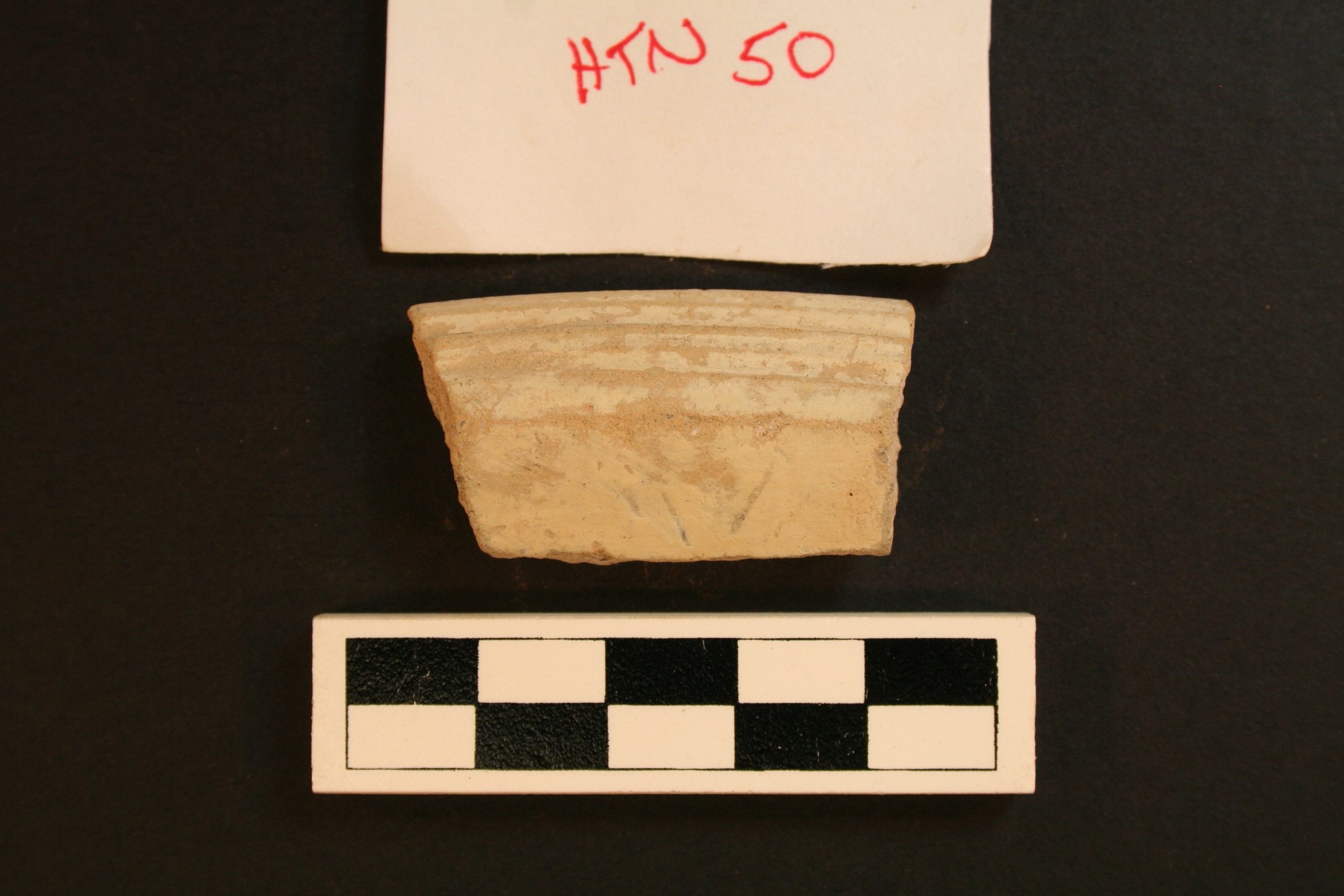

Supplement: Supplementary file 3 — Supplementary material [file mmc3.zip › Appendix A/HTN 50/50a.JPG]

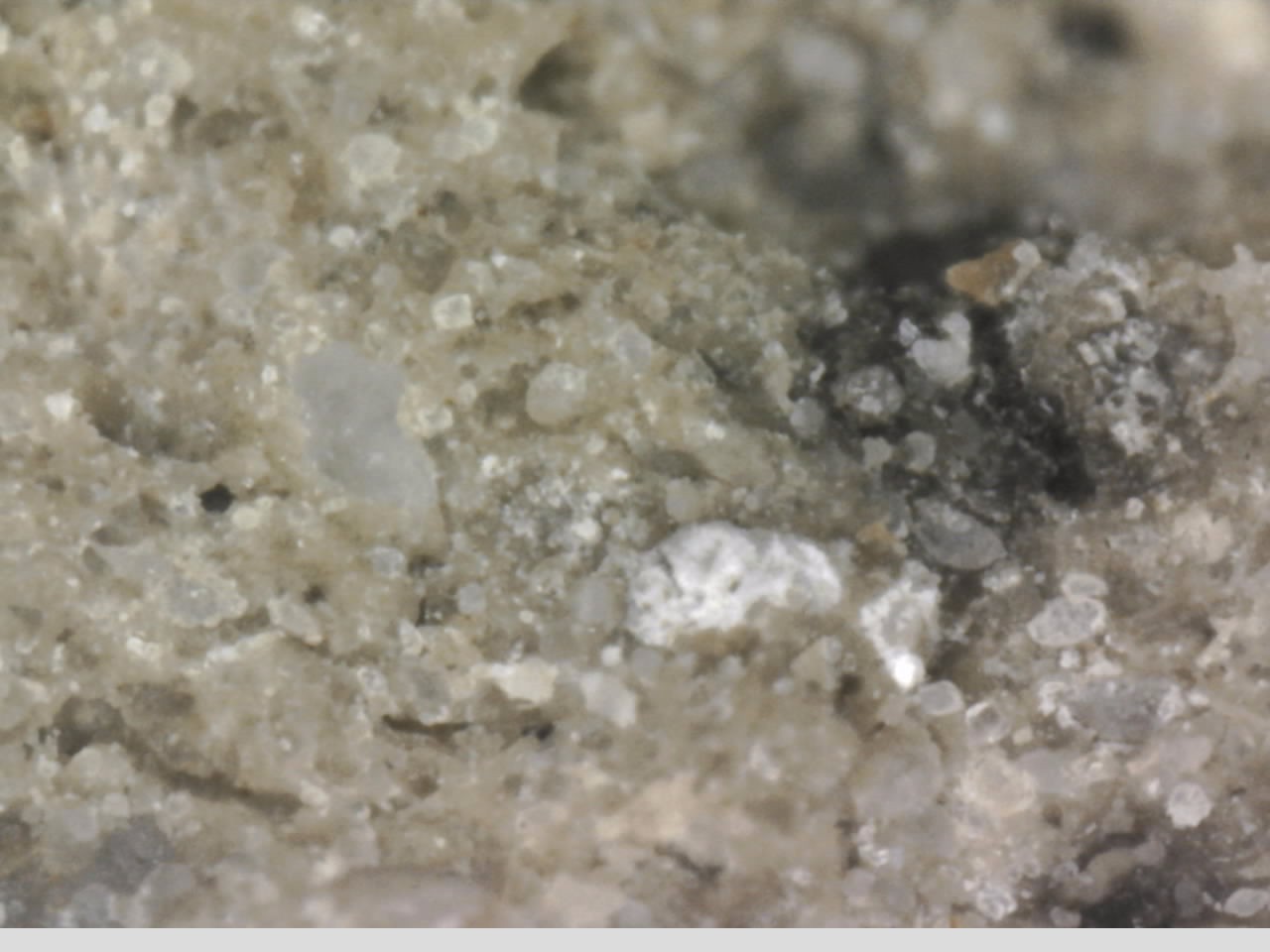

Supplement: Supplementary file 3 — Supplementary material [file mmc3.zip › Appendix A/HTN 50/HTN 50-250m-5.jpg]

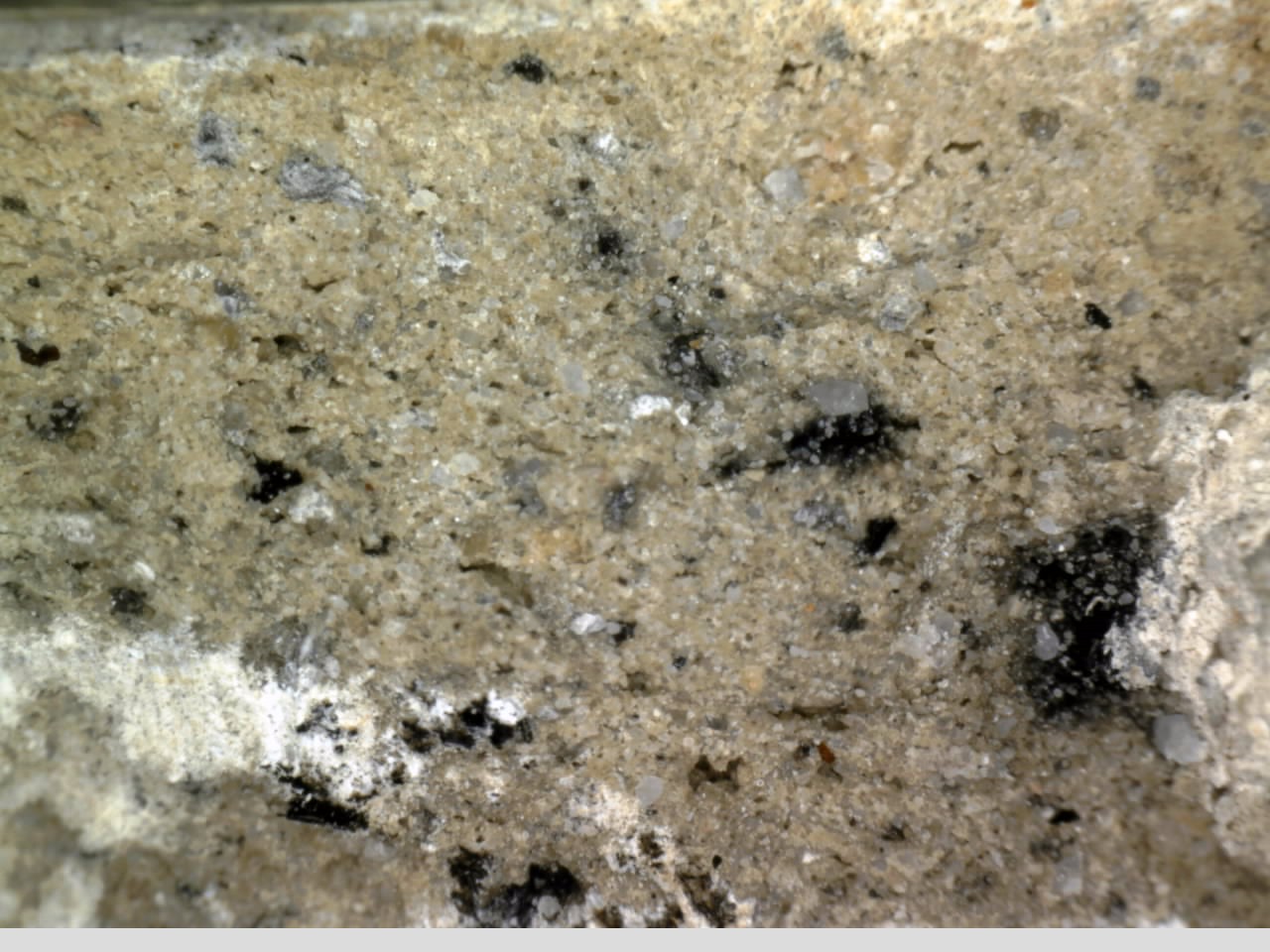

Supplement: Supplementary file 3 — Supplementary material [file mmc3.zip › Appendix A/HTN 50/HTN 50-50m-4.jpg]

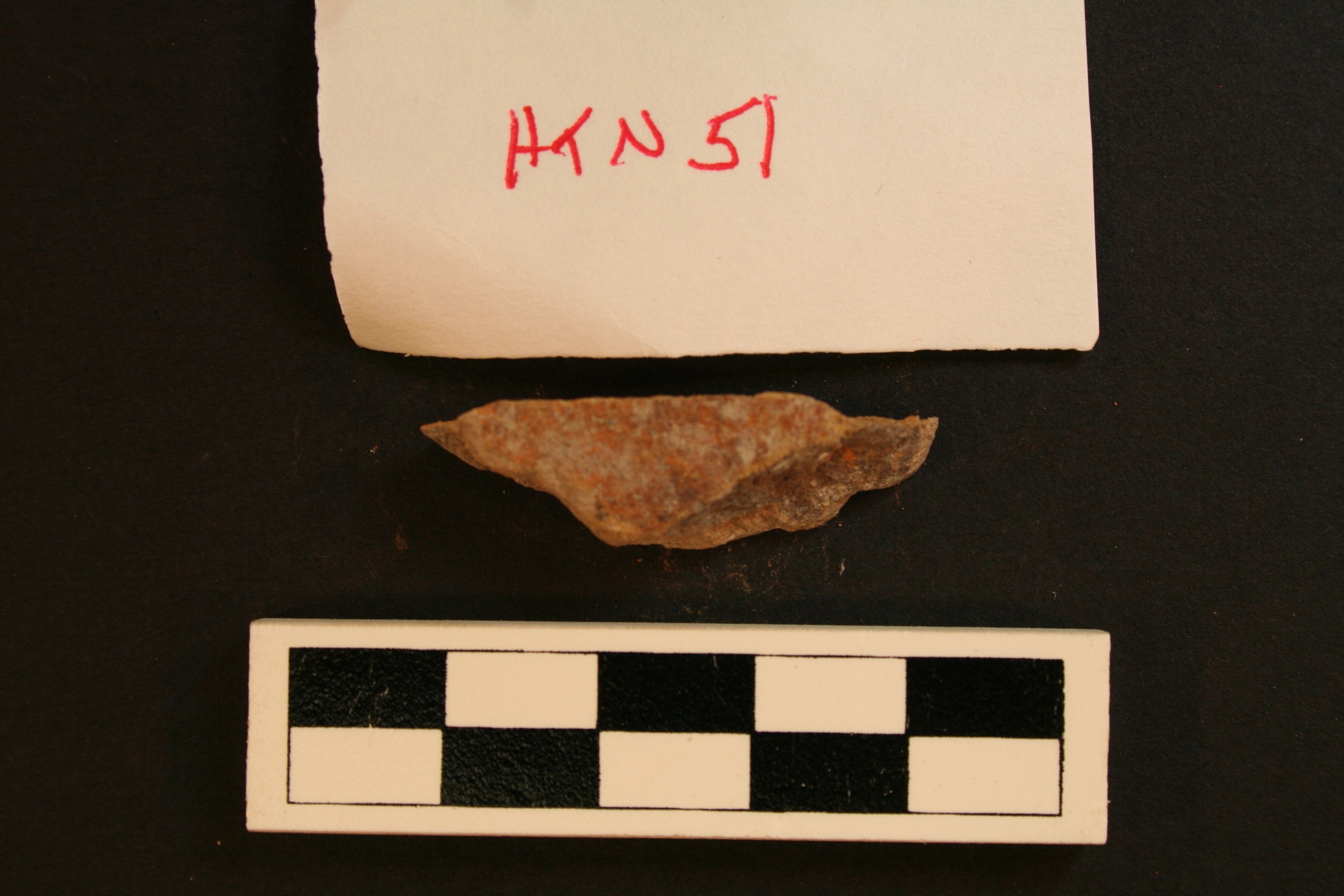

Supplement: Supplementary file 3 — Supplementary material [file mmc3.zip › Appendix A/HTN 51/51a.JPG]

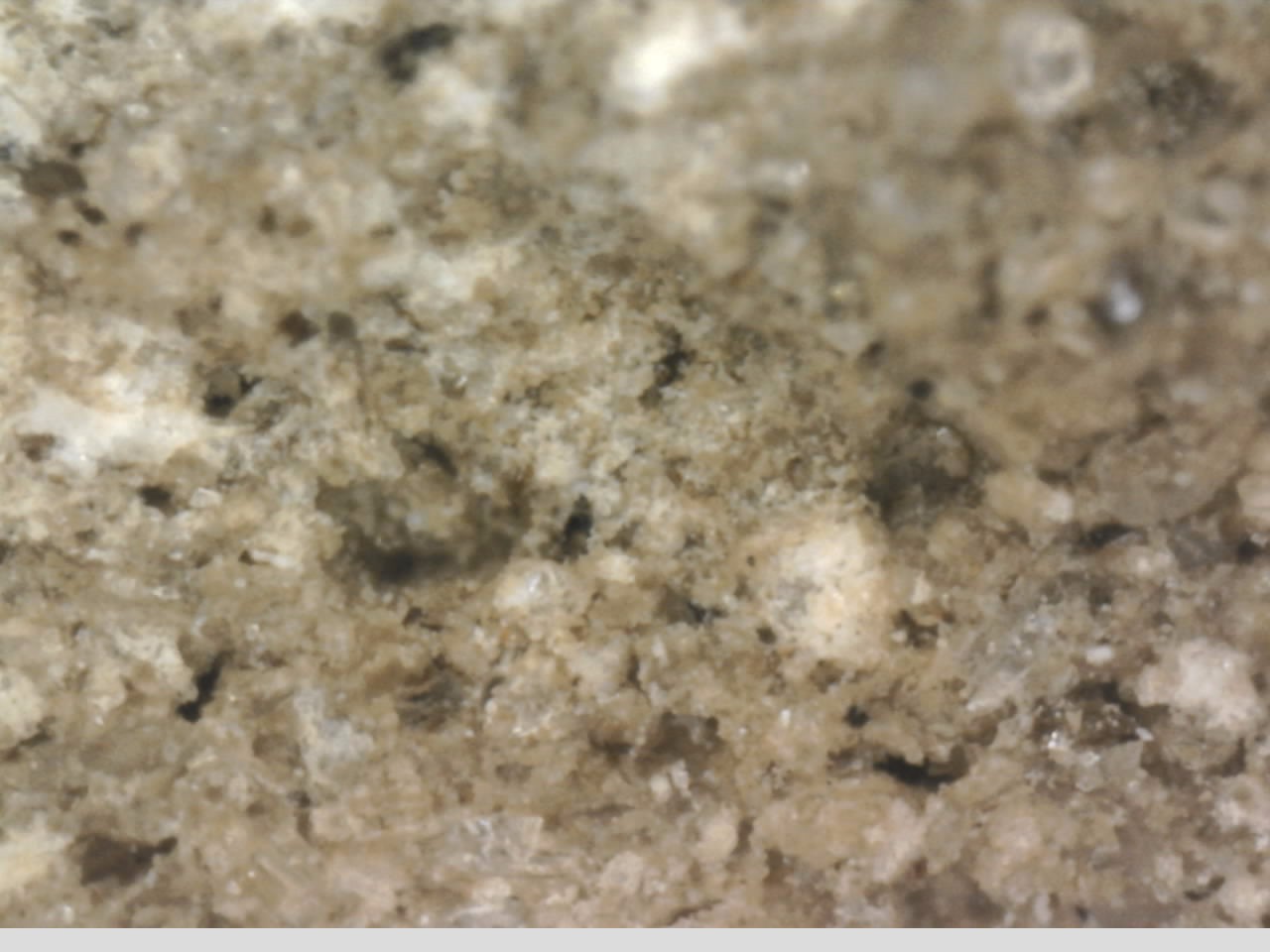

Supplement: Supplementary file 3 — Supplementary material [file mmc3.zip › Appendix A/HTN 51/HTN 51-250m-2.jpg]

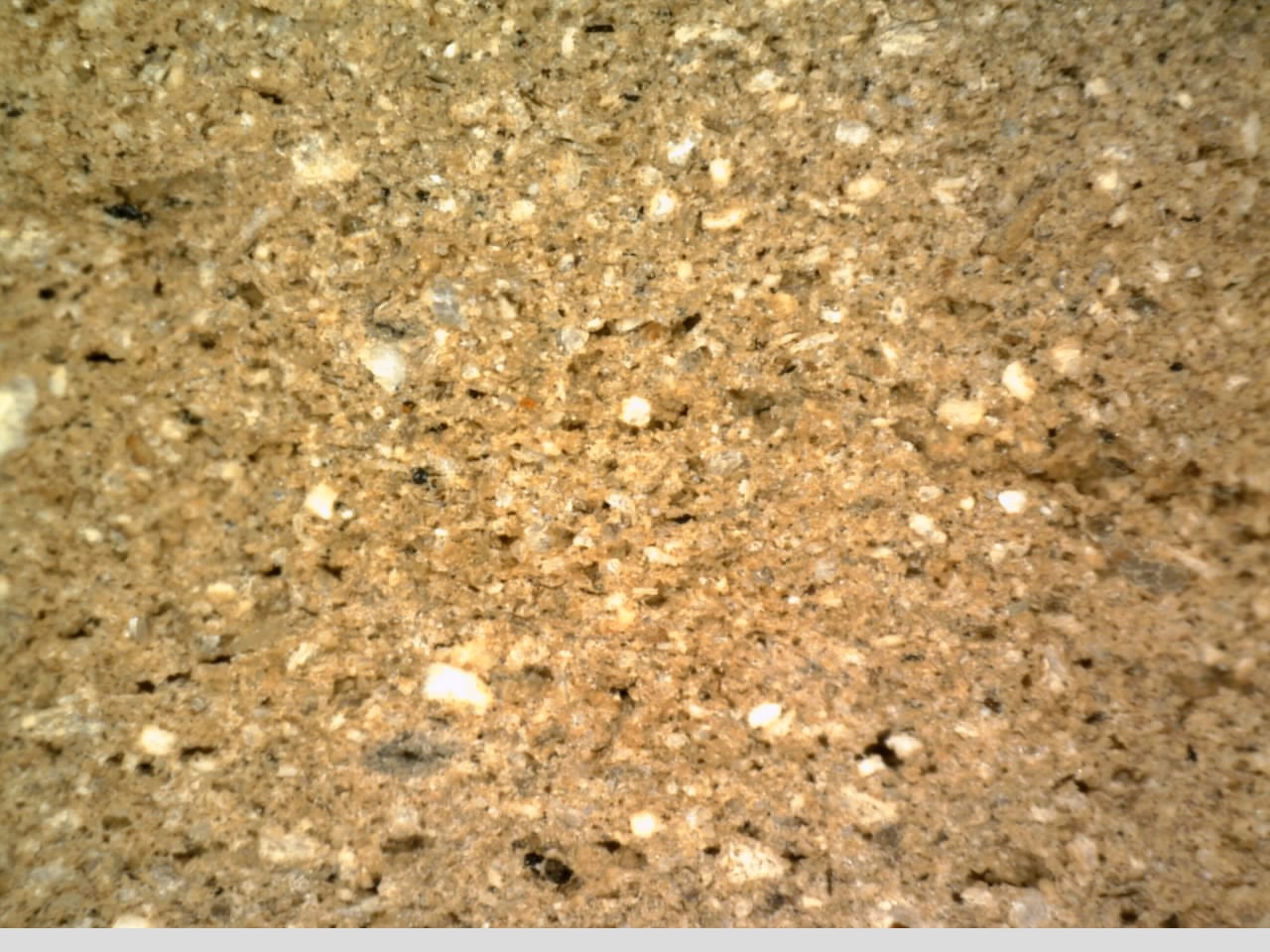

Supplement: Supplementary file 3 — Supplementary material [file mmc3.zip › Appendix A/HTN 51/HTN 51-50m-1.jpg]

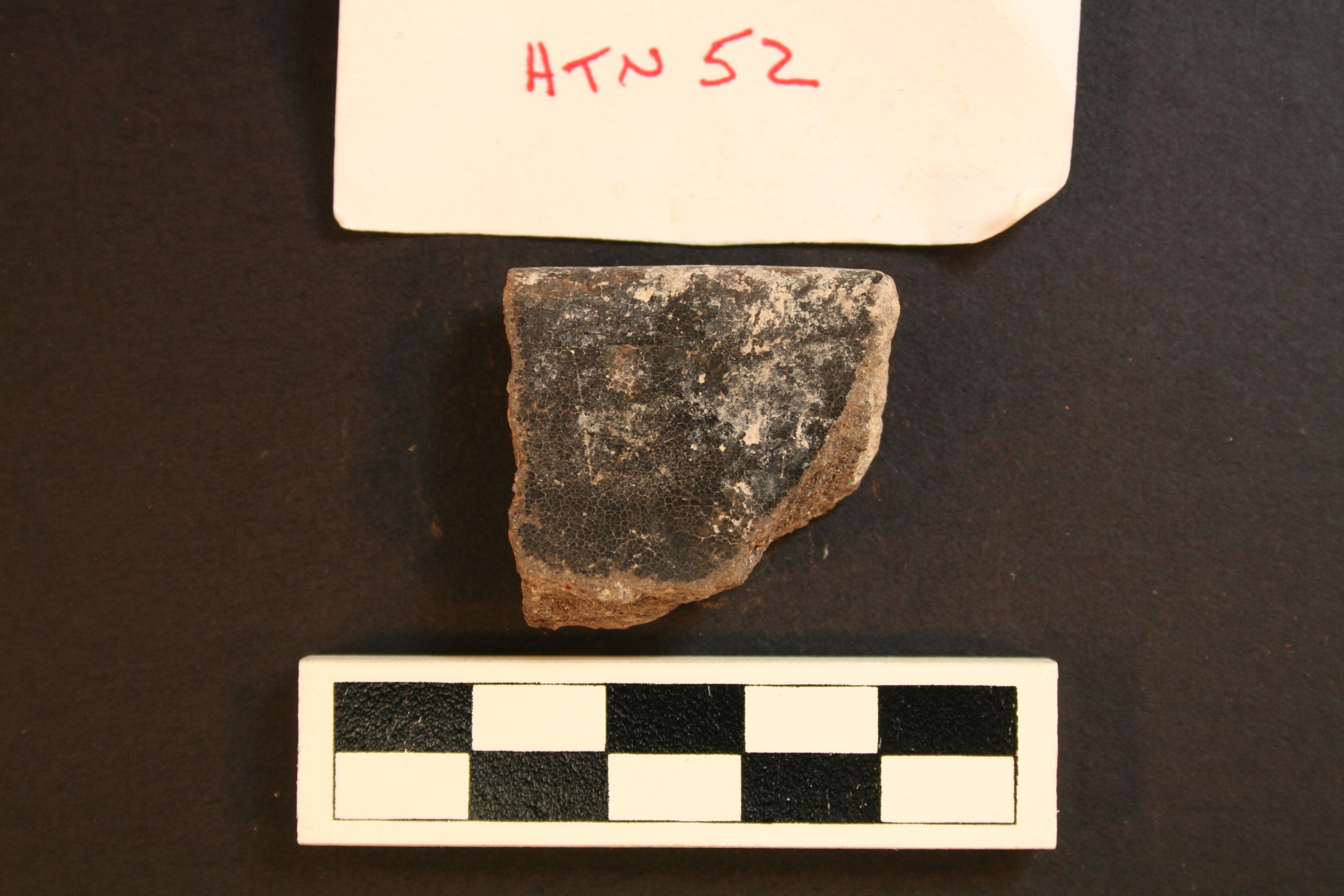

Supplement: Supplementary file 3 — Supplementary material [file mmc3.zip › Appendix A/HTN 52/52b.JPG]

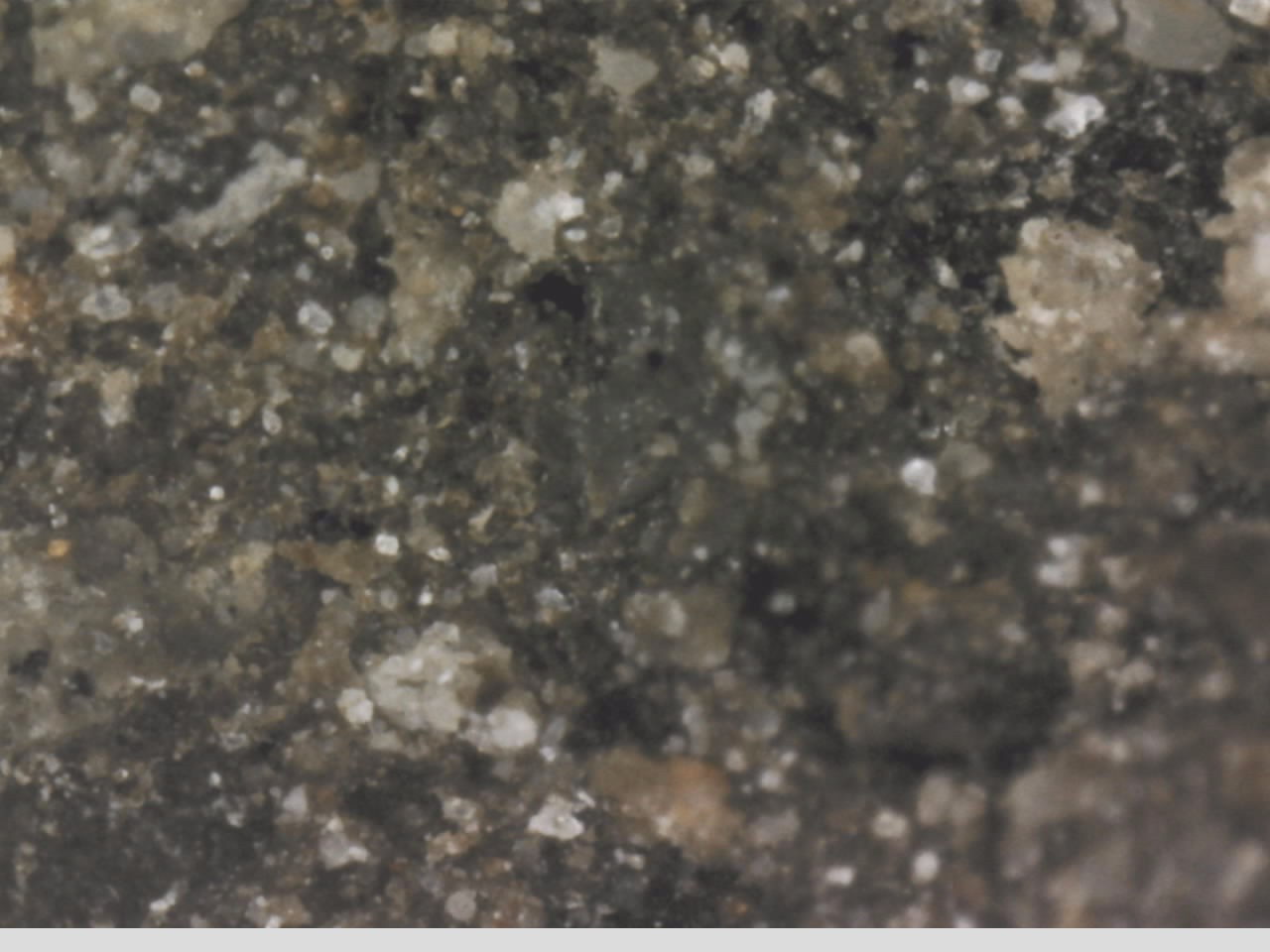

Supplement: Supplementary file 3 — Supplementary material [file mmc3.zip › Appendix A/HTN 52/HTN 52-250m-3.jpg]

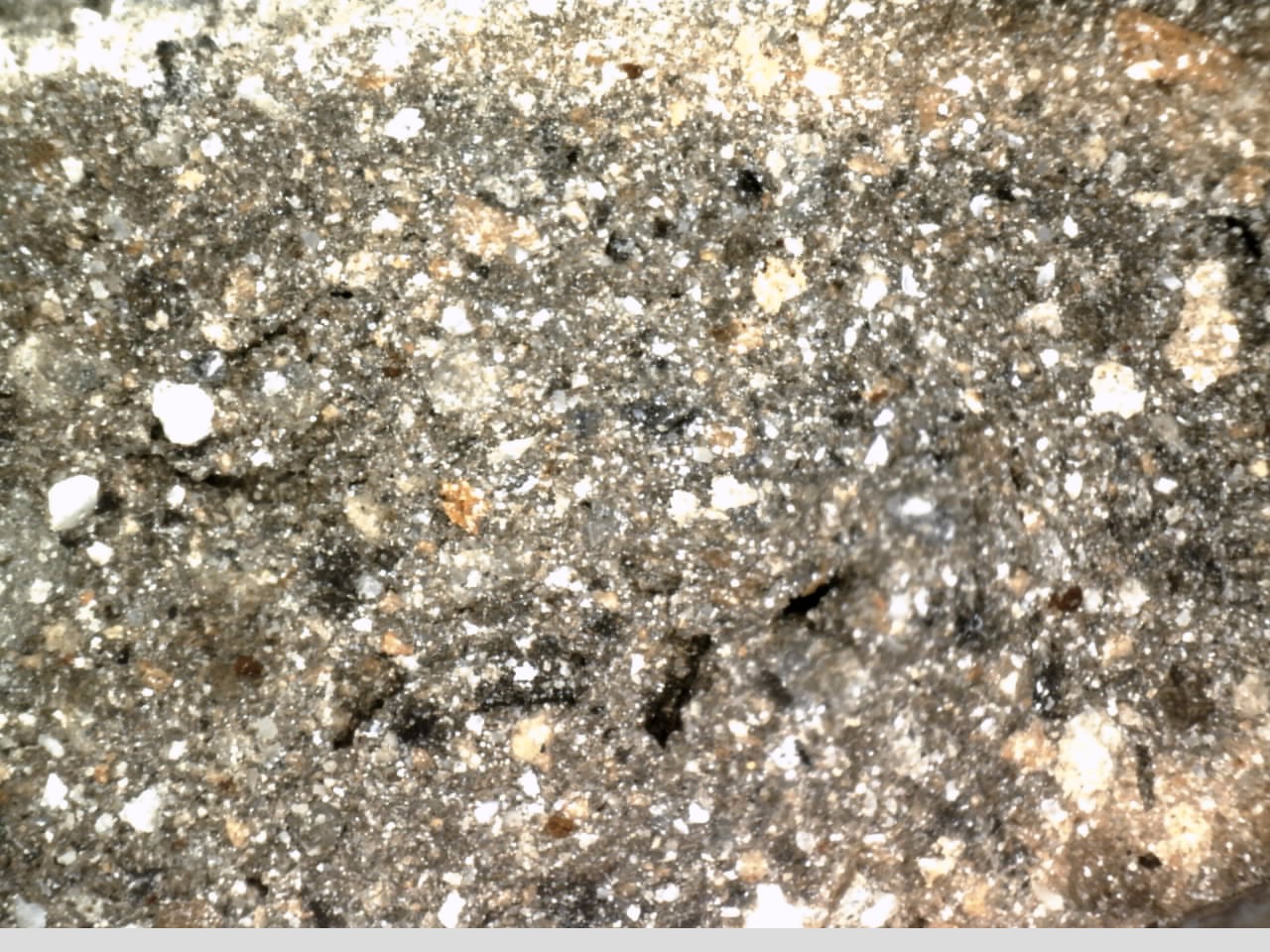

Supplement: Supplementary file 3 — Supplementary material [file mmc3.zip › Appendix A/HTN 52/HTN 52-50m-1.jpg]

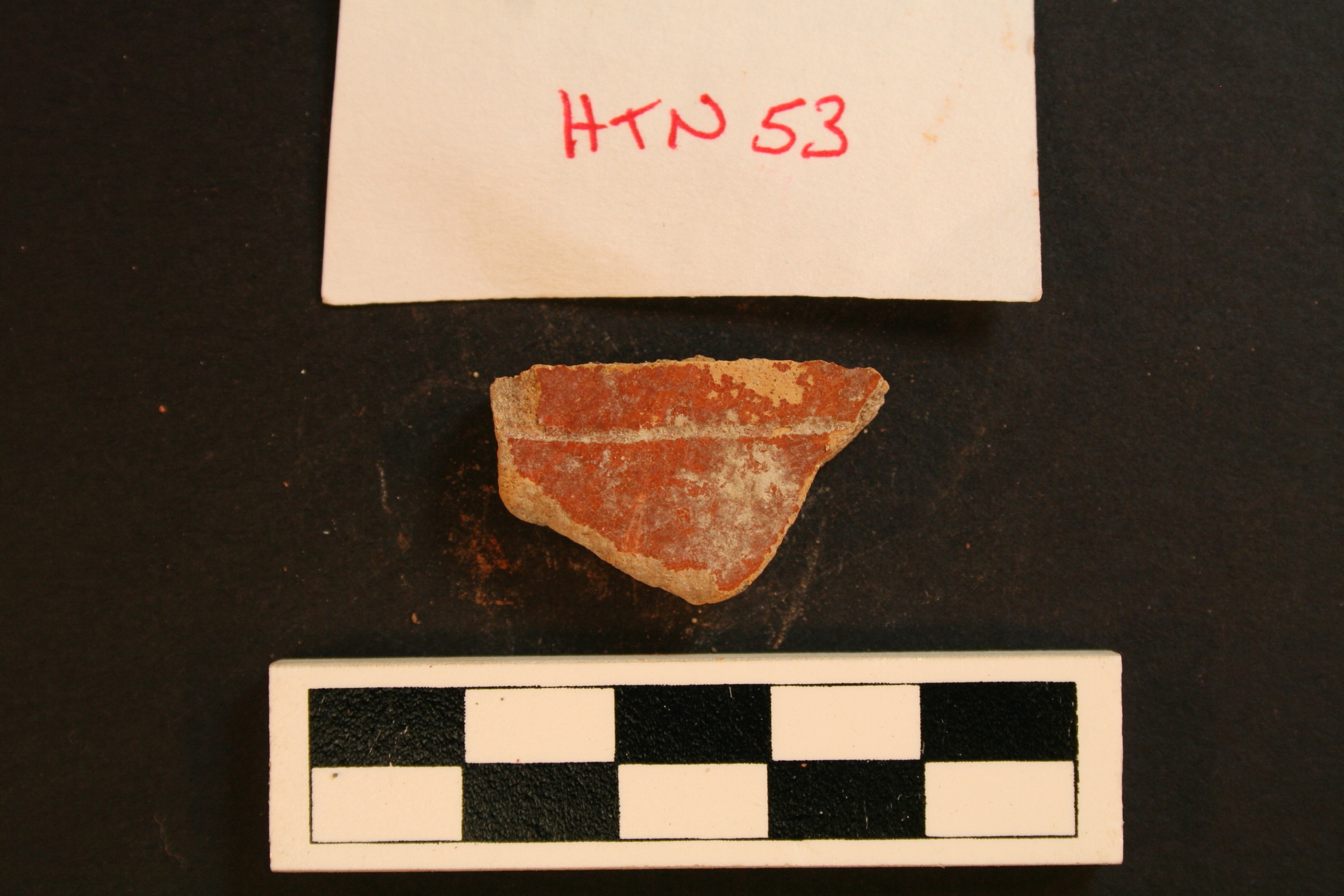

Supplement: Supplementary file 3 — Supplementary material [file mmc3.zip › Appendix A/HTN 53/53a.JPG]

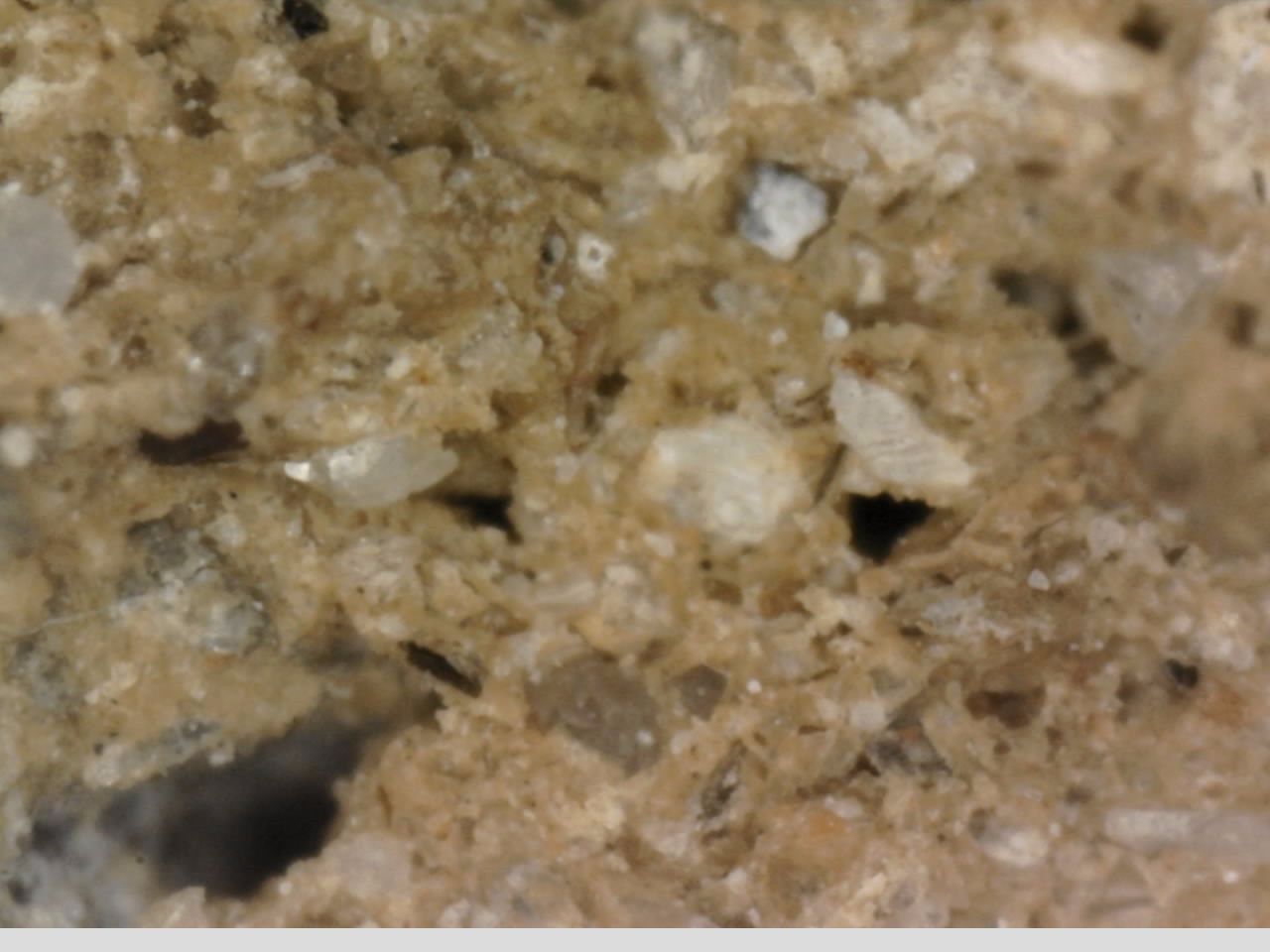

Supplement: Supplementary file 3 — Supplementary material [file mmc3.zip › Appendix A/HTN 53/HTN 53-250m-0.jpg]

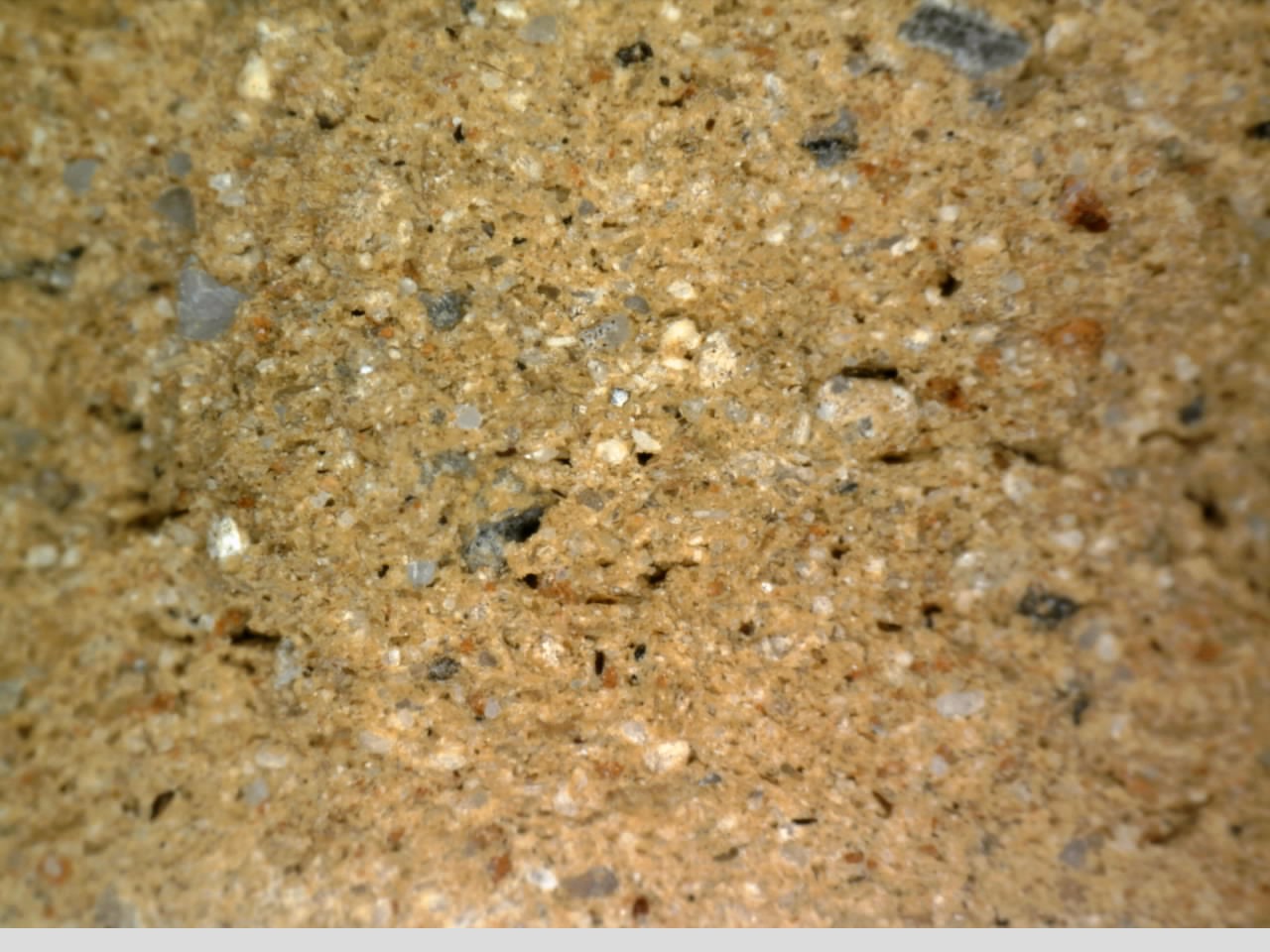

Supplement: Supplementary file 3 — Supplementary material [file mmc3.zip › Appendix A/HTN 53/HTN 53-50m-1.jpg]

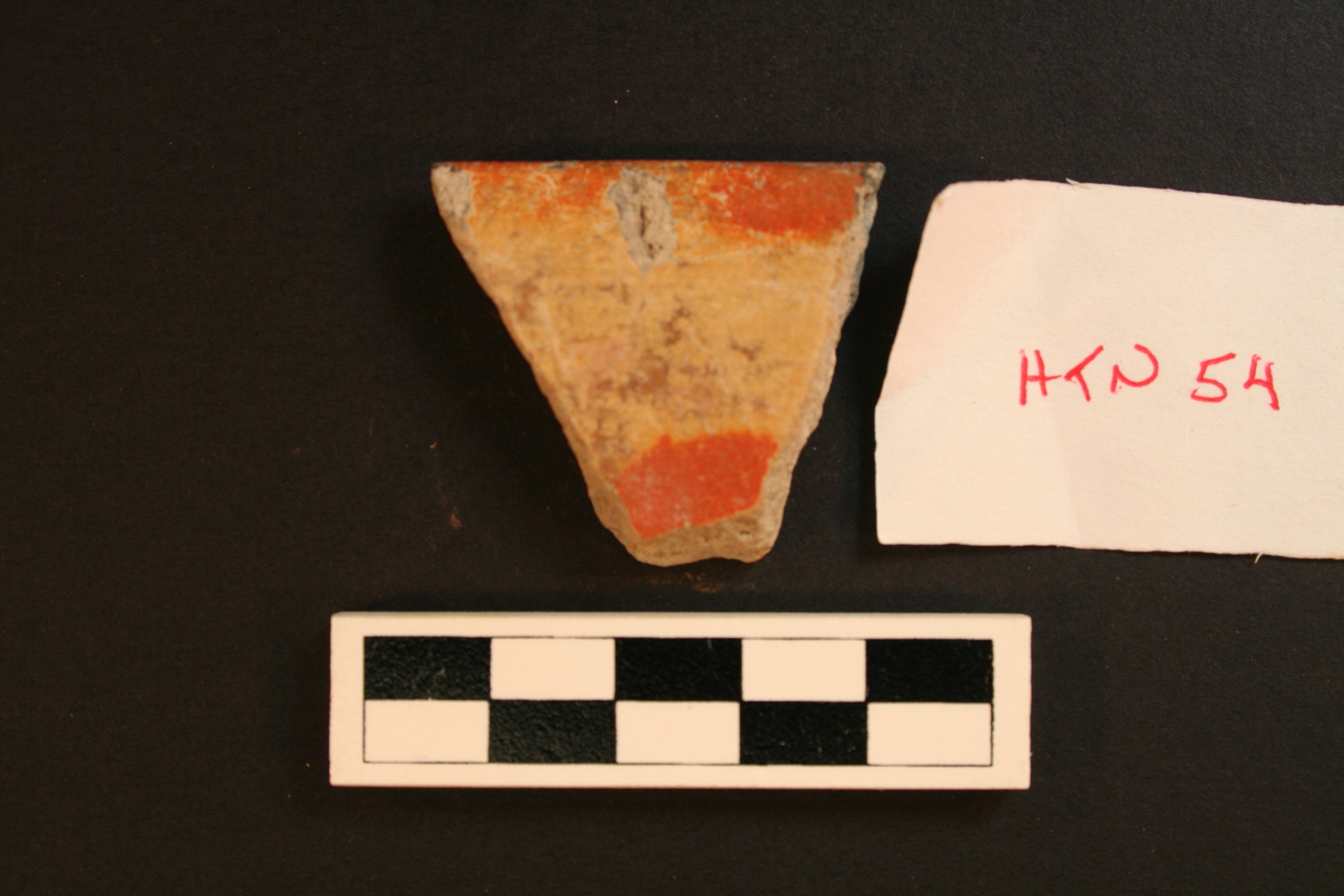

Supplement: Supplementary file 3 — Supplementary material [file mmc3.zip › Appendix A/HTN 54/54b.JPG]

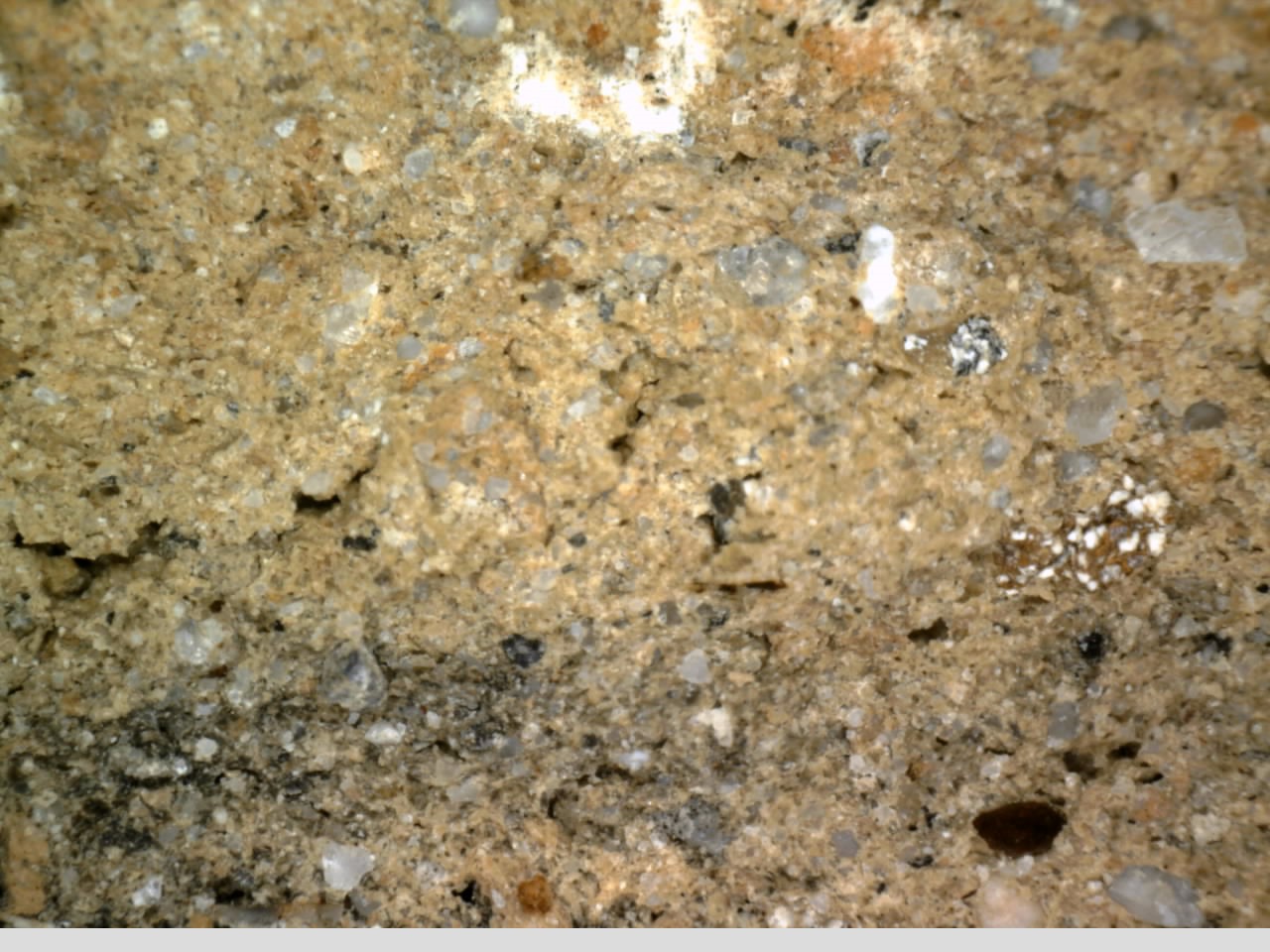

Supplement: Supplementary file 3 — Supplementary material [file mmc3.zip › Appendix A/HTN 54/HTN 54-50m-1.jpg]

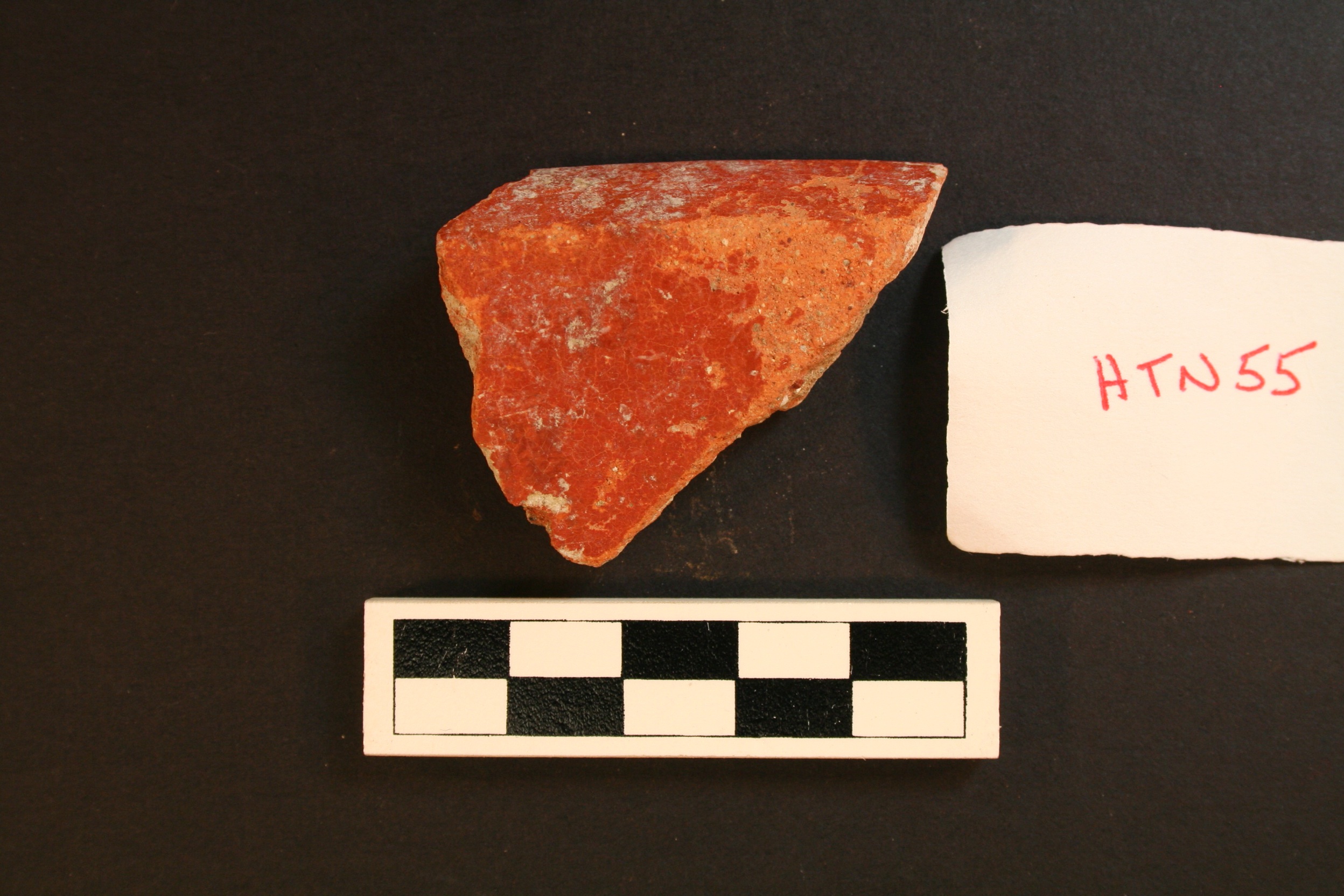

Supplement: Supplementary file 3 — Supplementary material [file mmc3.zip › Appendix A/HTN 55/55a.JPG]

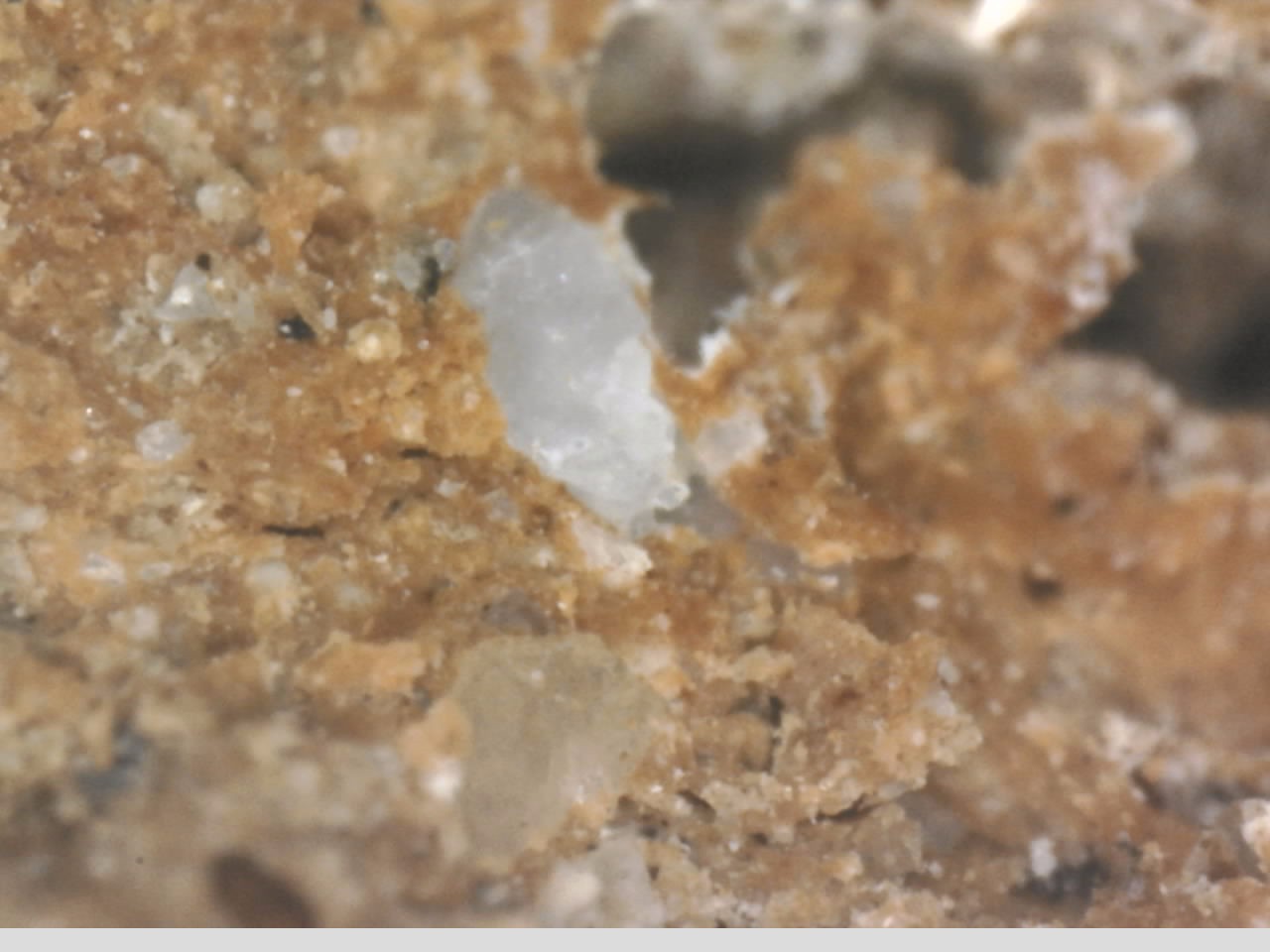

Supplement: Supplementary file 3 — Supplementary material [file mmc3.zip › Appendix A/HTN 55/HTN 55-250m-5.jpg]

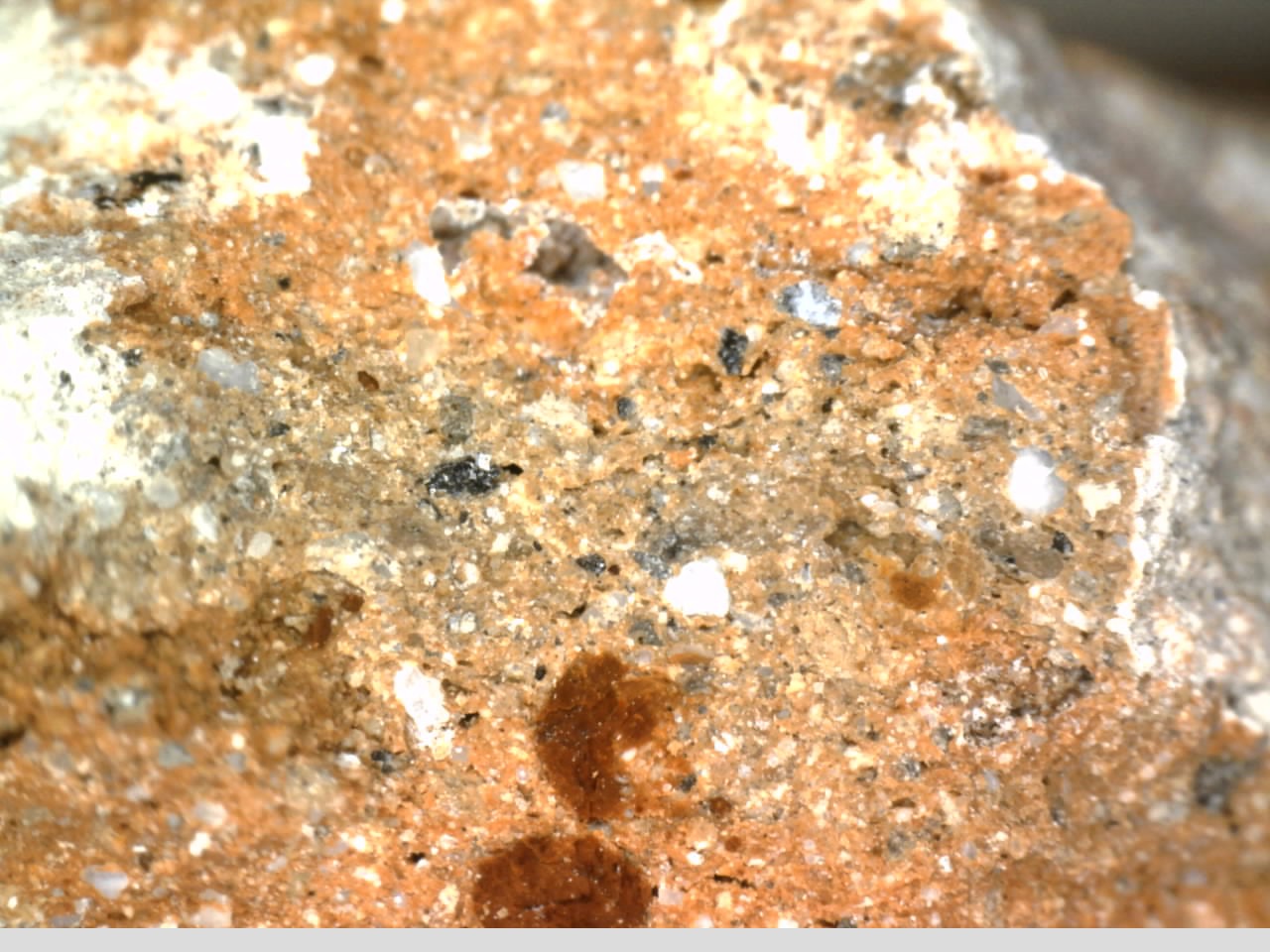

Supplement: Supplementary file 3 — Supplementary material [file mmc3.zip › Appendix A/HTN 55/HTN 55-50m-6.jpg]

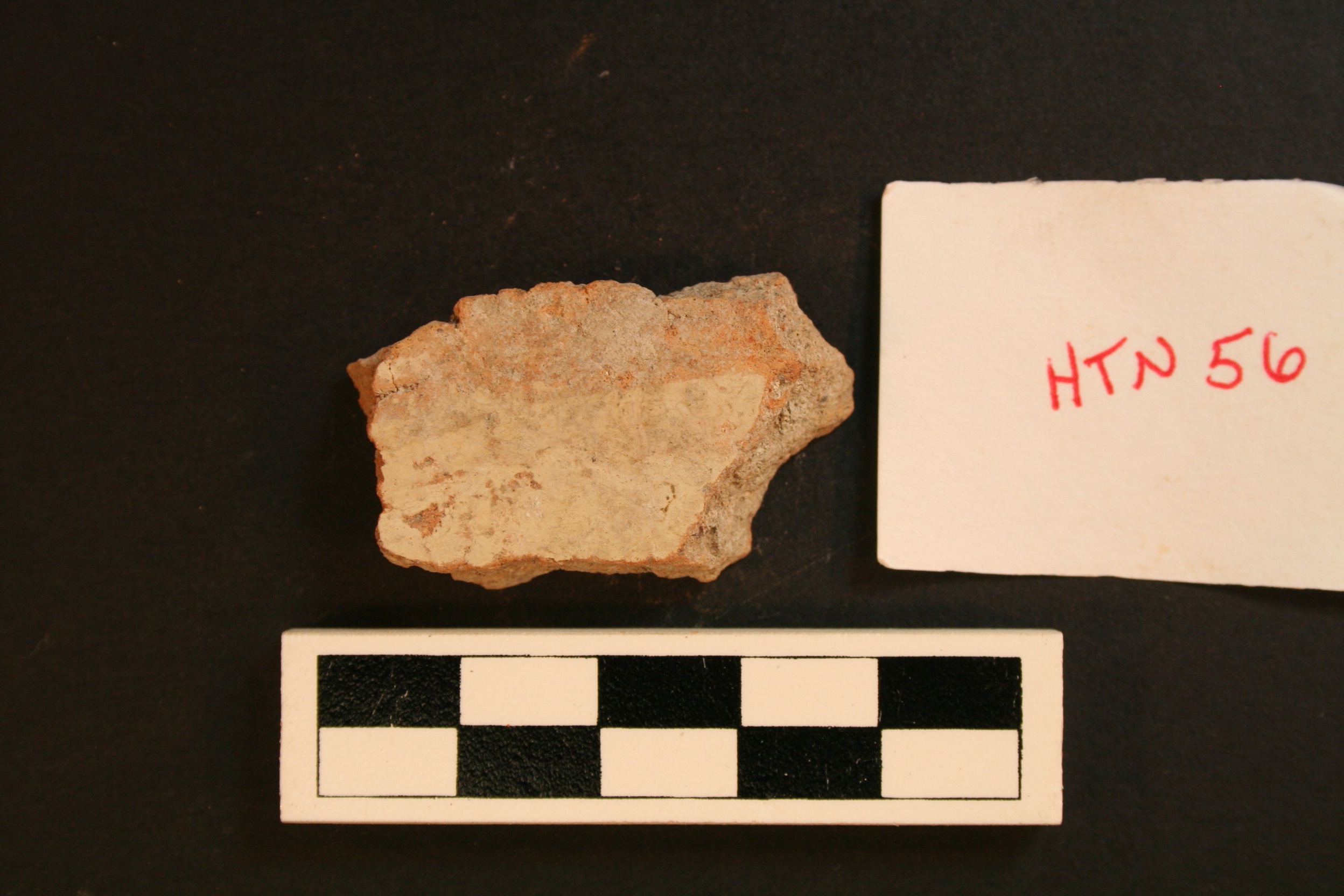

Supplement: Supplementary file 3 — Supplementary material [file mmc3.zip › Appendix A/HTN 56/56a.JPG]

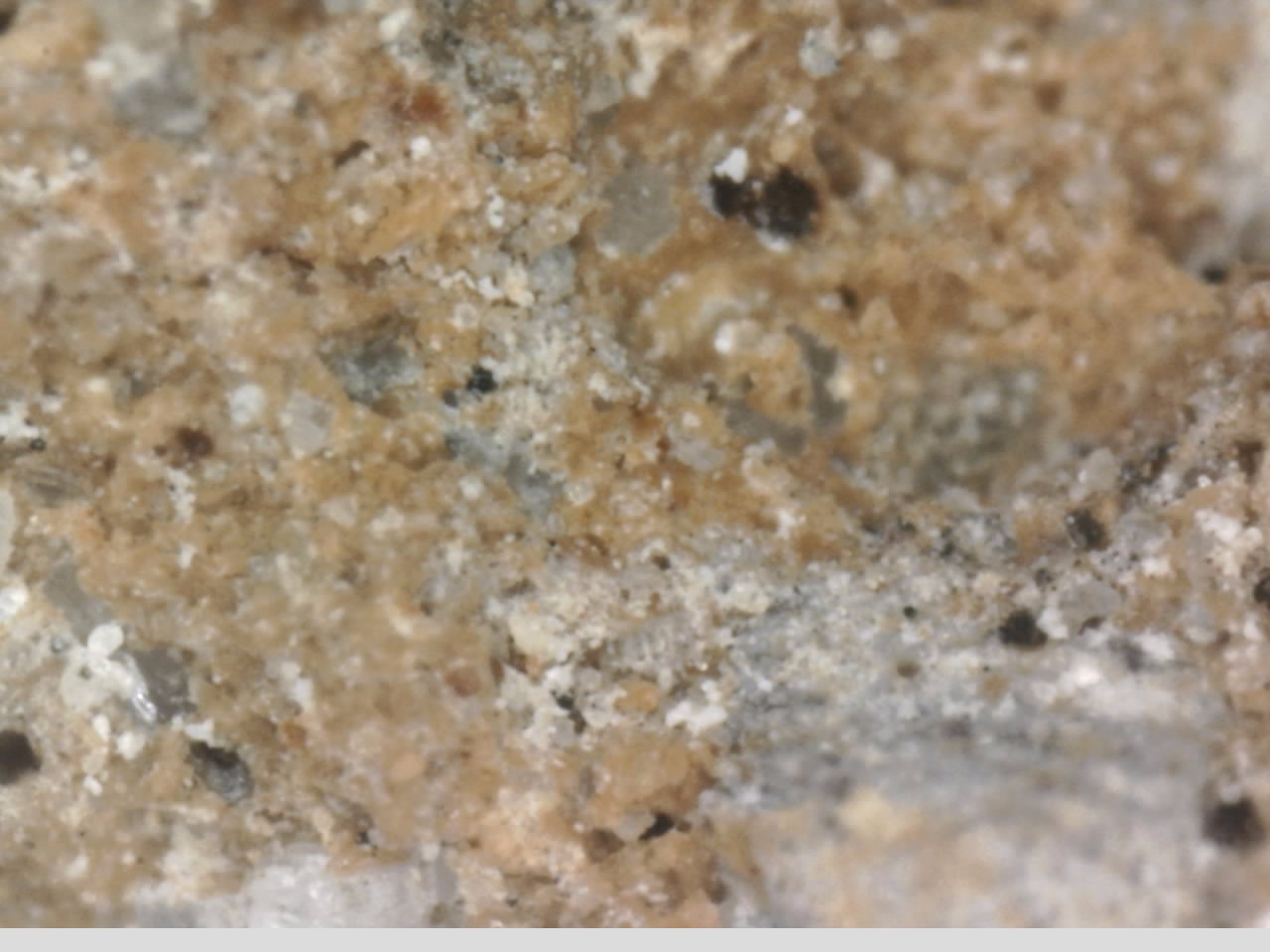

Supplement: Supplementary file 3 — Supplementary material [file mmc3.zip › Appendix A/HTN 56/HTN 56-250m-9.jpg]

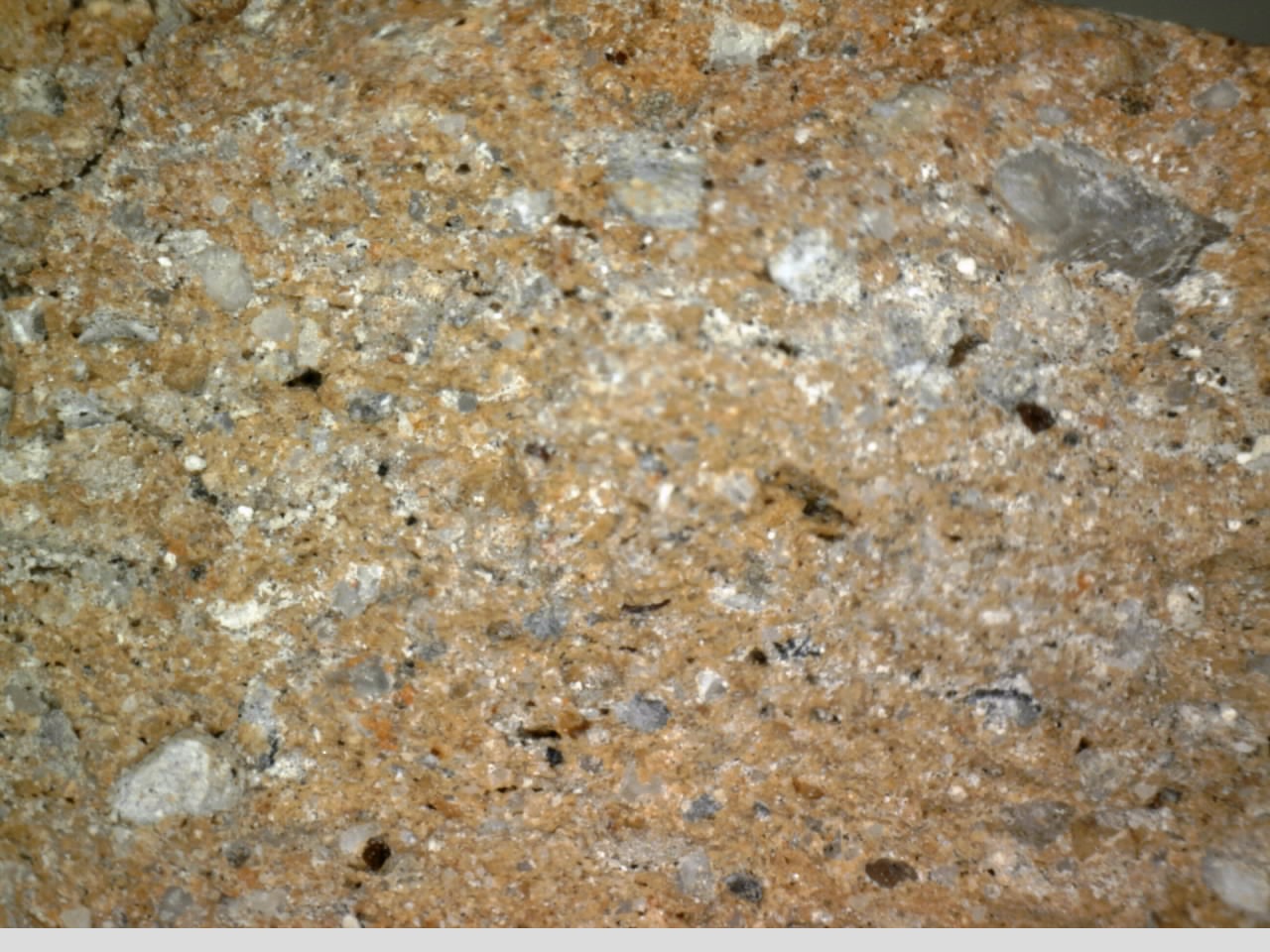

Supplement: Supplementary file 3 — Supplementary material [file mmc3.zip › Appendix A/HTN 56/HTN 56-50m-0.jpg]

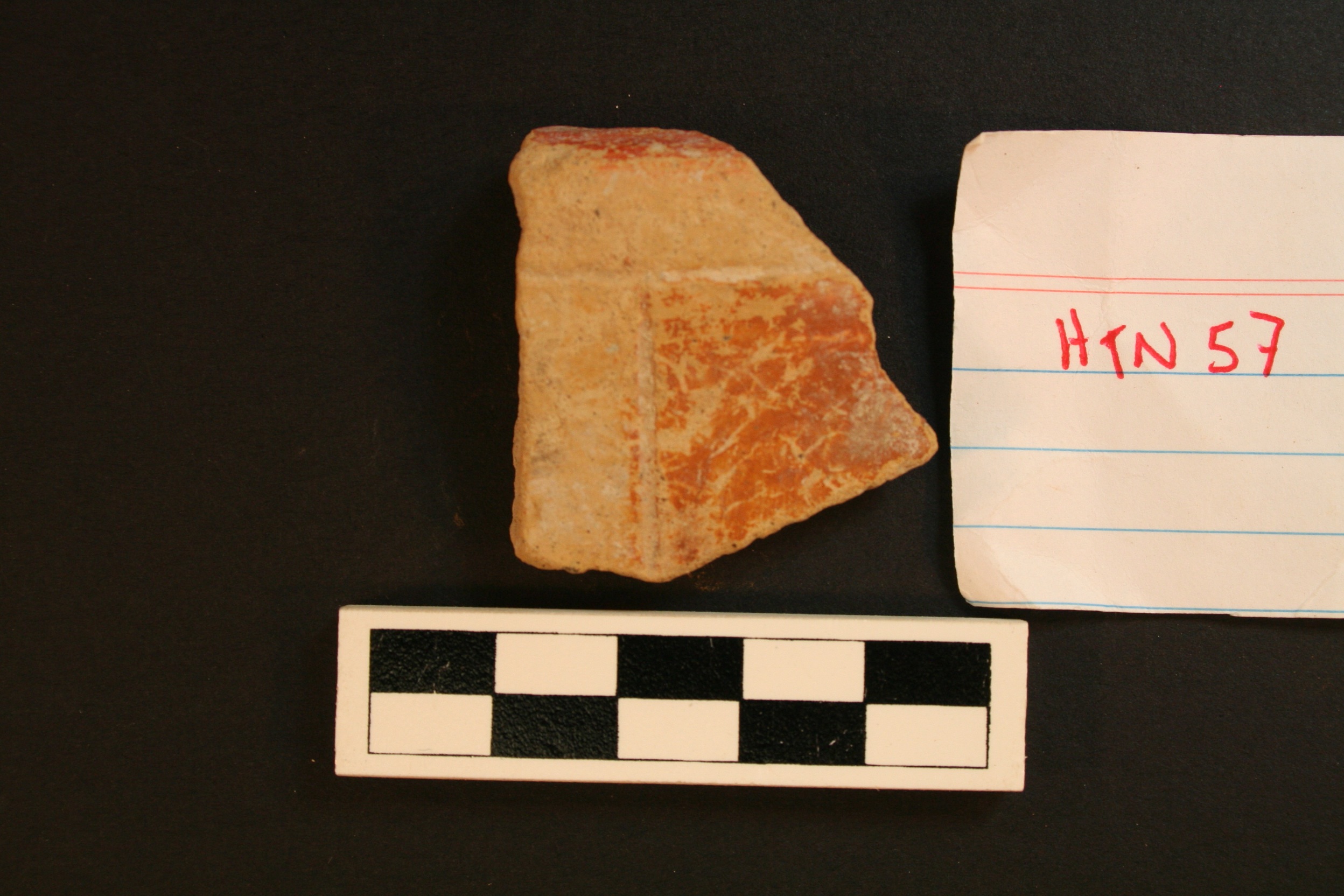

Supplement: Supplementary file 3 — Supplementary material [file mmc3.zip › Appendix A/HTN 57/57a.JPG]

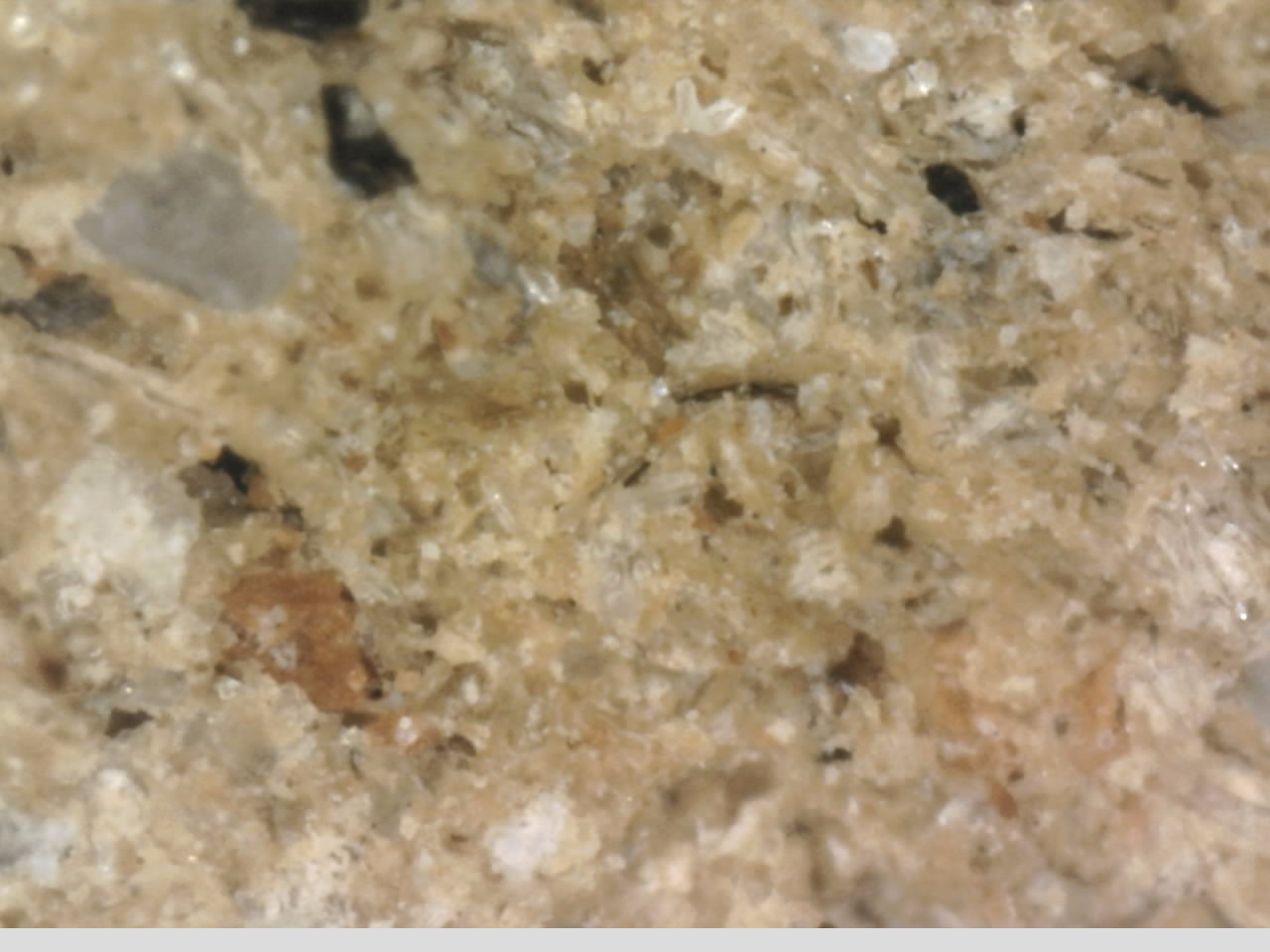

Supplement: Supplementary file 3 — Supplementary material [file mmc3.zip › Appendix A/HTN 57/HTN 57-250m-3.jpg]

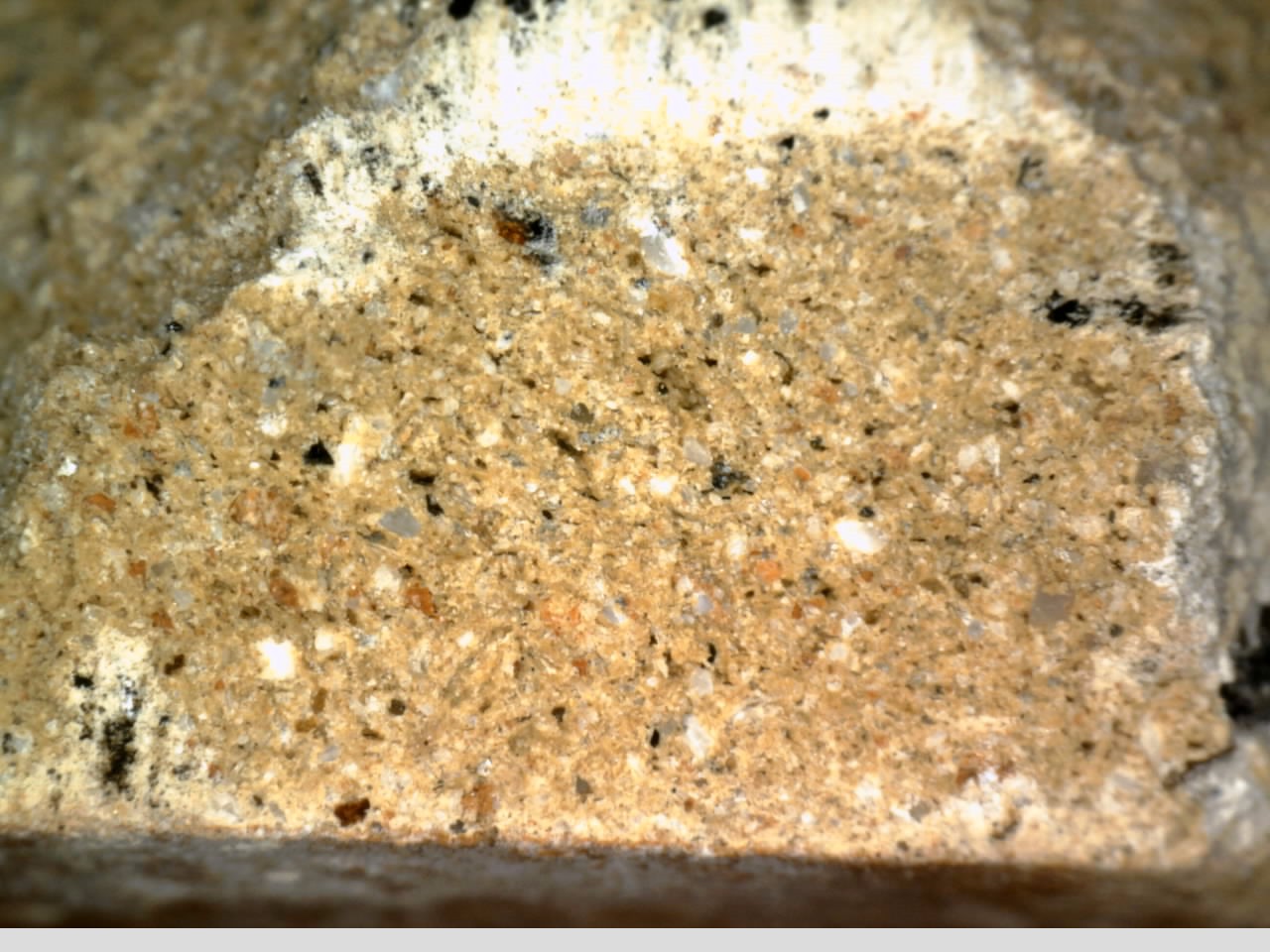

Supplement: Supplementary file 3 — Supplementary material [file mmc3.zip › Appendix A/HTN 57/HTN 57-50m-7.jpg]

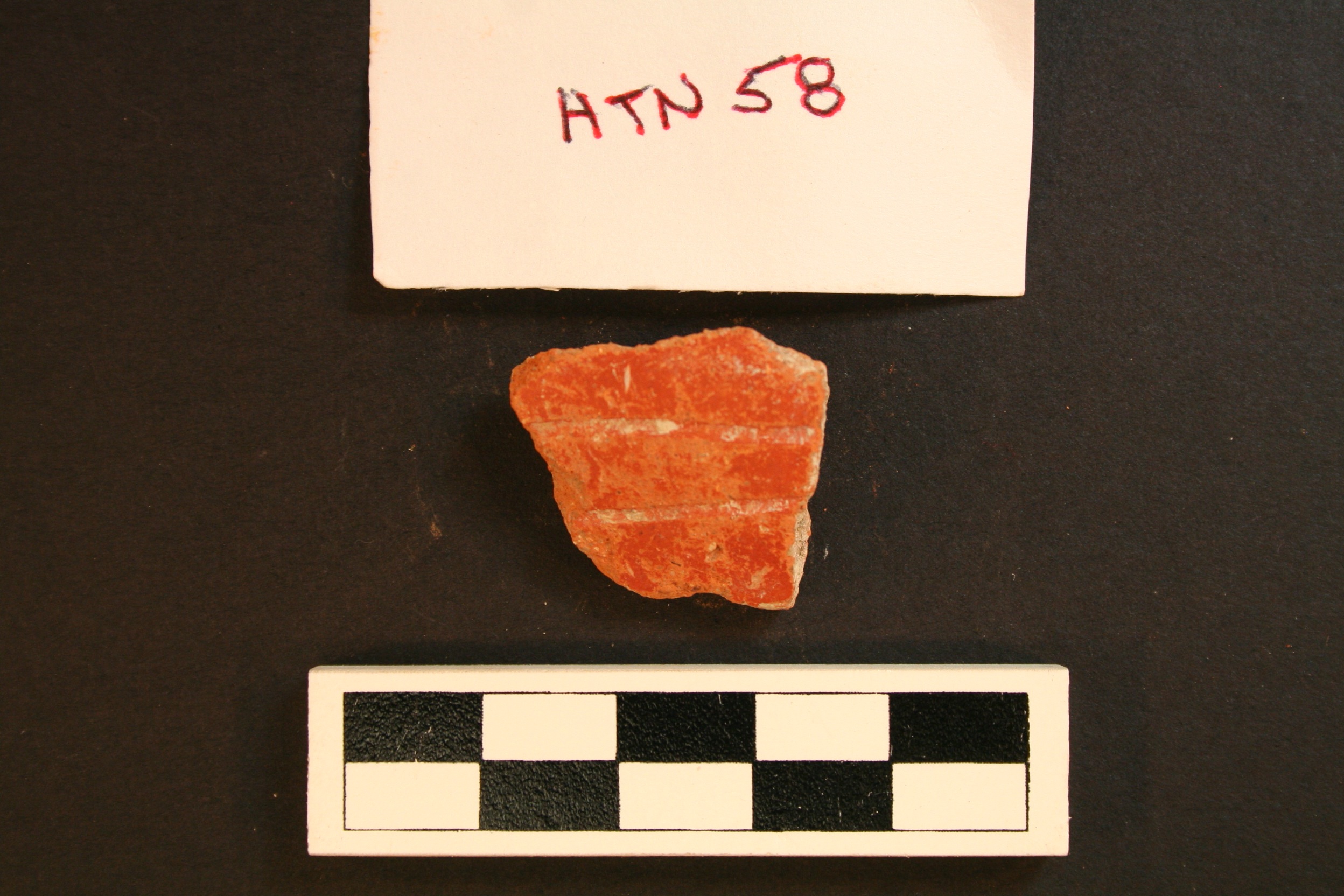

Supplement: Supplementary file 3 — Supplementary material [file mmc3.zip › Appendix A/HTN 58/58a.JPG]

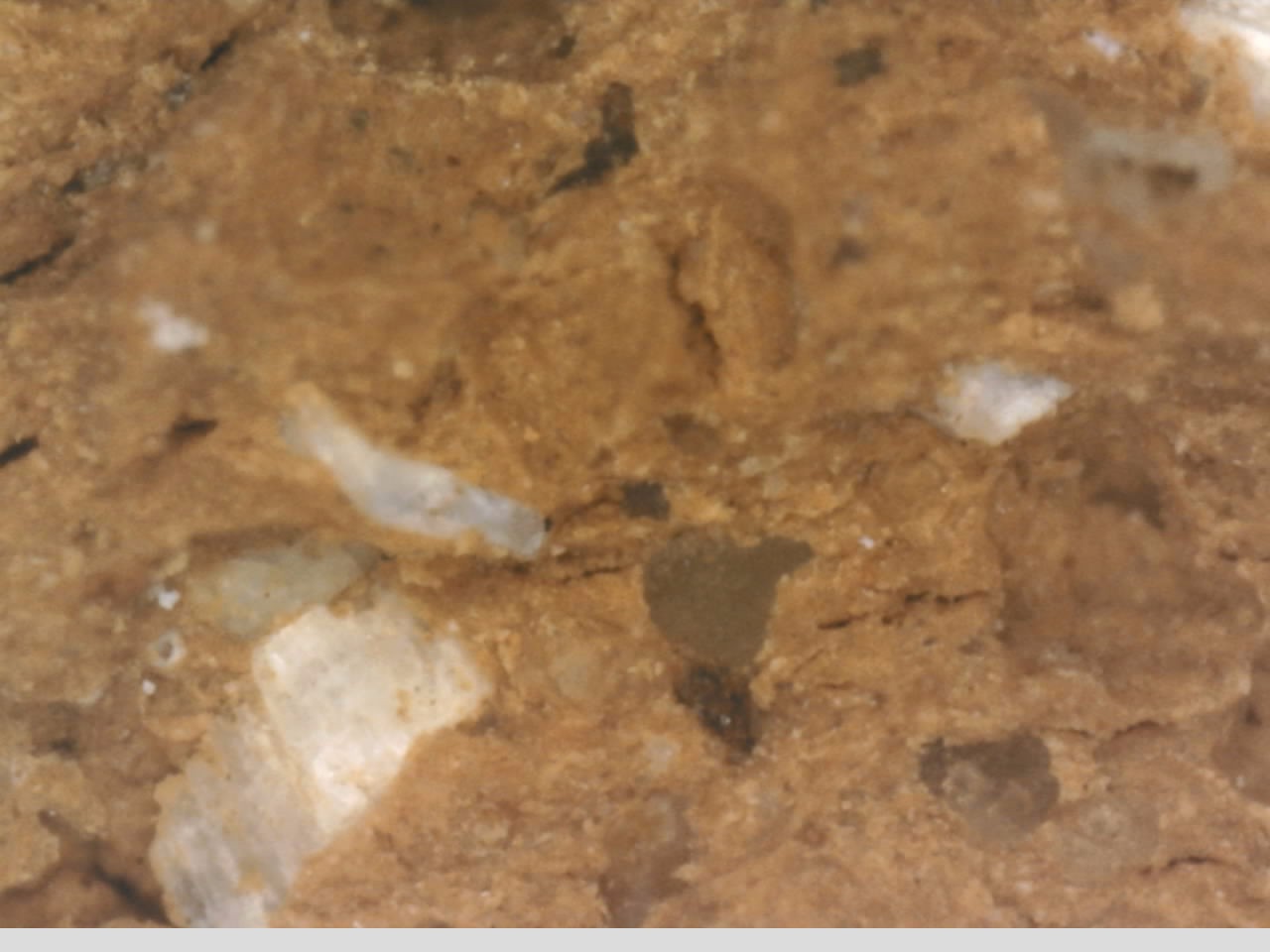

Supplement: Supplementary file 3 — Supplementary material [file mmc3.zip › Appendix A/HTN 58/HTN 58-250m-4.jpg]

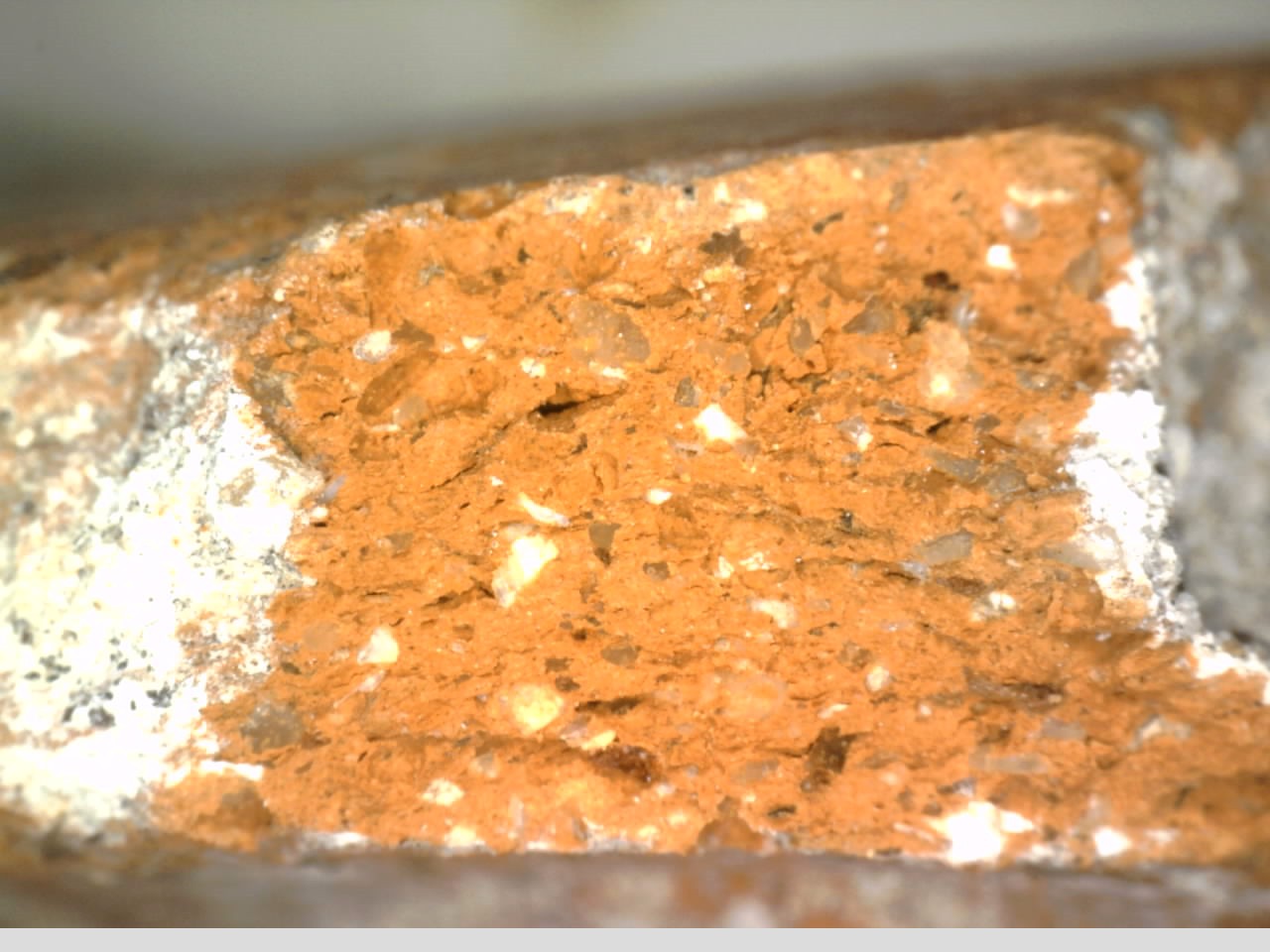

Supplement: Supplementary file 3 — Supplementary material [file mmc3.zip › Appendix A/HTN 58/HTN 58-50m-7.jpg]

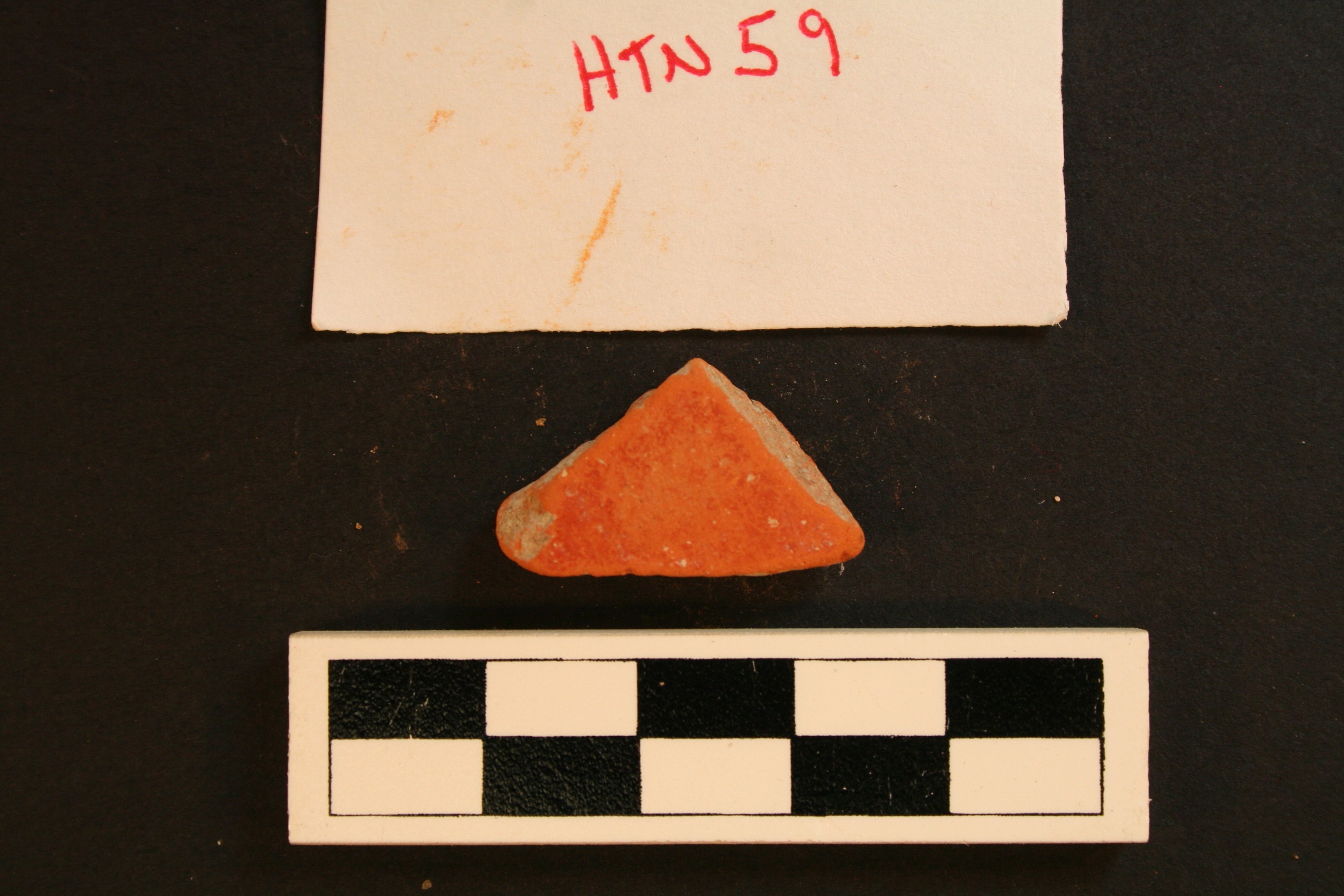

Supplement: Supplementary file 3 — Supplementary material [file mmc3.zip › Appendix A/HTN 59/59a.JPG]

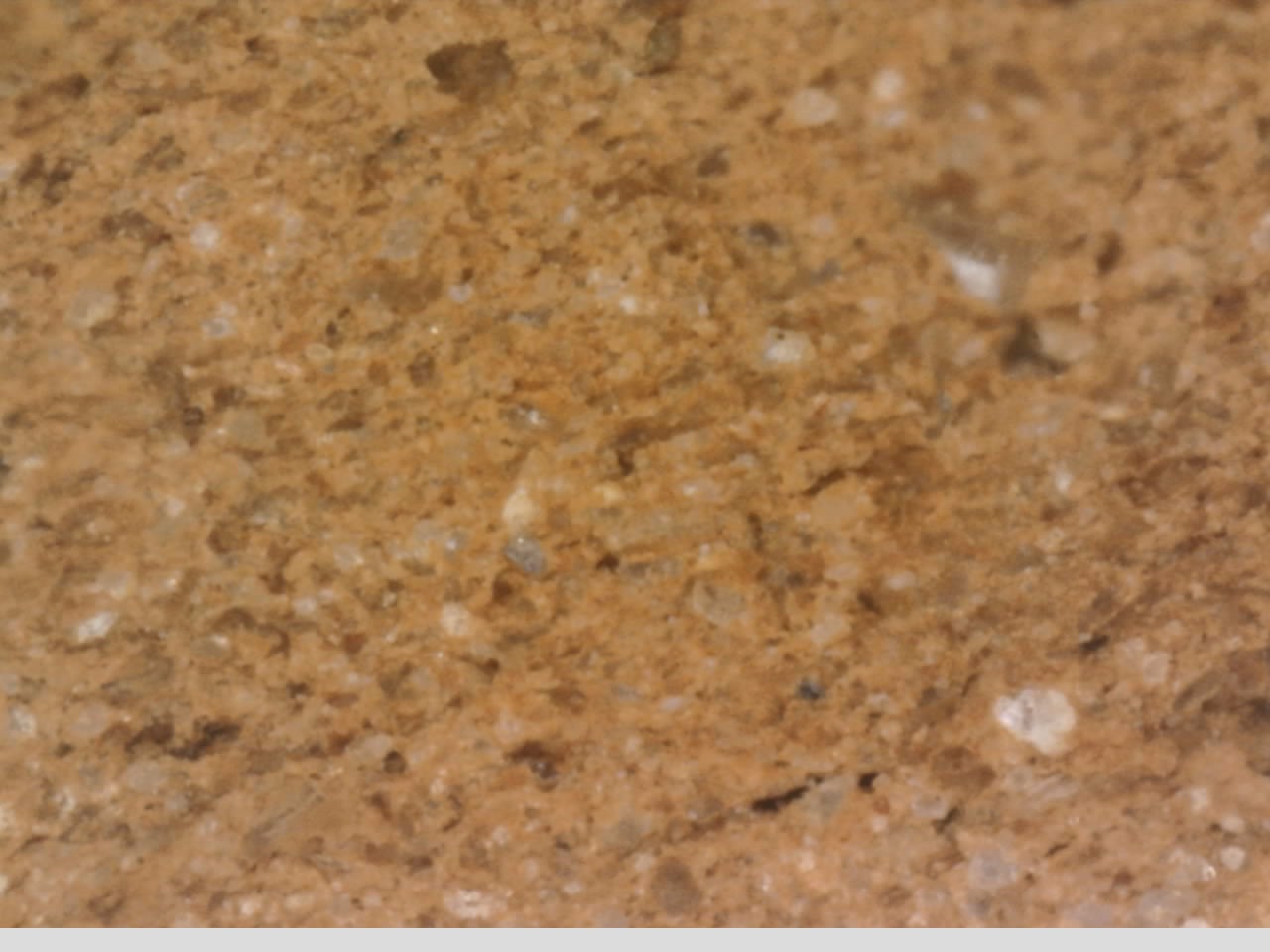

Supplement: Supplementary file 3 — Supplementary material [file mmc3.zip › Appendix A/HTN 59/HTN 59-250m-2.jpg]

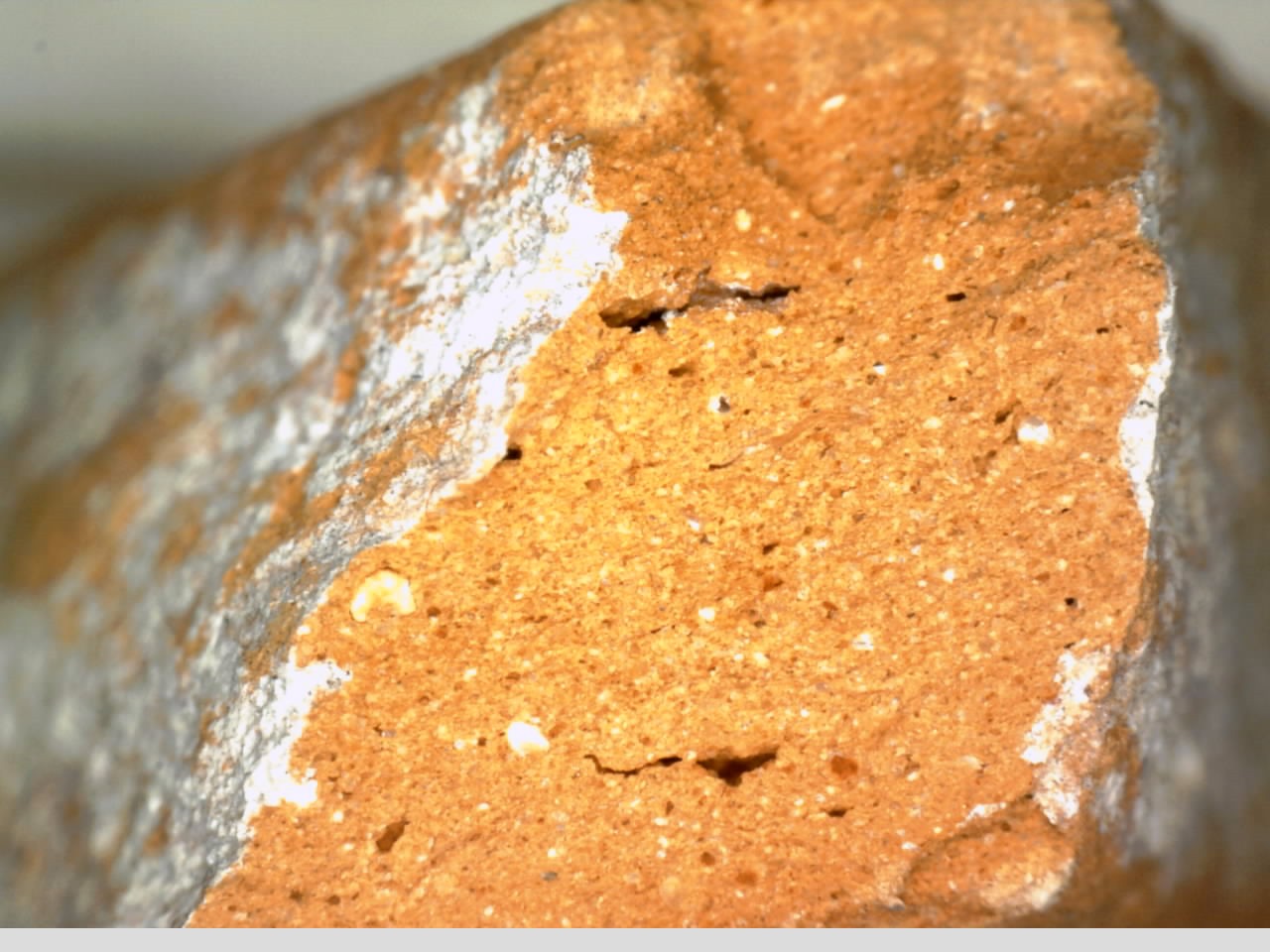

Supplement: Supplementary file 3 — Supplementary material [file mmc3.zip › Appendix A/HTN 59/HTN 59-50m-4.jpg]

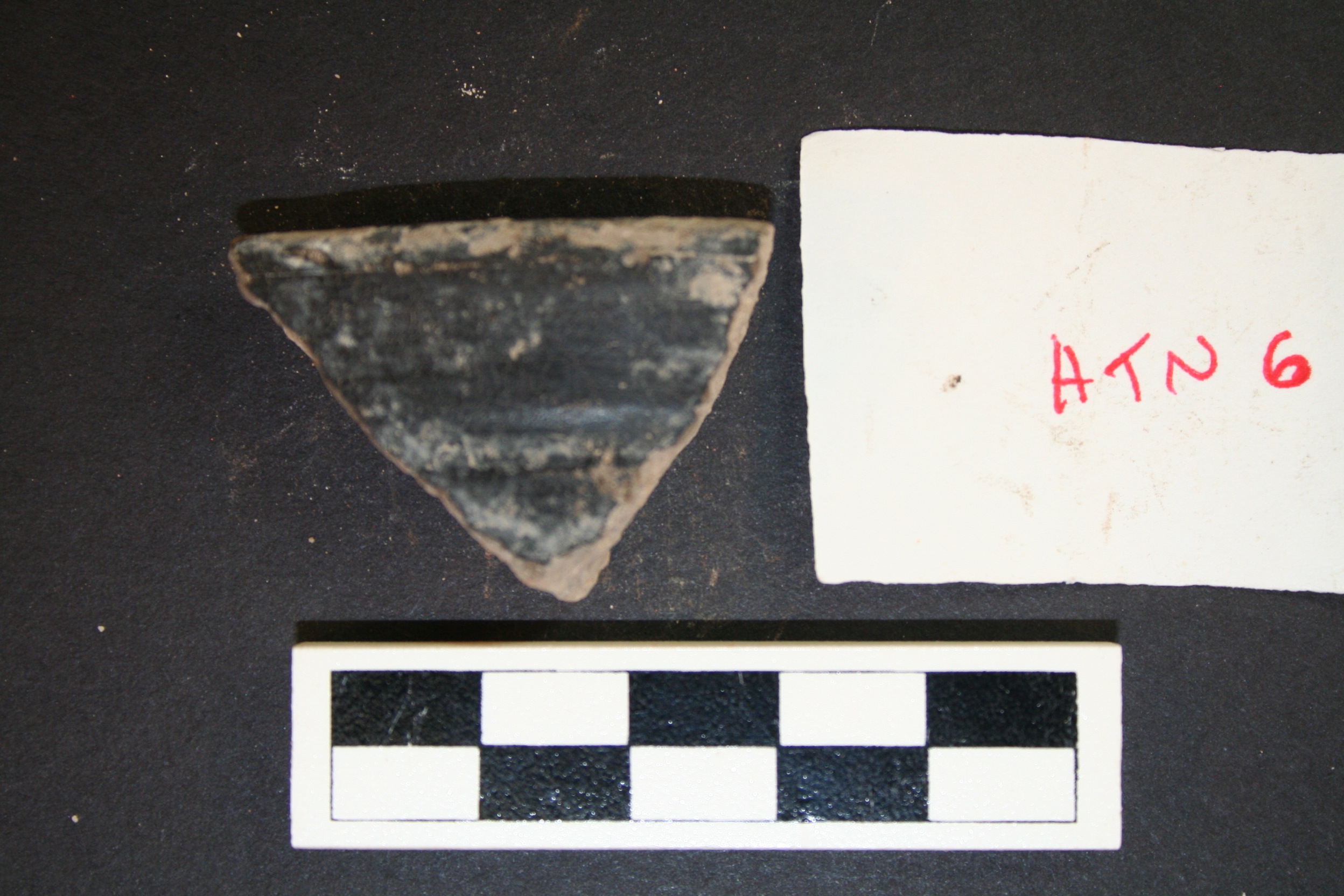

Supplement: Supplementary file 3 — Supplementary material [file mmc3.zip › Appendix A/HTN 6/6a.JPG]

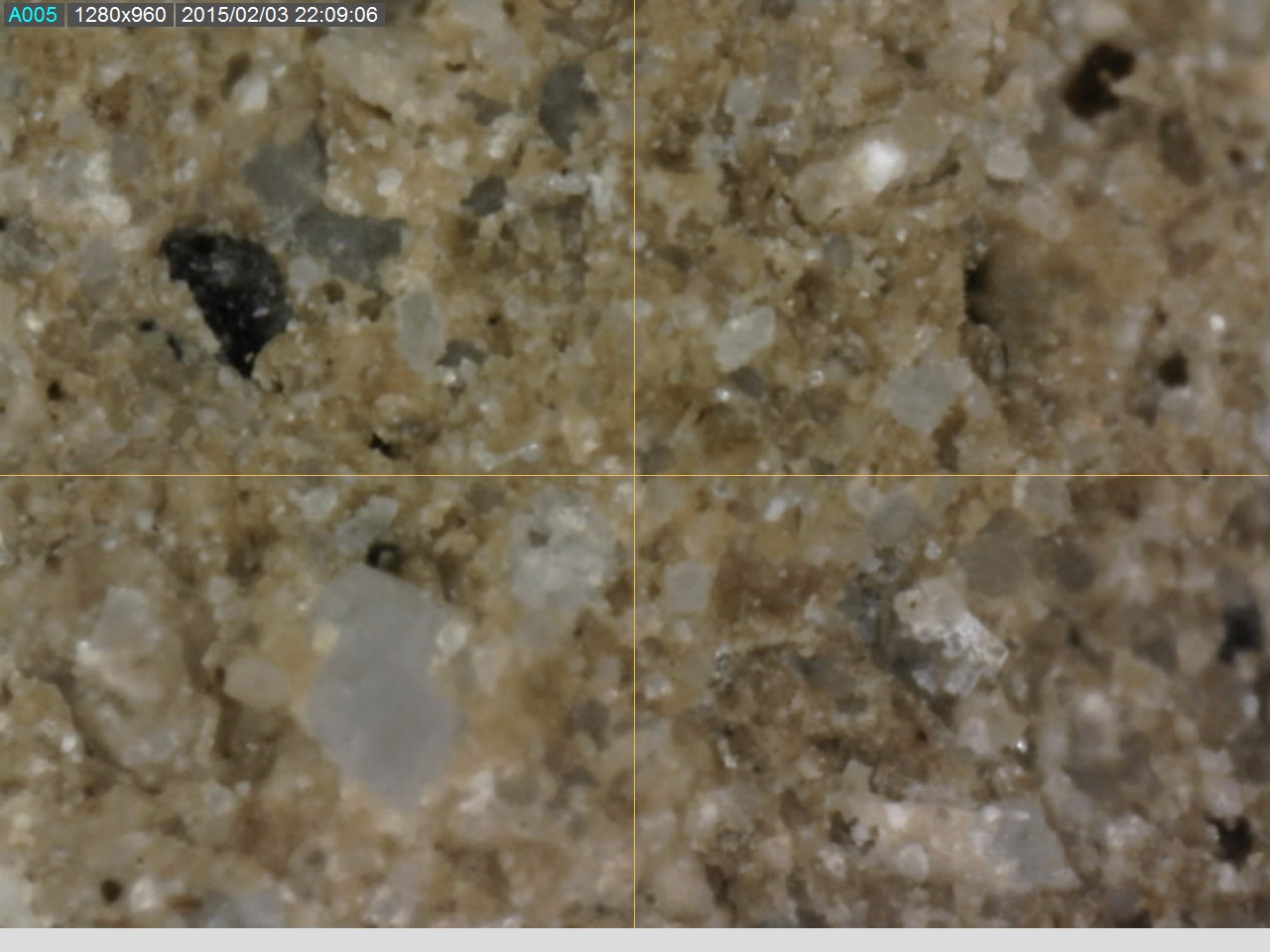

Supplement: Supplementary file 3 — Supplementary material [file mmc3.zip › Appendix A/HTN 6/HTN 6-250m-0.jpg]

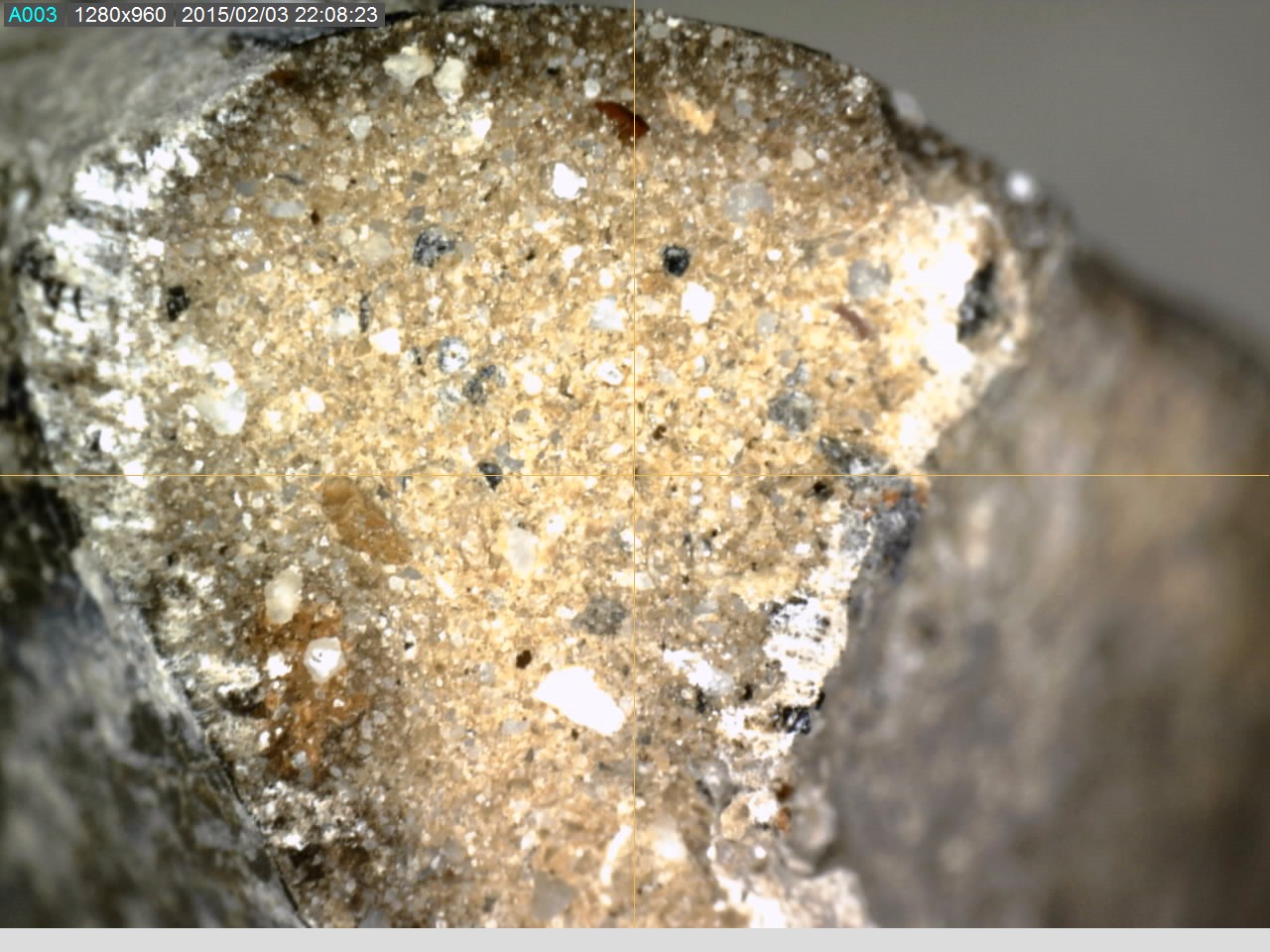

Supplement: Supplementary file 3 — Supplementary material [file mmc3.zip › Appendix A/HTN 6/HTN 6-50m-2.jpg]

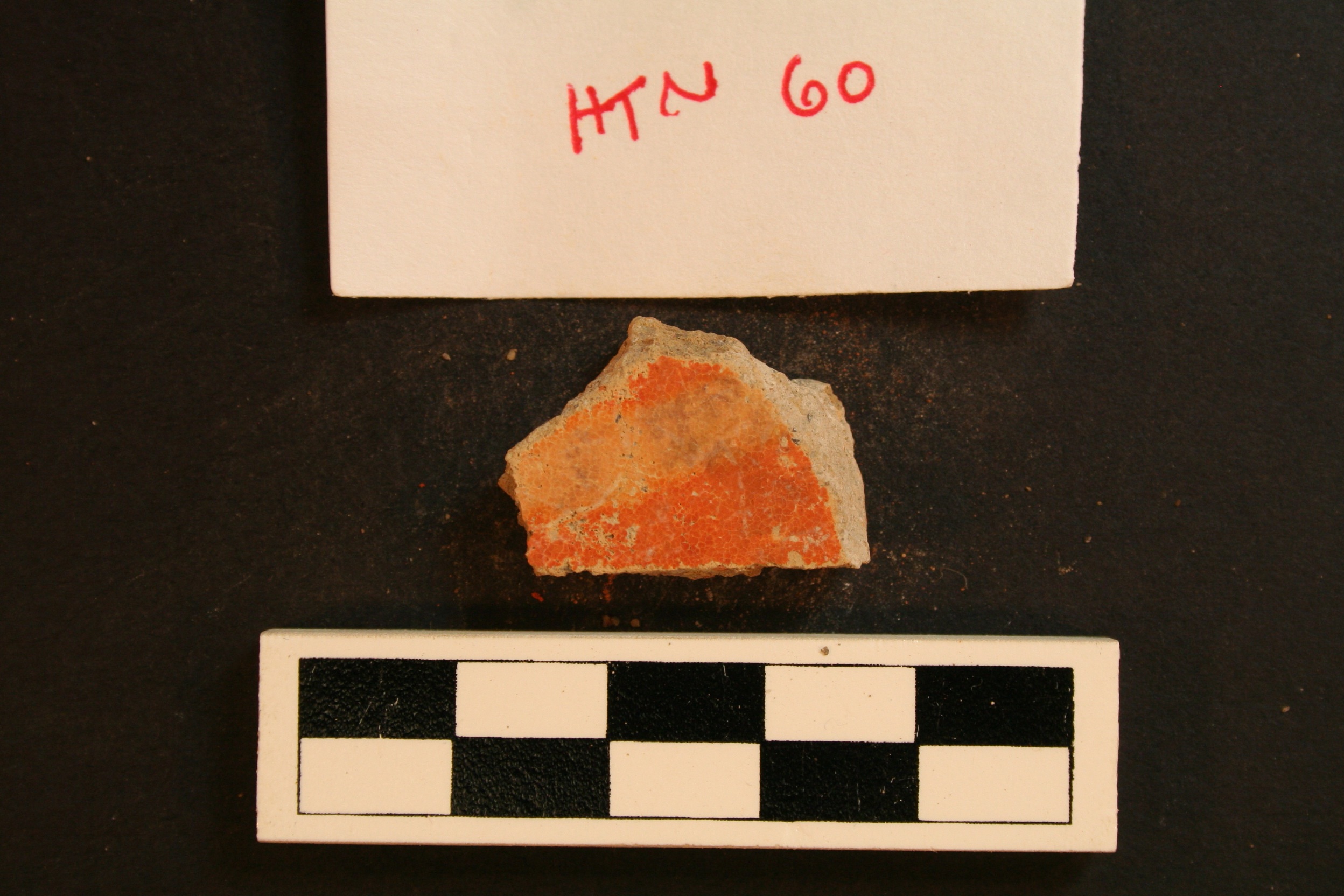

Supplement: Supplementary file 3 — Supplementary material [file mmc3.zip › Appendix A/HTN 60/60a.JPG]

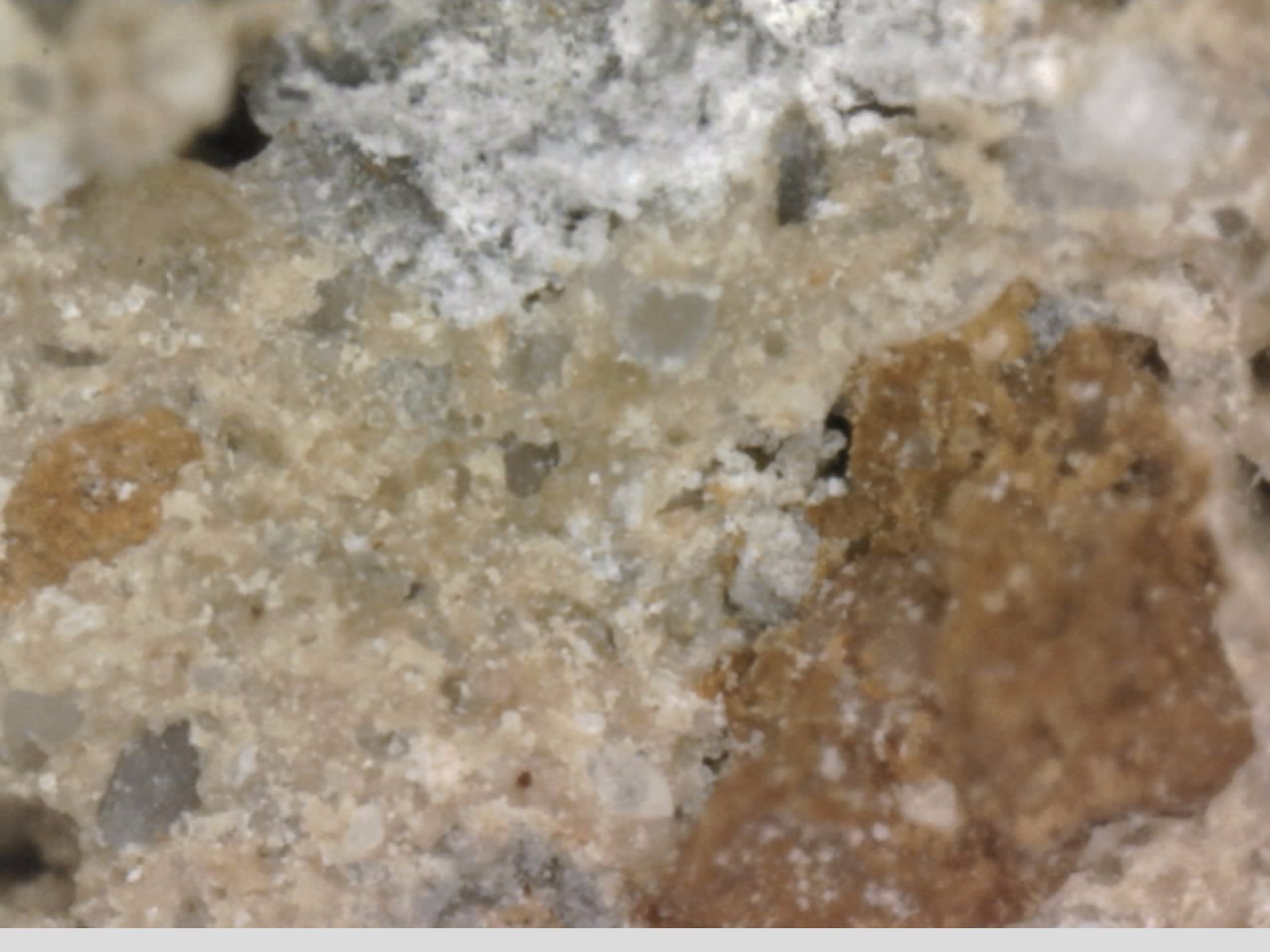

Supplement: Supplementary file 3 — Supplementary material [file mmc3.zip › Appendix A/HTN 60/HTN 60-250m-1.jpg]

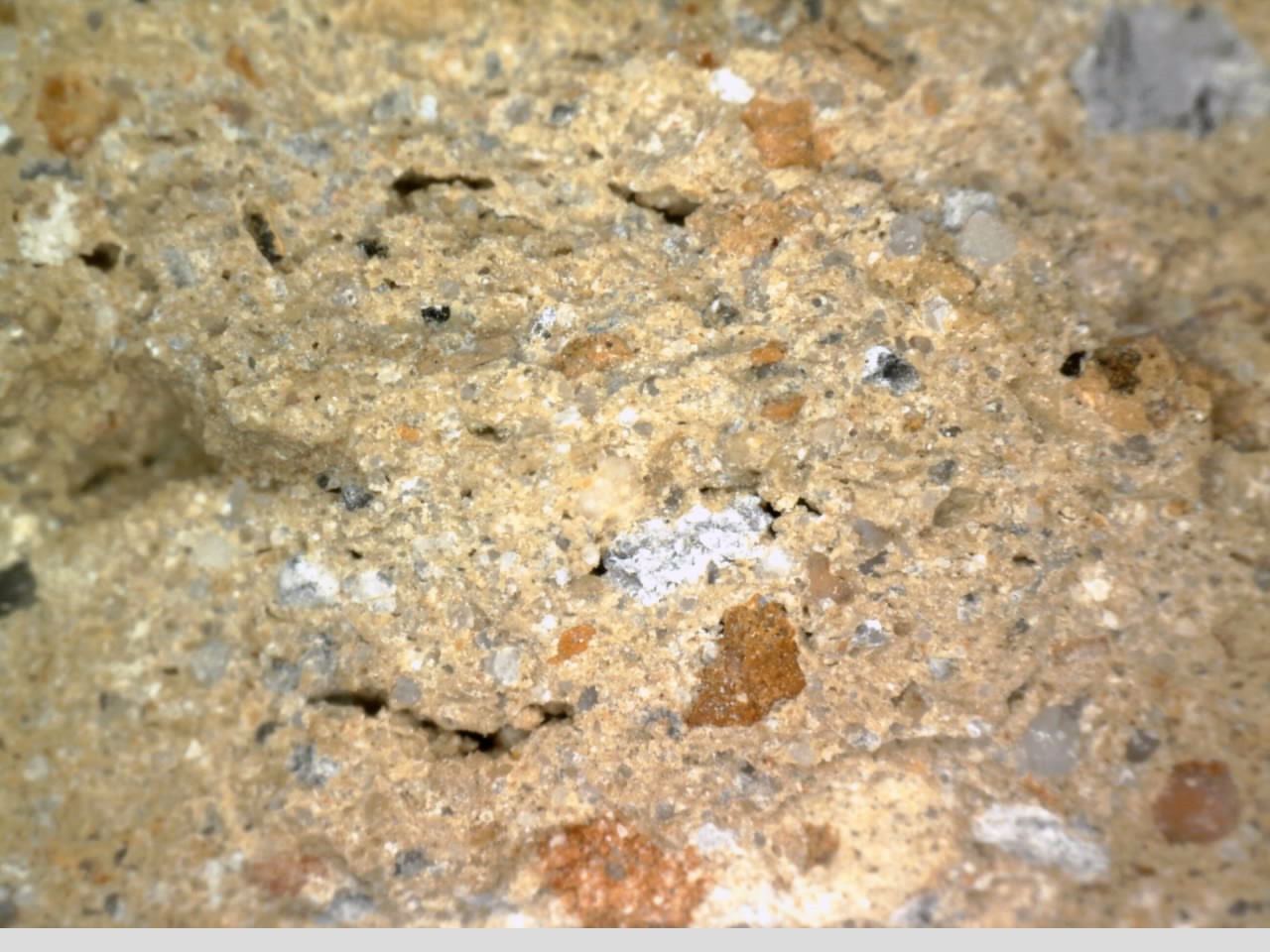

Supplement: Supplementary file 3 — Supplementary material [file mmc3.zip › Appendix A/HTN 60/HTN 60-50m-1.jpg]

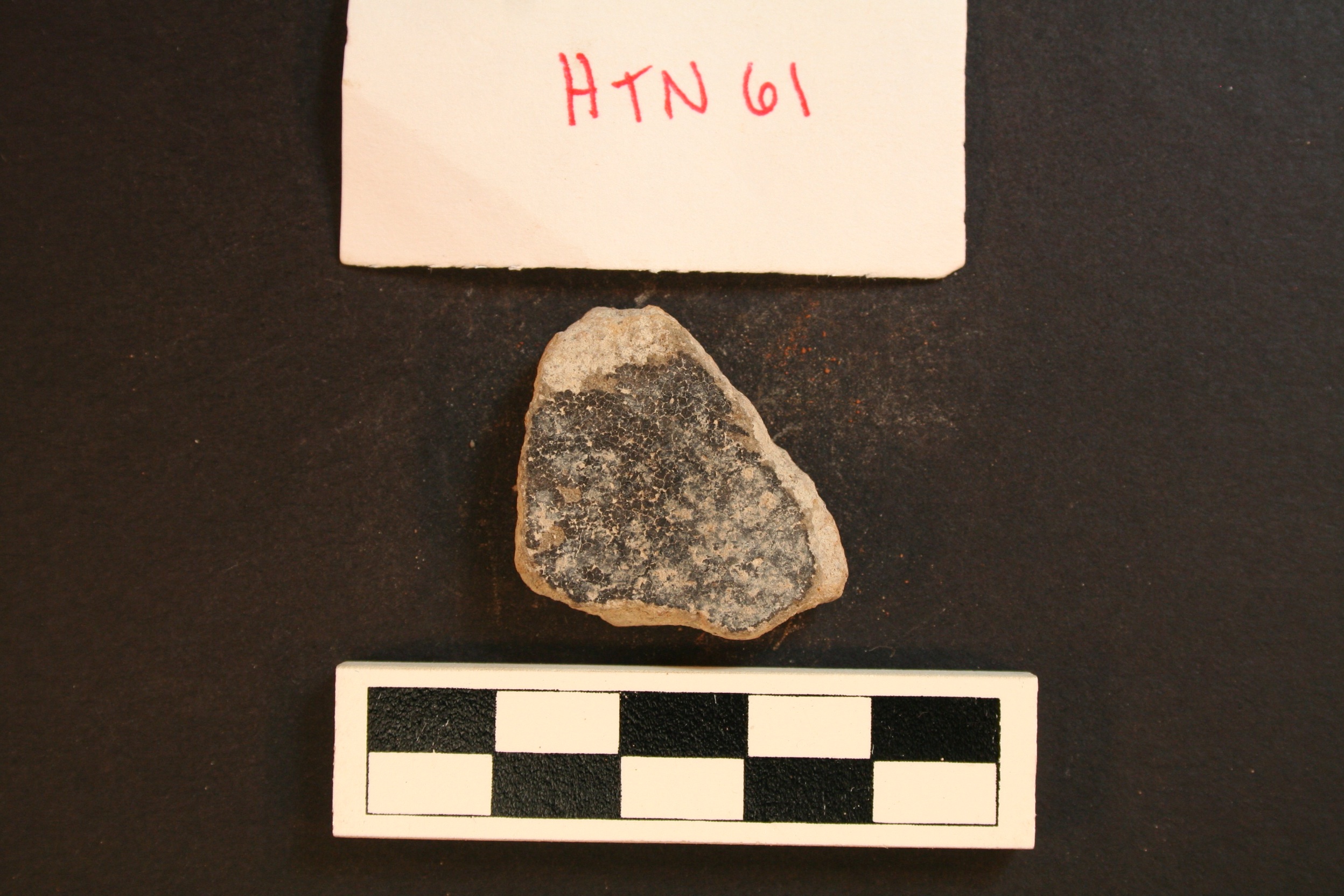

Supplement: Supplementary file 3 — Supplementary material [file mmc3.zip › Appendix A/HTN 61/61a.JPG]

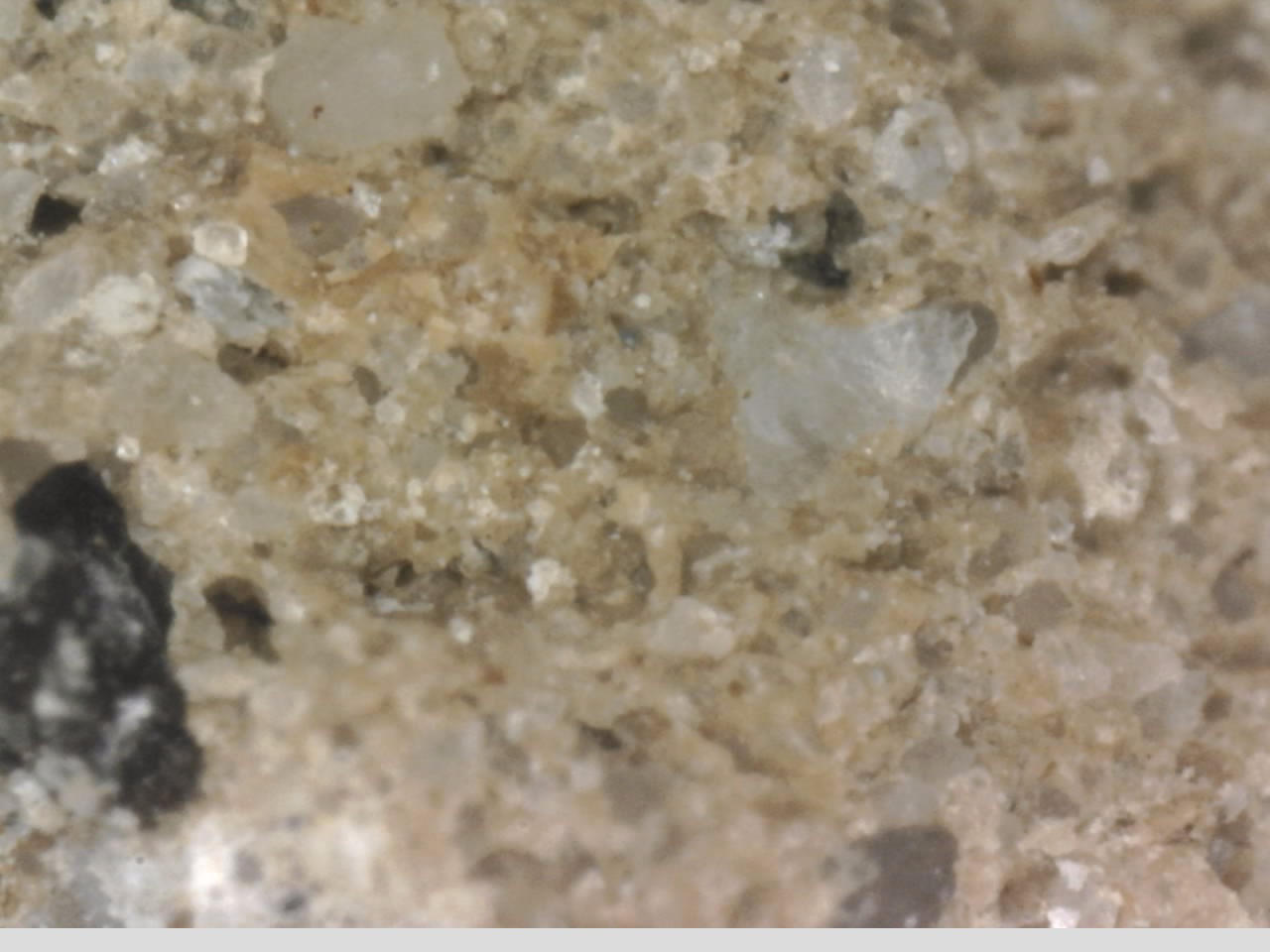

Supplement: Supplementary file 3 — Supplementary material [file mmc3.zip › Appendix A/HTN 61/HTN 61-250m-3.jpg]

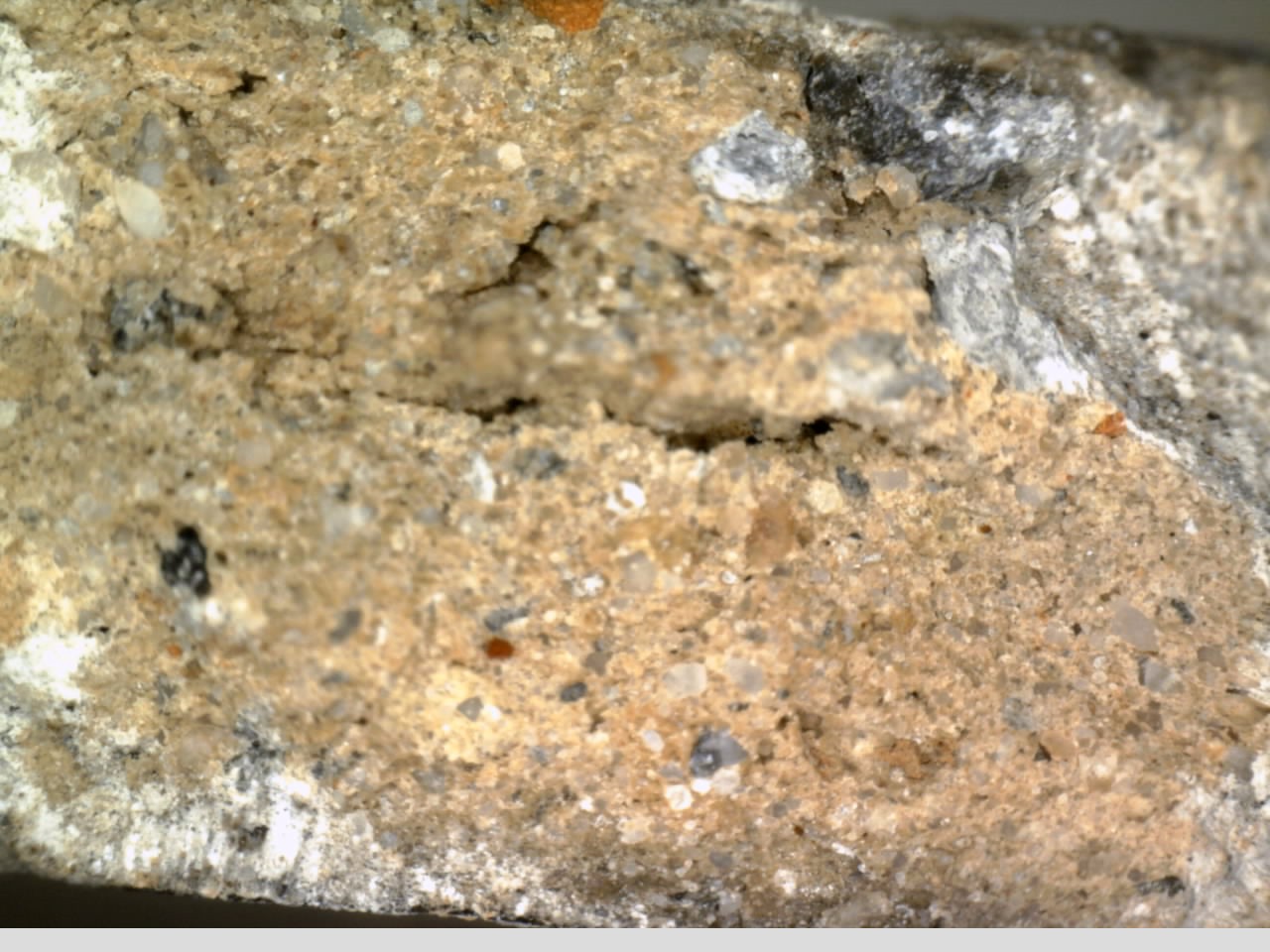

Supplement: Supplementary file 3 — Supplementary material [file mmc3.zip › Appendix A/HTN 61/HTN 61-50m-0.jpg]

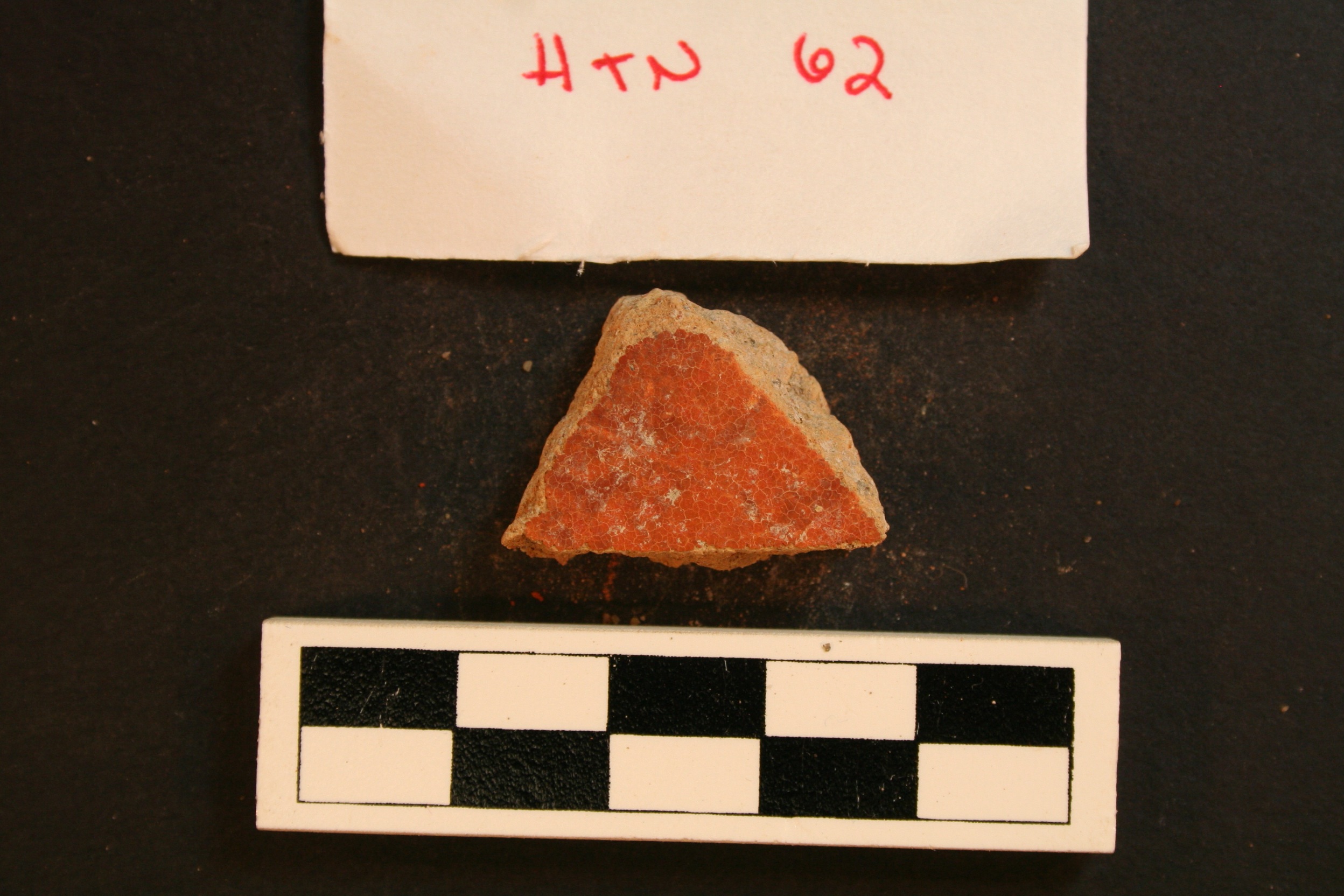

Supplement: Supplementary file 3 — Supplementary material [file mmc3.zip › Appendix A/HTN 62/62a.JPG]

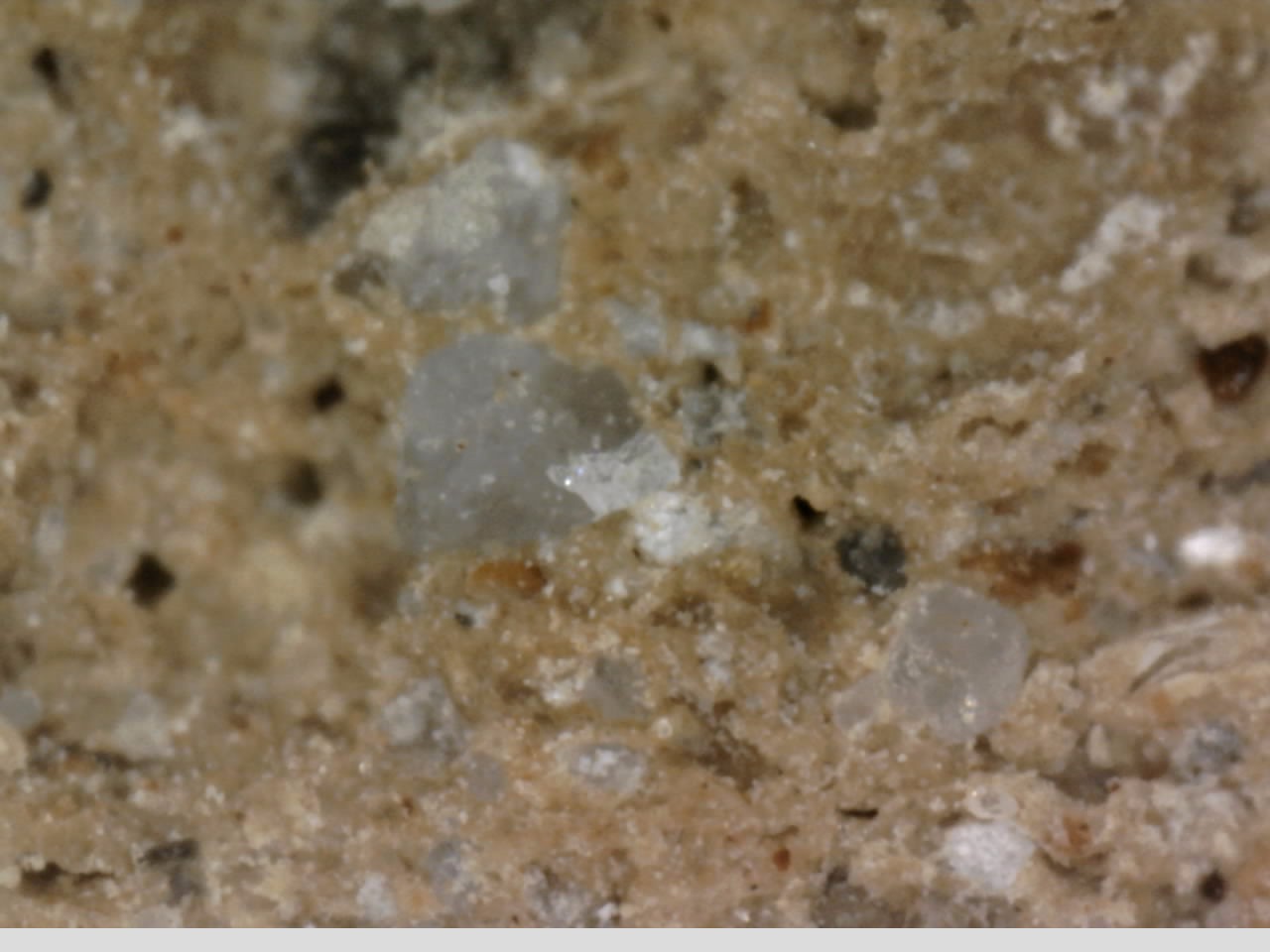

Supplement: Supplementary file 3 — Supplementary material [file mmc3.zip › Appendix A/HTN 62/HTN 62-250m-1.jpg]

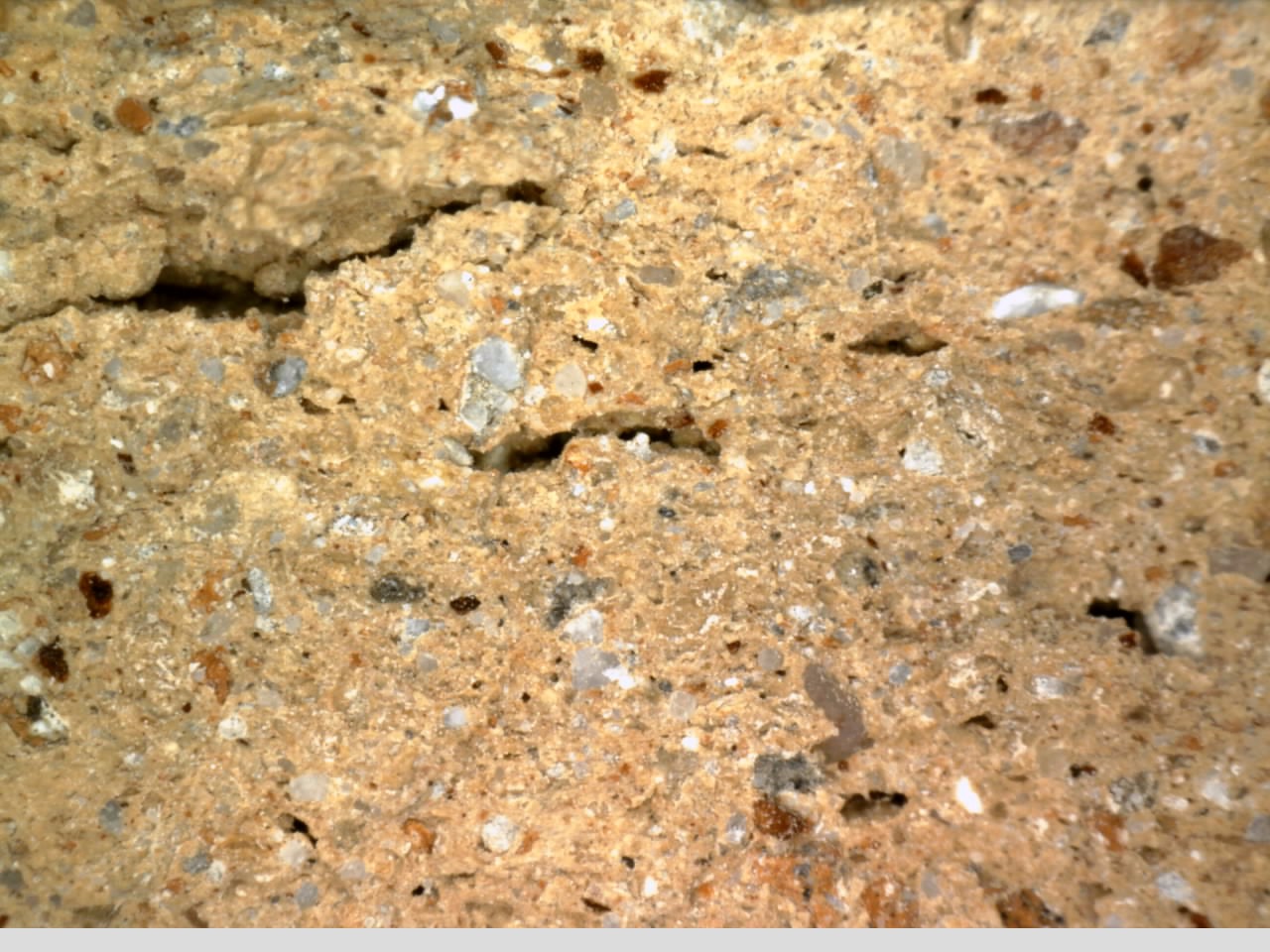

Supplement: Supplementary file 3 — Supplementary material [file mmc3.zip › Appendix A/HTN 62/HTN 62-50m-1.jpg]

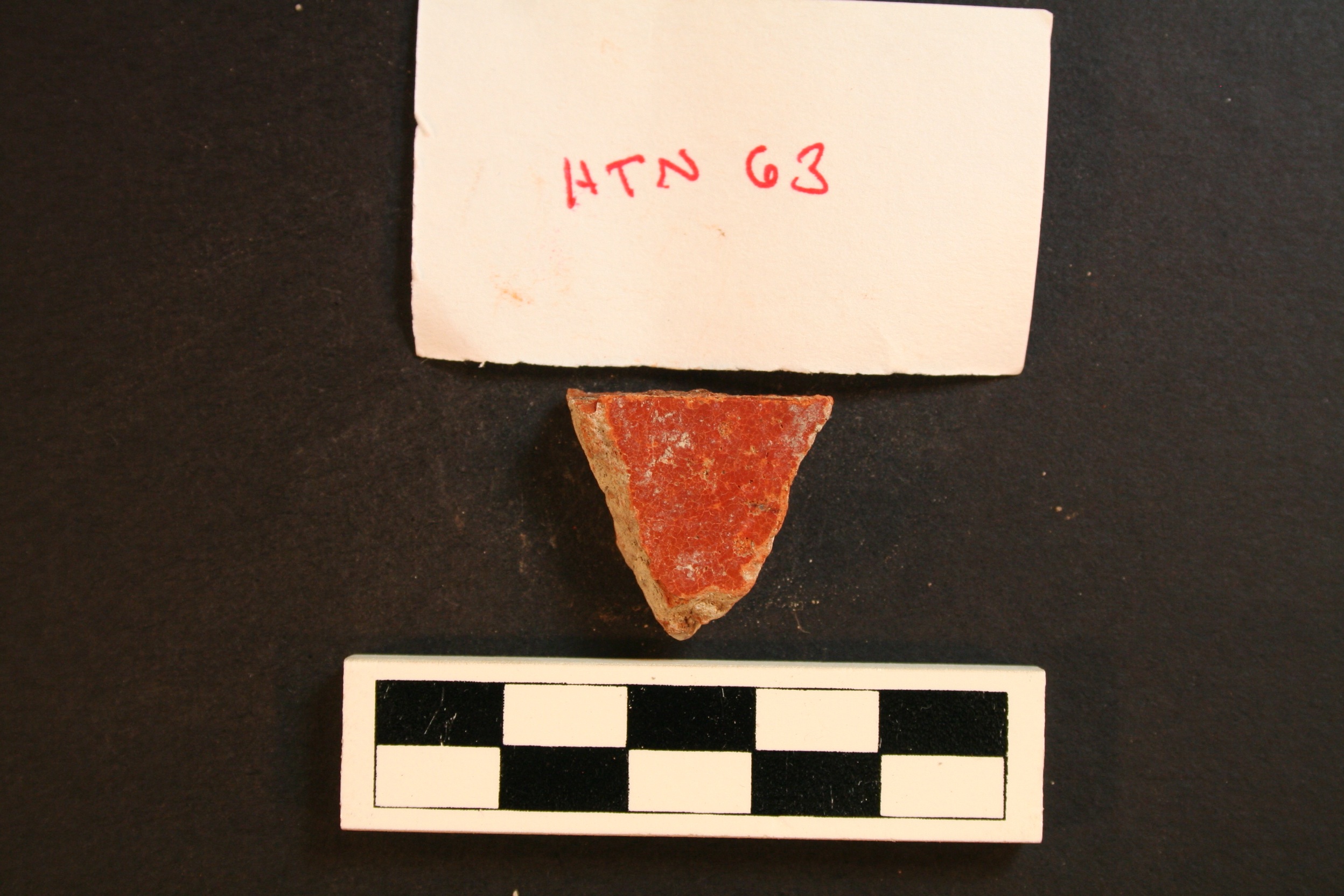

Supplement: Supplementary file 3 — Supplementary material [file mmc3.zip › Appendix A/HTN 63/63a.JPG]

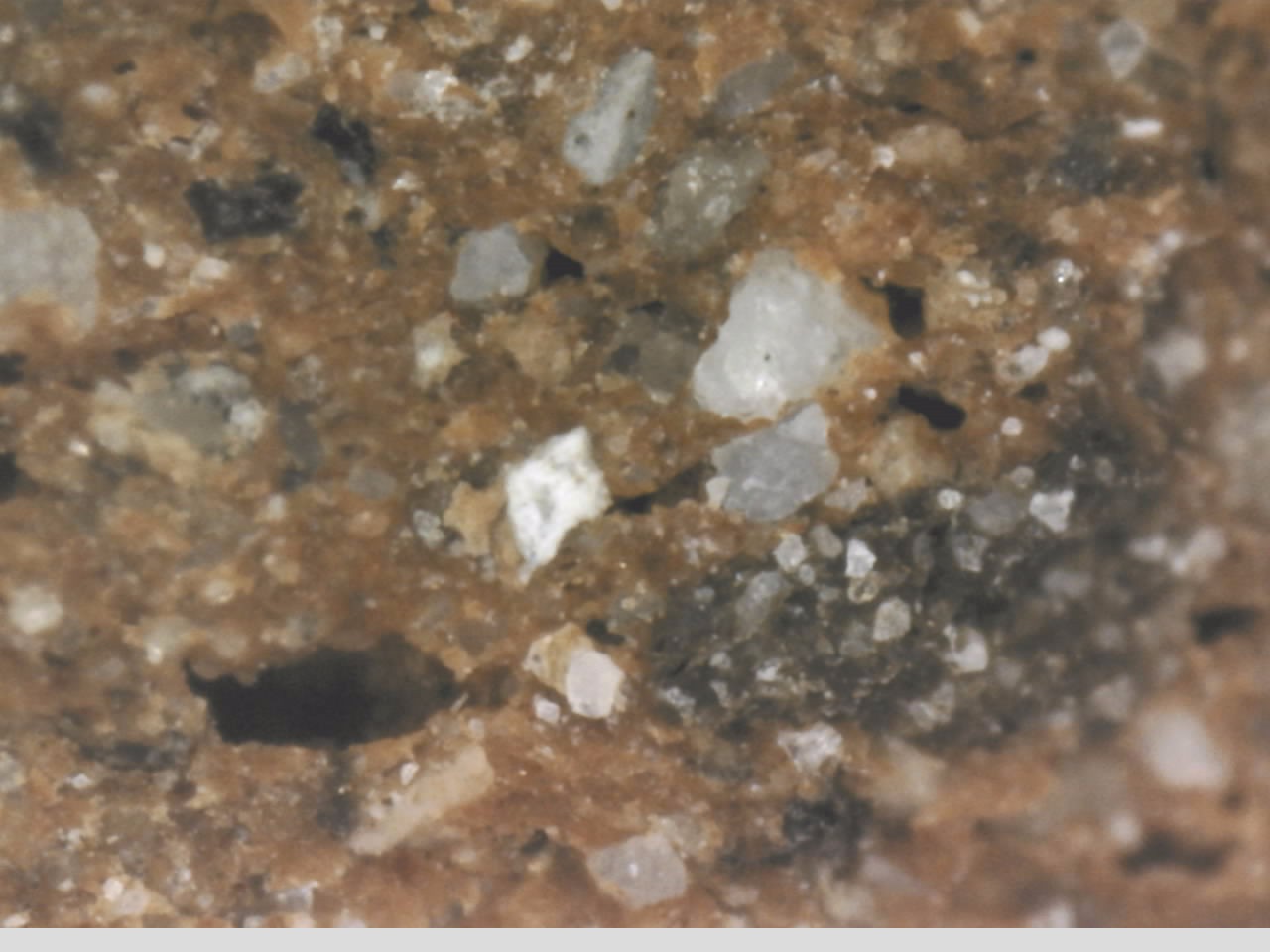

Supplement: Supplementary file 3 — Supplementary material [file mmc3.zip › Appendix A/HTN 63/HTN 63-250m-7.jpg]

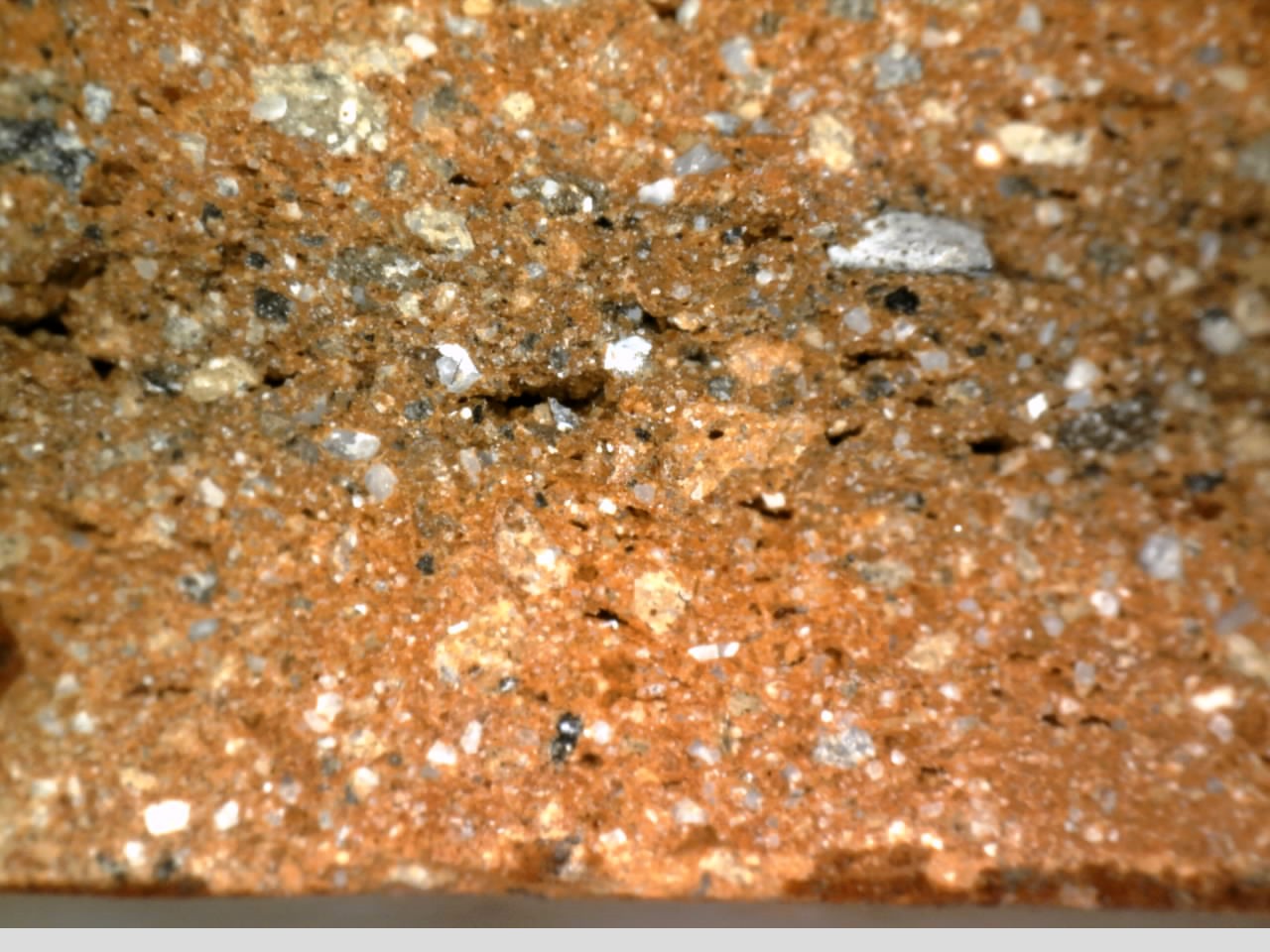

Supplement: Supplementary file 3 — Supplementary material [file mmc3.zip › Appendix A/HTN 63/HTn 63-50m-2.jpg]

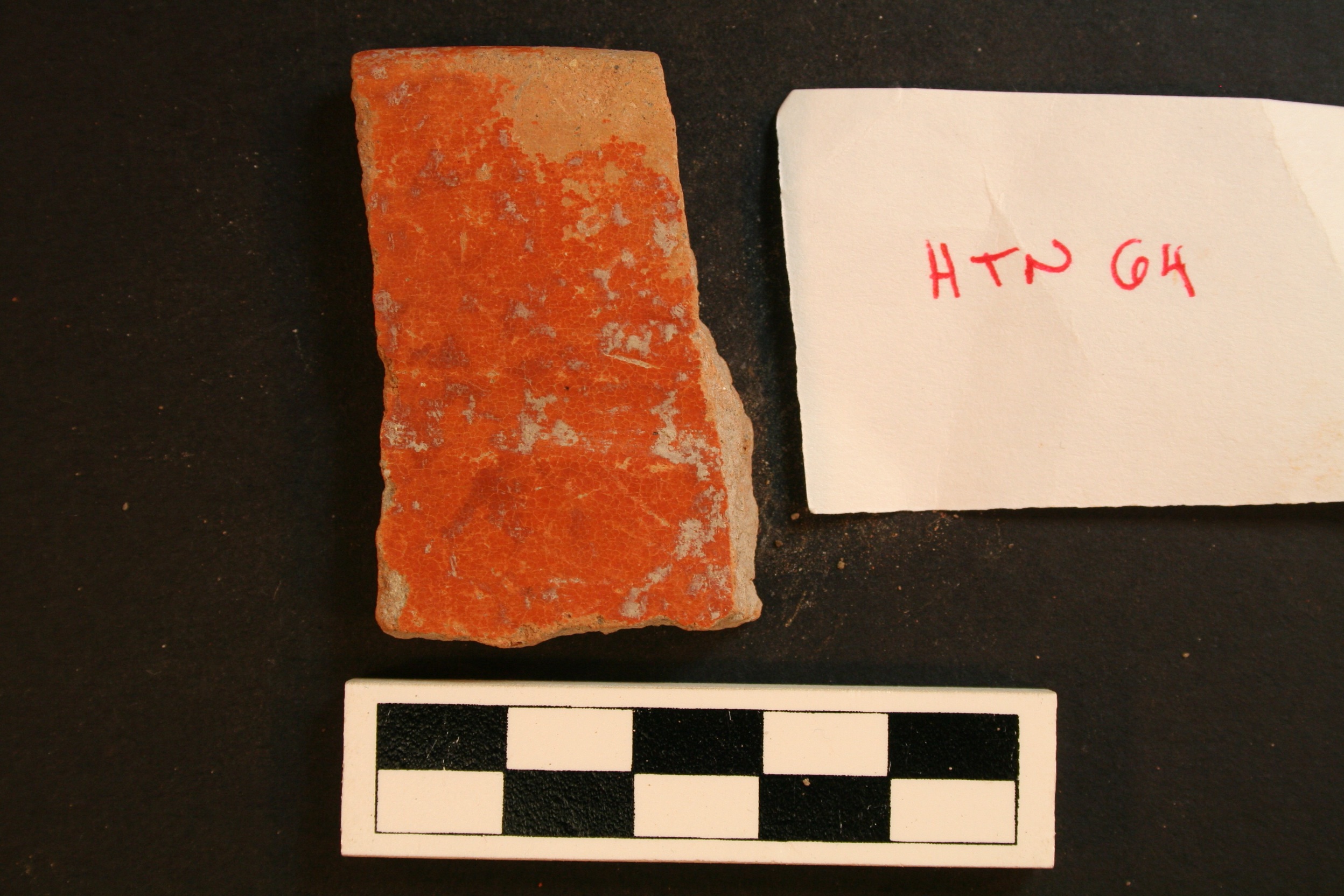

Supplement: Supplementary file 3 — Supplementary material [file mmc3.zip › Appendix A/HTN 64/64a.JPG]

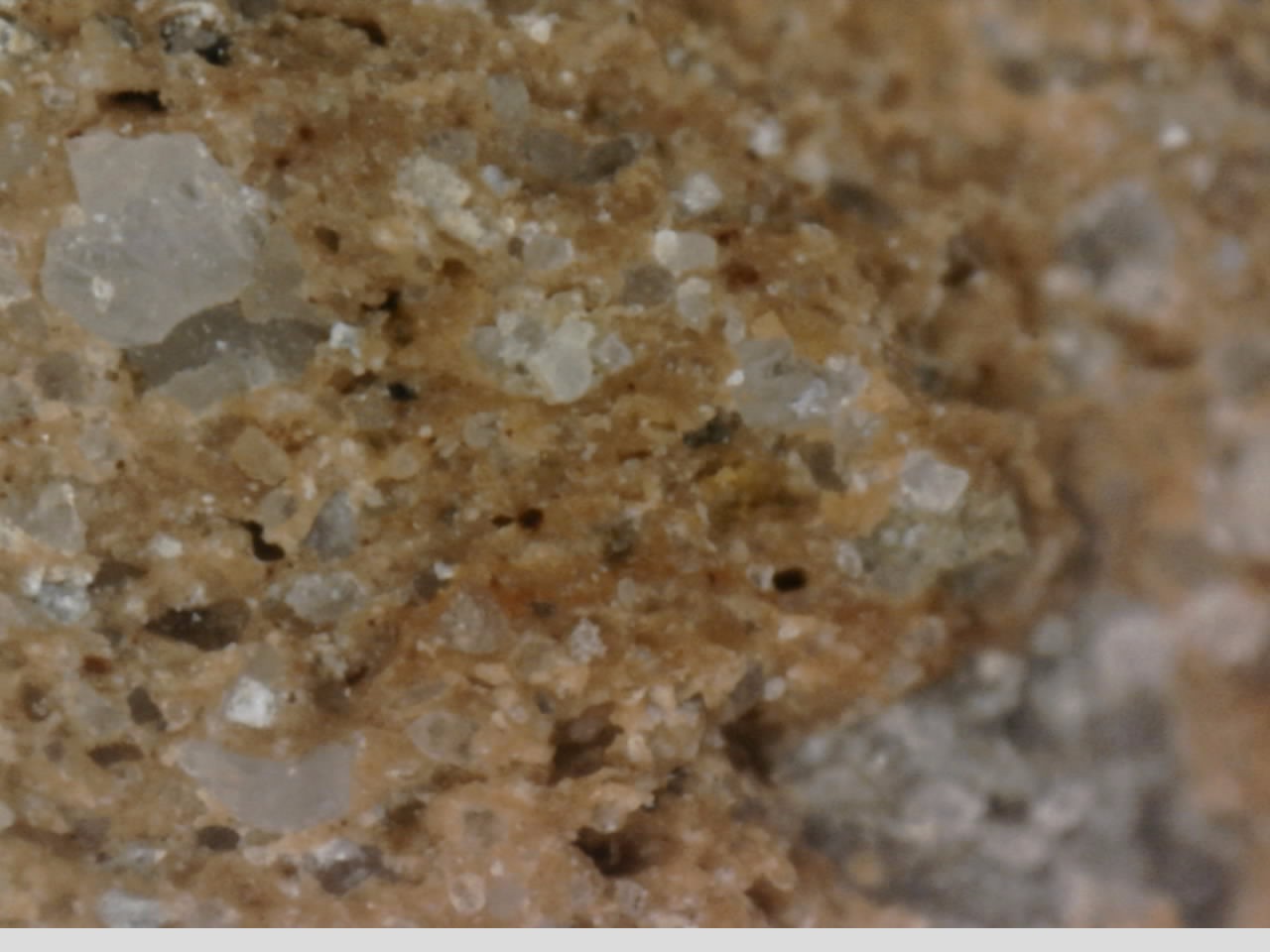

Supplement: Supplementary file 3 — Supplementary material [file mmc3.zip › Appendix A/HTN 64/HTN 64-250m-0.jpg]

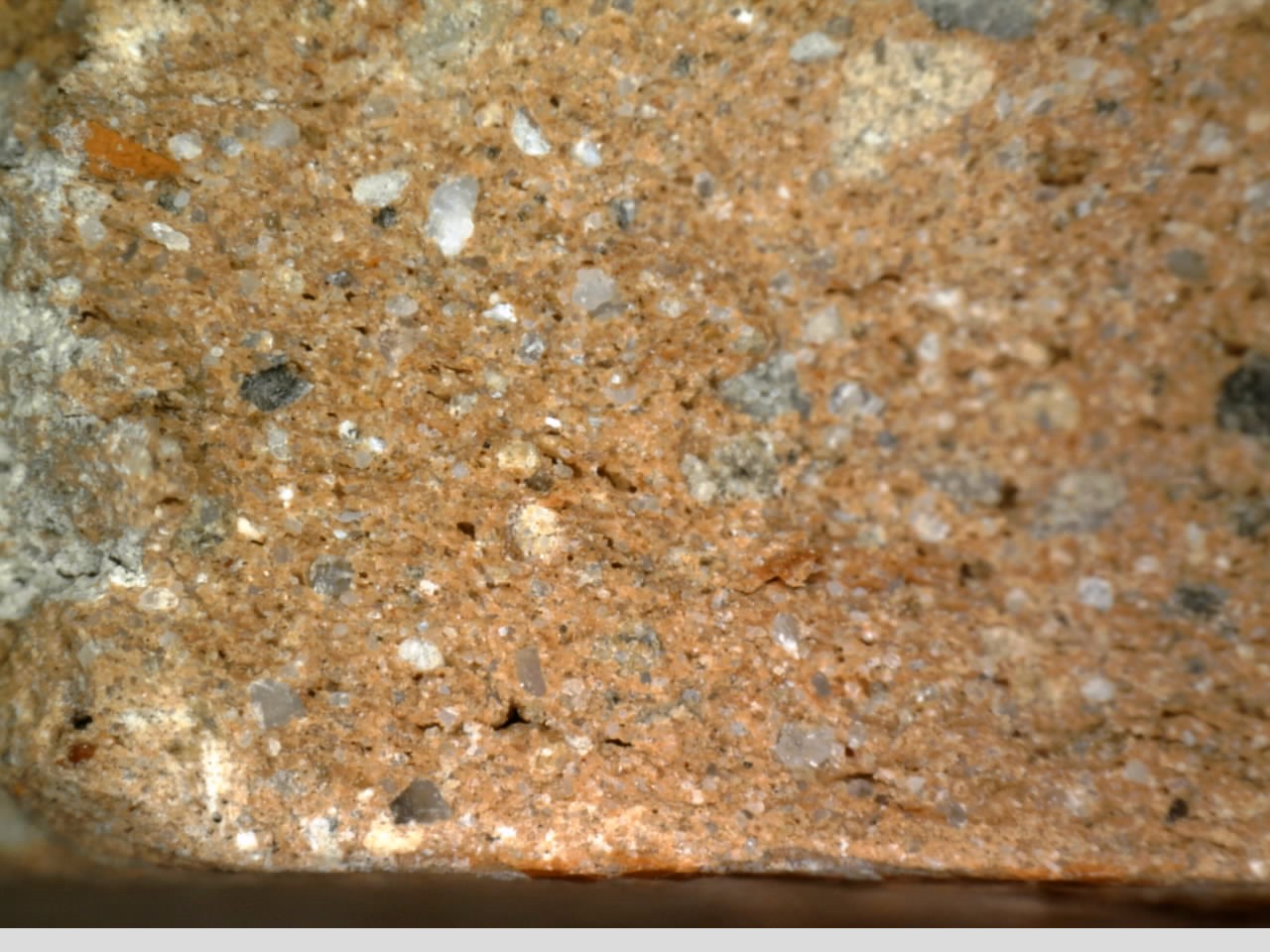

Supplement: Supplementary file 3 — Supplementary material [file mmc3.zip › Appendix A/HTN 64/HTN 64-50m-1.jpg]

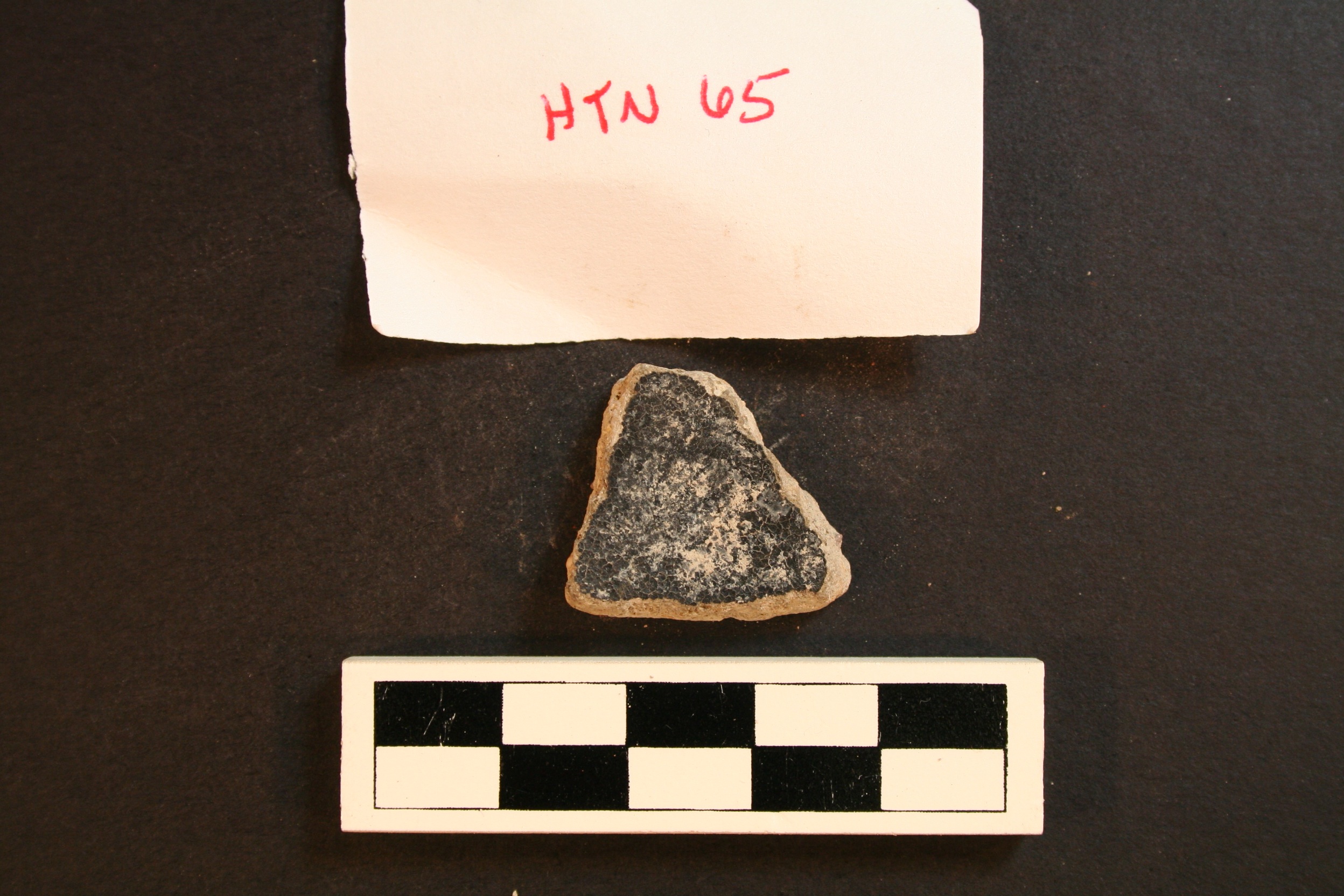

Supplement: Supplementary file 3 — Supplementary material [file mmc3.zip › Appendix A/HTN 65/65a.JPG]

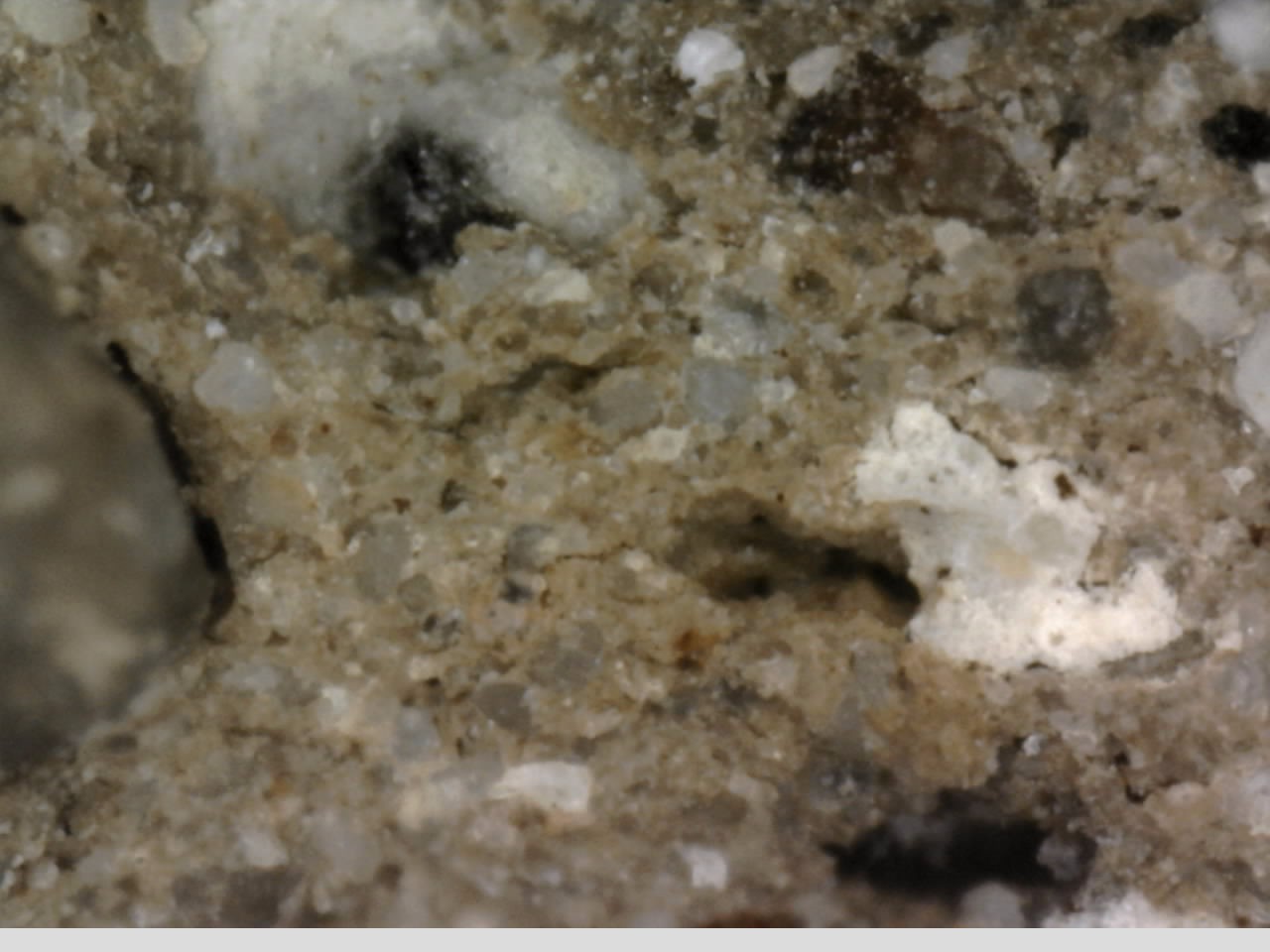

Supplement: Supplementary file 3 — Supplementary material [file mmc3.zip › Appendix A/HTN 65/HTN 65-250m-1.jpg]

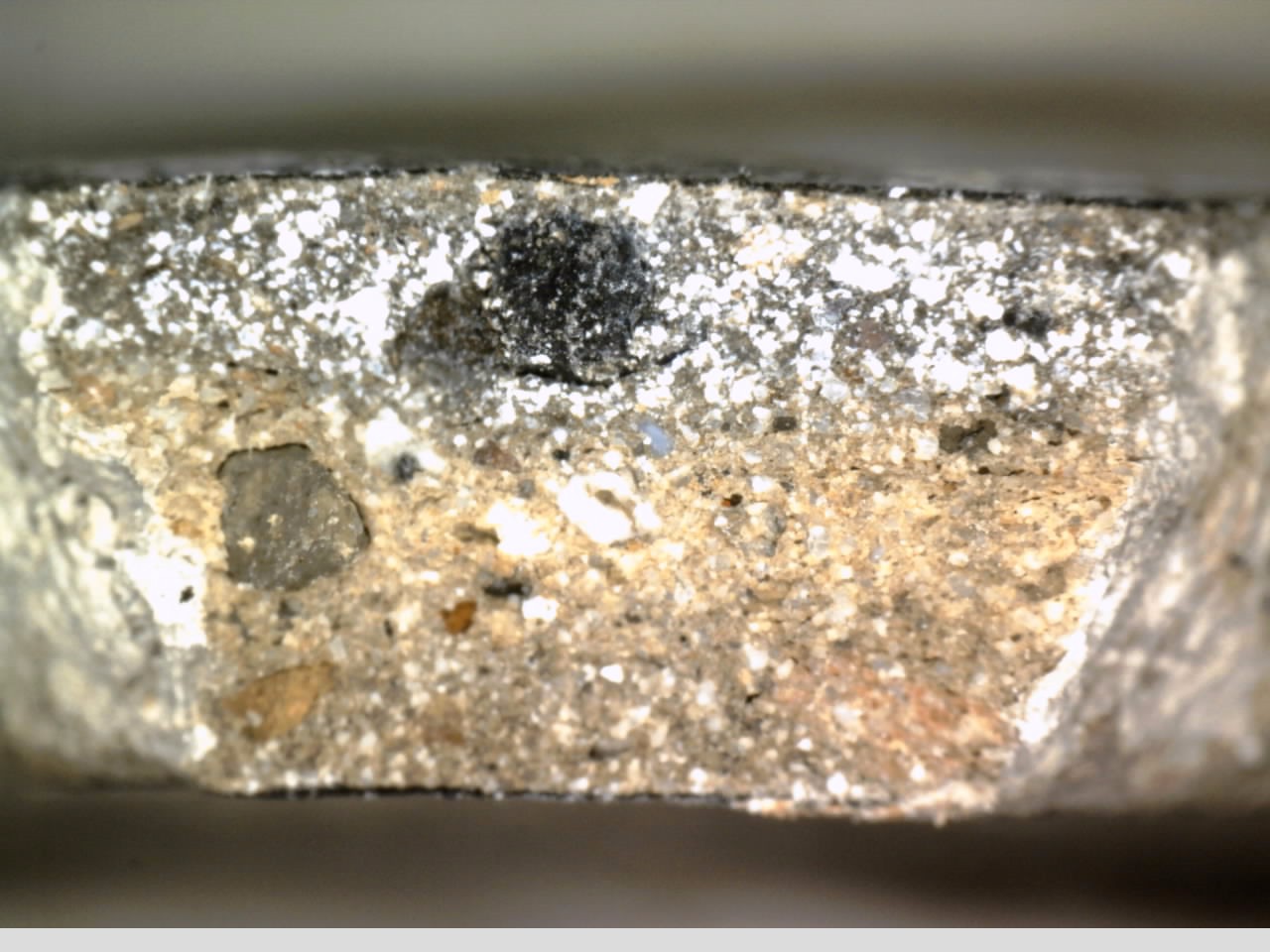

Supplement: Supplementary file 3 — Supplementary material [file mmc3.zip › Appendix A/HTN 65/HTN 65-50m-3.jpg]

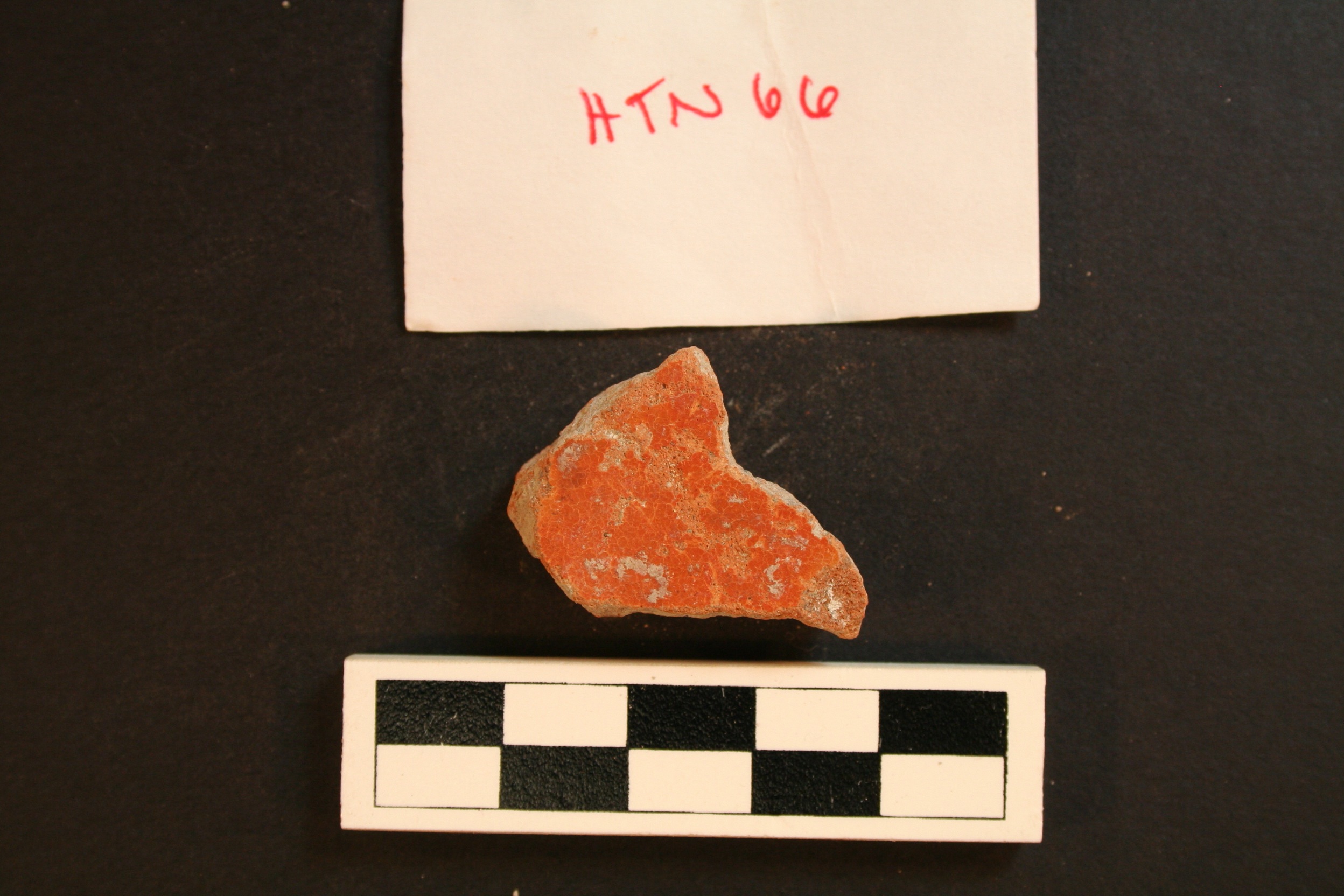

Supplement: Supplementary file 3 — Supplementary material [file mmc3.zip › Appendix A/HTN 66/66a.JPG]

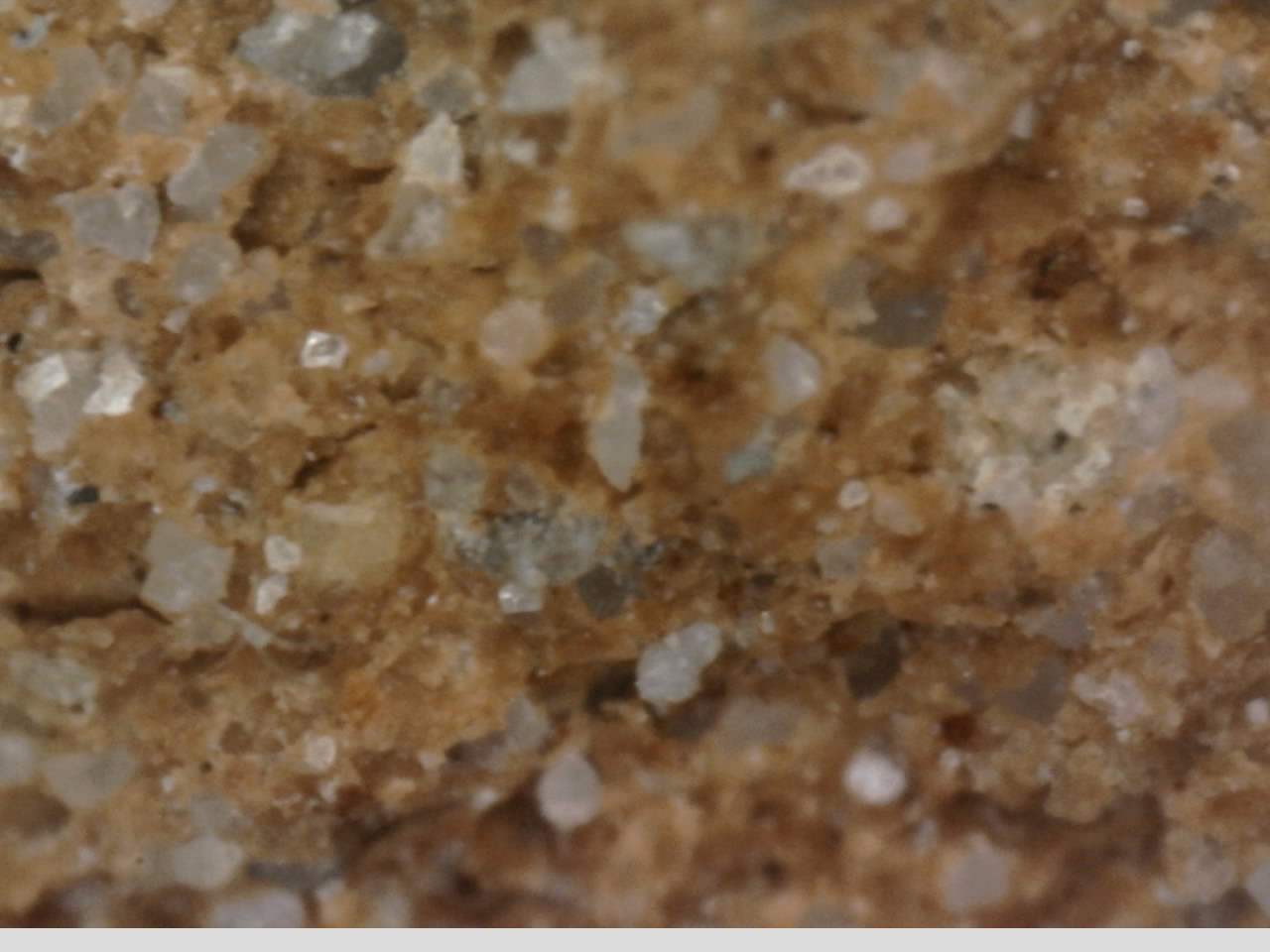

Supplement: Supplementary file 3 — Supplementary material [file mmc3.zip › Appendix A/HTN 66/HTN 66-250m-0.jpg]

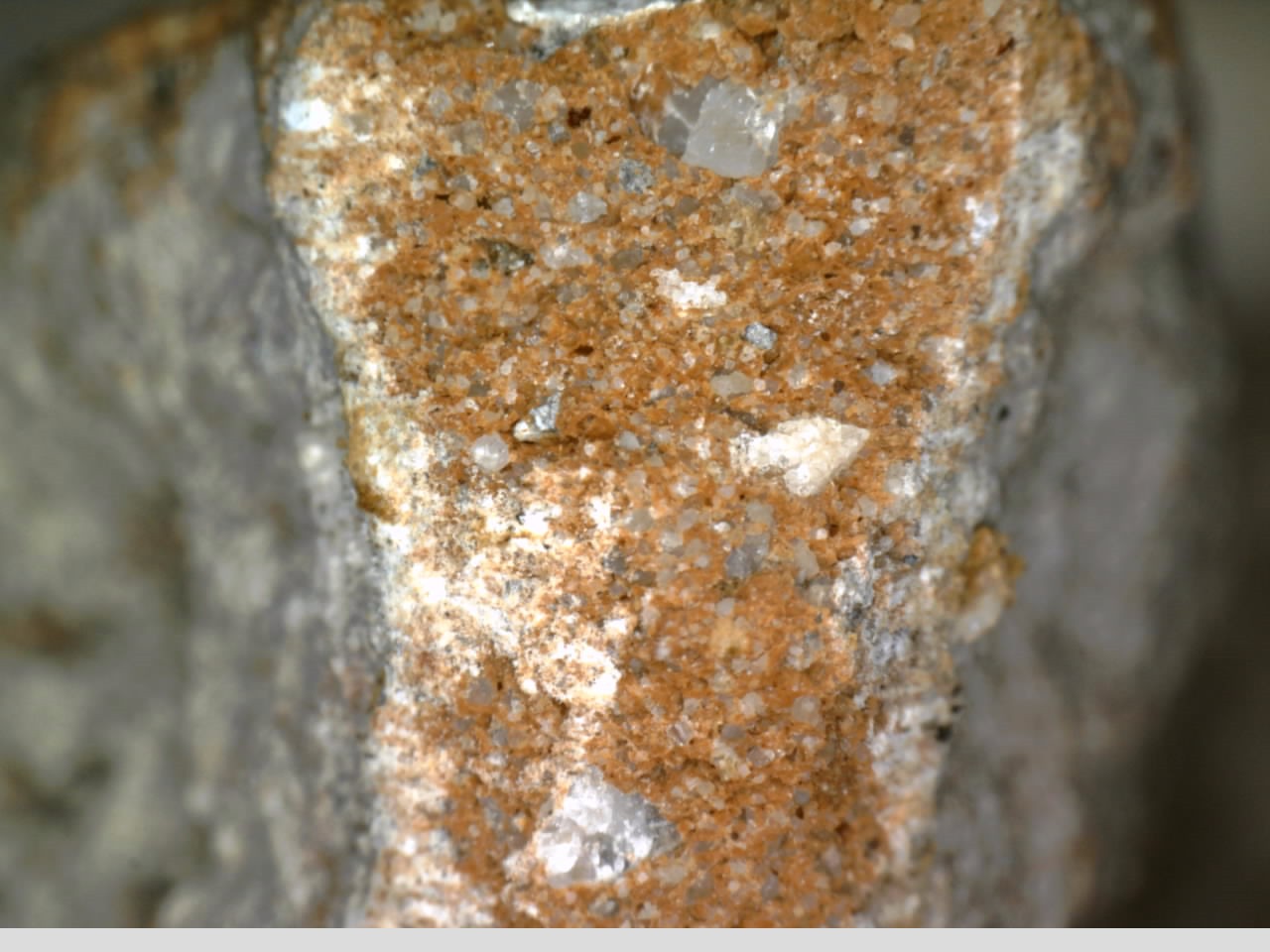

Supplement: Supplementary file 3 — Supplementary material [file mmc3.zip › Appendix A/HTN 66/HTN 66-50m-2.jpg]

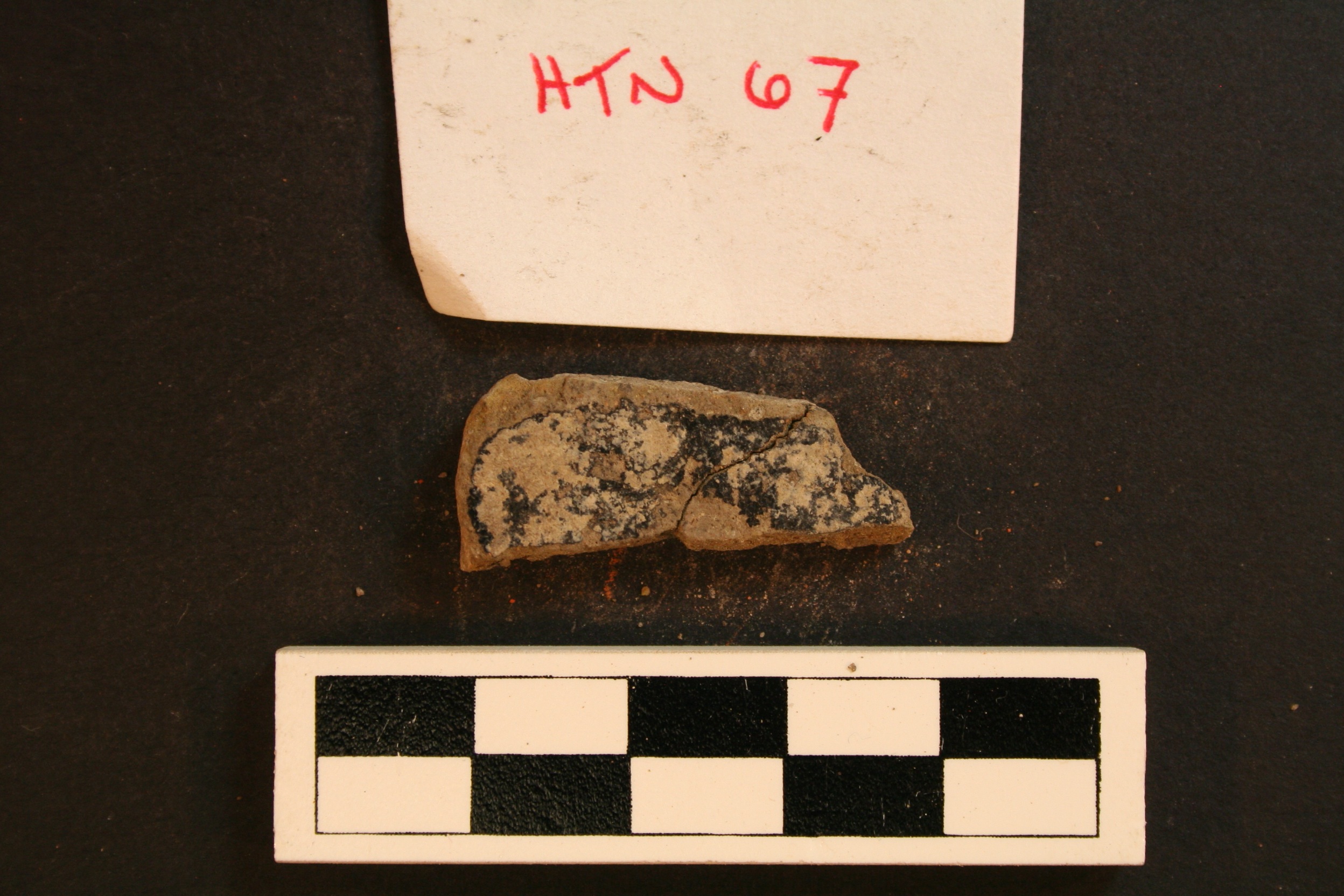

Supplement: Supplementary file 3 — Supplementary material [file mmc3.zip › Appendix A/HTN 67/67a.JPG]

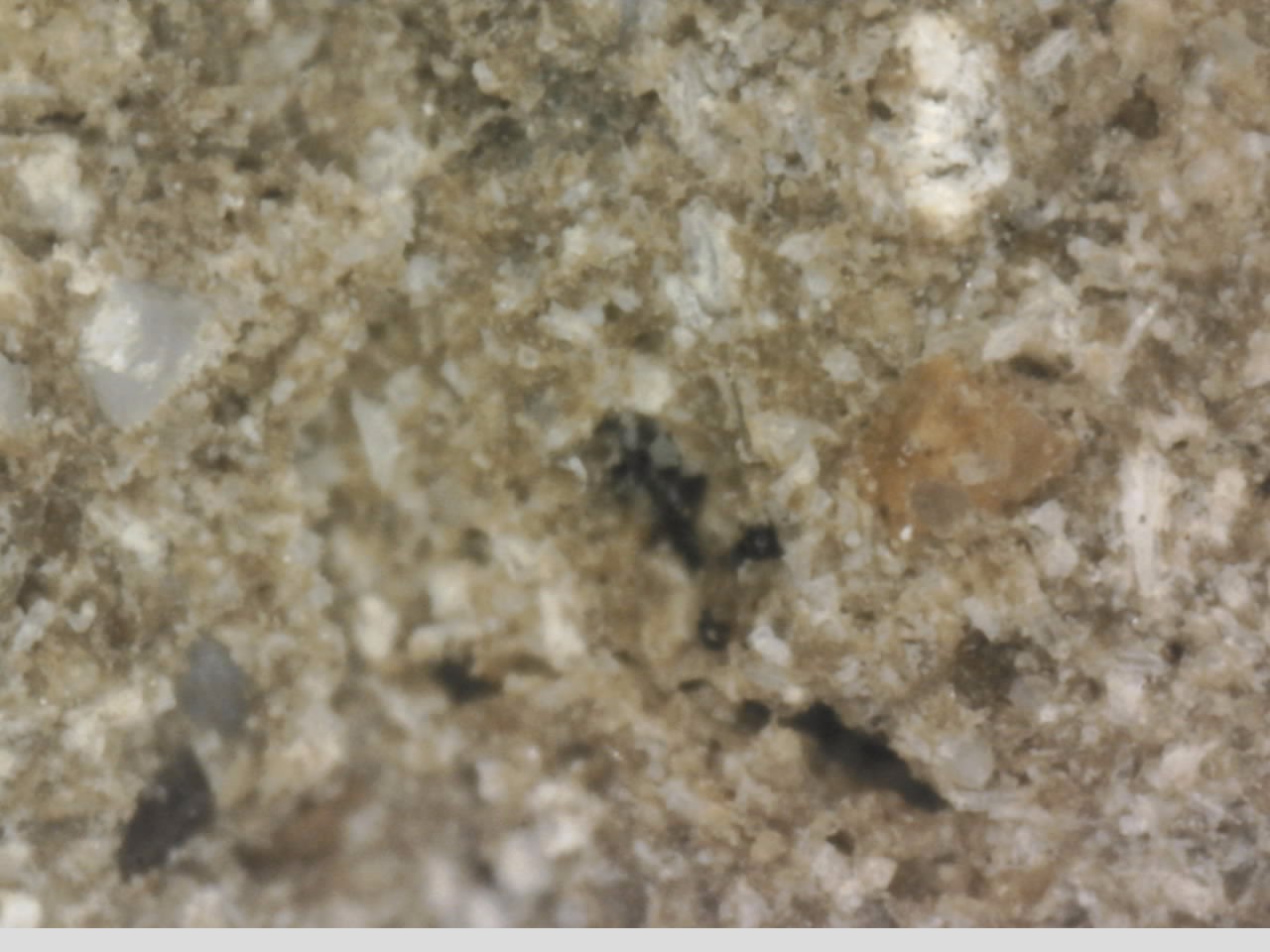

Supplement: Supplementary file 3 — Supplementary material [file mmc3.zip › Appendix A/HTN 67/HTN 67-250m-4.jpg]

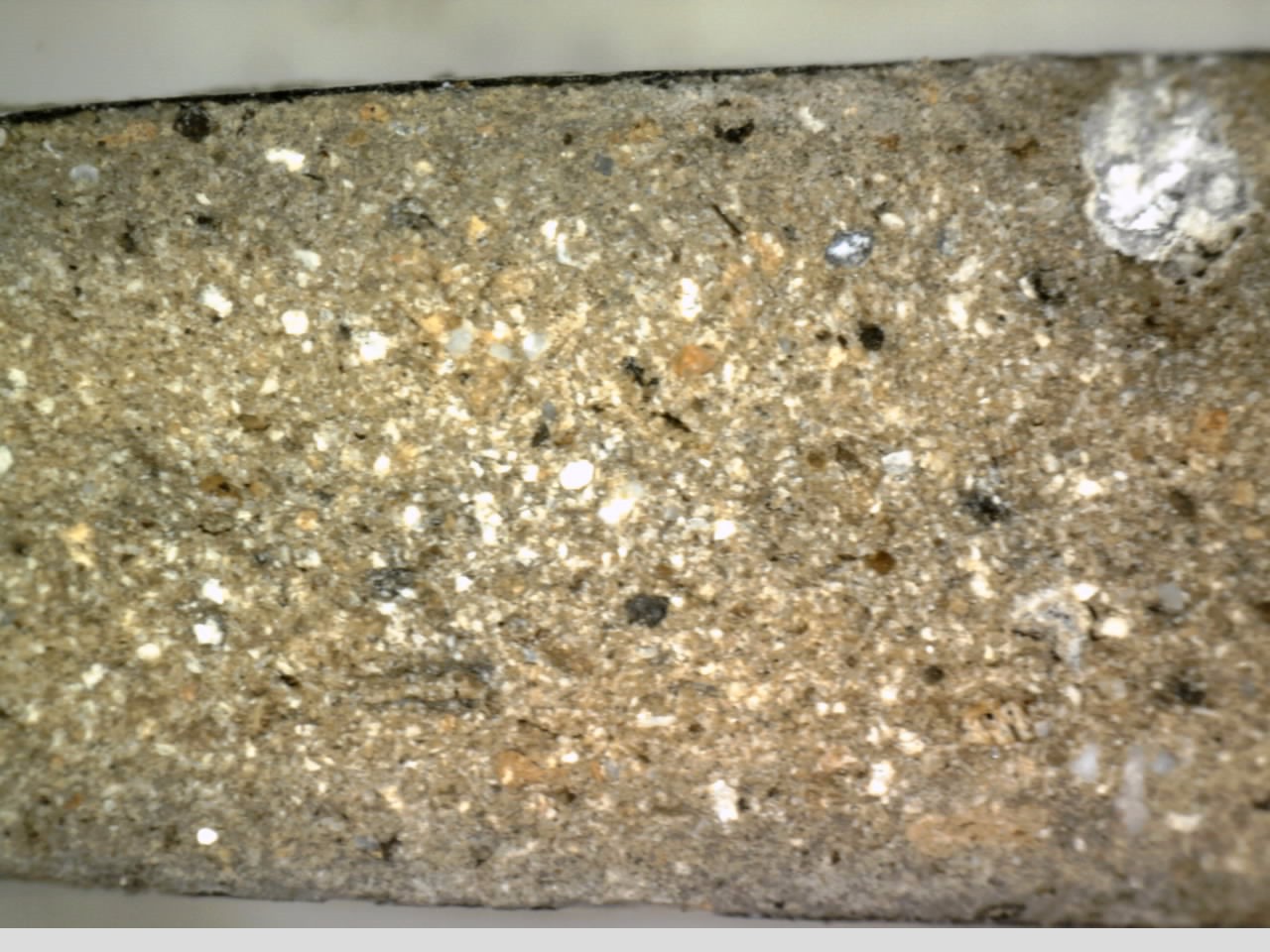

Supplement: Supplementary file 3 — Supplementary material [file mmc3.zip › Appendix A/HTN 67/HTN 67-50m-3.jpg]

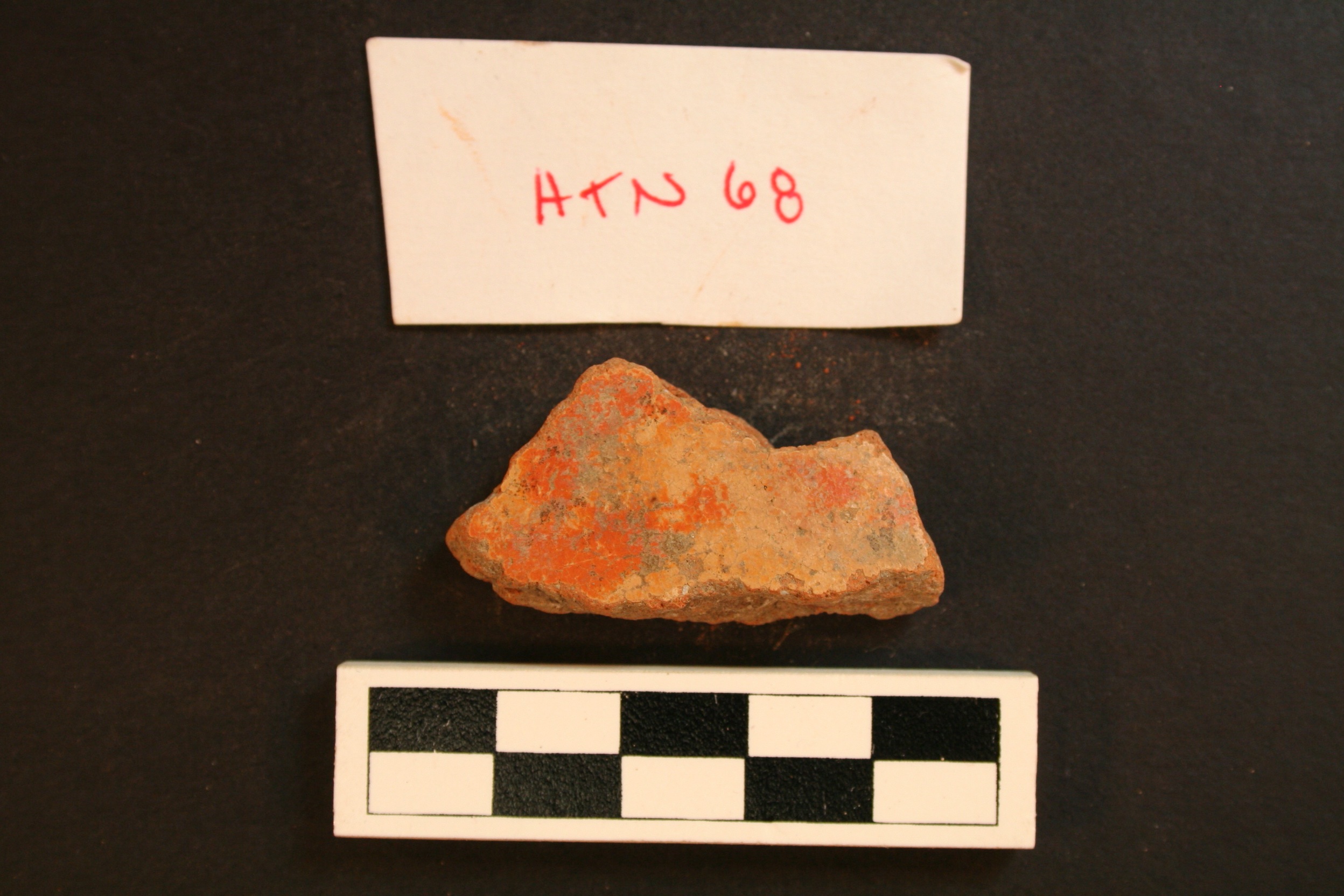

Supplement: Supplementary file 3 — Supplementary material [file mmc3.zip › Appendix A/HTN 68/68a.JPG]

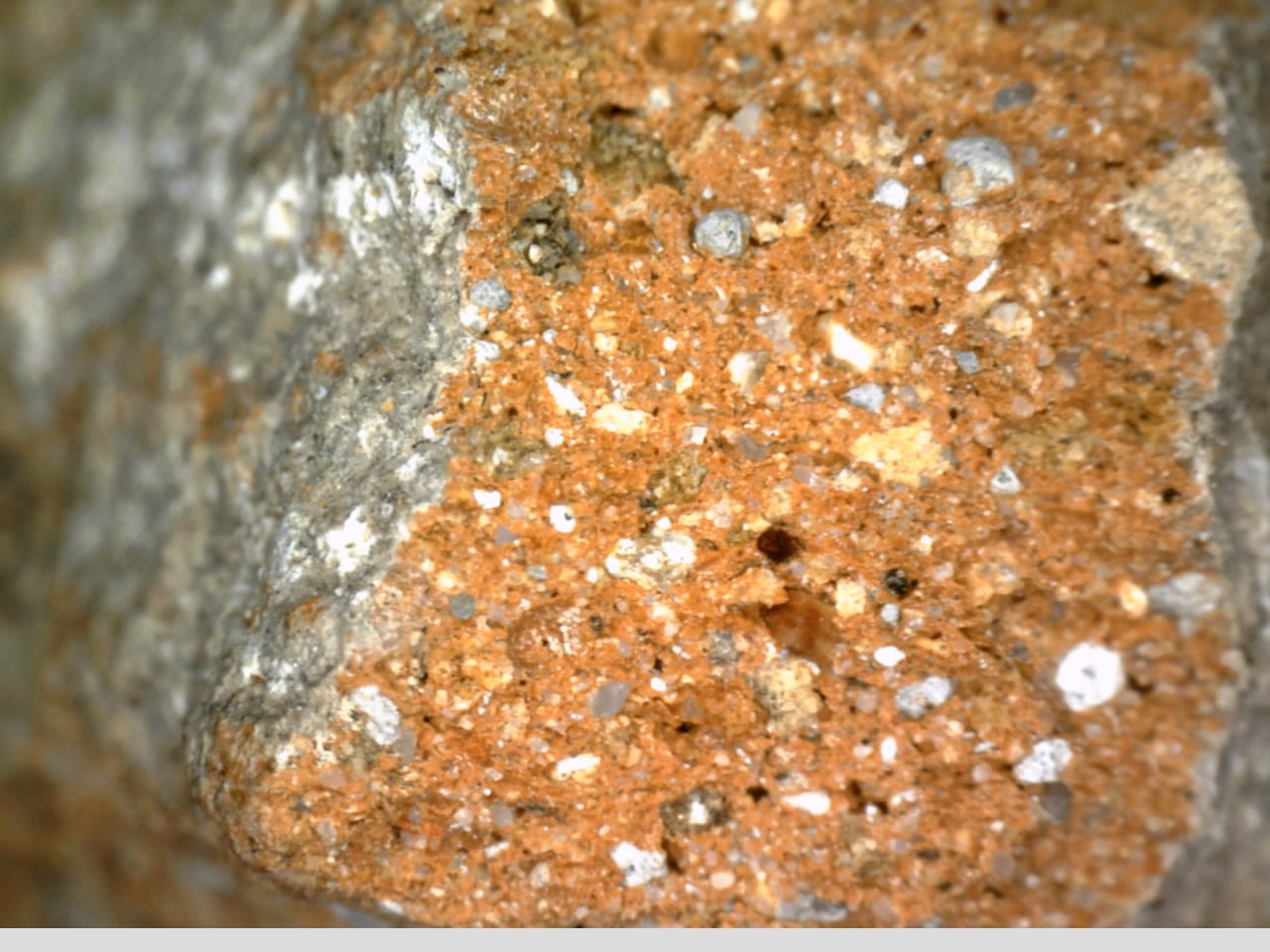

Supplement: Supplementary file 3 — Supplementary material [file mmc3.zip › Appendix A/HTN 68/HTN 68-50m-3.jpg]

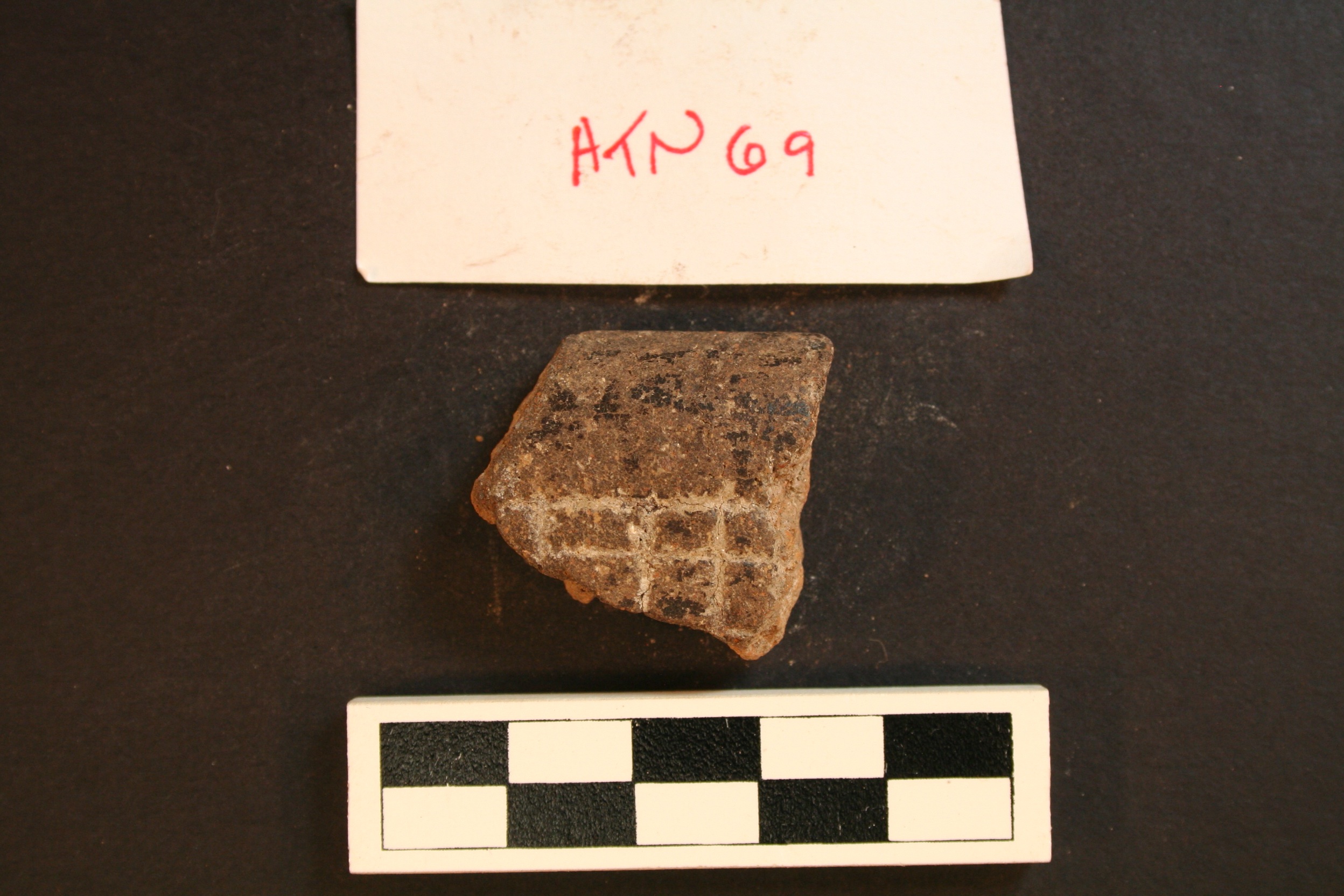

Supplement: Supplementary file 3 — Supplementary material [file mmc3.zip › Appendix A/HTN 69/69a.JPG]

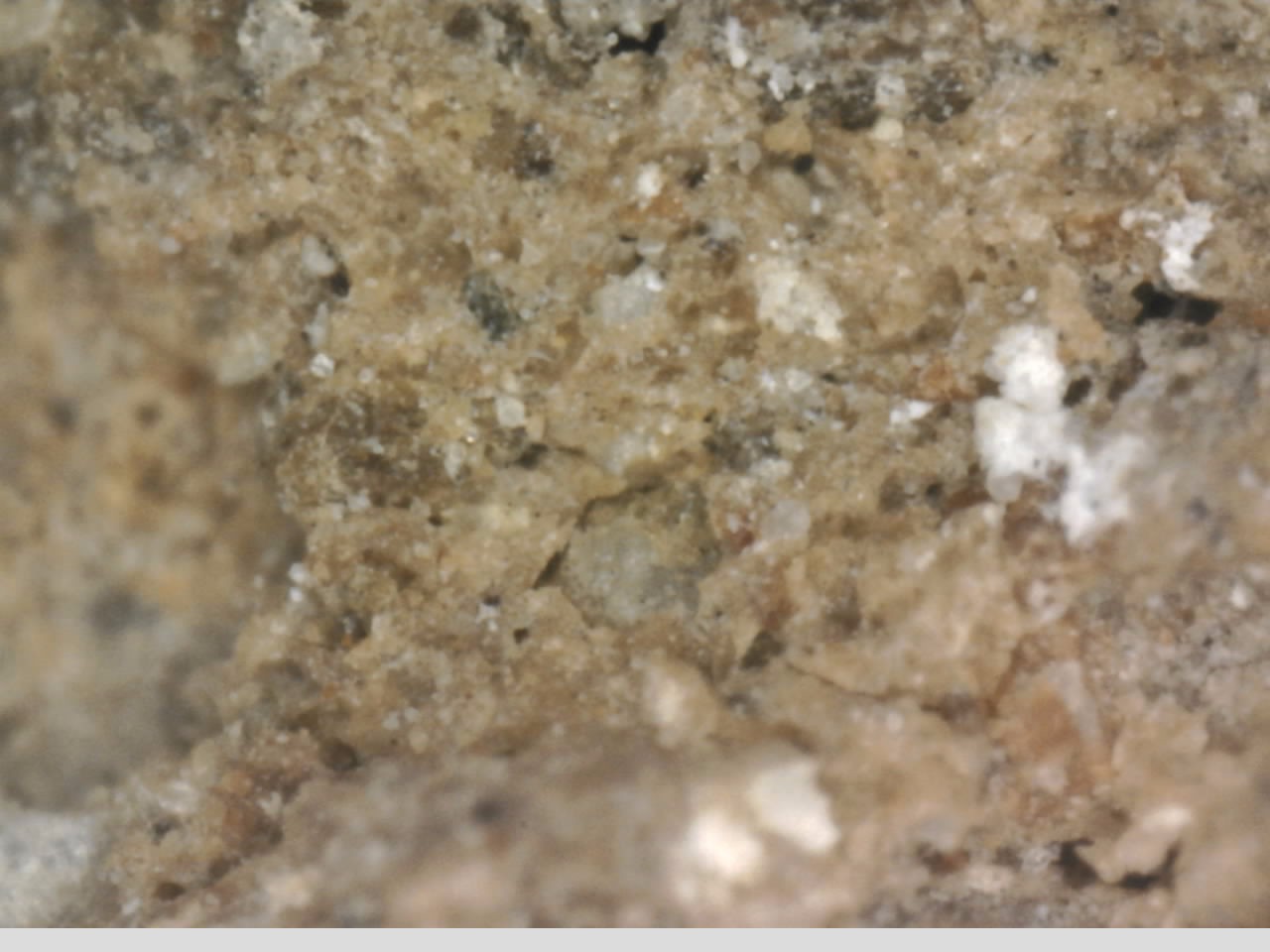

Supplement: Supplementary file 3 — Supplementary material [file mmc3.zip › Appendix A/HTN 69/HTN 69-250m-5.jpg]

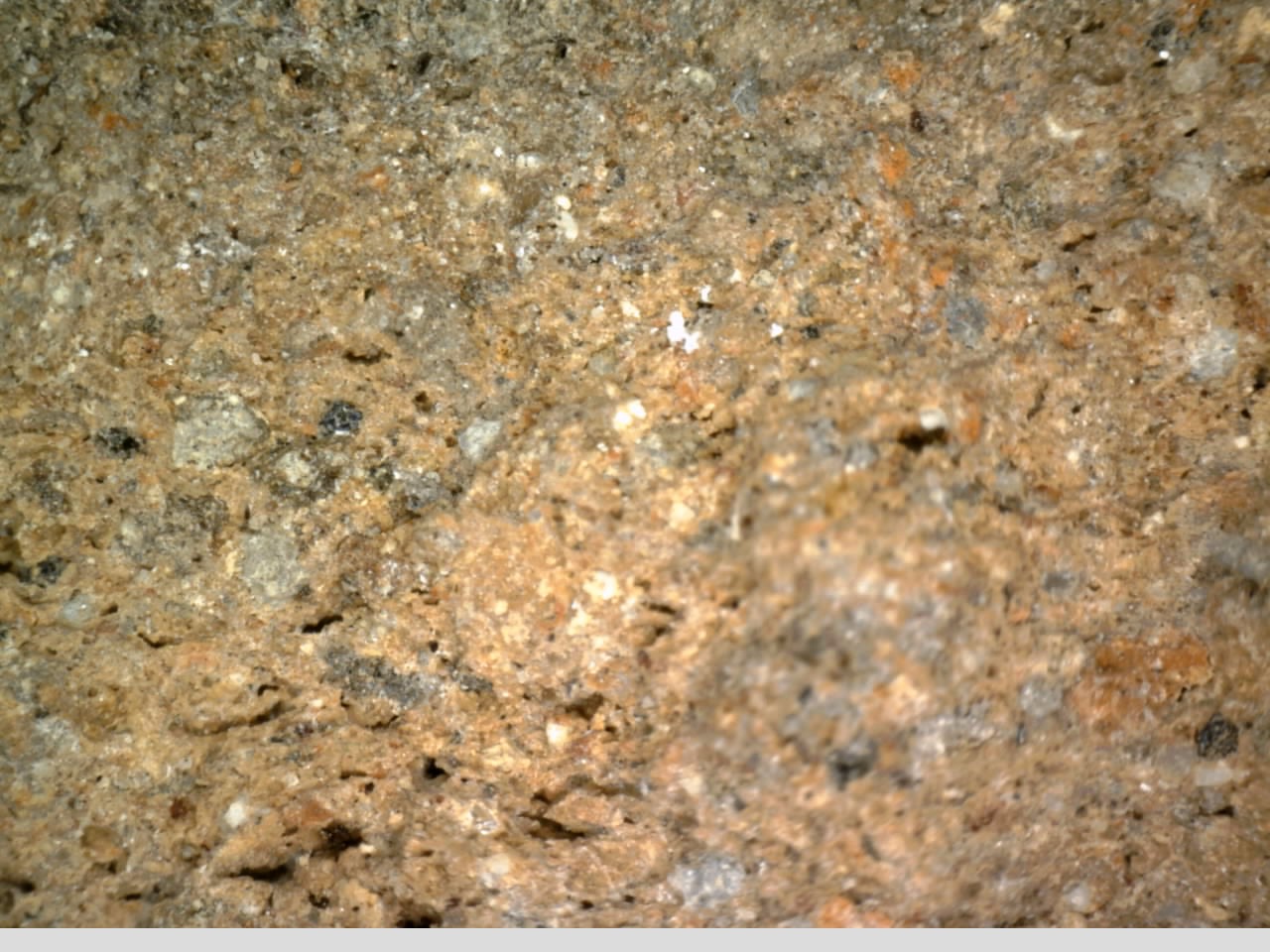

Supplement: Supplementary file 3 — Supplementary material [file mmc3.zip › Appendix A/HTN 69/HTN 69-50m-1.jpg]

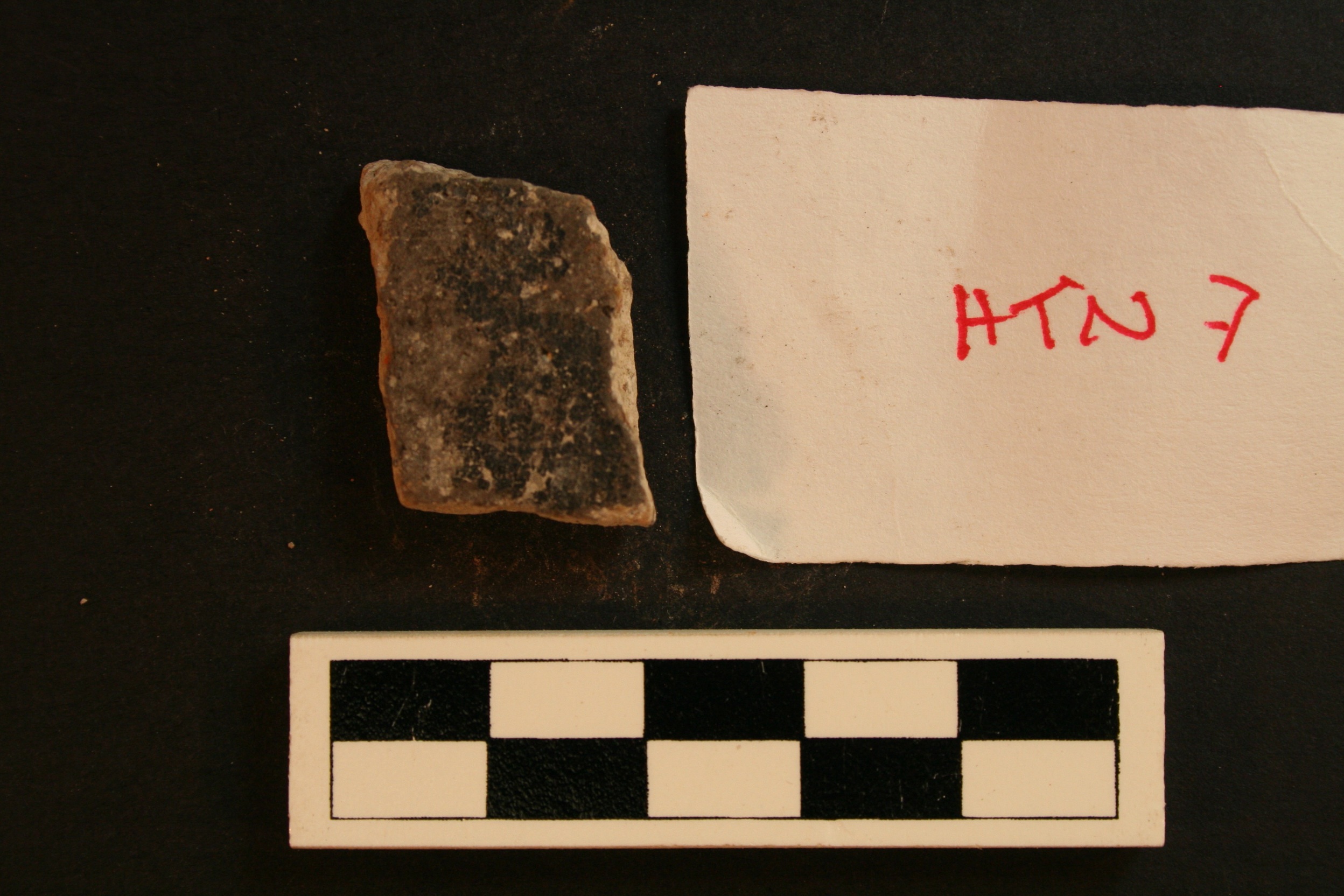

Supplement: Supplementary file 3 — Supplementary material [file mmc3.zip › Appendix A/HTN 7/7a.JPG]

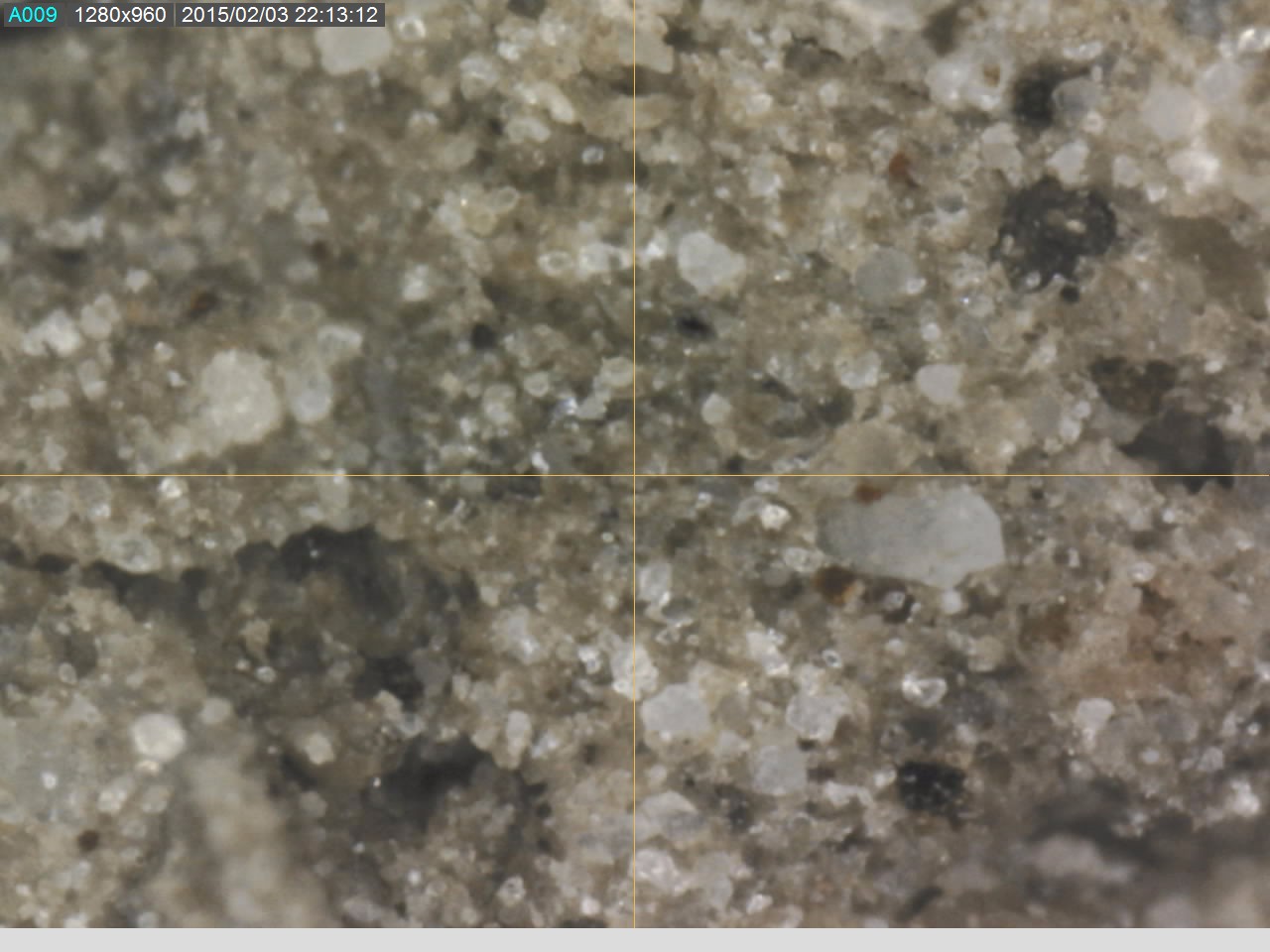

Supplement: Supplementary file 3 — Supplementary material [file mmc3.zip › Appendix A/HTN 7/HTN 7-250m-2.jpg]

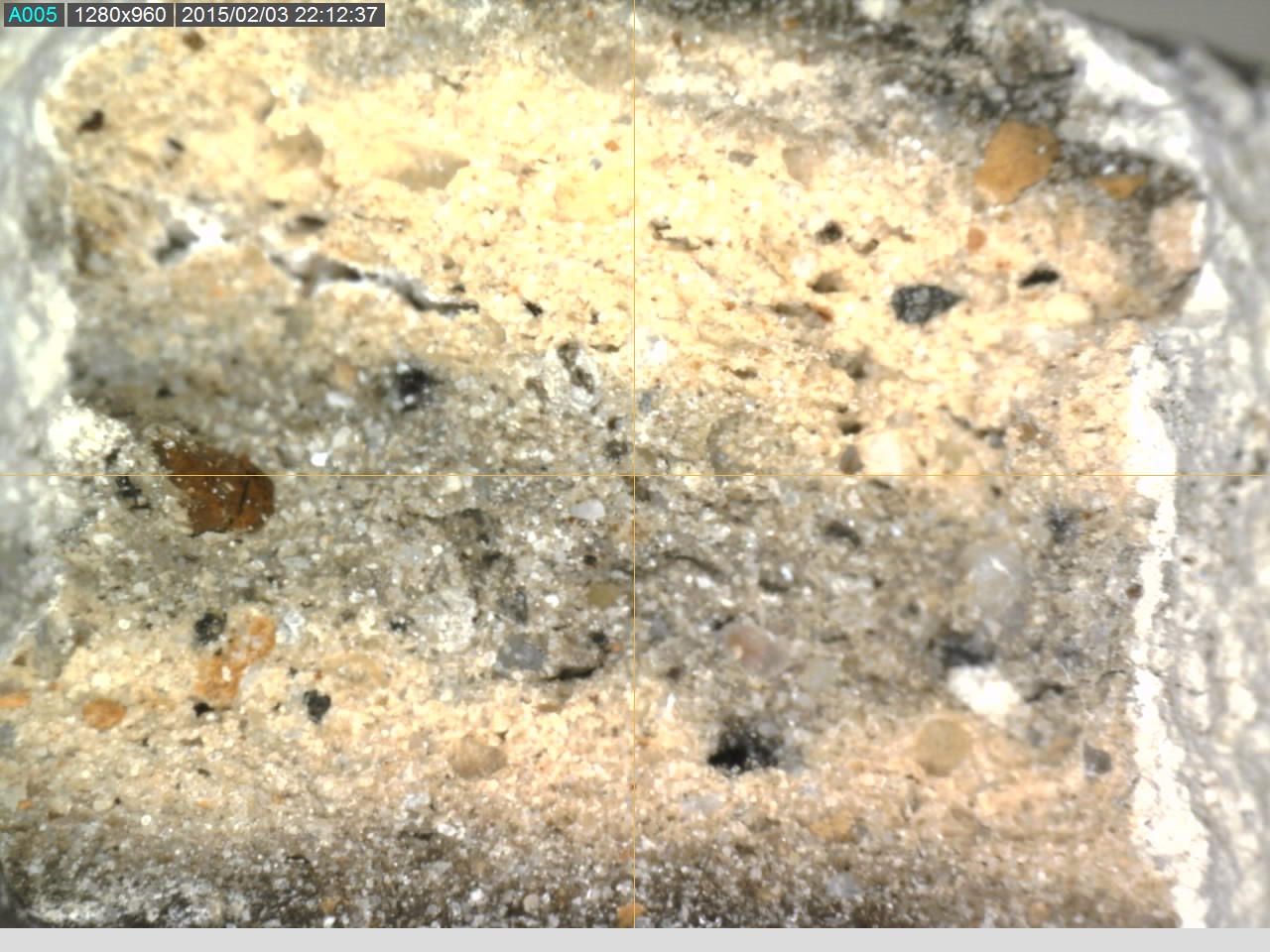

Supplement: Supplementary file 3 — Supplementary material [file mmc3.zip › Appendix A/HTN 7/HTN 7-50m-4.jpg]

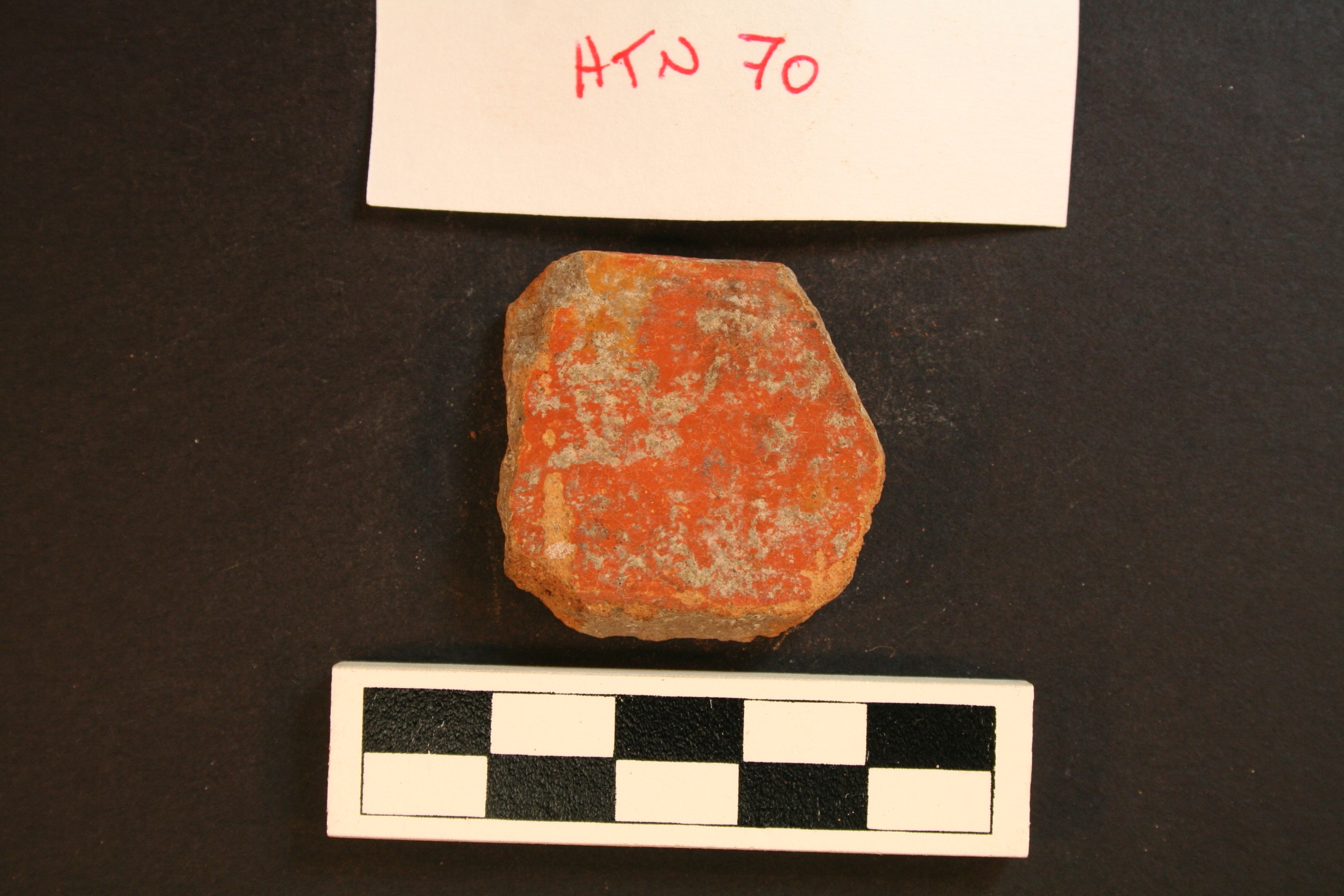

Supplement: Supplementary file 3 — Supplementary material [file mmc3.zip › Appendix A/HTN 70/70a.JPG]

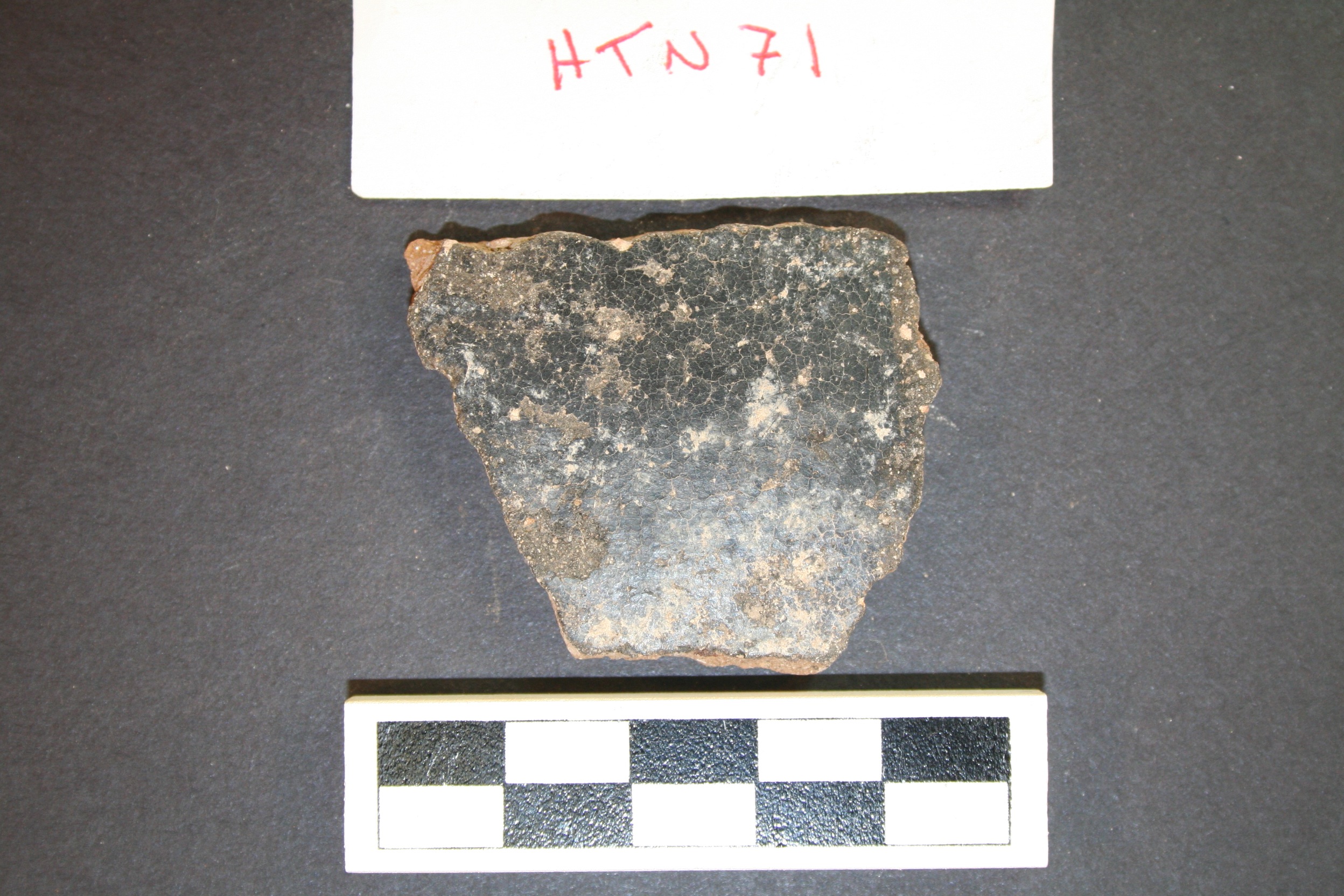

Supplement: Supplementary file 3 — Supplementary material [file mmc3.zip › Appendix A/HTN 71/71a.JPG]

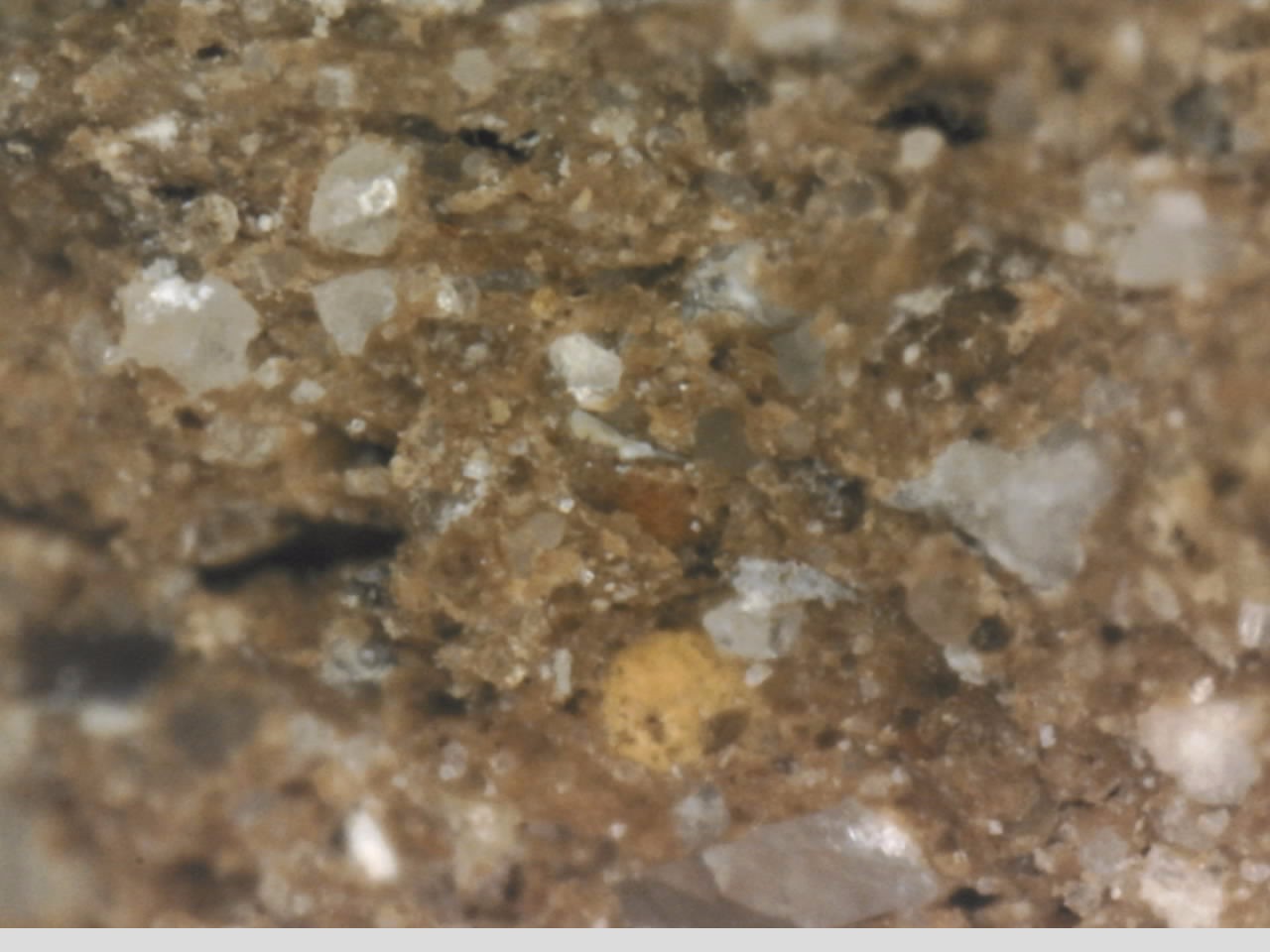

Supplement: Supplementary file 3 — Supplementary material [file mmc3.zip › Appendix A/HTN 71/HTN 71-250m-4.jpg]

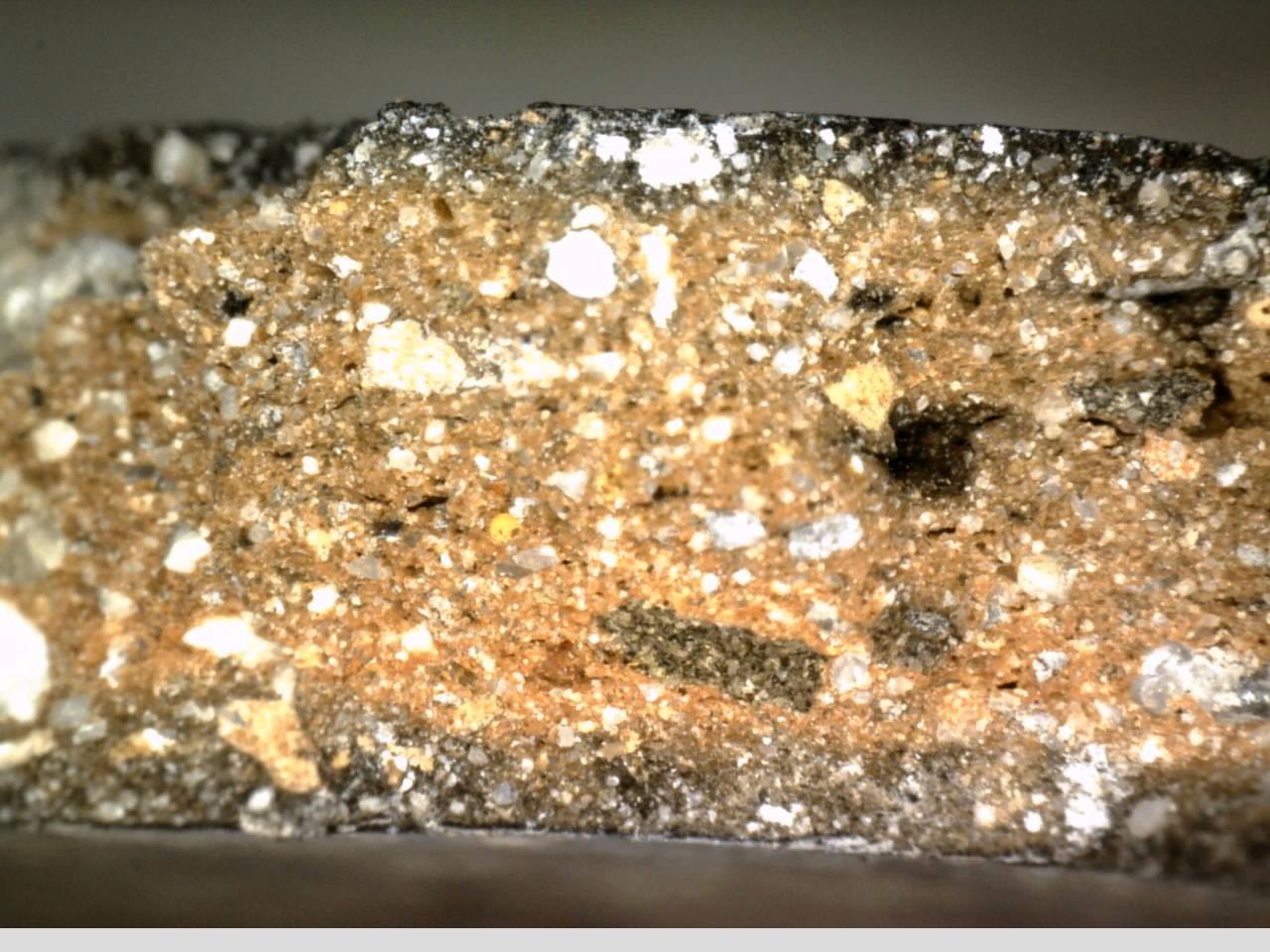

Supplement: Supplementary file 3 — Supplementary material [file mmc3.zip › Appendix A/HTN 71/HTN 71-50m-0.jpg]
