# Supplementary figures and images for: An atlas of paste fabrics and supplemental paste compositional data from late middle preclassic-period ceramics at the Maya site of Holtun, Guatemala (part 1 of 2)
Source: Data Brief. 2017 Mar 19;12:55–67. doi: 10.1016/j.dib.2017.03.024 (PMC5376253; doi:10.1016/j.dib.2017.03.024)

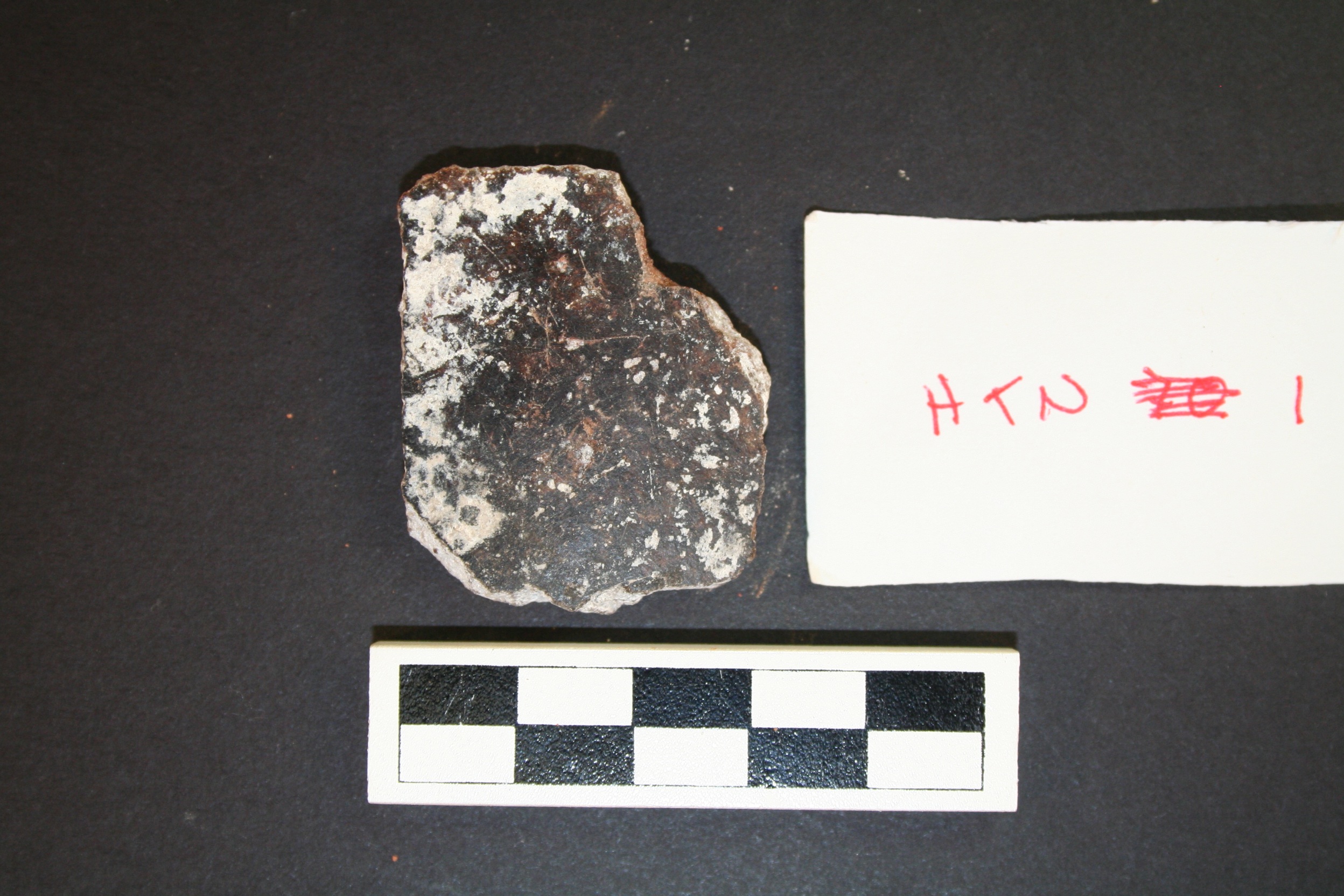

Supplement: Supplementary file 3 — Supplementary material [file mmc3.zip › Appendix A/HTN 1/1.JPG]

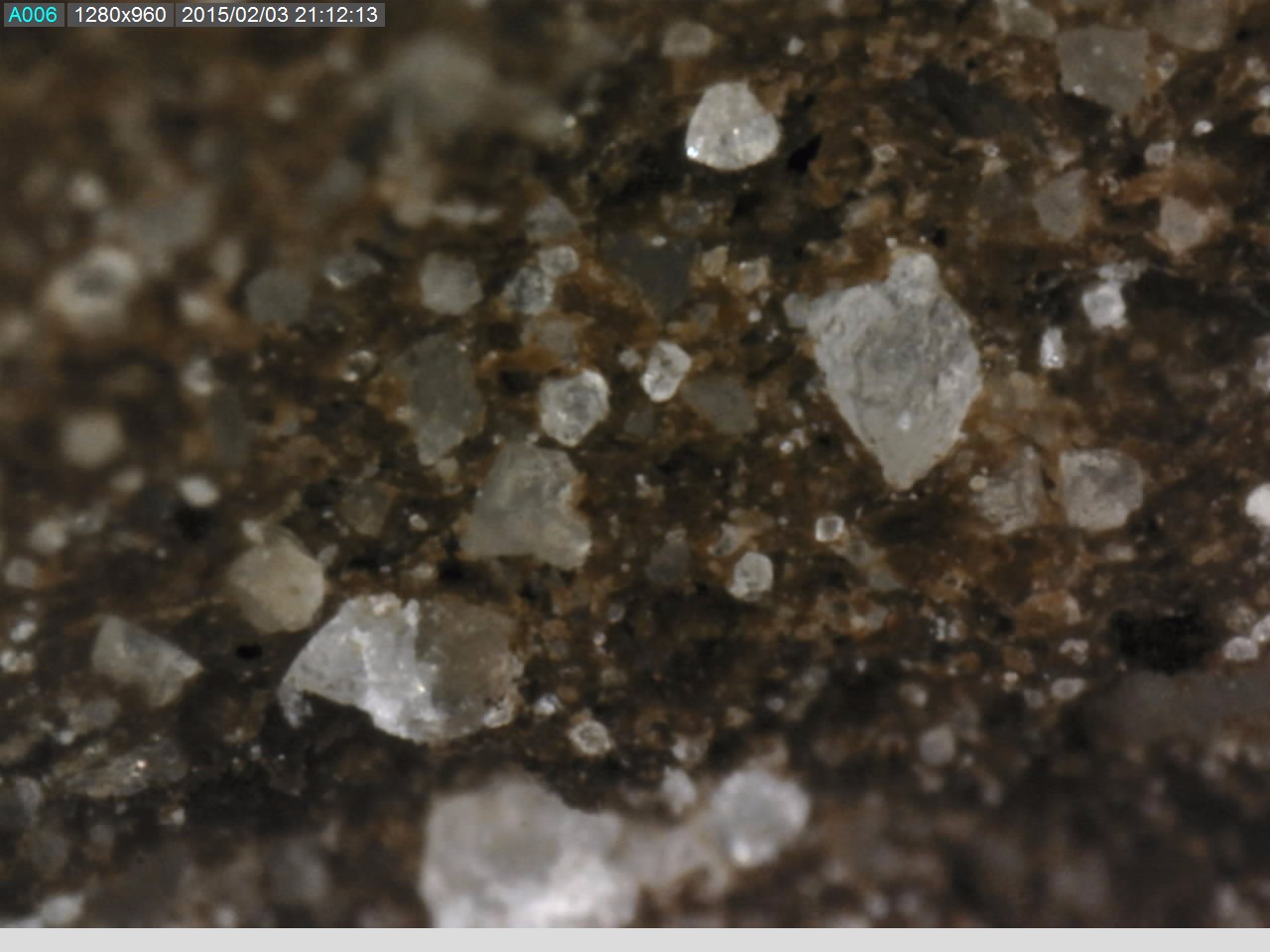

Supplement: Supplementary file 3 — Supplementary material [file mmc3.zip › Appendix A/HTN 1/HTN 1-250m-0.jpg]

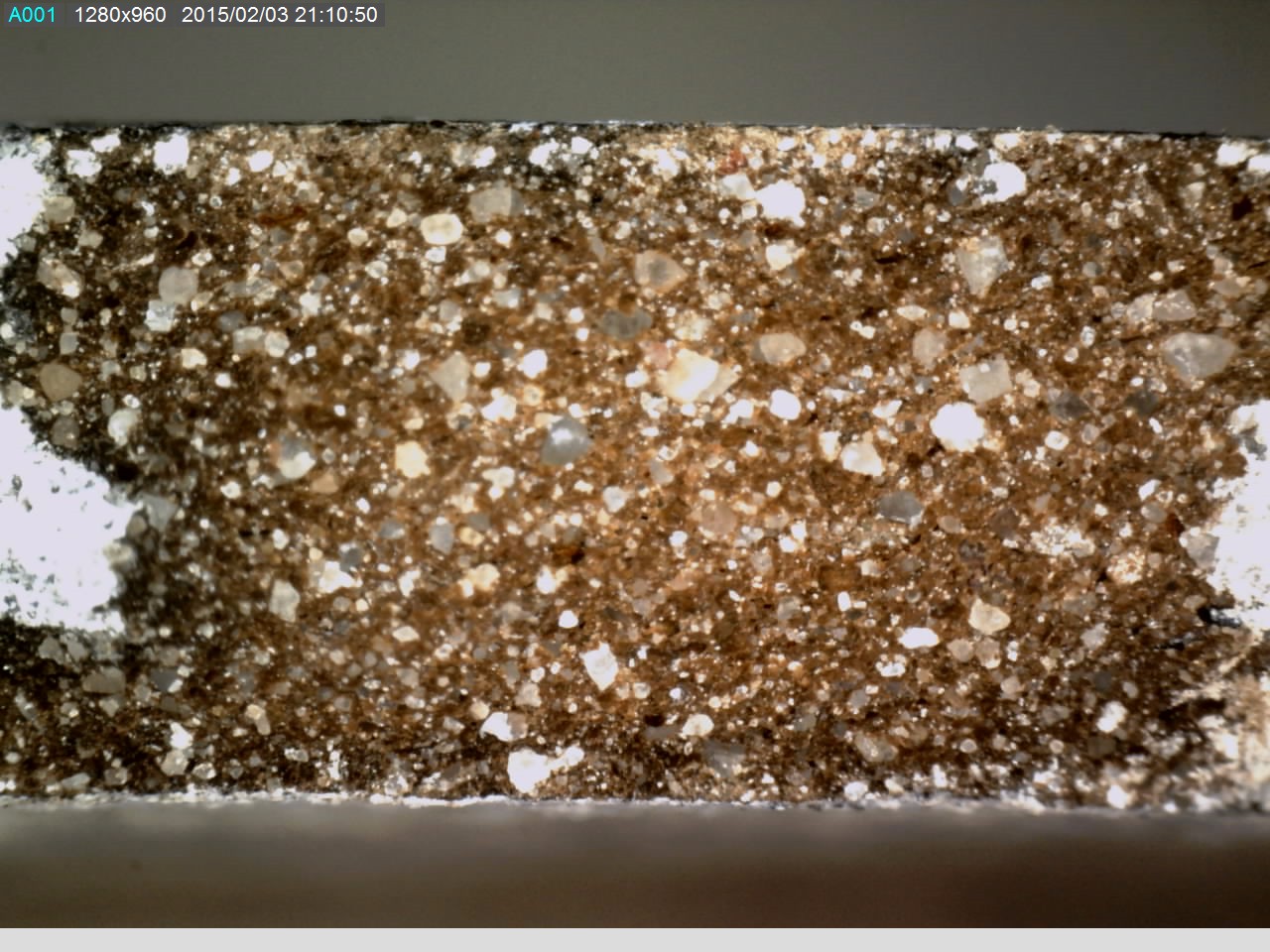

Supplement: Supplementary file 3 — Supplementary material [file mmc3.zip › Appendix A/HTN 1/HTN 1-50m-0.jpg]

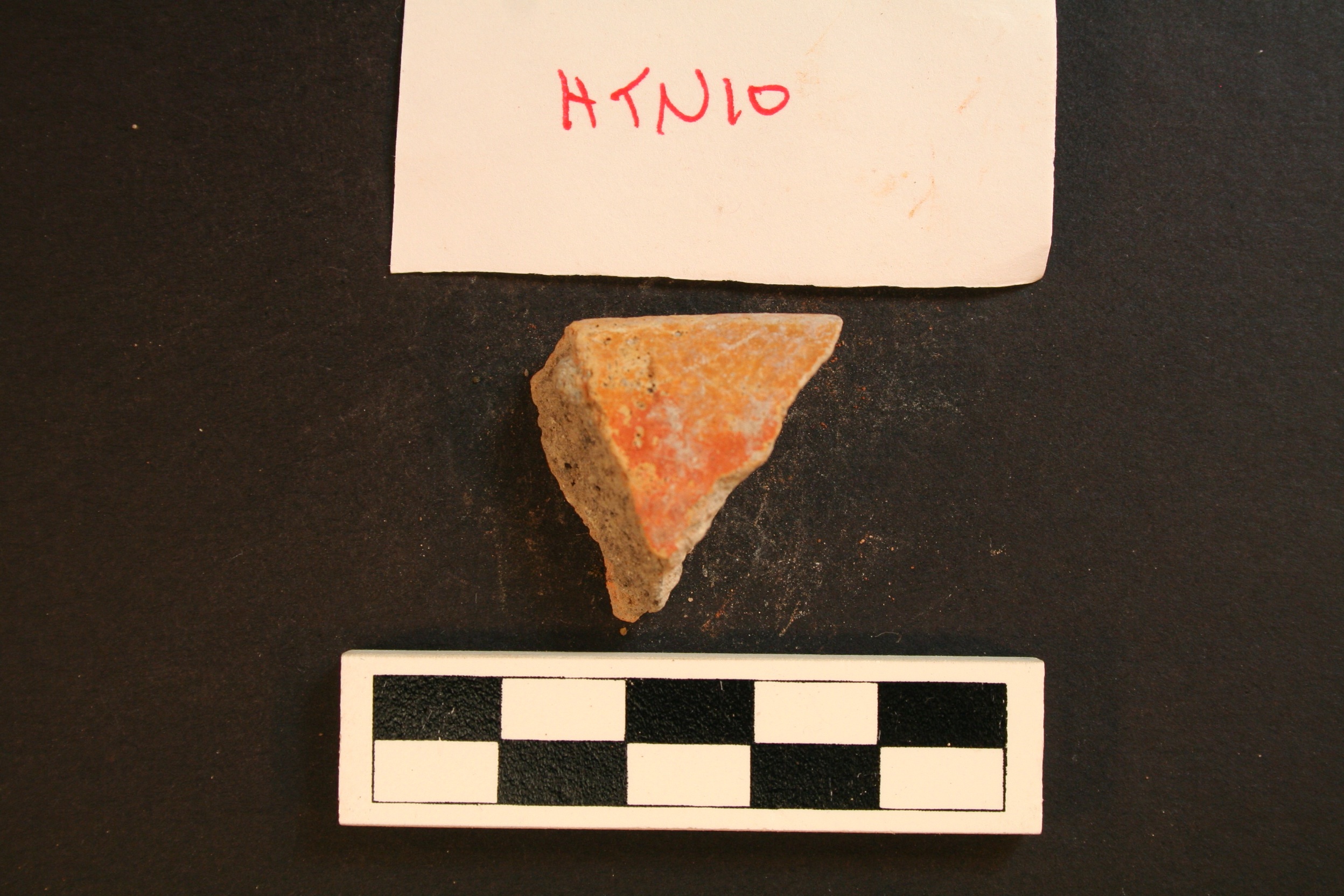

Supplement: Supplementary file 3 — Supplementary material [file mmc3.zip › Appendix A/HTN 10/10a.JPG]

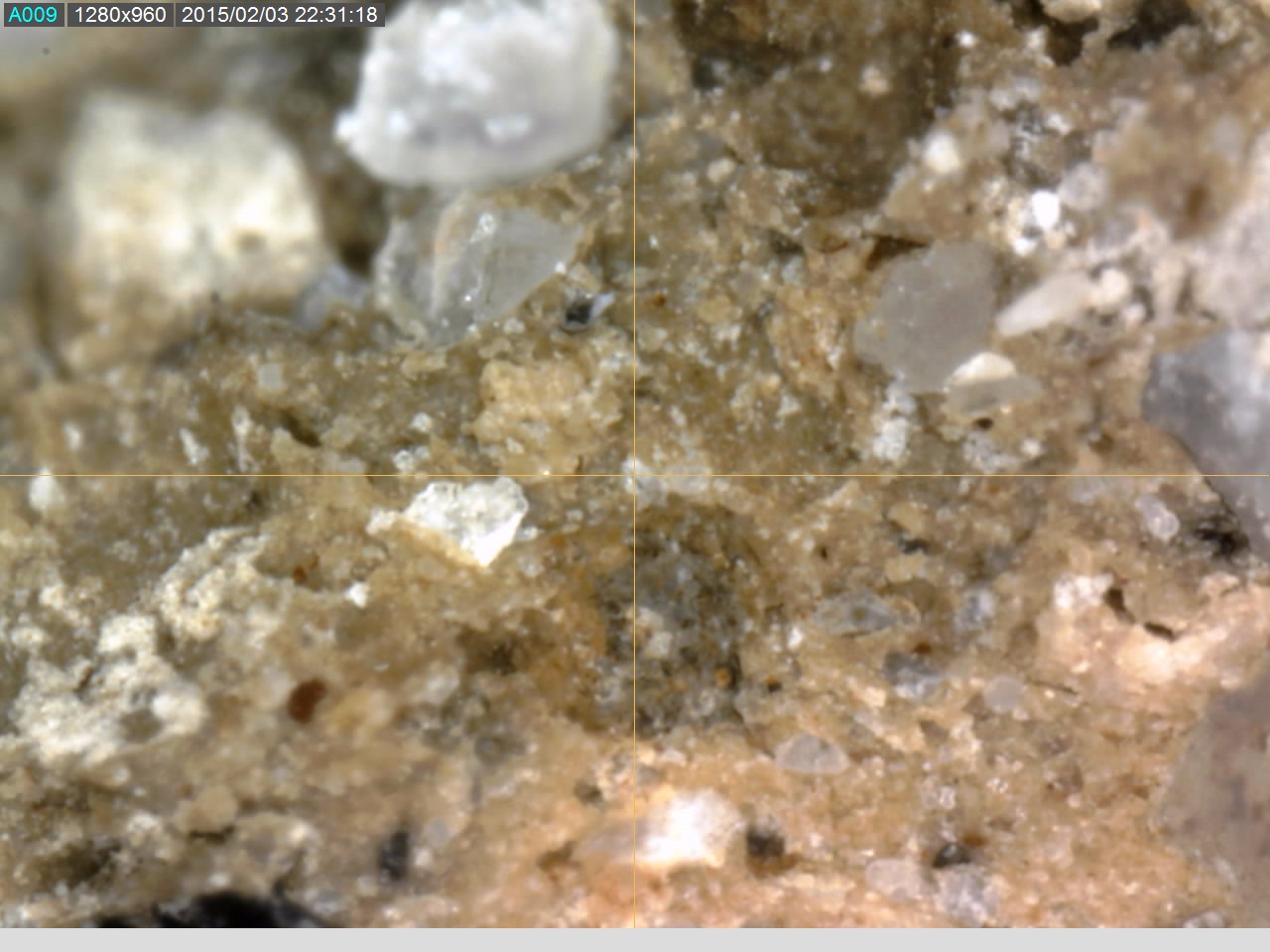

Supplement: Supplementary file 3 — Supplementary material [file mmc3.zip › Appendix A/HTN 10/HTN 10-250m-5.jpg]

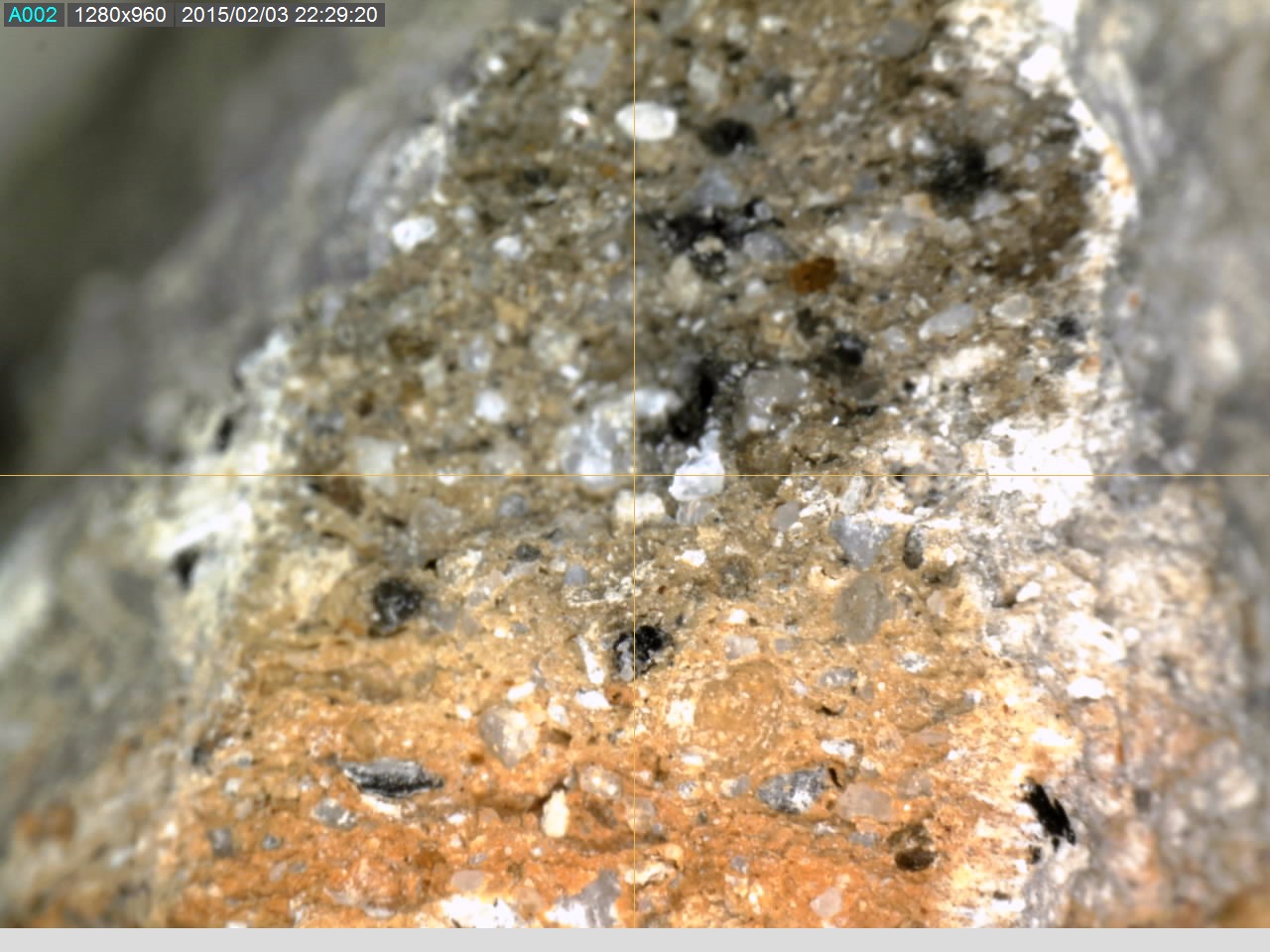

Supplement: Supplementary file 3 — Supplementary material [file mmc3.zip › Appendix A/HTN 10/HTN 10-50m-1.jpg]

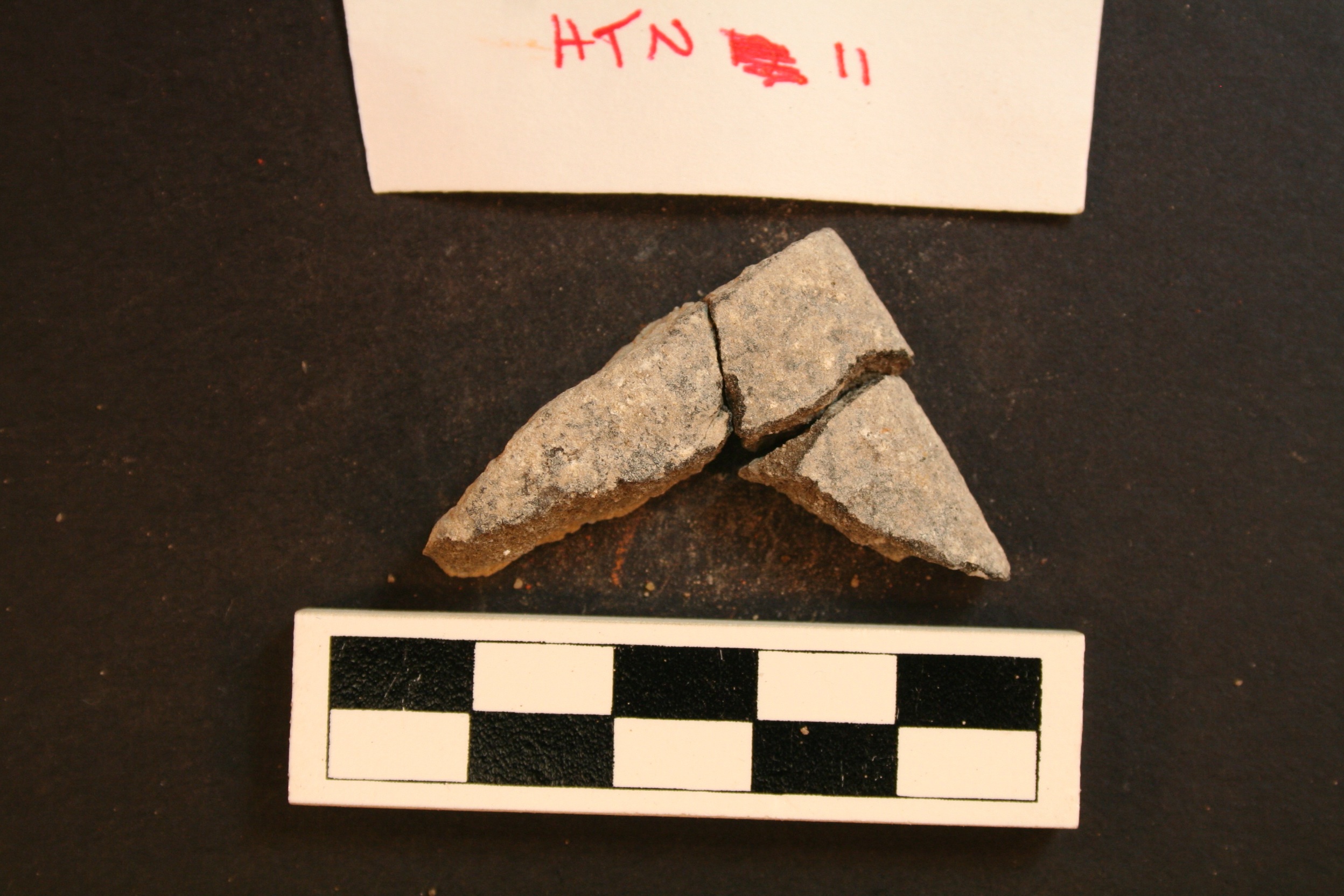

Supplement: Supplementary file 3 — Supplementary material [file mmc3.zip › Appendix A/HTN 11/11a.JPG]

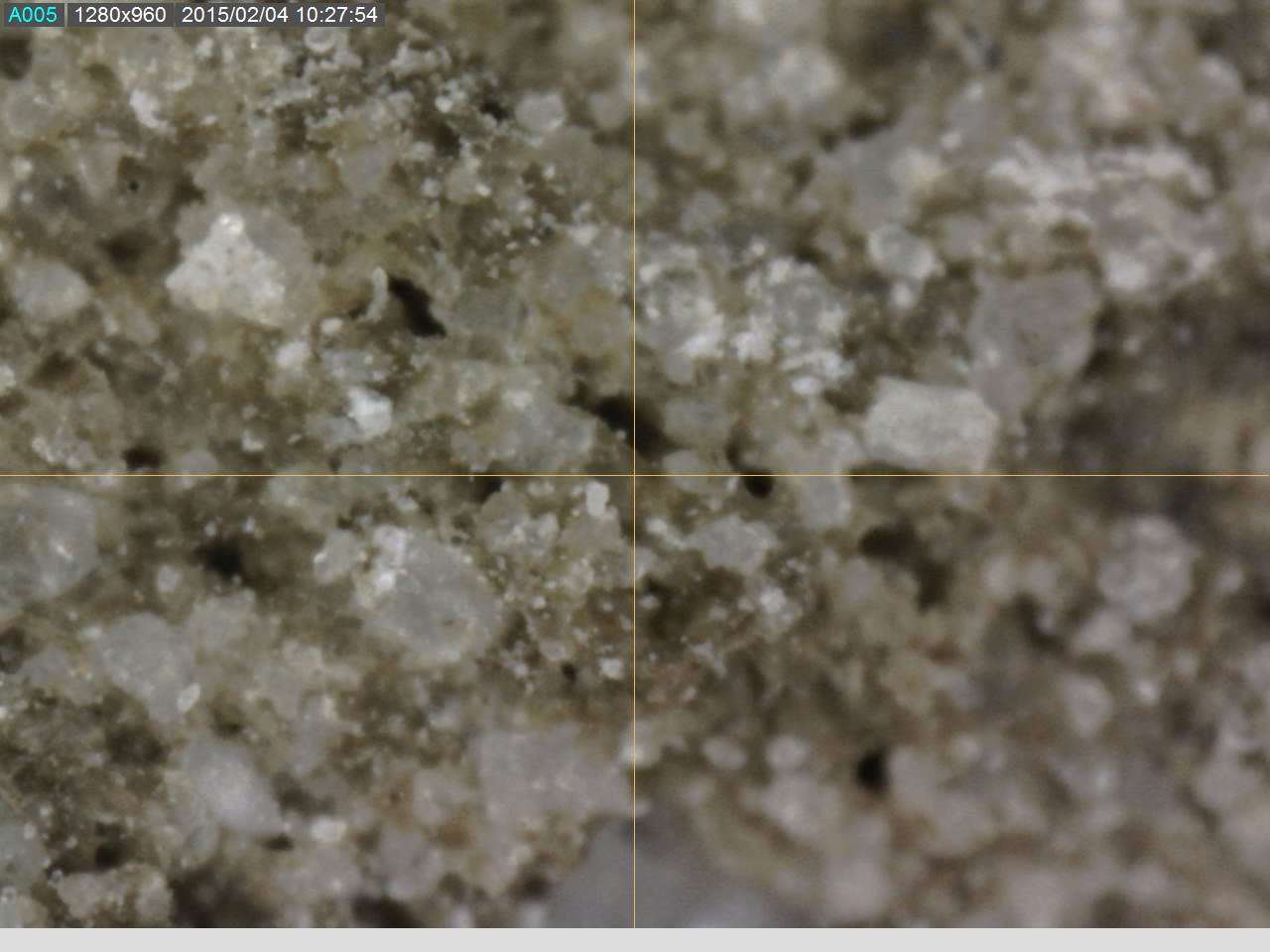

Supplement: Supplementary file 3 — Supplementary material [file mmc3.zip › Appendix A/HTN 11/HTN 11-250m-0.jpg]

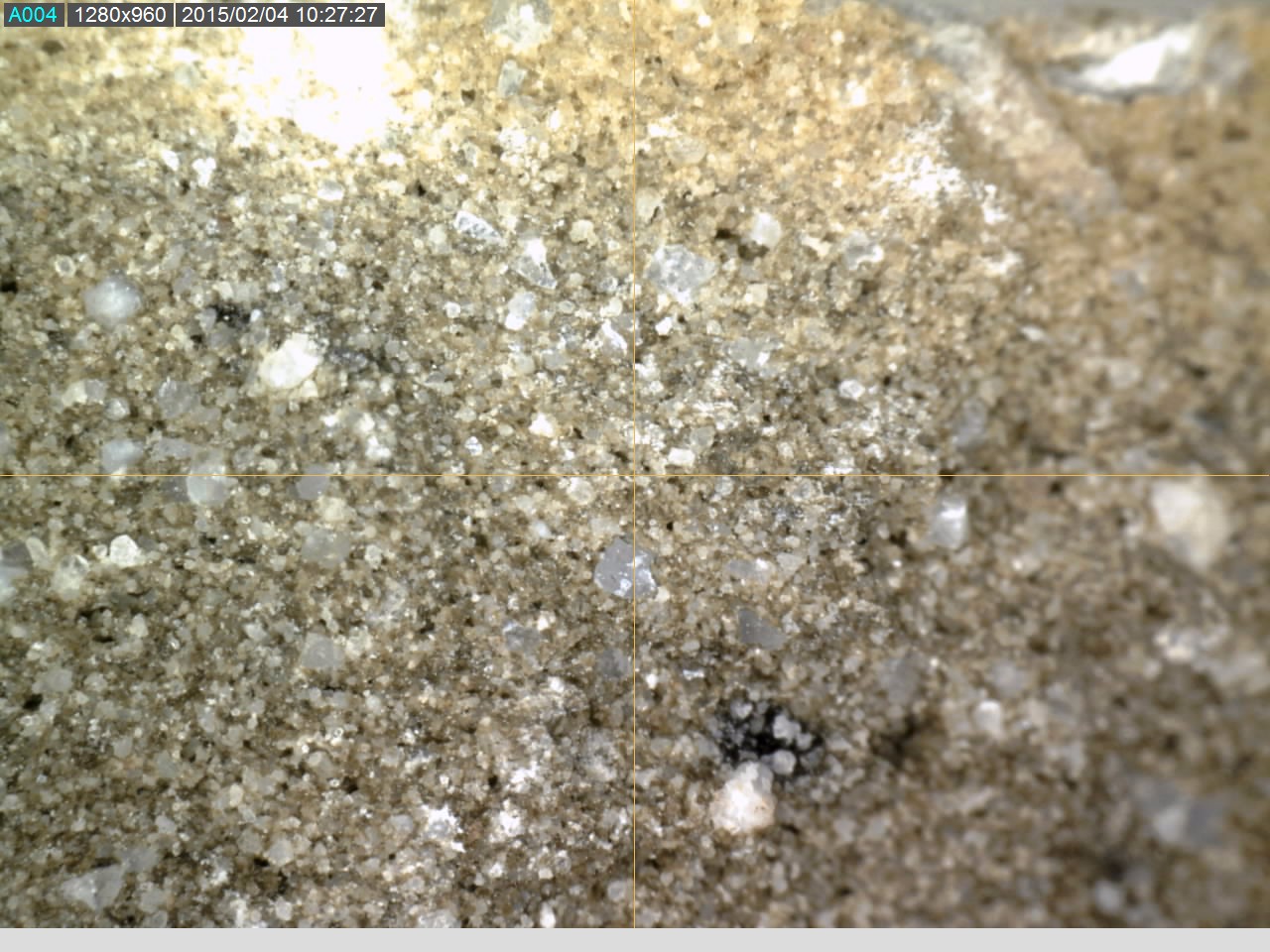

Supplement: Supplementary file 3 — Supplementary material [file mmc3.zip › Appendix A/HTN 11/HTN 11-50m-3.jpg]

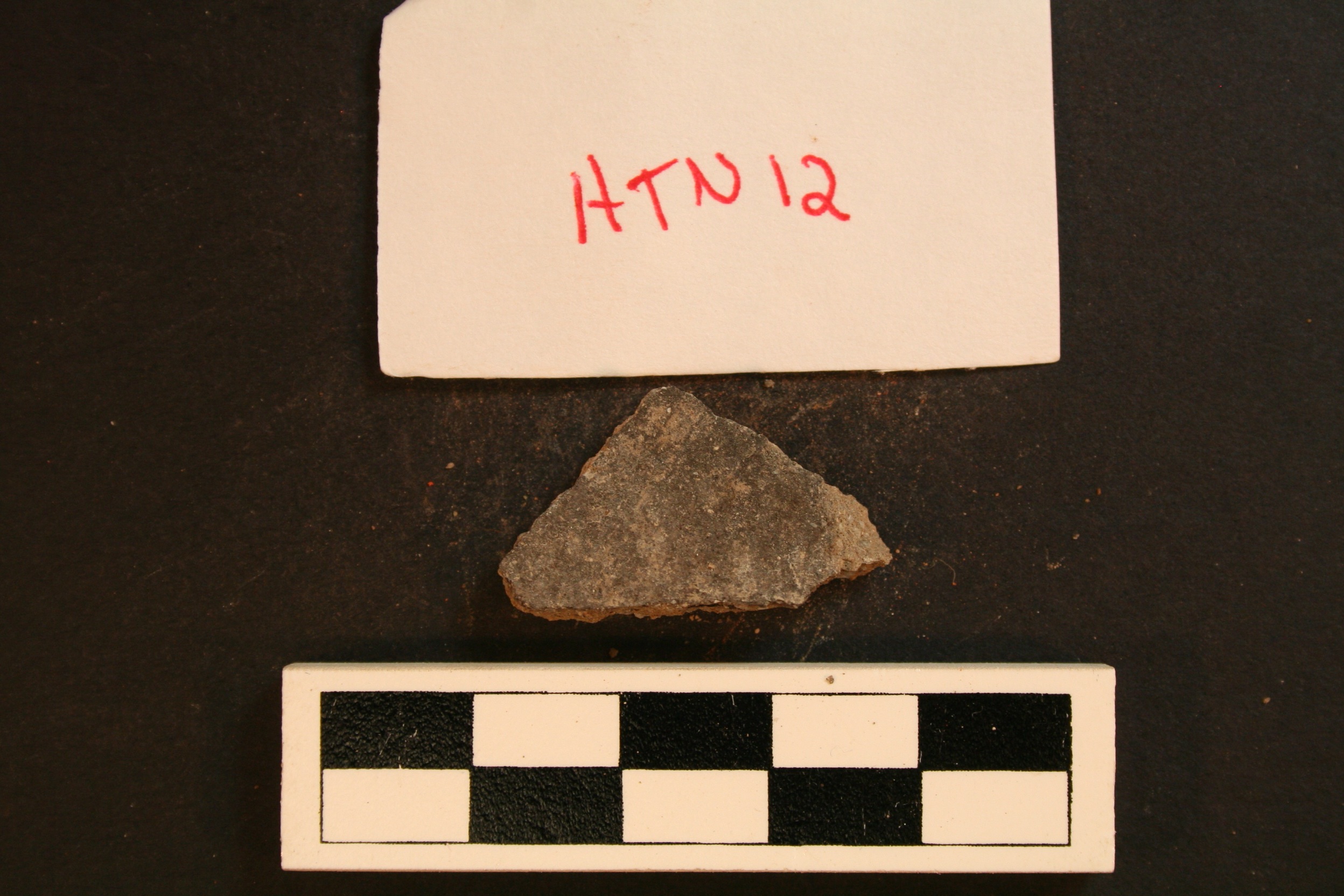

Supplement: Supplementary file 3 — Supplementary material [file mmc3.zip › Appendix A/HTN 12/12a.JPG]

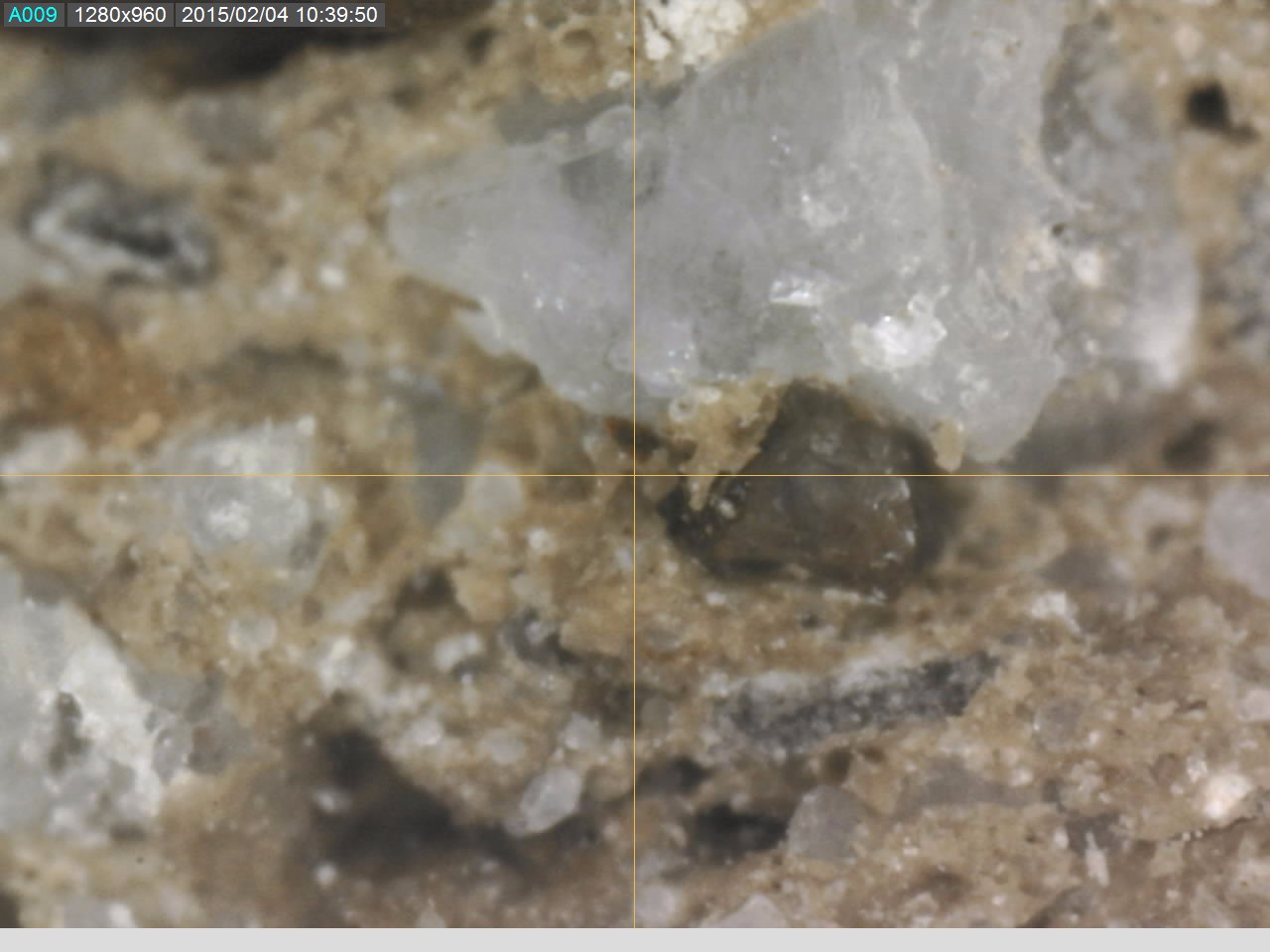

Supplement: Supplementary file 3 — Supplementary material [file mmc3.zip › Appendix A/HTN 12/HTN 12-250m-2.jpg]

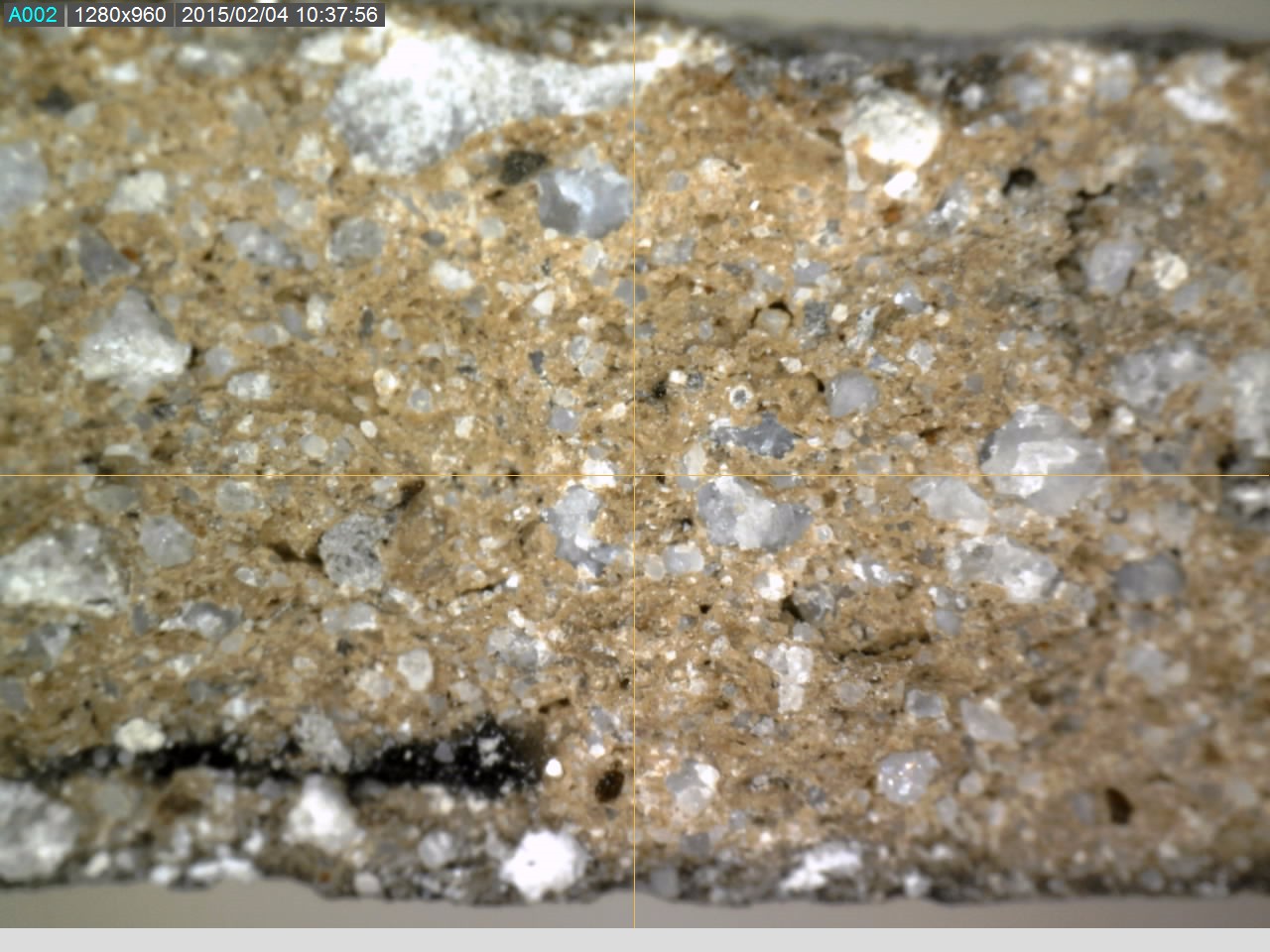

Supplement: Supplementary file 3 — Supplementary material [file mmc3.zip › Appendix A/HTN 12/HTN 12-50m-1.jpg]

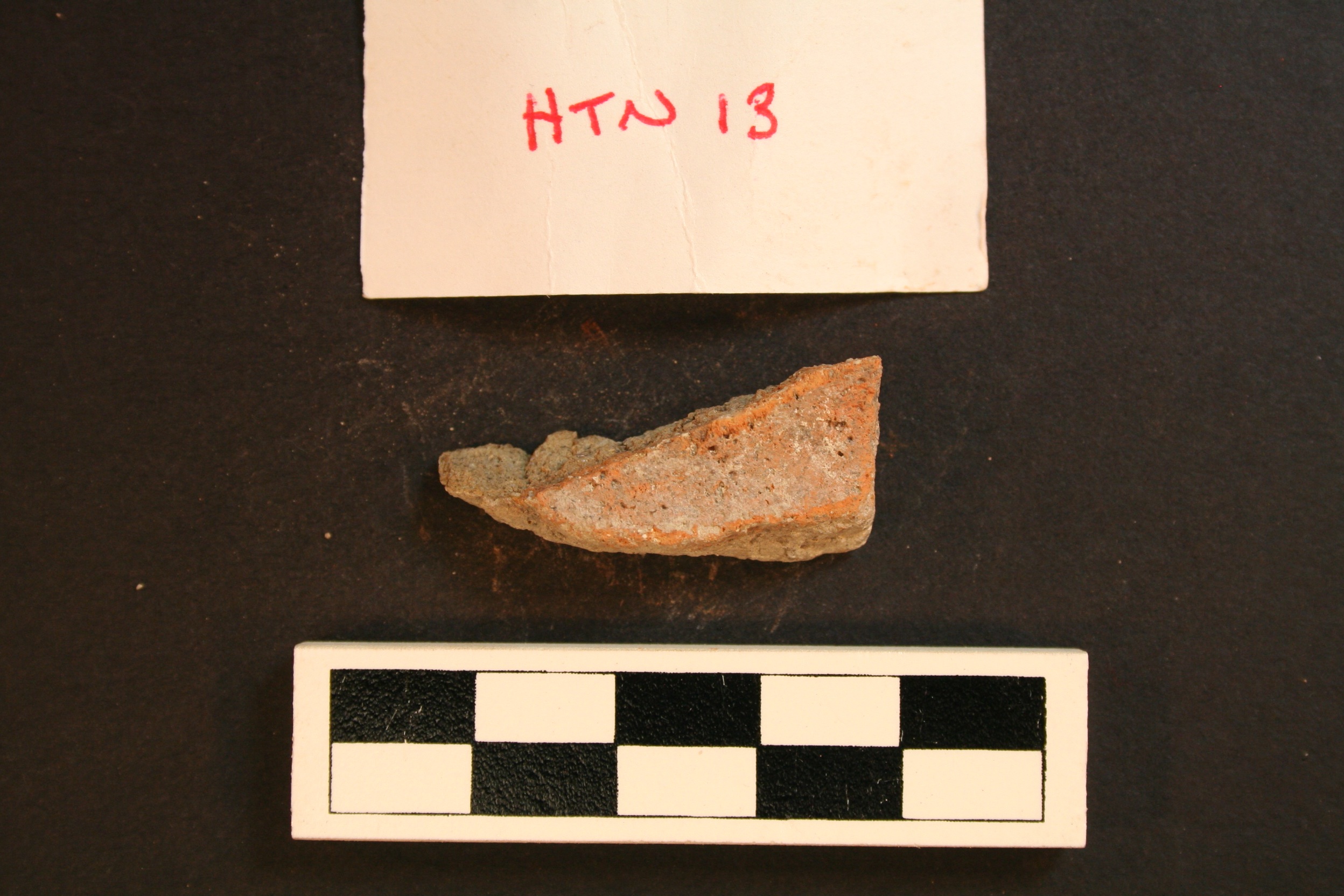

Supplement: Supplementary file 3 — Supplementary material [file mmc3.zip › Appendix A/HTN 13/13a.JPG]

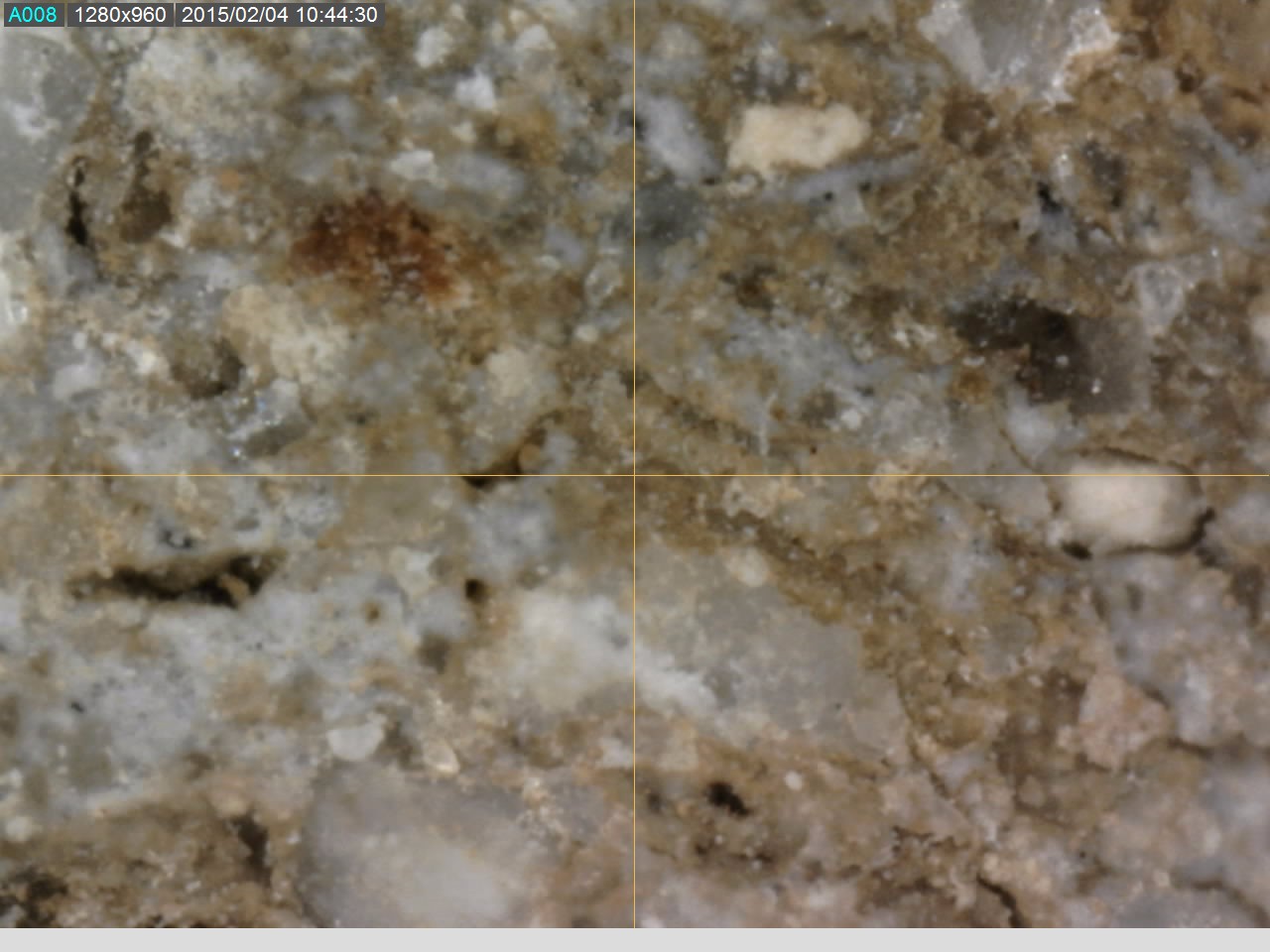

Supplement: Supplementary file 3 — Supplementary material [file mmc3.zip › Appendix A/HTN 13/HTN 13-250m-0.jpg]

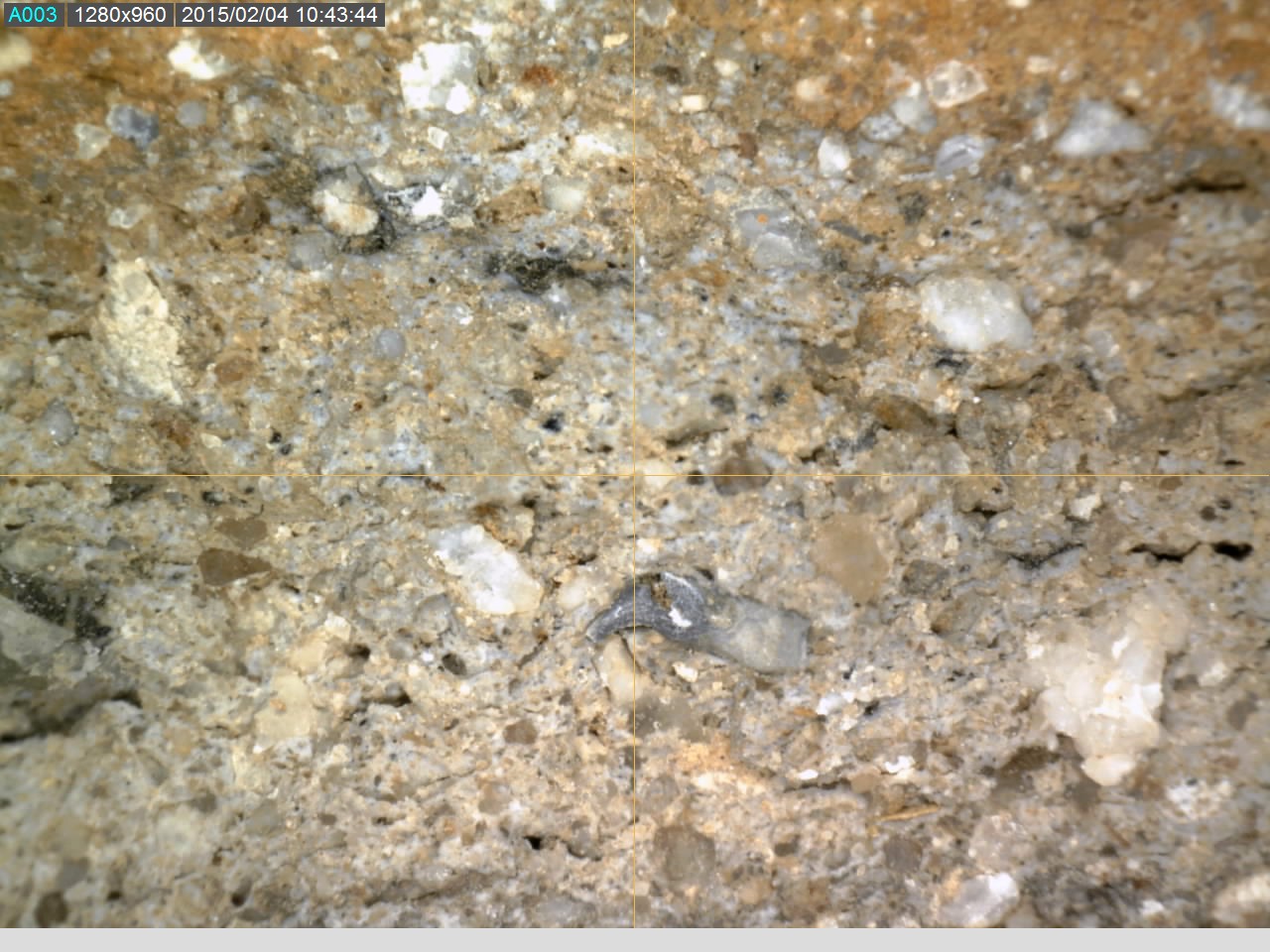

Supplement: Supplementary file 3 — Supplementary material [file mmc3.zip › Appendix A/HTN 13/HTN 13-50m-2.jpg]

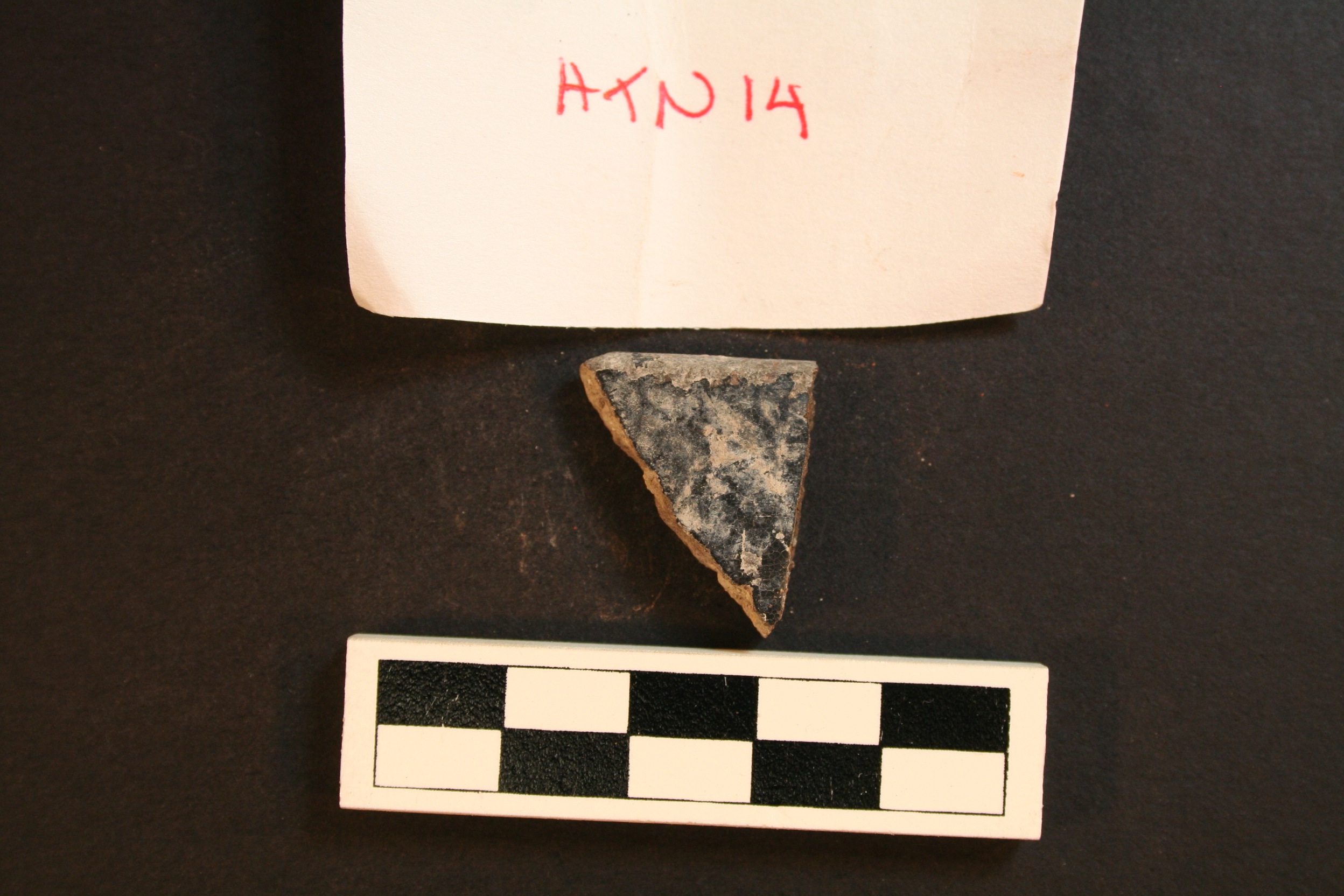

Supplement: Supplementary file 3 — Supplementary material [file mmc3.zip › Appendix A/HTN 14/14a.JPG]

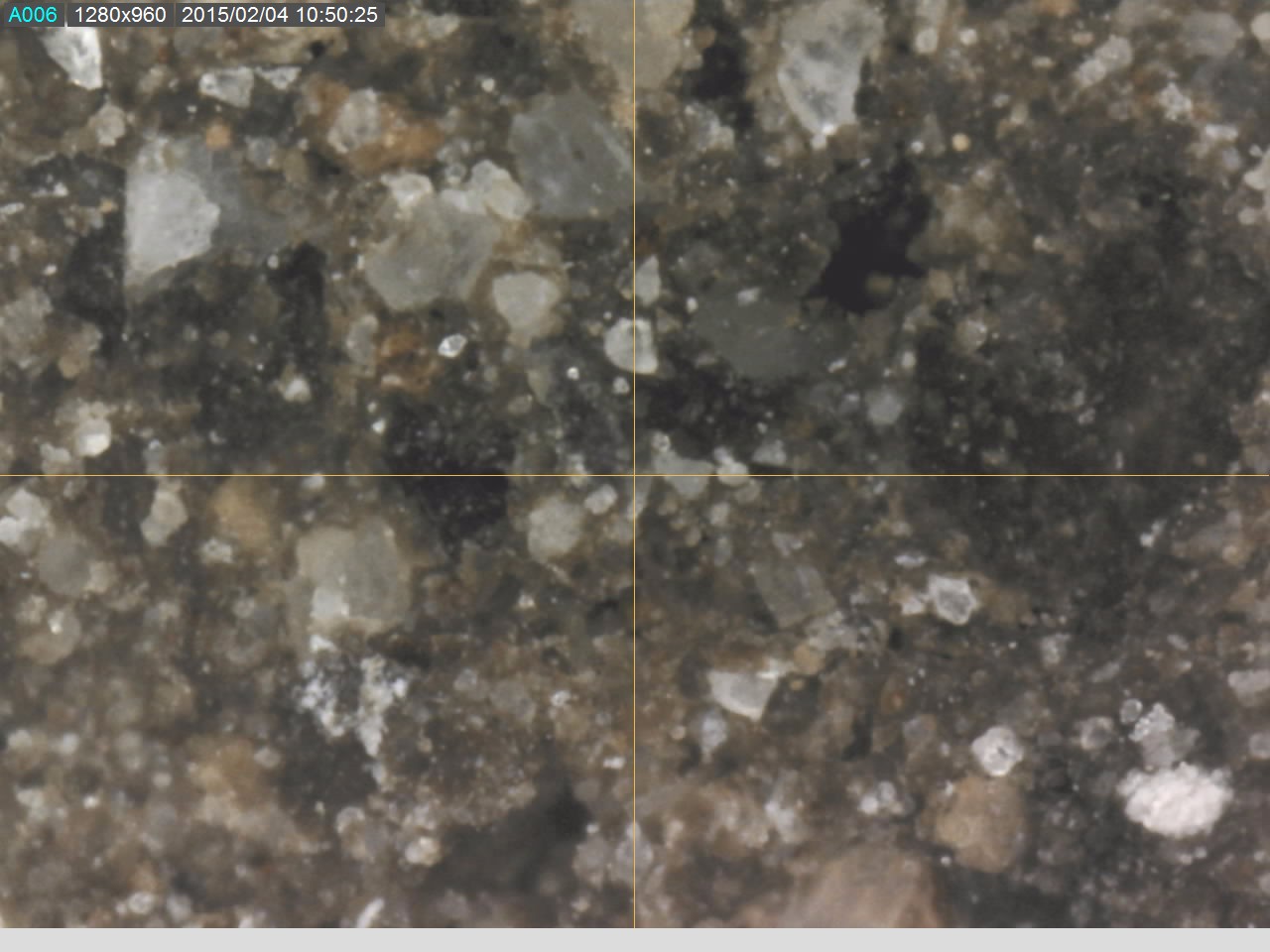

Supplement: Supplementary file 3 — Supplementary material [file mmc3.zip › Appendix A/HTN 14/HTN 14-250m-0.jpg]

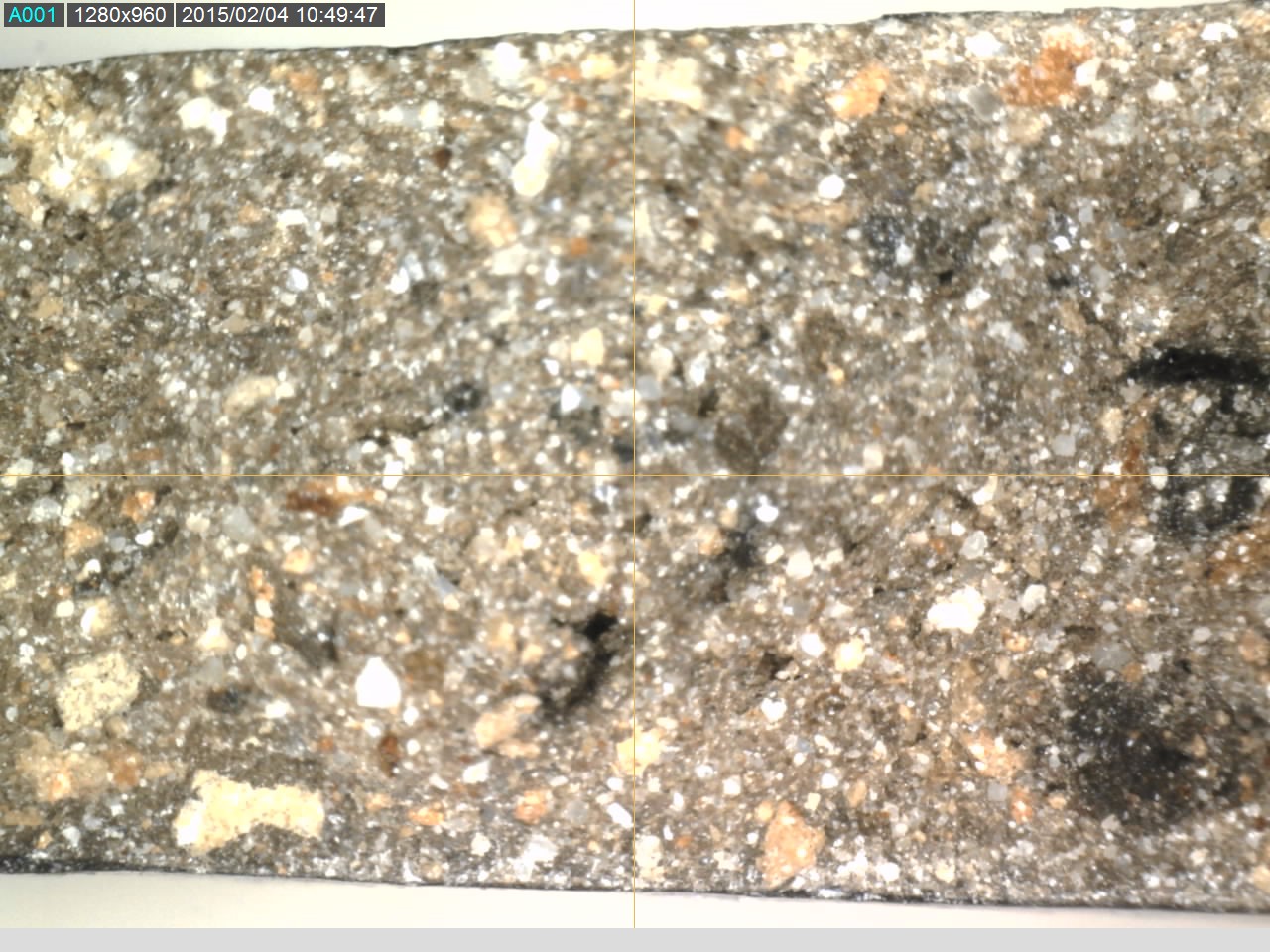

Supplement: Supplementary file 3 — Supplementary material [file mmc3.zip › Appendix A/HTN 14/HTN 14-50m-0.jpg]

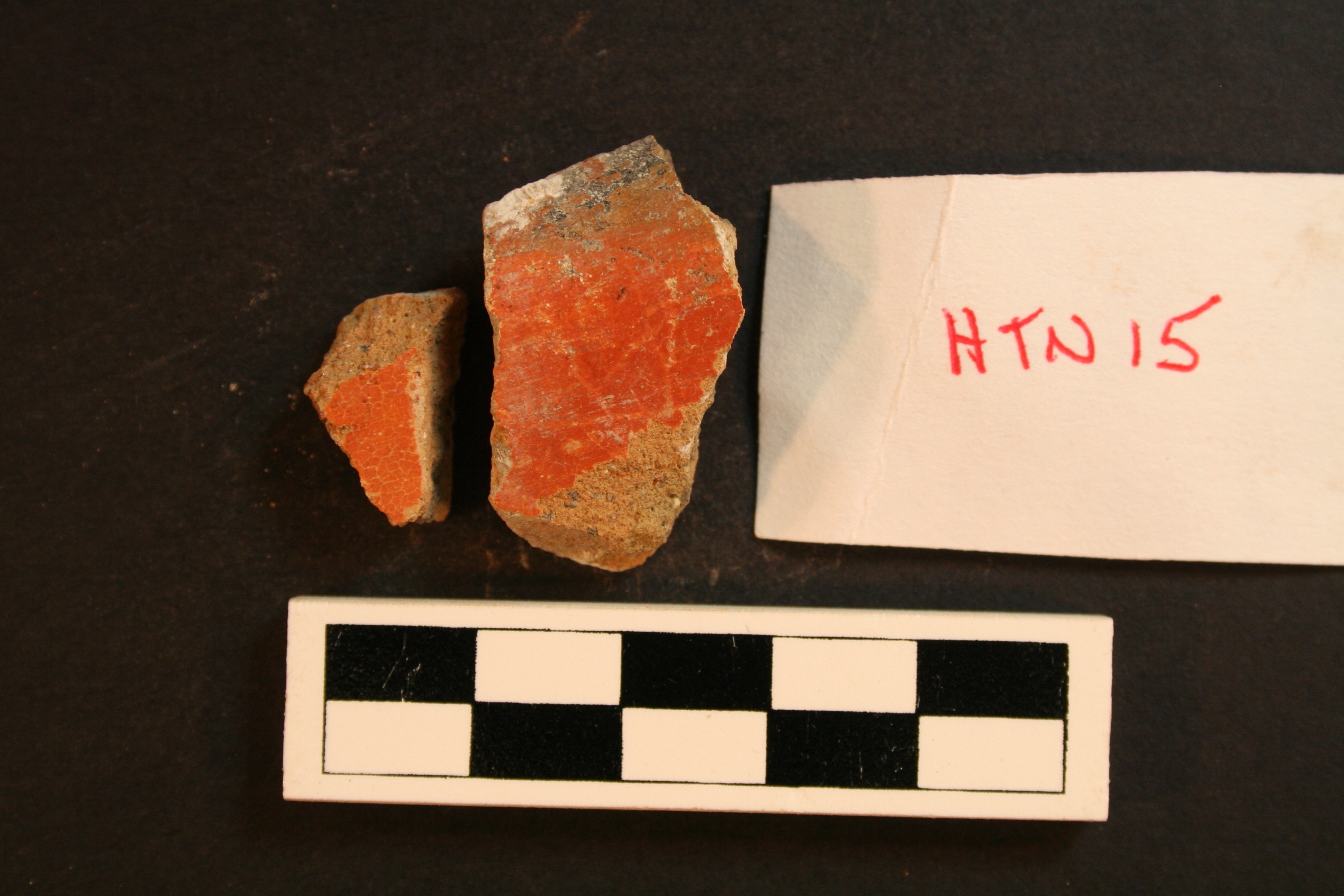

Supplement: Supplementary file 3 — Supplementary material [file mmc3.zip › Appendix A/HTN 15/15a.JPG]

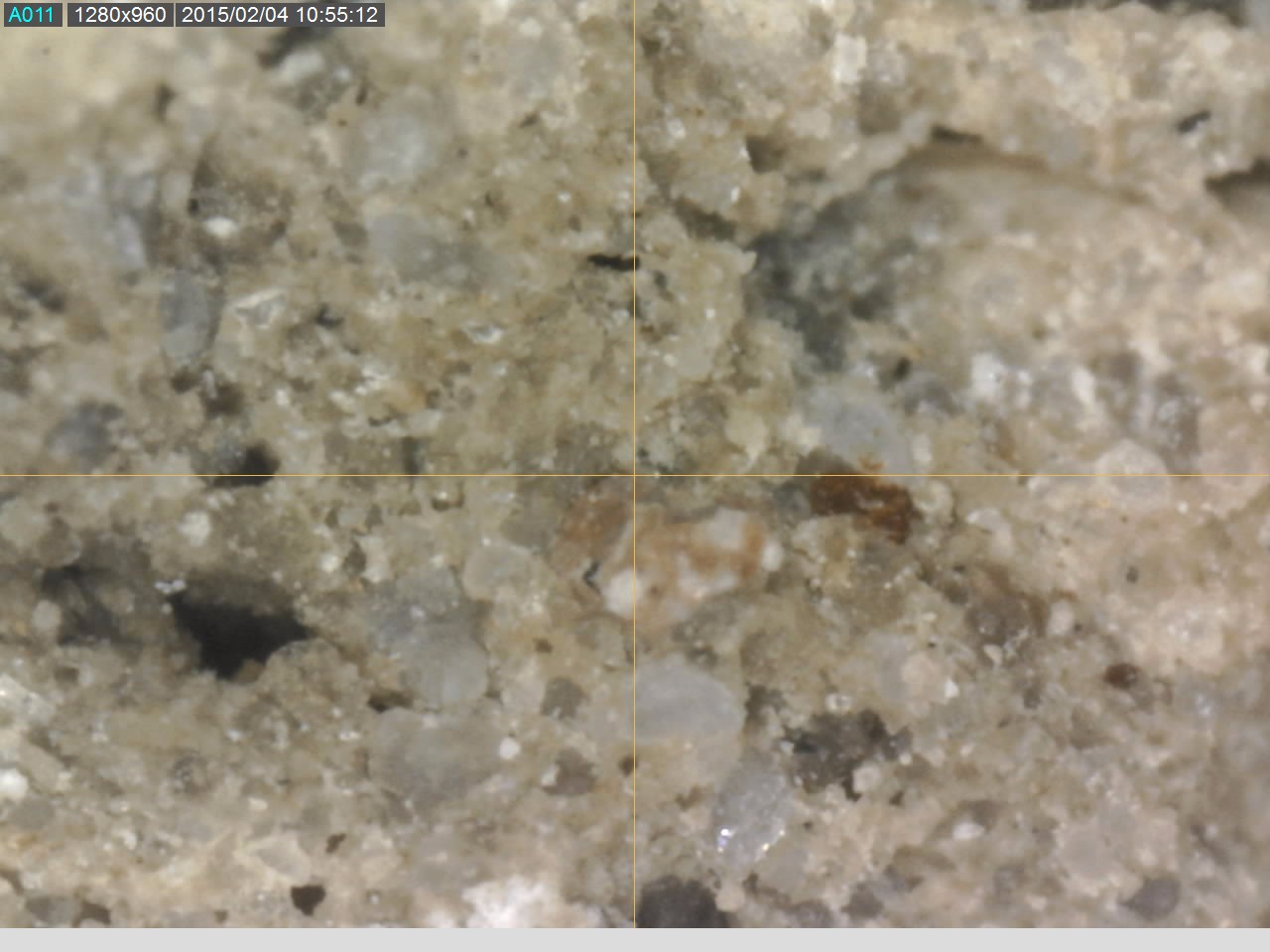

Supplement: Supplementary file 3 — Supplementary material [file mmc3.zip › Appendix A/HTN 15/HTN 15-250m-2.jpg]

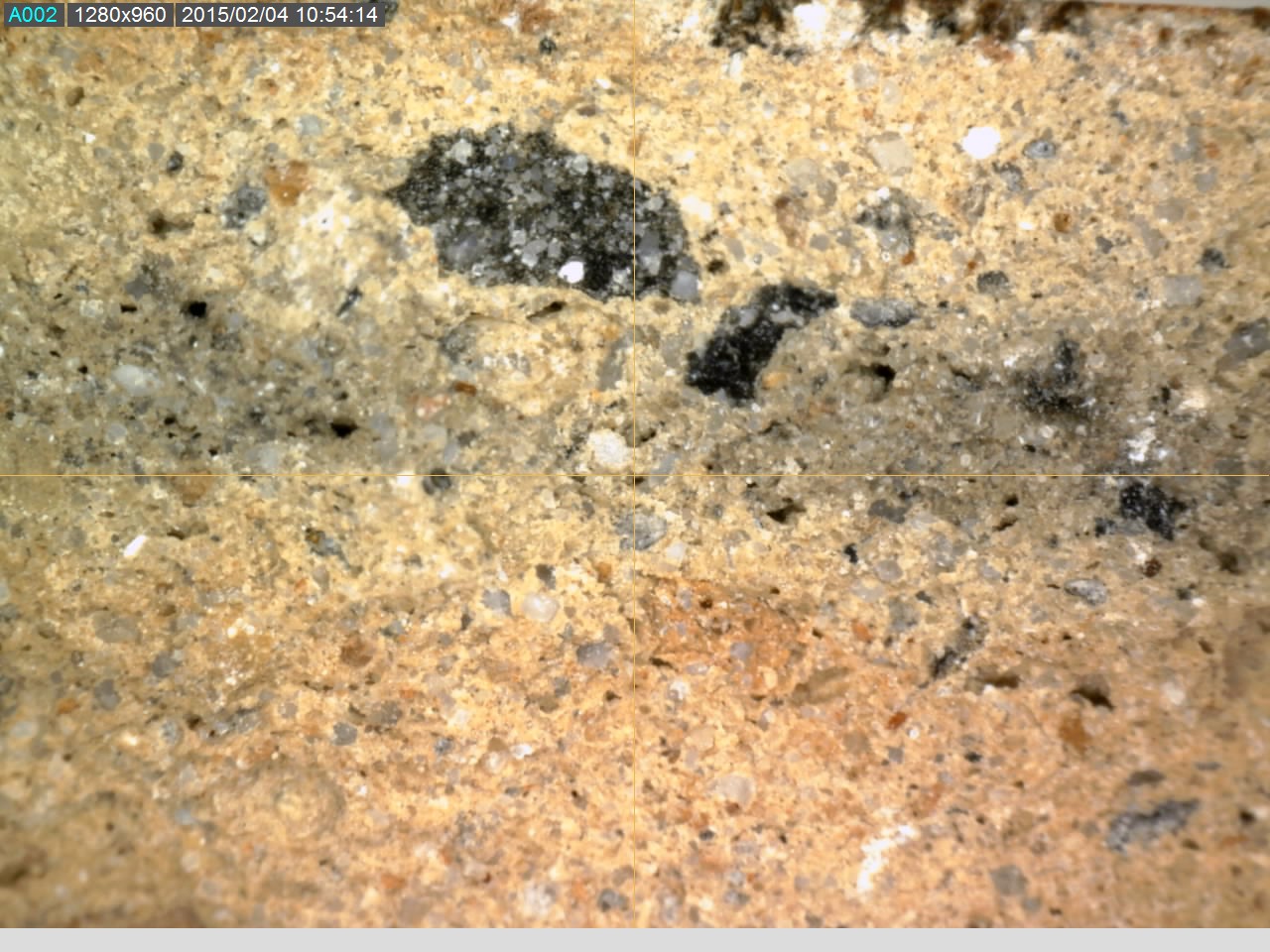

Supplement: Supplementary file 3 — Supplementary material [file mmc3.zip › Appendix A/HTN 15/HTN 15-50m-1.jpg]

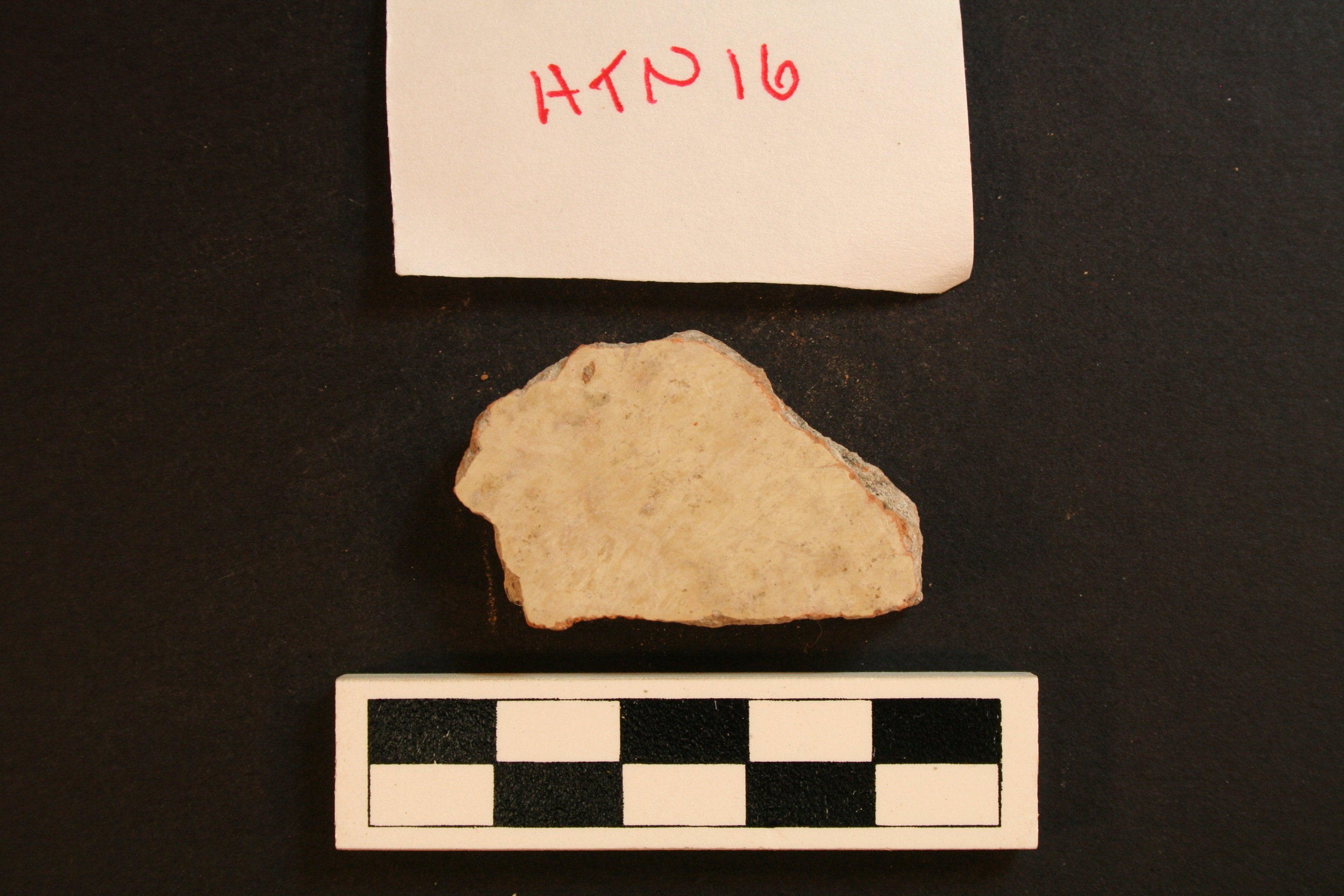

Supplement: Supplementary file 3 — Supplementary material [file mmc3.zip › Appendix A/HTN 16/16a.JPG]

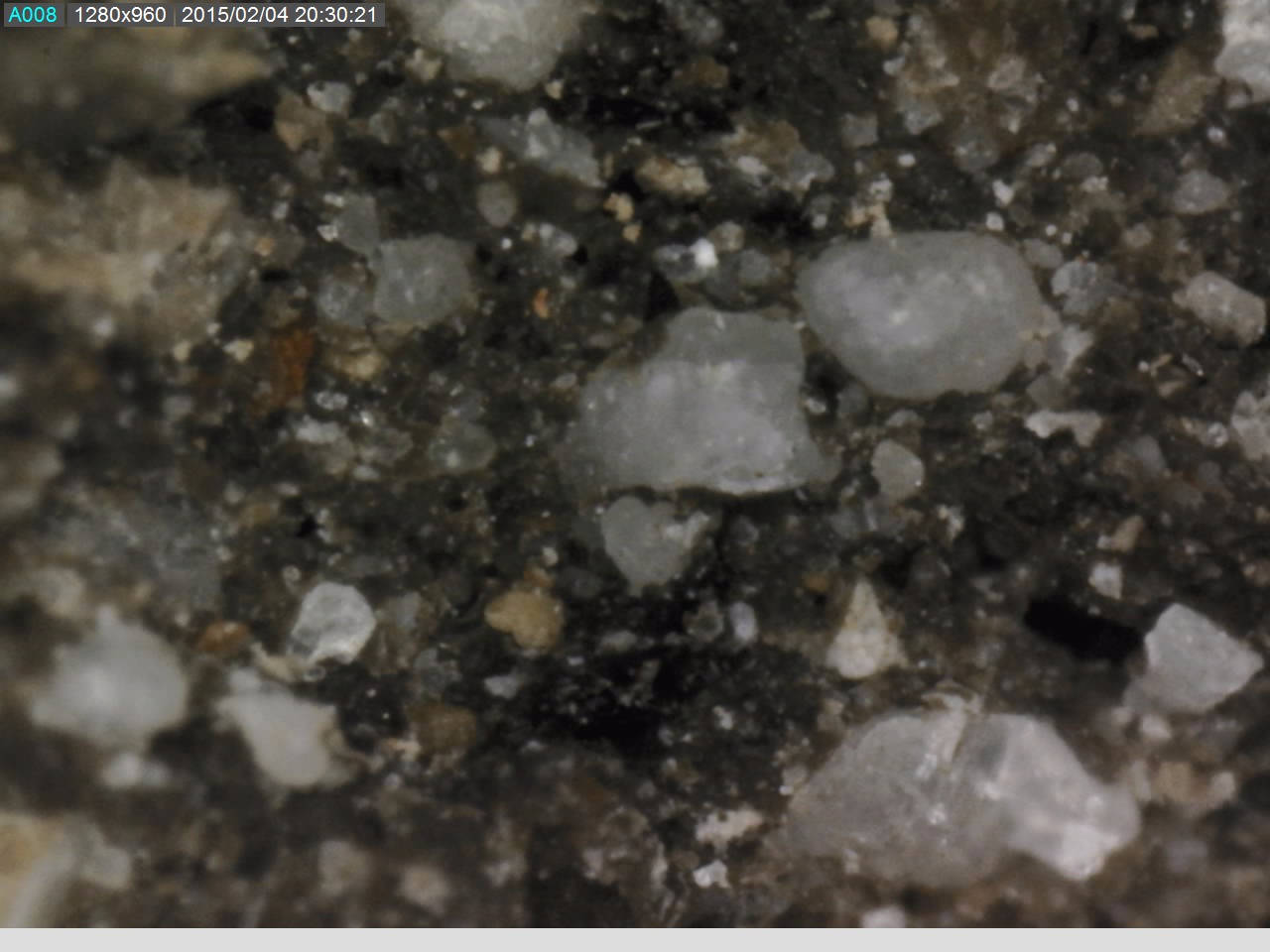

Supplement: Supplementary file 3 — Supplementary material [file mmc3.zip › Appendix A/HTN 16/HTN 16-250m-0.jpg]

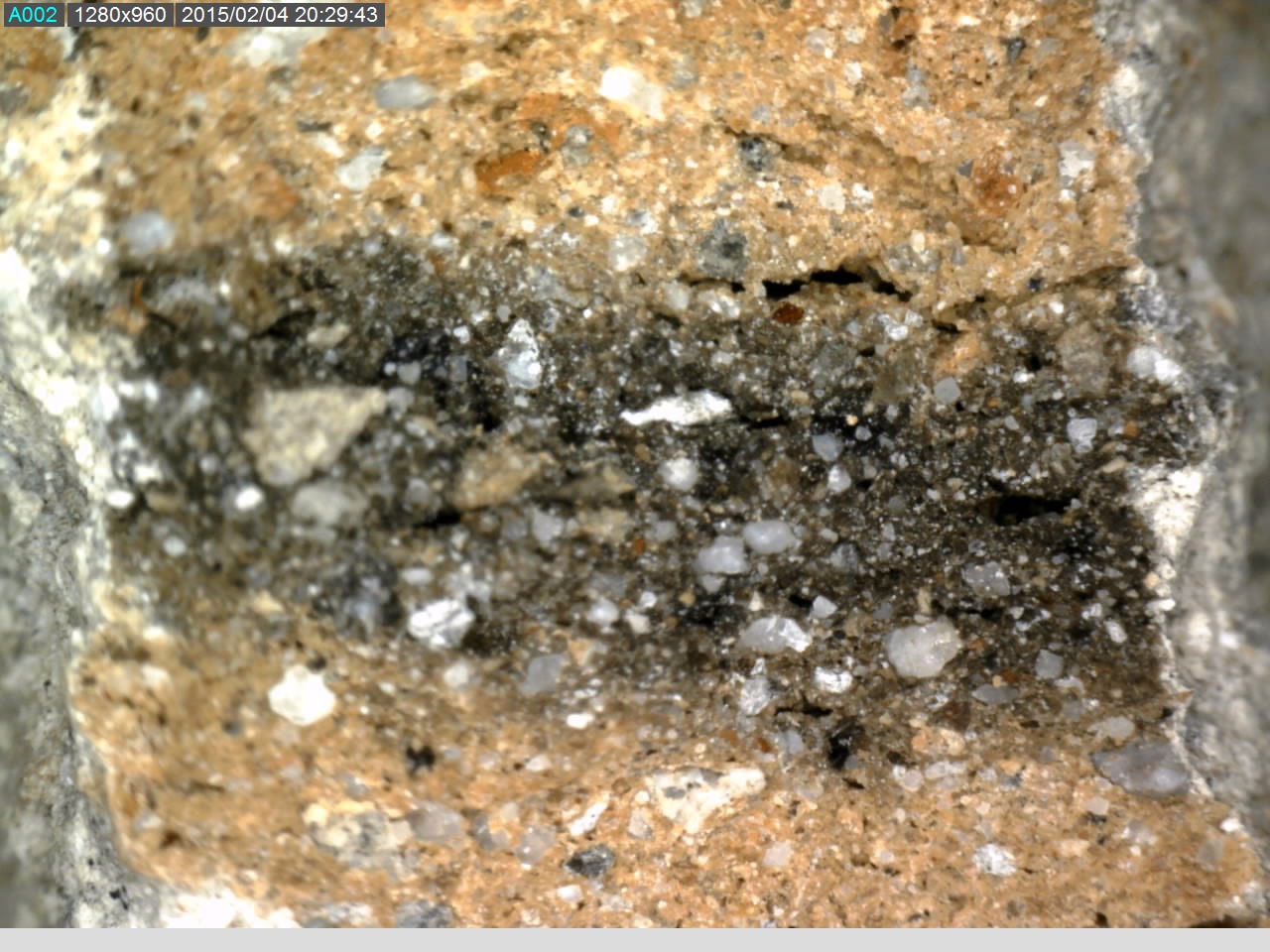

Supplement: Supplementary file 3 — Supplementary material [file mmc3.zip › Appendix A/HTN 16/HTN 16-50m-1.jpg]

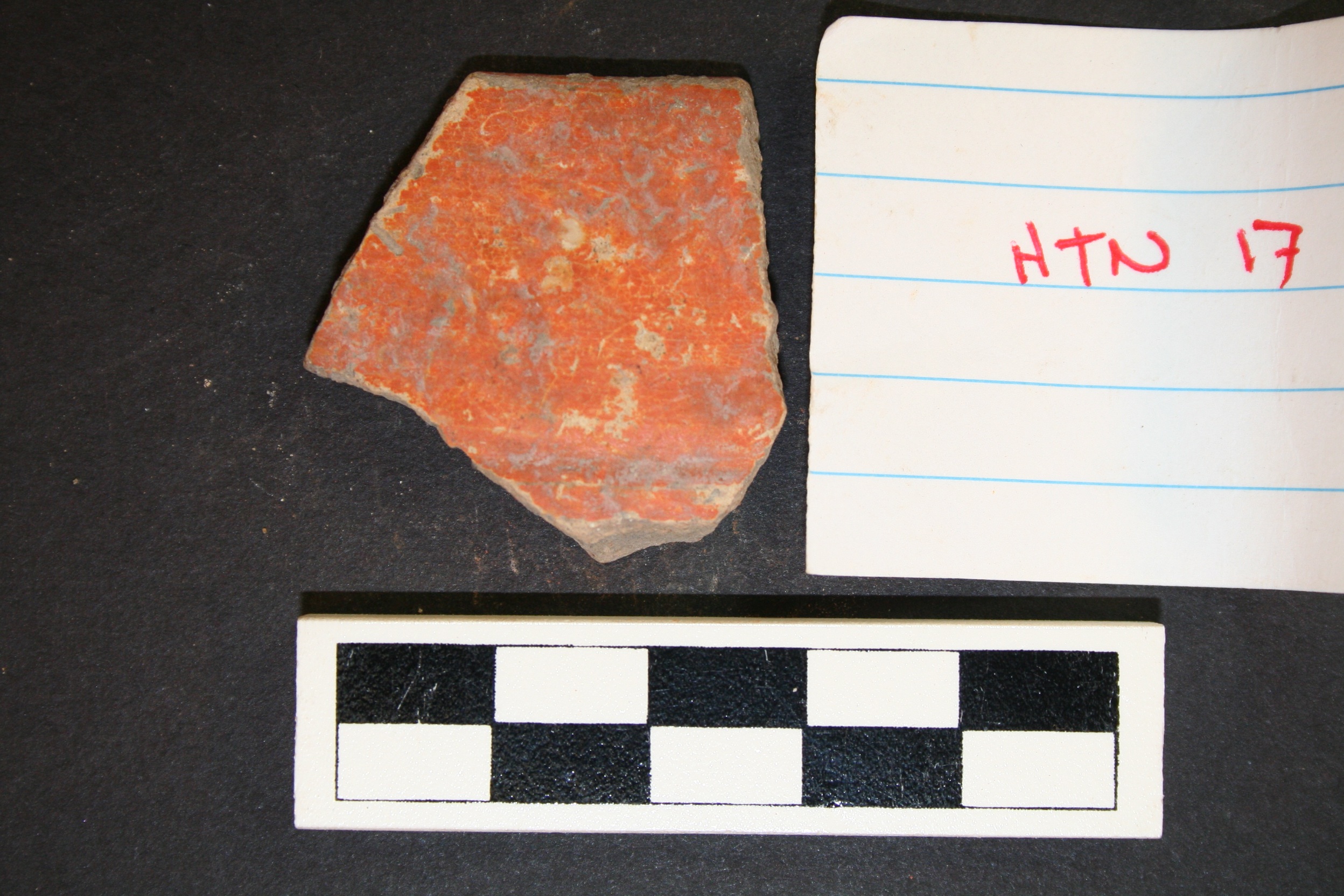

Supplement: Supplementary file 3 — Supplementary material [file mmc3.zip › Appendix A/HTN 17/17a.JPG]

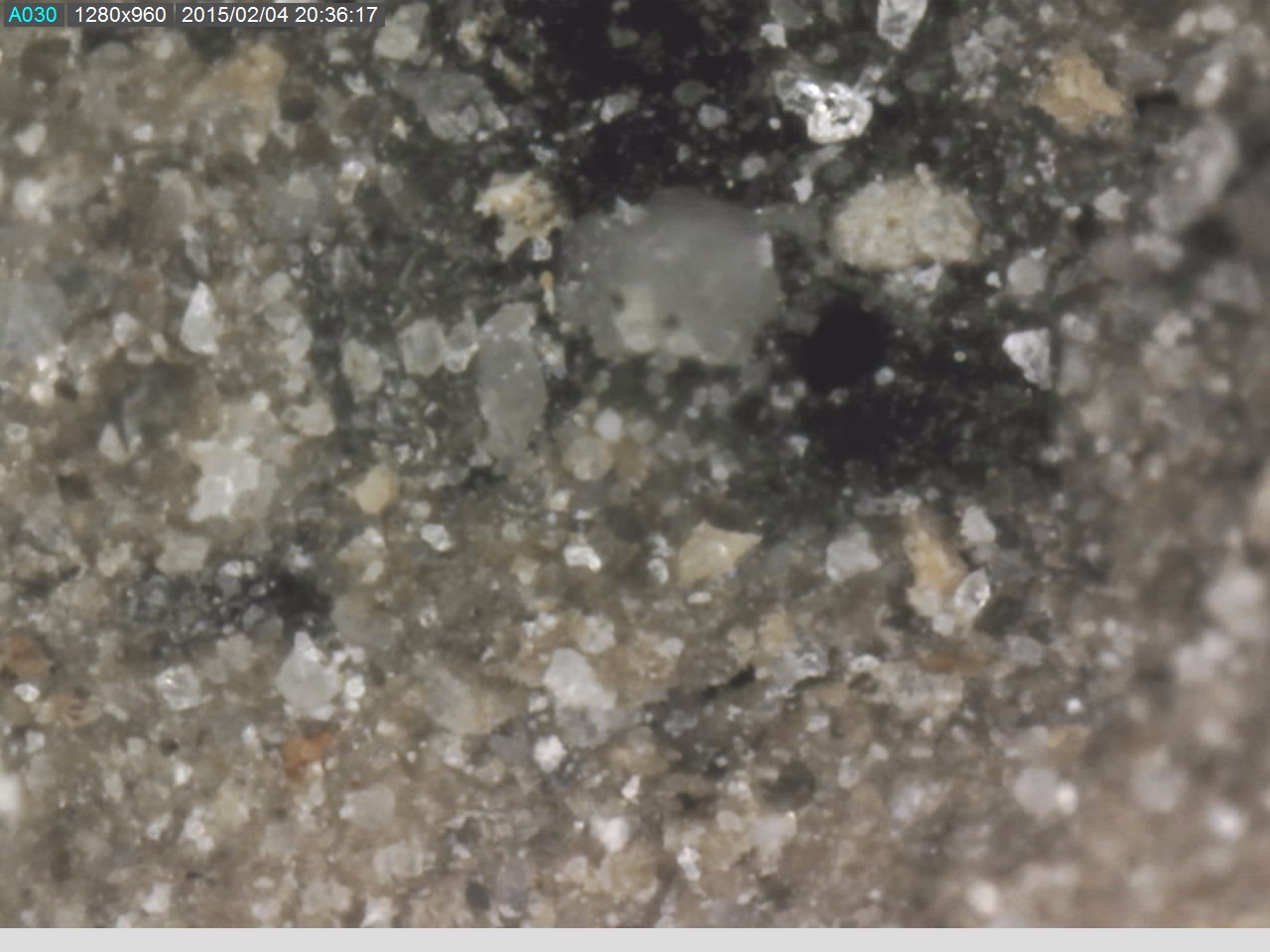

Supplement: Supplementary file 3 — Supplementary material [file mmc3.zip › Appendix A/HTN 17/HTN 17-250m-4.jpg]

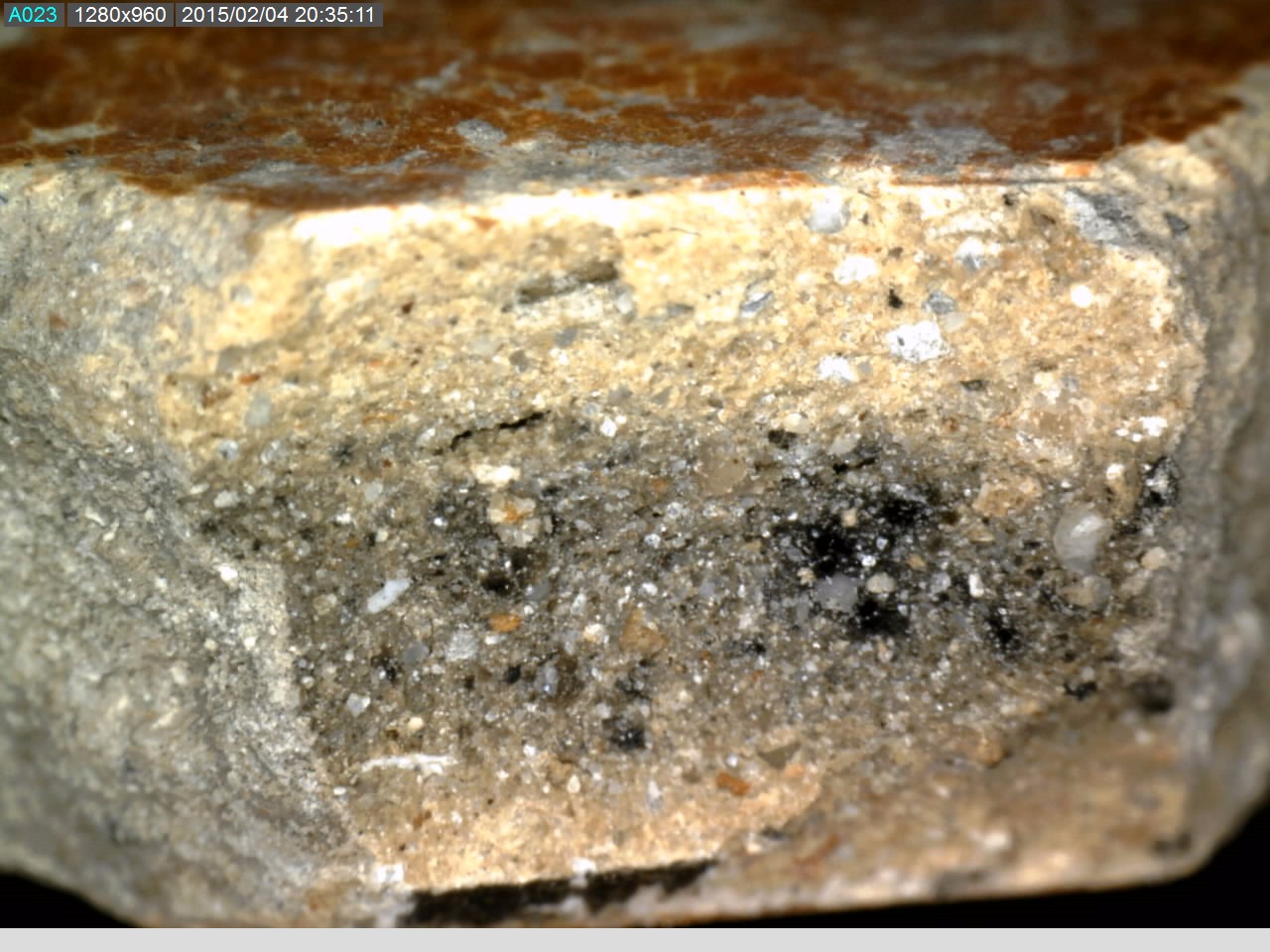

Supplement: Supplementary file 3 — Supplementary material [file mmc3.zip › Appendix A/HTN 17/HTN 17-50m-5.jpg]

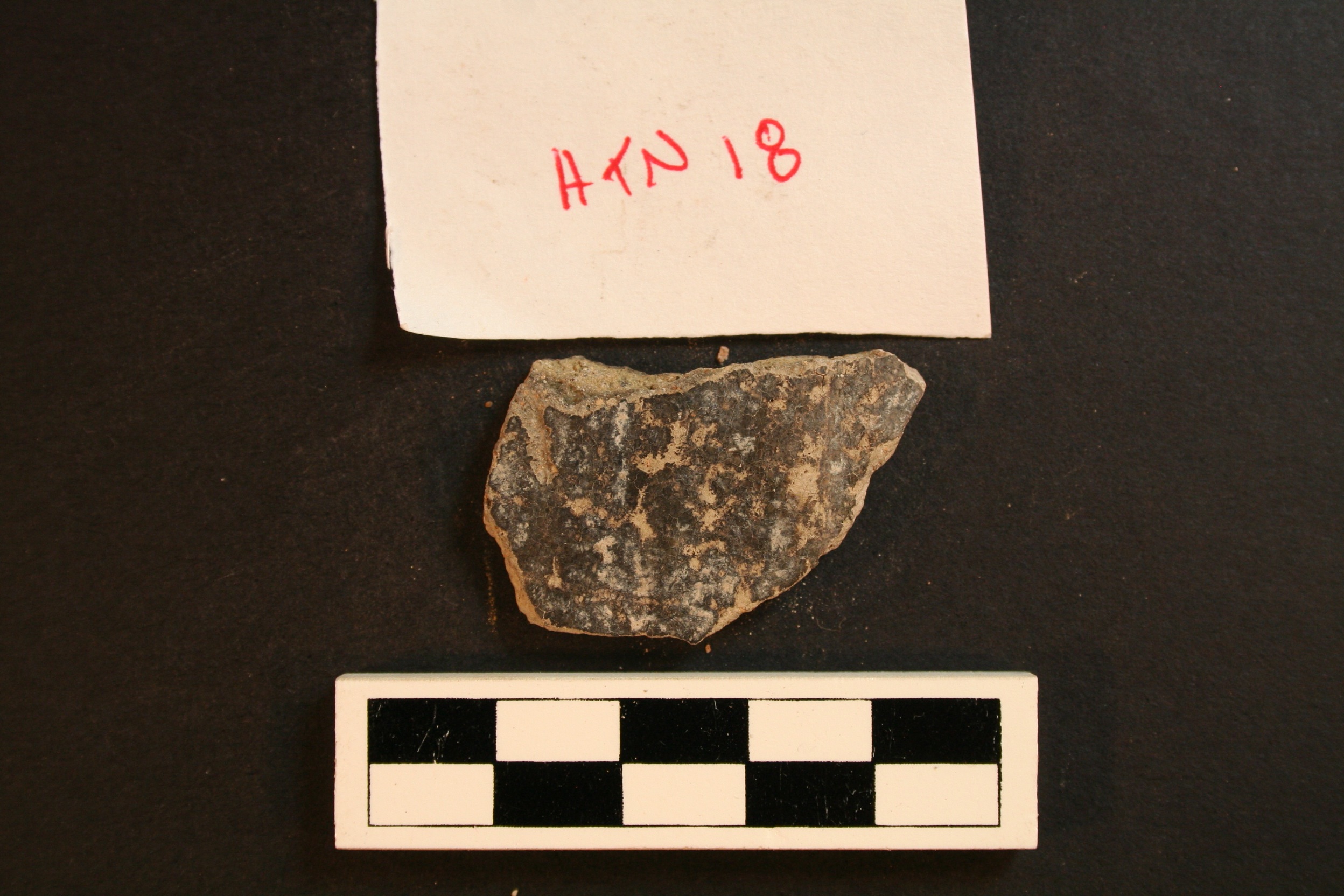

Supplement: Supplementary file 3 — Supplementary material [file mmc3.zip › Appendix A/HTN 18/18a.JPG]

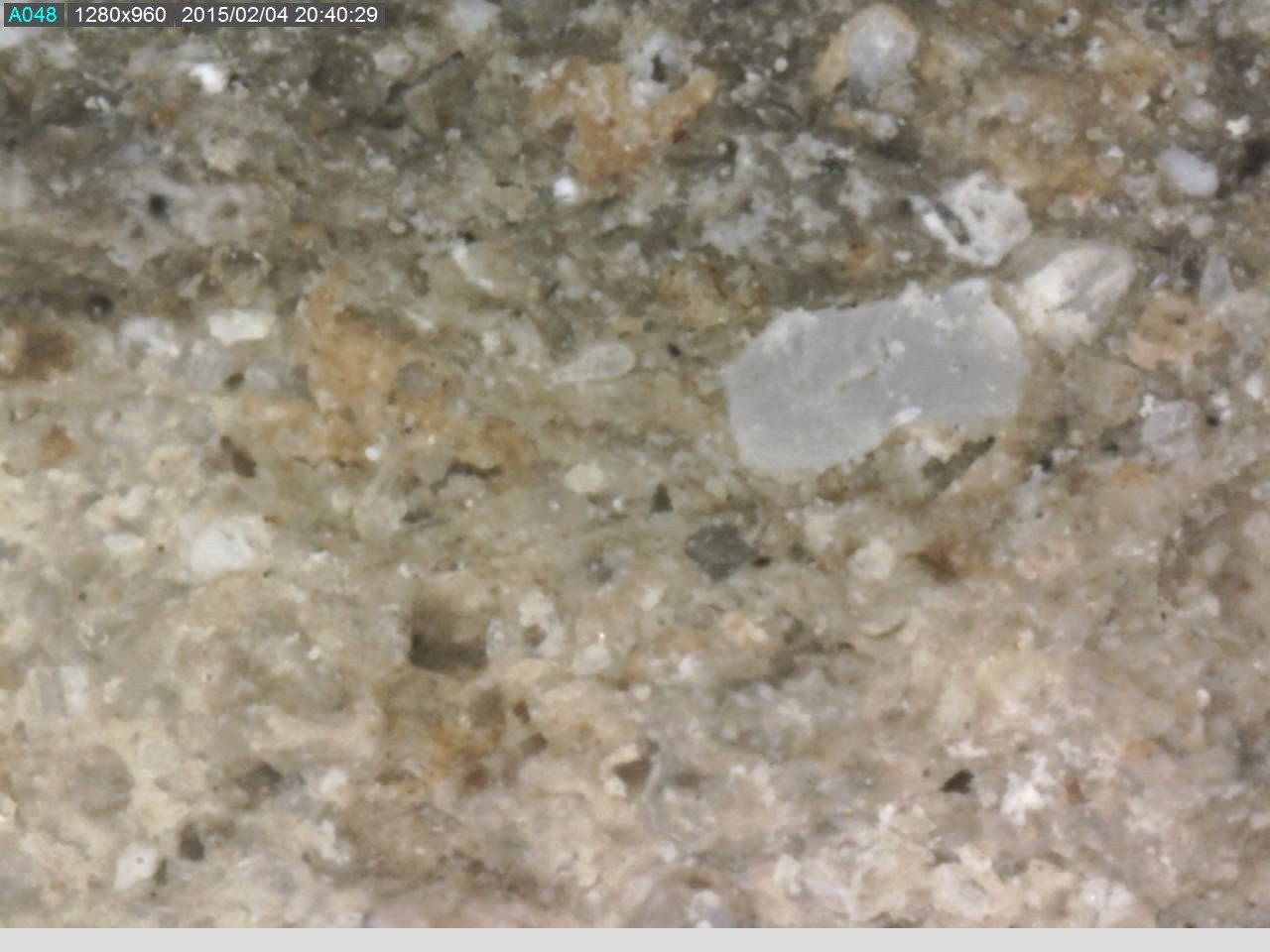

Supplement: Supplementary file 3 — Supplementary material [file mmc3.zip › Appendix A/HTN 18/HTN 18-250m-5.jpg]

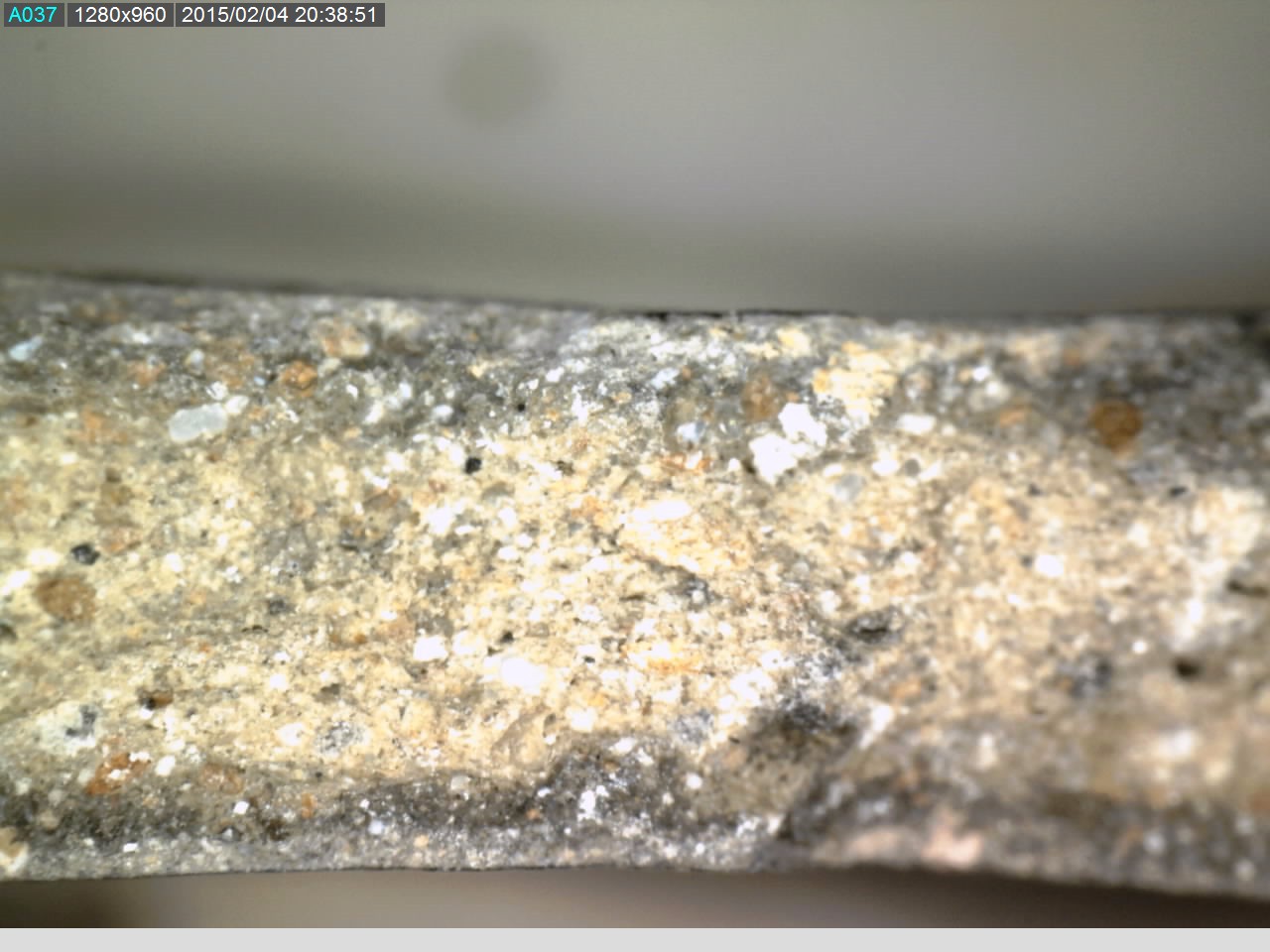

Supplement: Supplementary file 3 — Supplementary material [file mmc3.zip › Appendix A/HTN 18/HTN 18-50m-0.jpg]

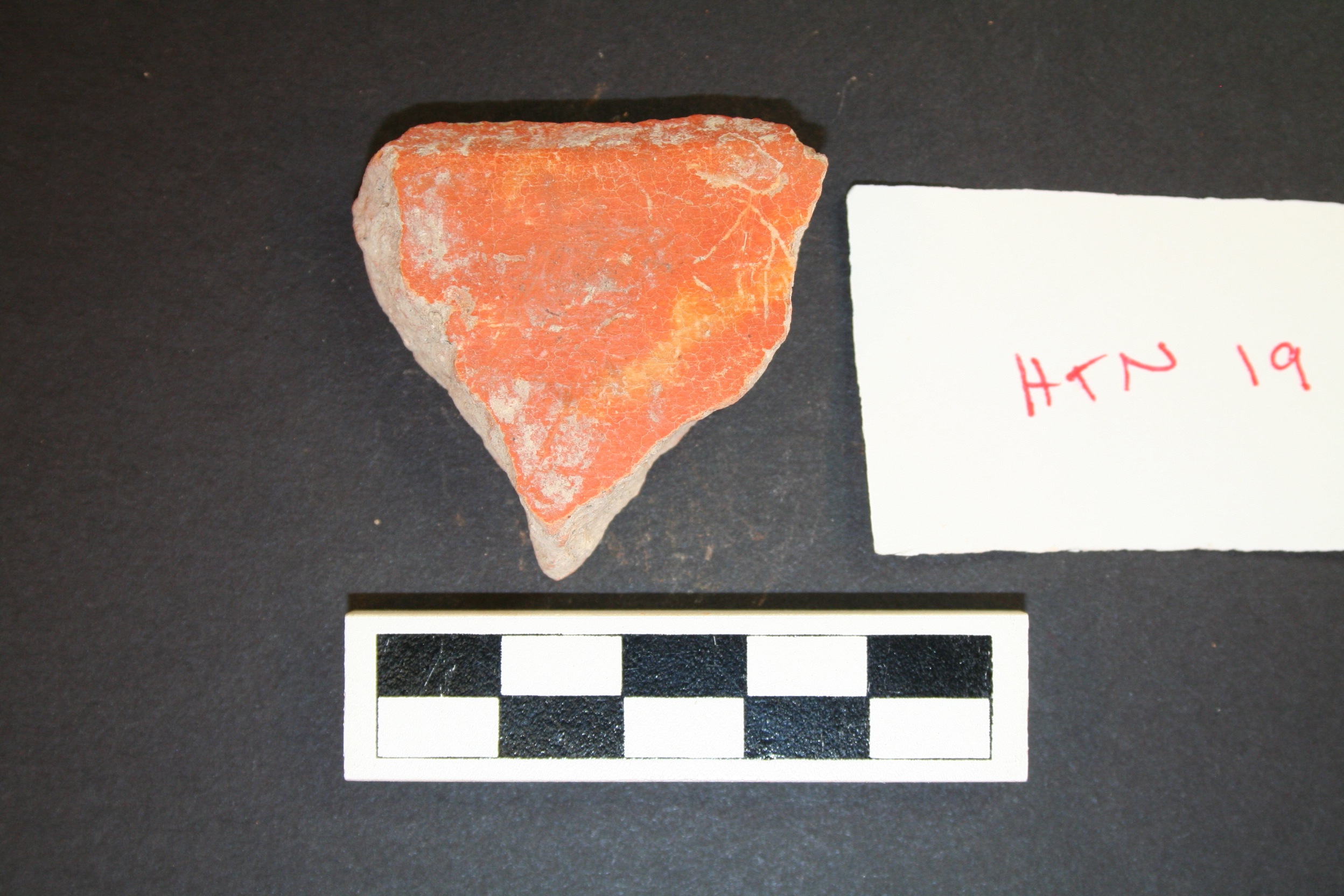

Supplement: Supplementary file 3 — Supplementary material [file mmc3.zip › Appendix A/HTN 19/19a.JPG]

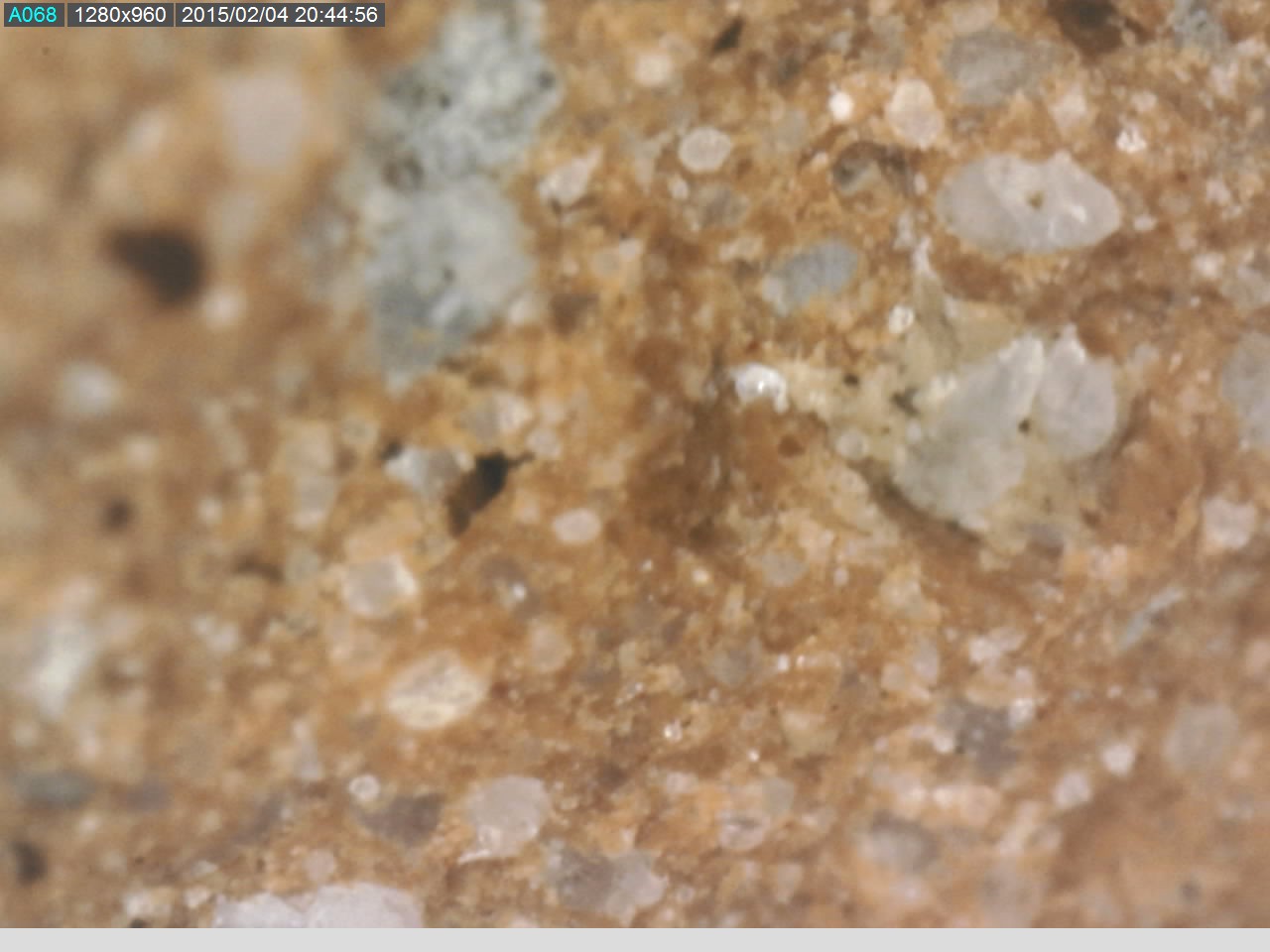

Supplement: Supplementary file 3 — Supplementary material [file mmc3.zip › Appendix A/HTN 19/HTN 19-250m-8.jpg]

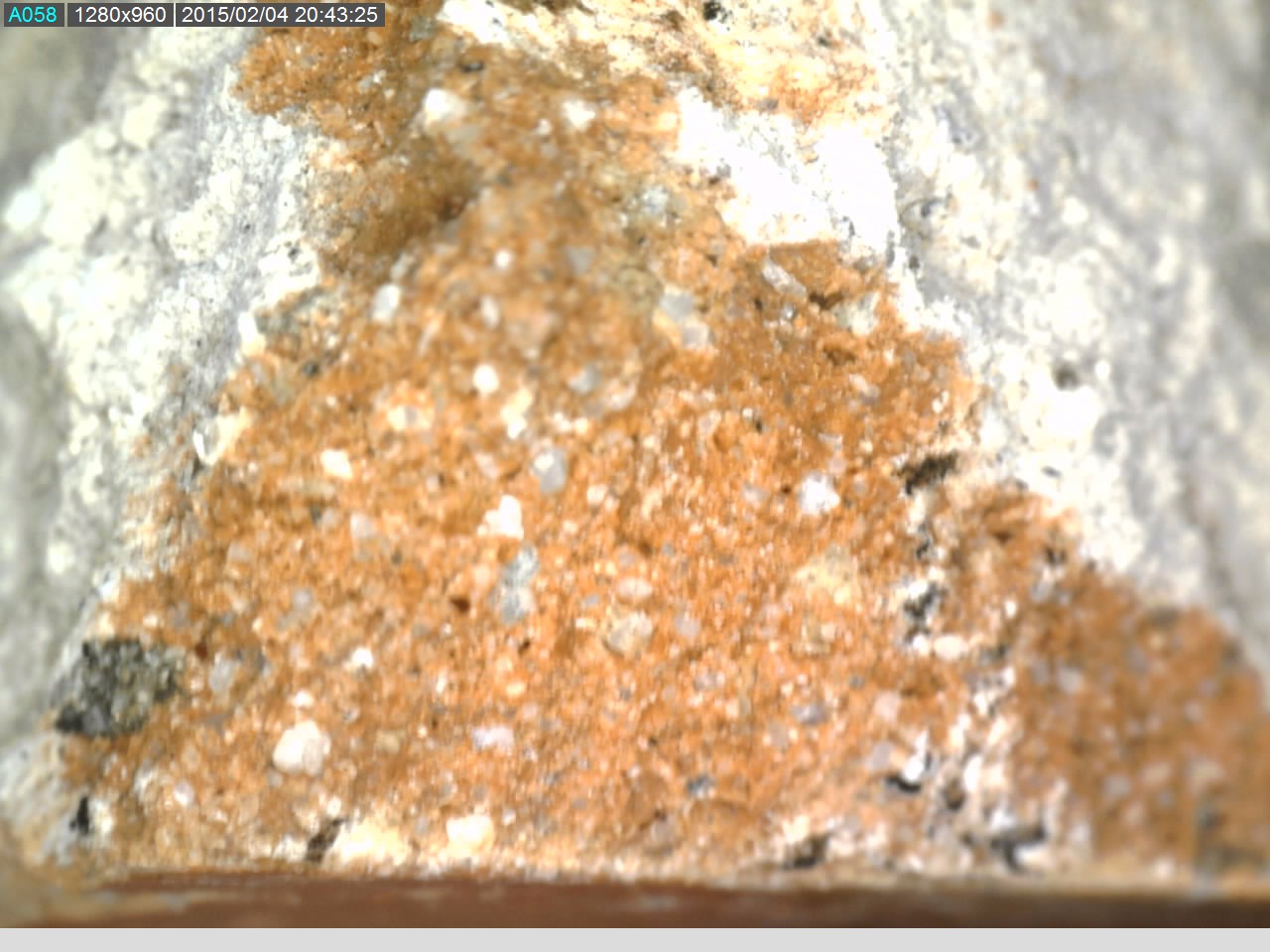

Supplement: Supplementary file 3 — Supplementary material [file mmc3.zip › Appendix A/HTN 19/HTN 19-50m-9.jpg]

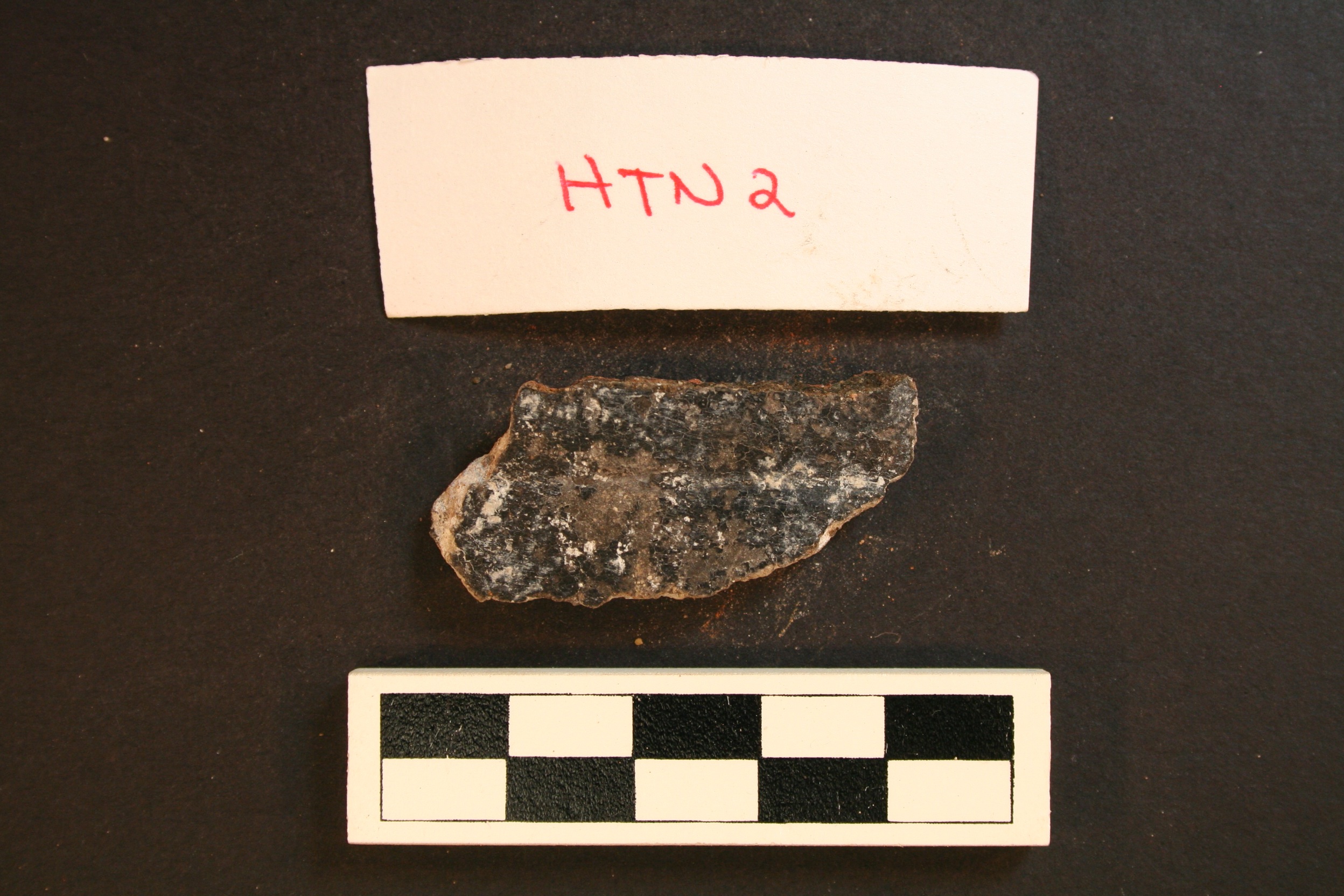

Supplement: Supplementary file 3 — Supplementary material [file mmc3.zip › Appendix A/HTN 2/2b.JPG]

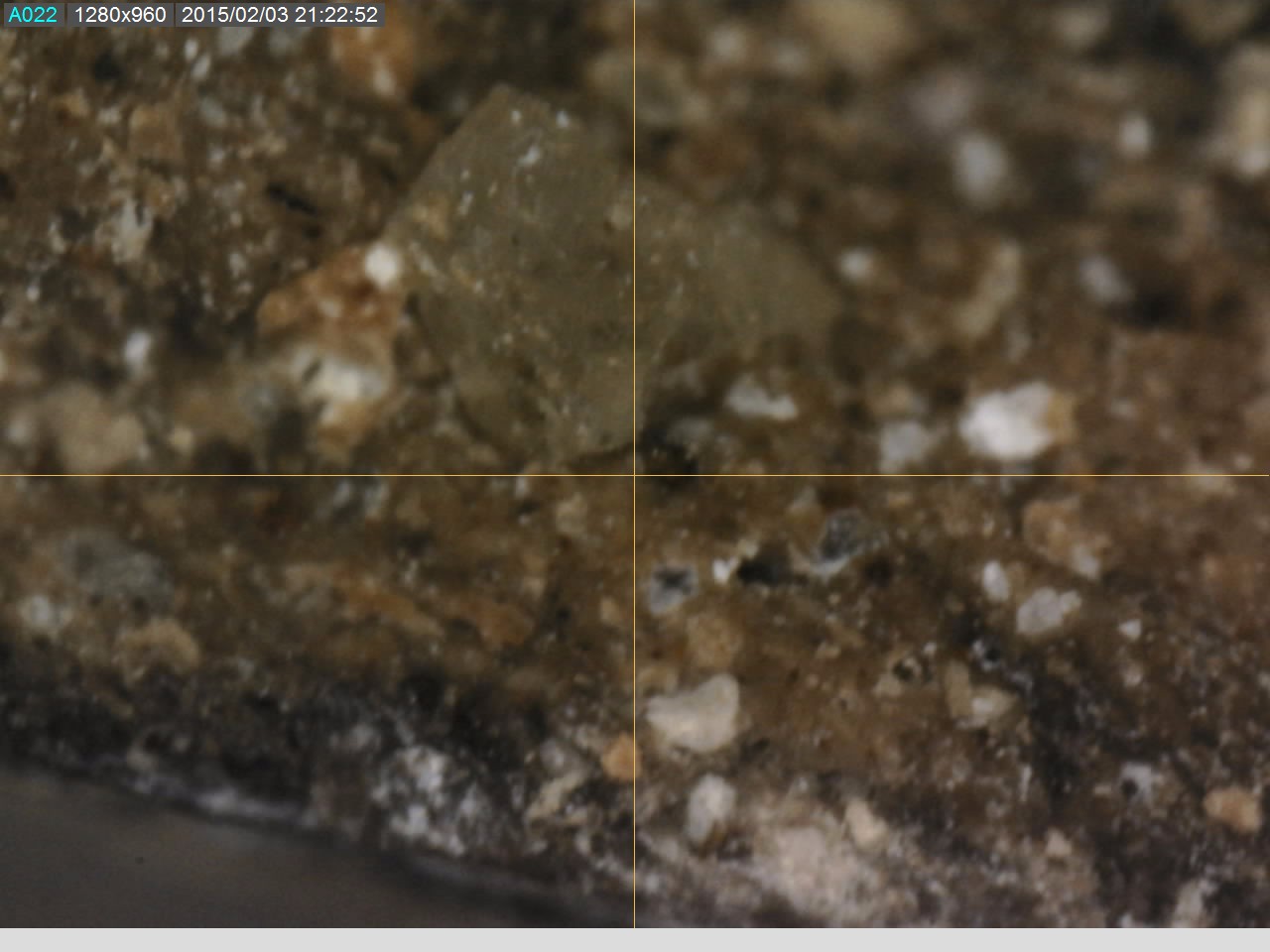

Supplement: Supplementary file 3 — Supplementary material [file mmc3.zip › Appendix A/HTN 2/HTN 2-250m-5.jpg]

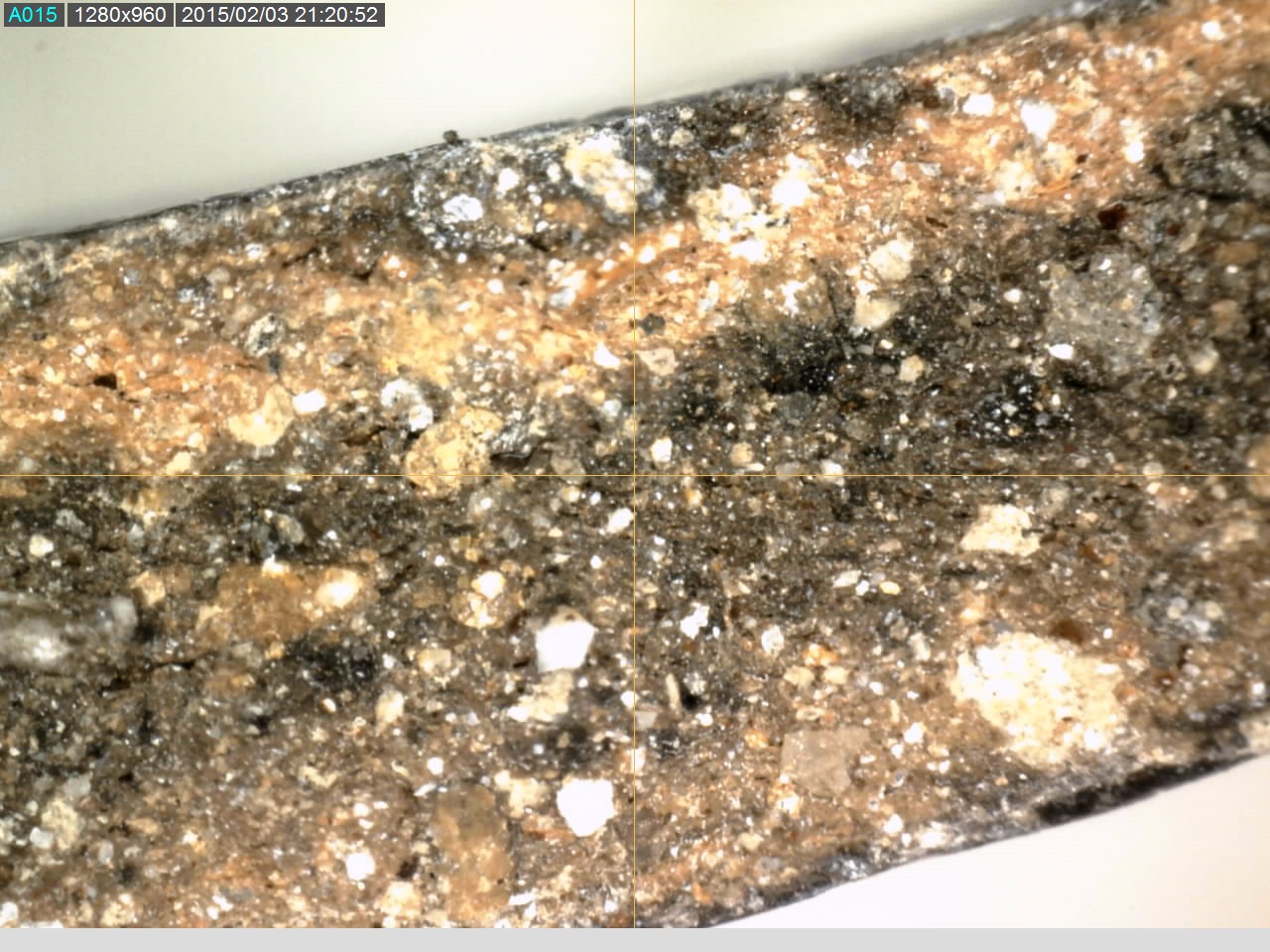

Supplement: Supplementary file 3 — Supplementary material [file mmc3.zip › Appendix A/HTN 2/HTN 2-50m-3.jpg]

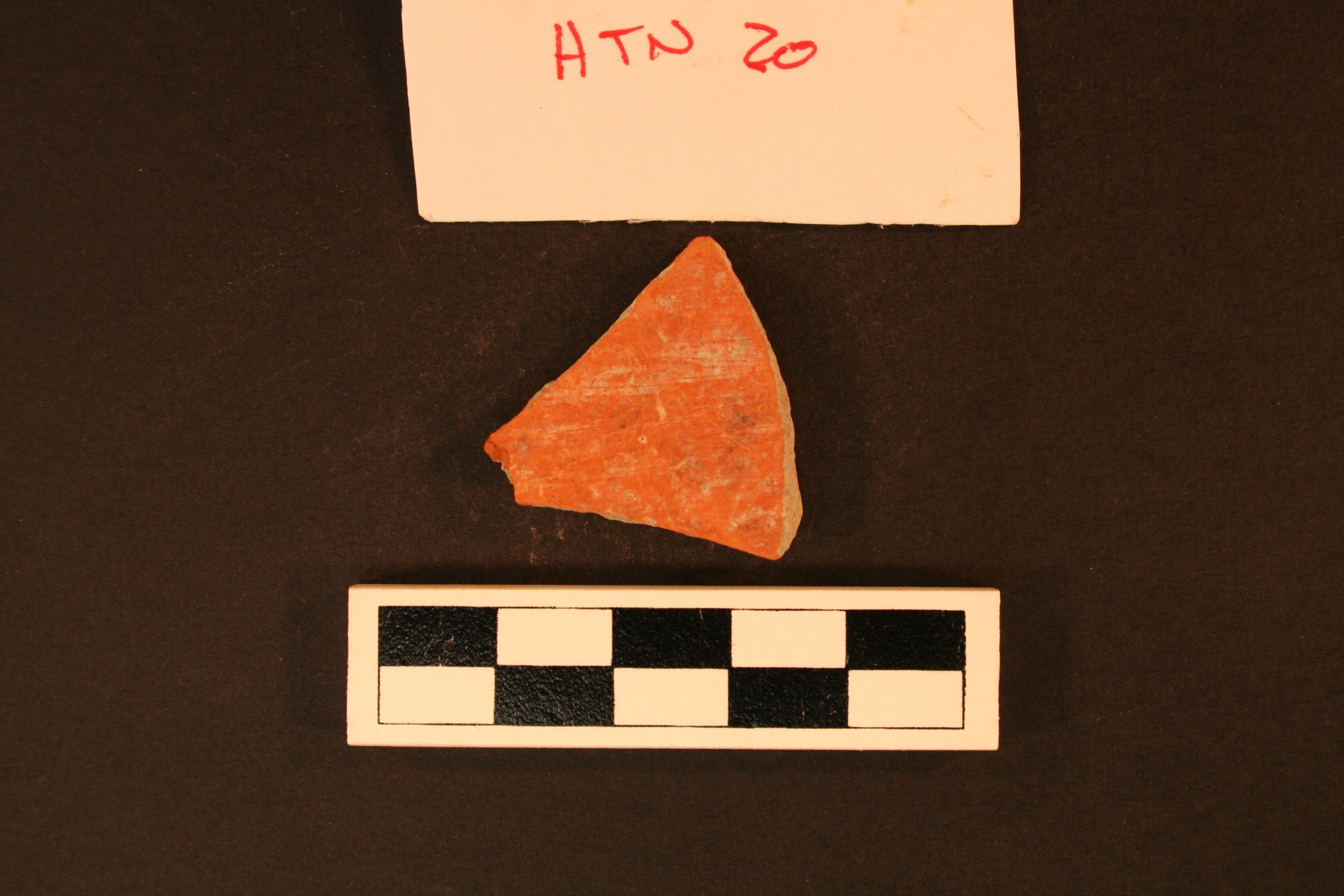

Supplement: Supplementary file 3 — Supplementary material [file mmc3.zip › Appendix A/HTN 20/20a.JPG]

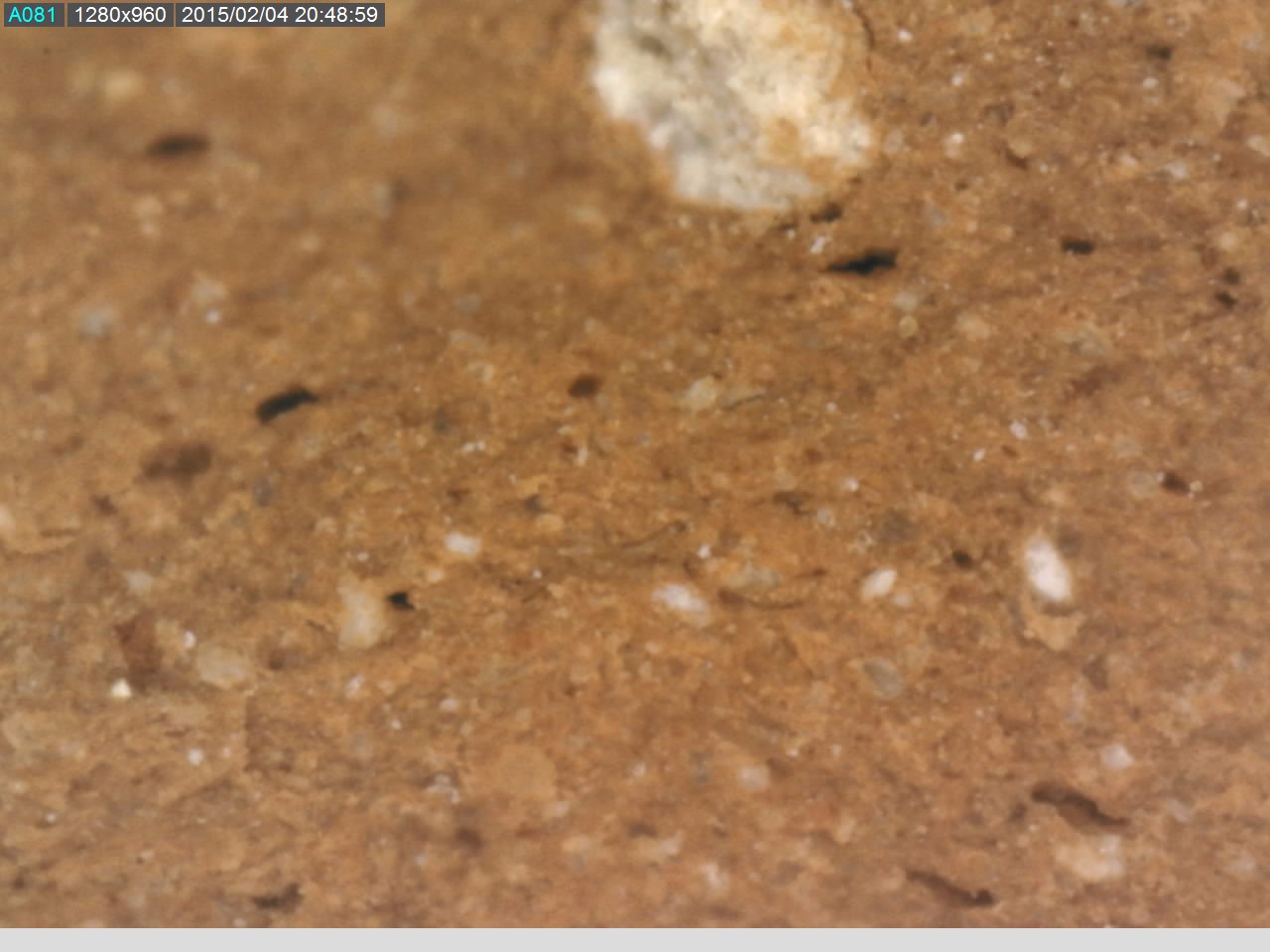

Supplement: Supplementary file 3 — Supplementary material [file mmc3.zip › Appendix A/HTN 20/HTN 20-250m-4.jpg]

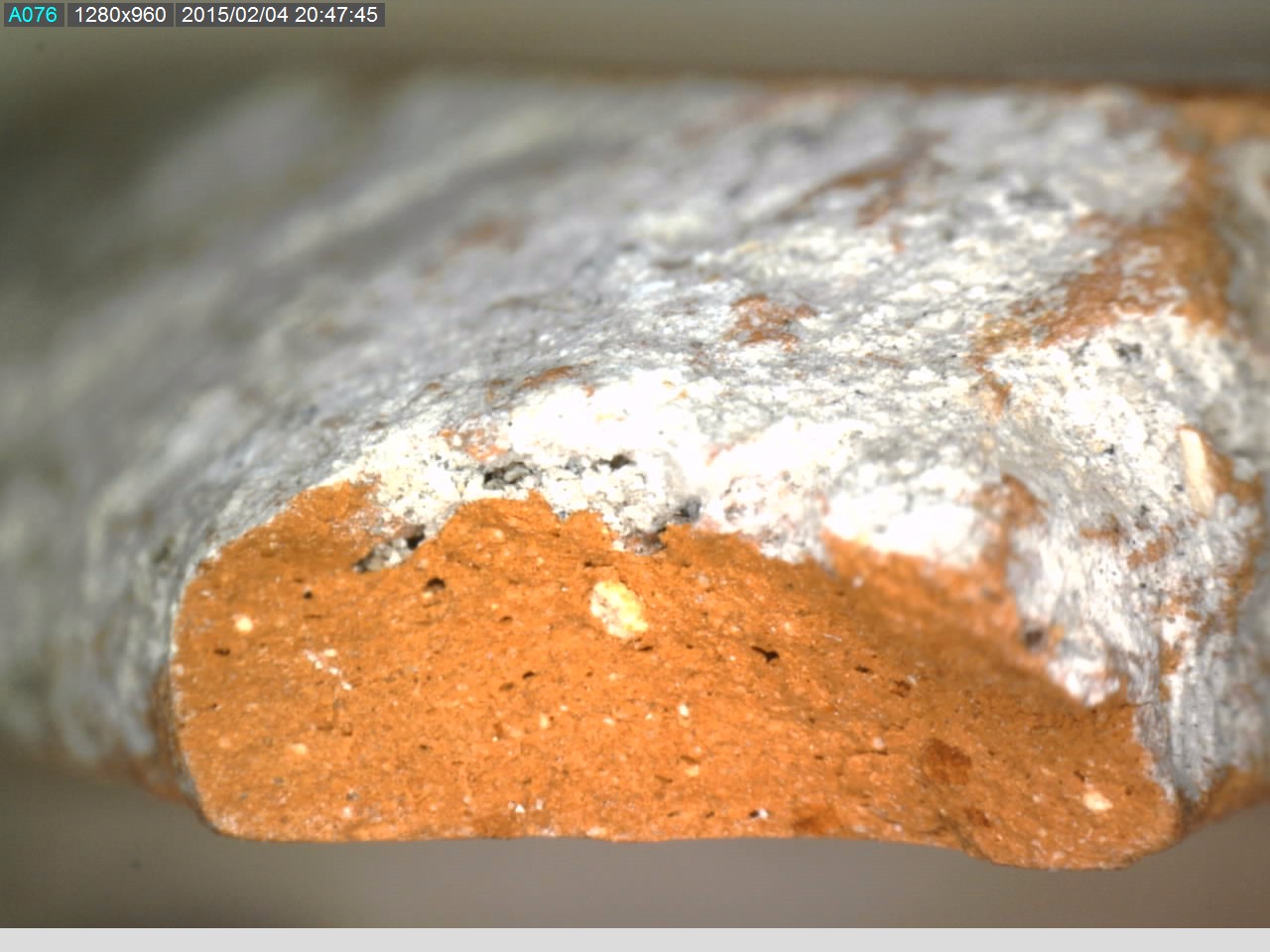

Supplement: Supplementary file 3 — Supplementary material [file mmc3.zip › Appendix A/HTN 20/HTN 20-50m-6.jpg]

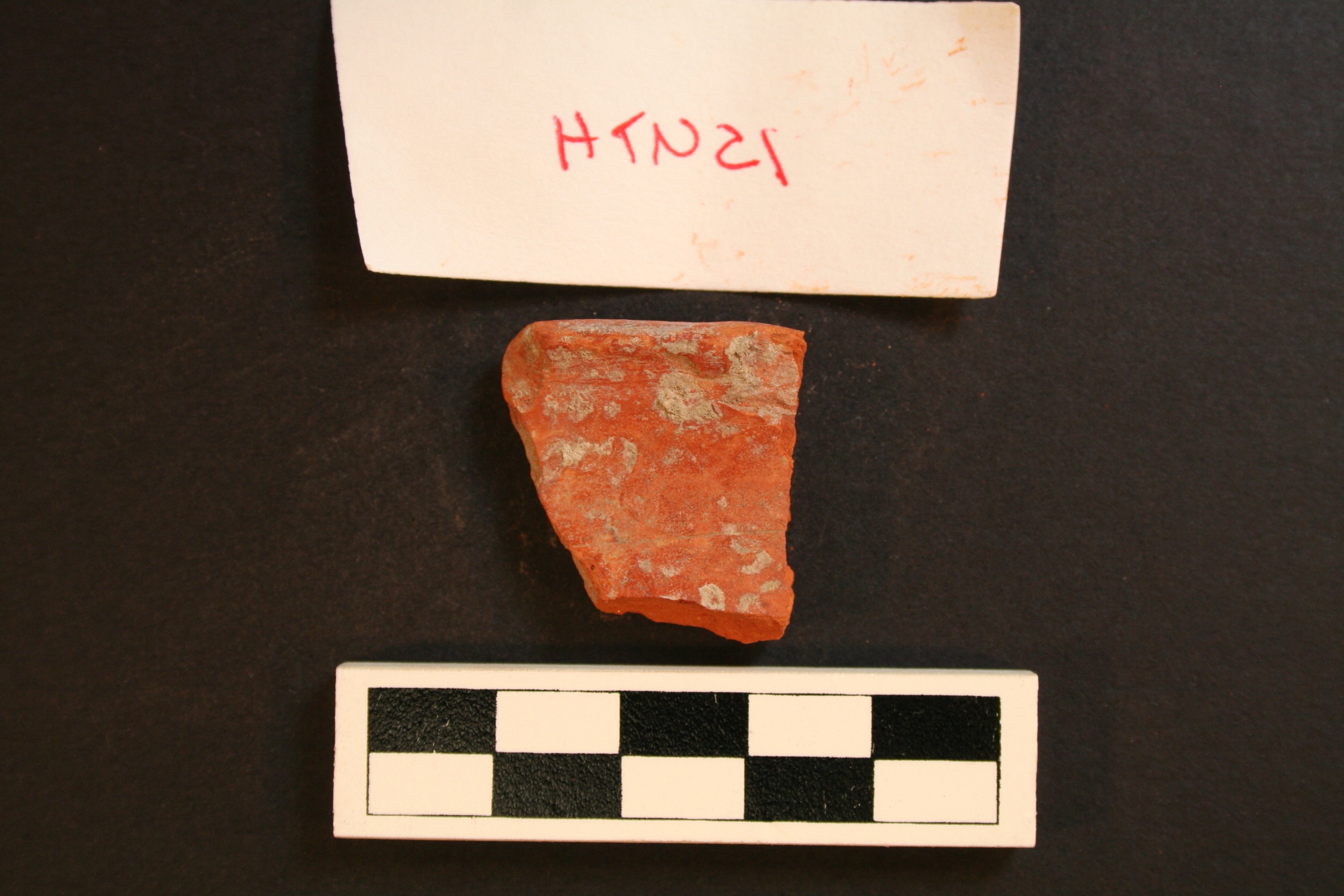

Supplement: Supplementary file 3 — Supplementary material [file mmc3.zip › Appendix A/HTN 21/21a.JPG]

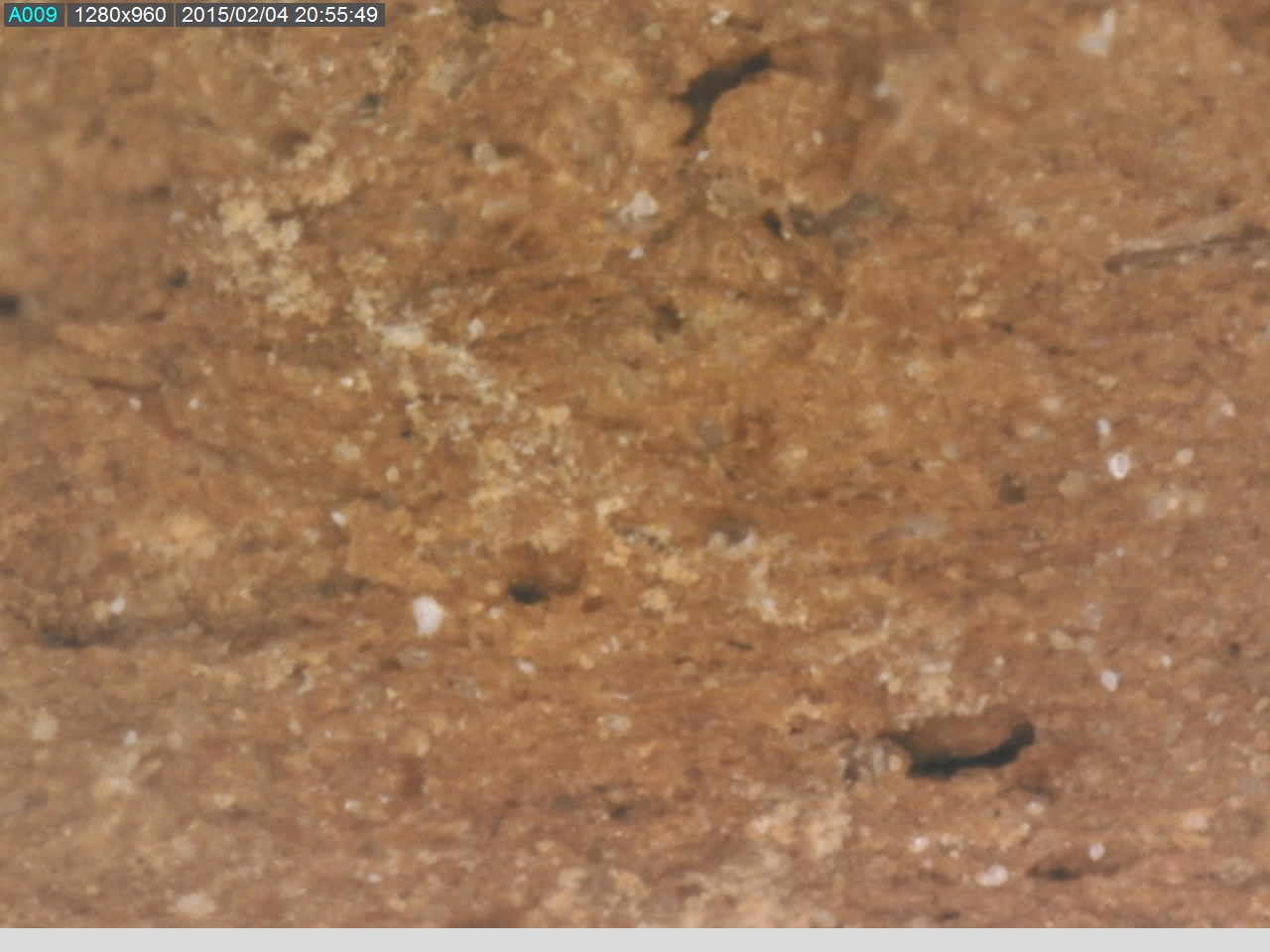

Supplement: Supplementary file 3 — Supplementary material [file mmc3.zip › Appendix A/HTN 21/HTN 21-250m-2.jpg]

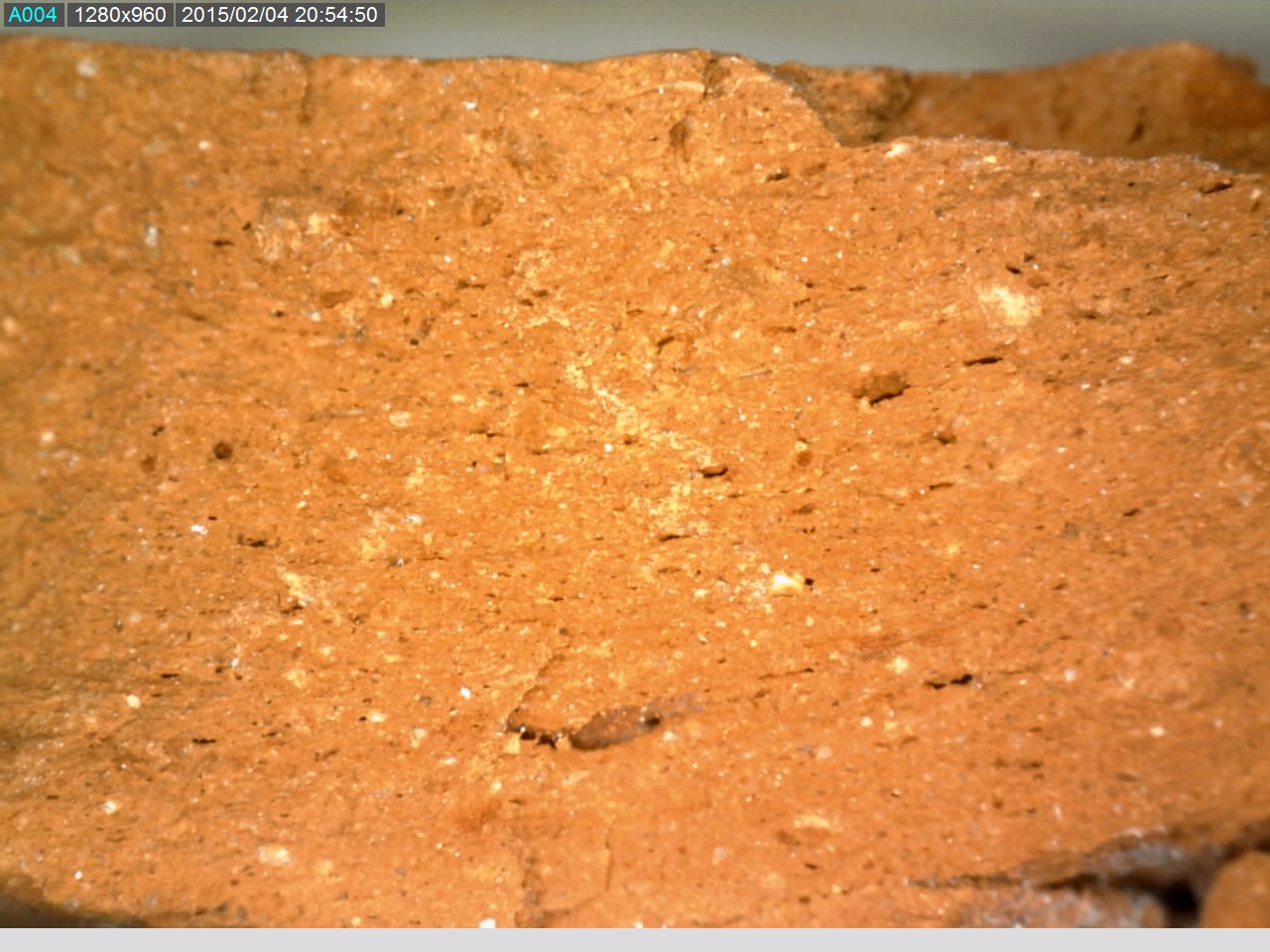

Supplement: Supplementary file 3 — Supplementary material [file mmc3.zip › Appendix A/HTN 21/HTN 21-50m-3.jpg]

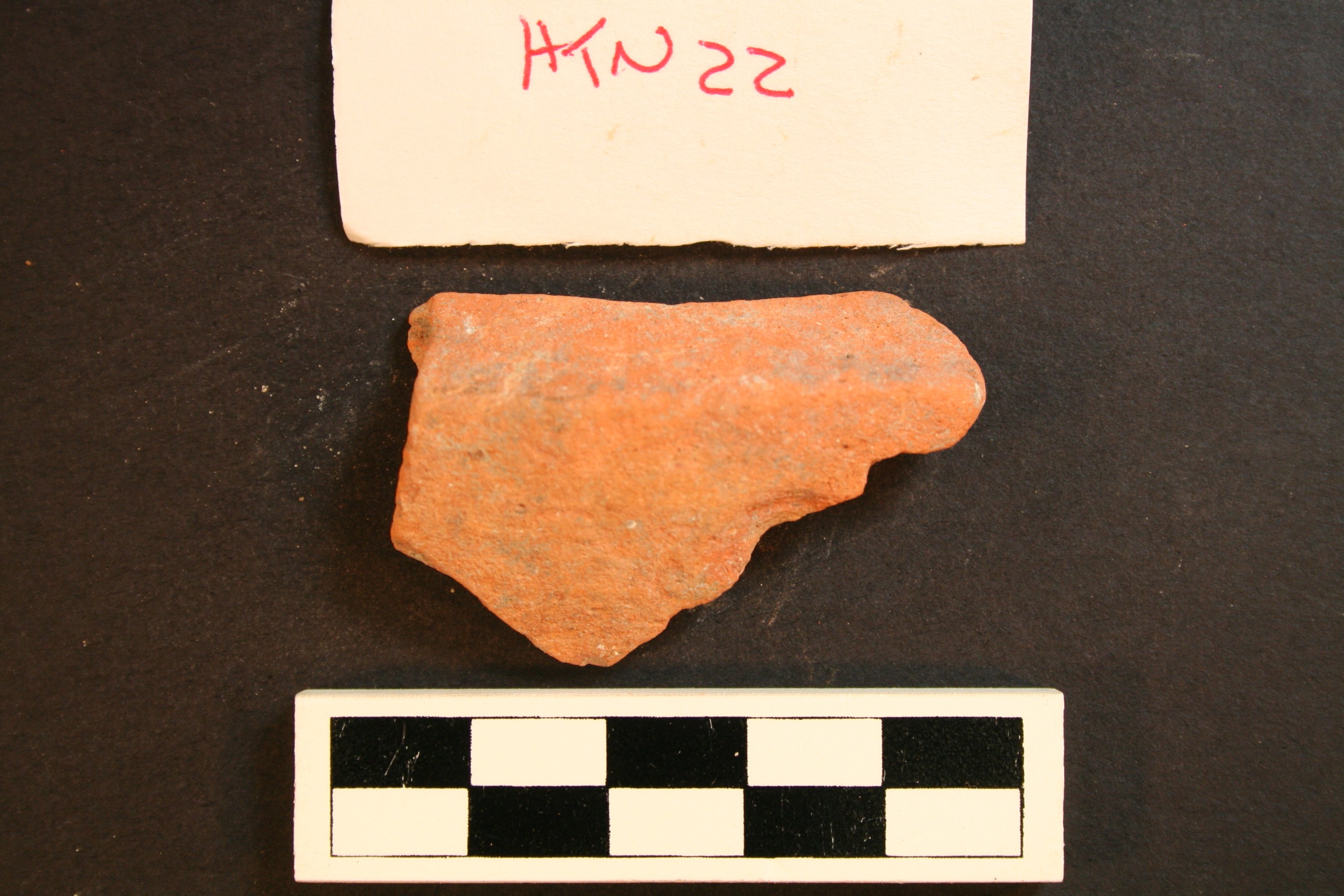

Supplement: Supplementary file 3 — Supplementary material [file mmc3.zip › Appendix A/HTN 22/22a.JPG]

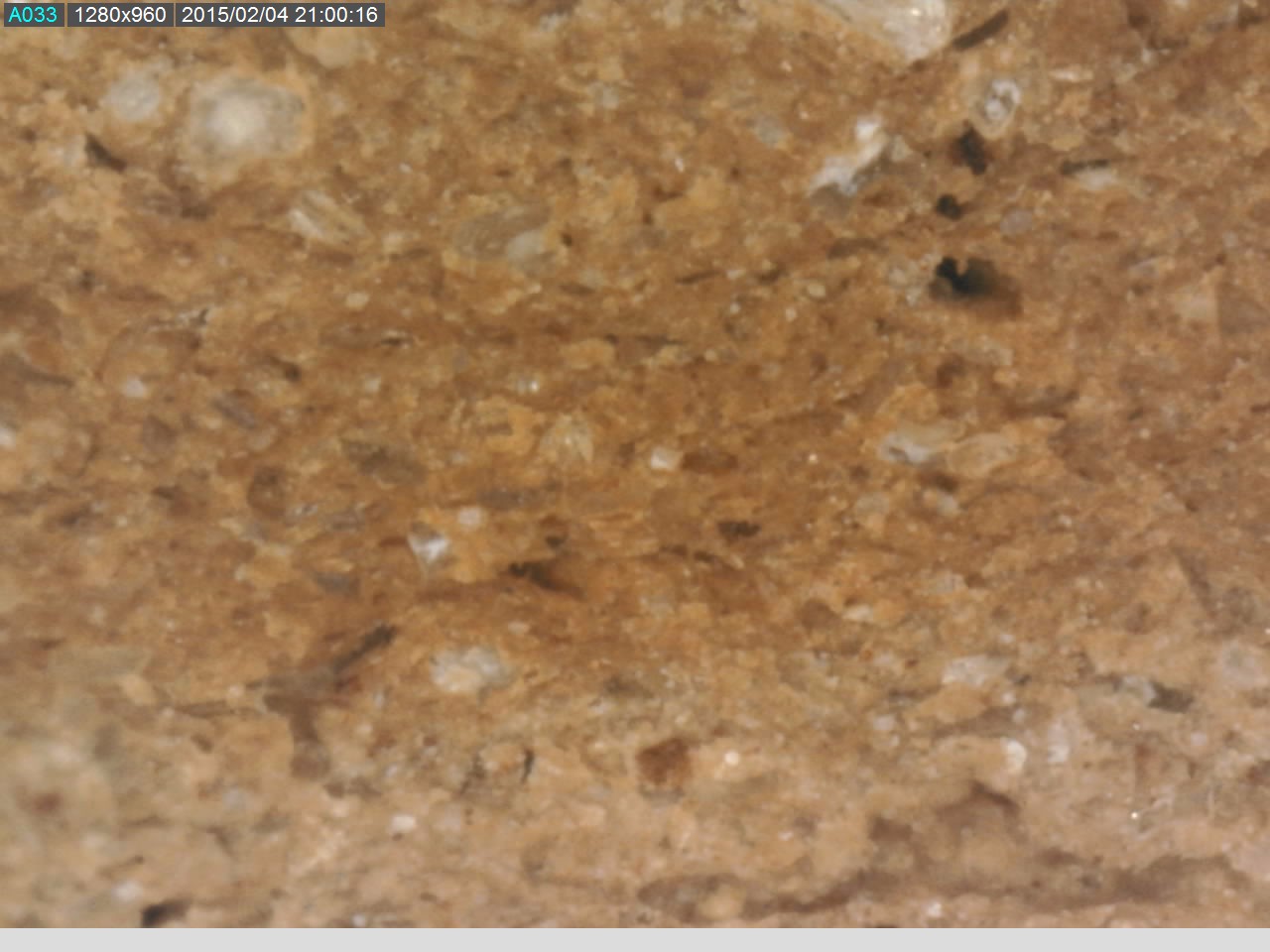

Supplement: Supplementary file 3 — Supplementary material [file mmc3.zip › Appendix A/HTN 22/HTN 22-250m-7.jpg]

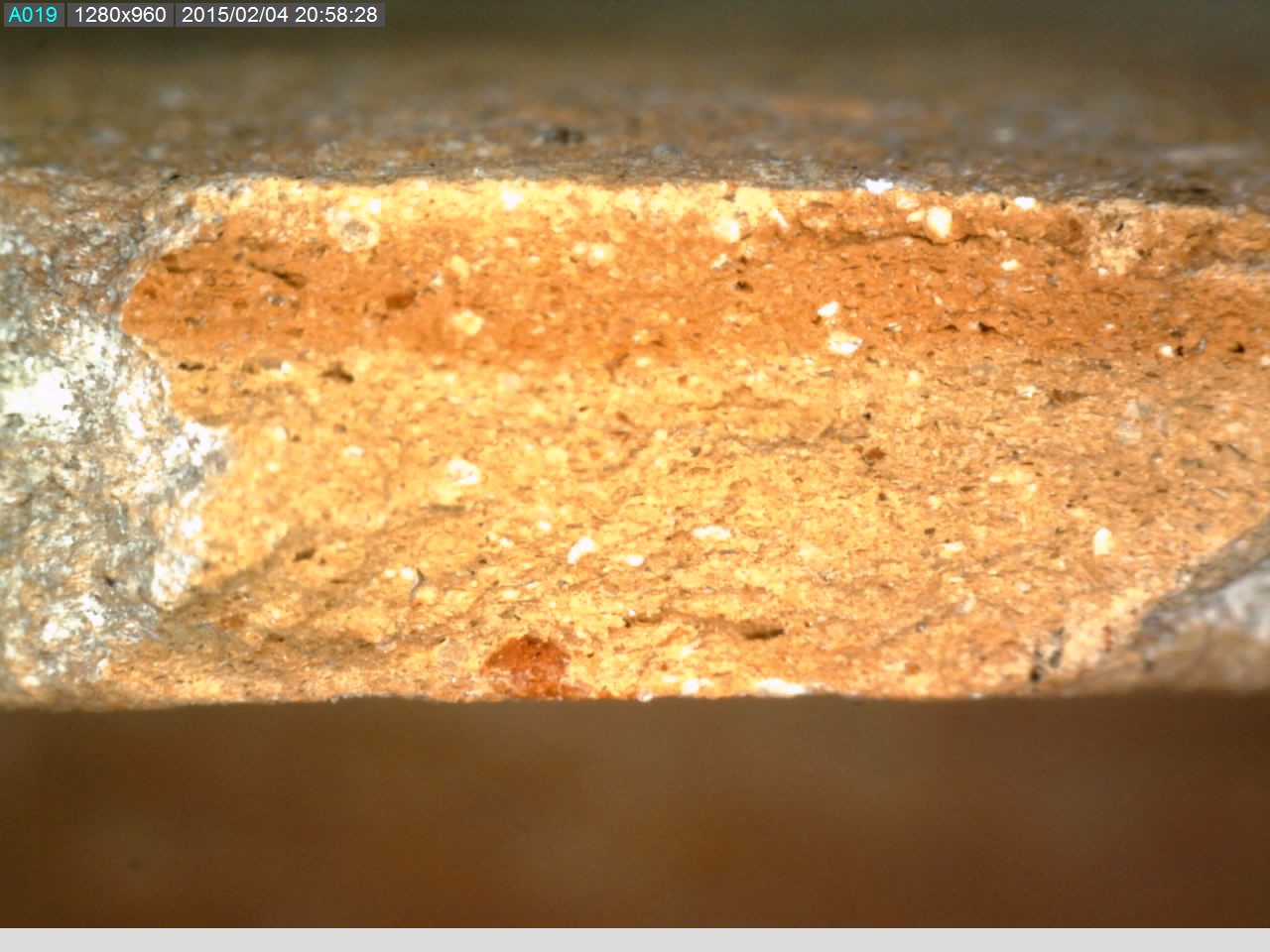

Supplement: Supplementary file 3 — Supplementary material [file mmc3.zip › Appendix A/HTN 22/HTN 22-50m-2.jpg]

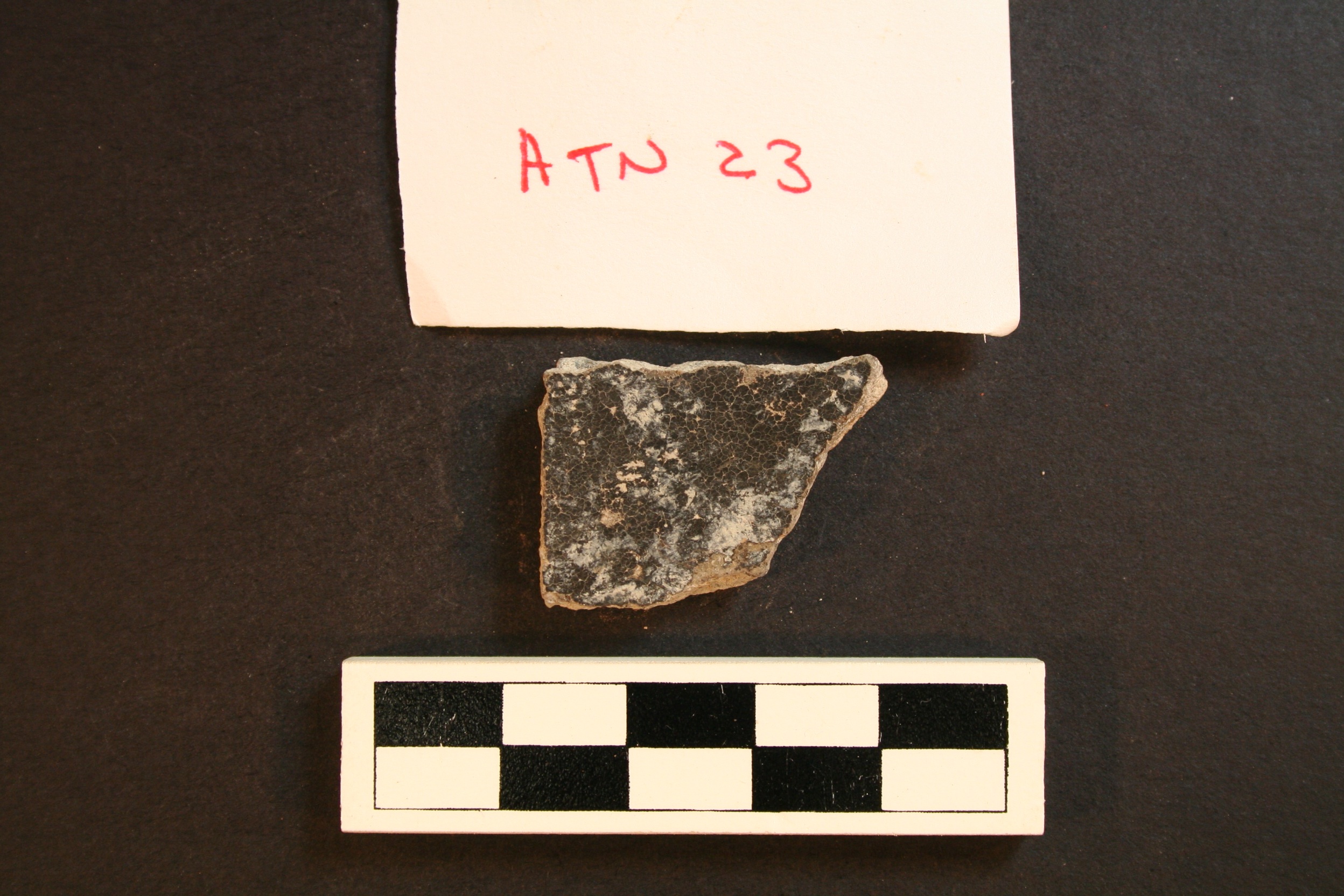

Supplement: Supplementary file 3 — Supplementary material [file mmc3.zip › Appendix A/HTN 23/23a.JPG]

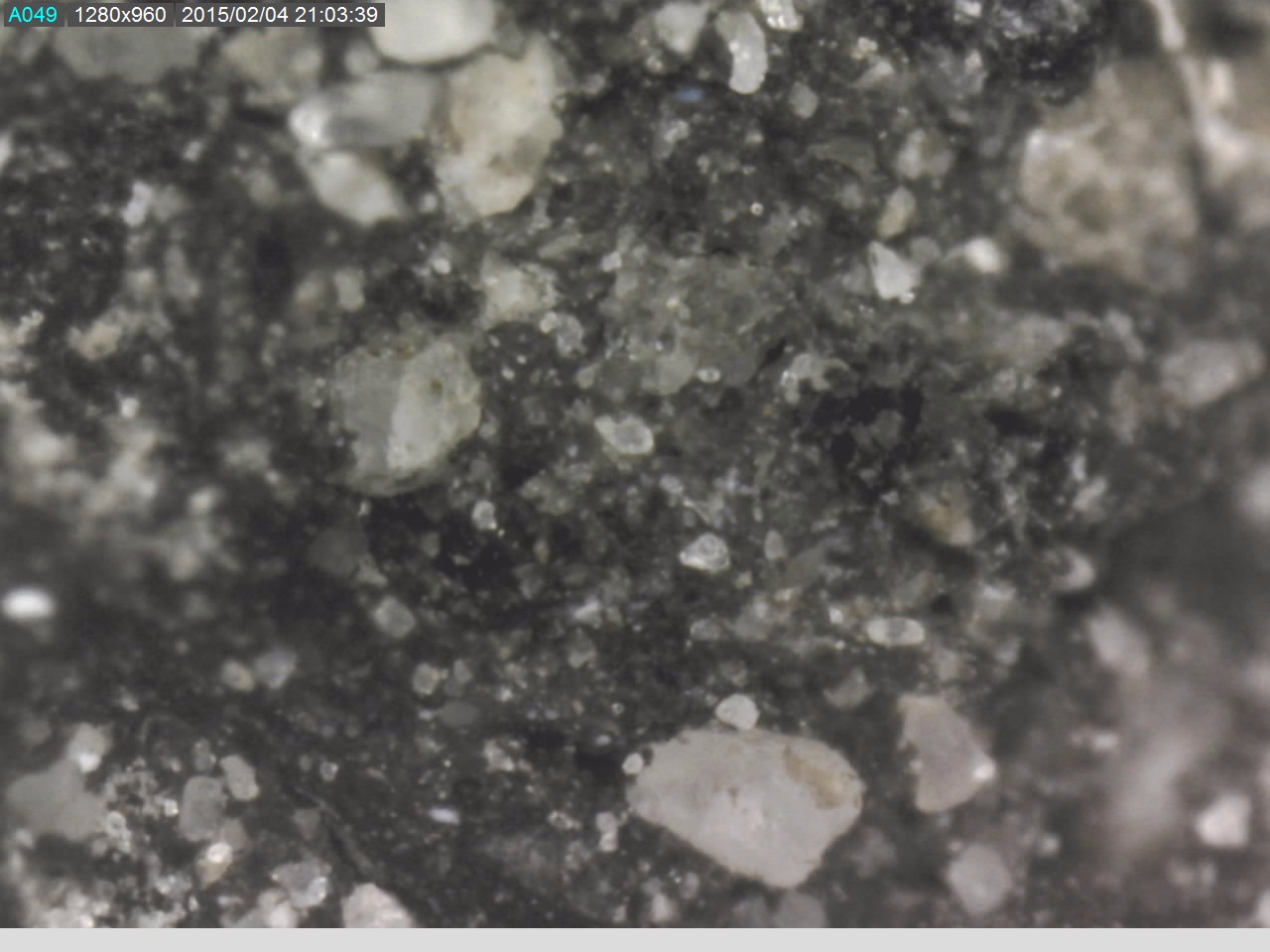

Supplement: Supplementary file 3 — Supplementary material [file mmc3.zip › Appendix A/HTN 23/HTN 23-250m-1.jpg]

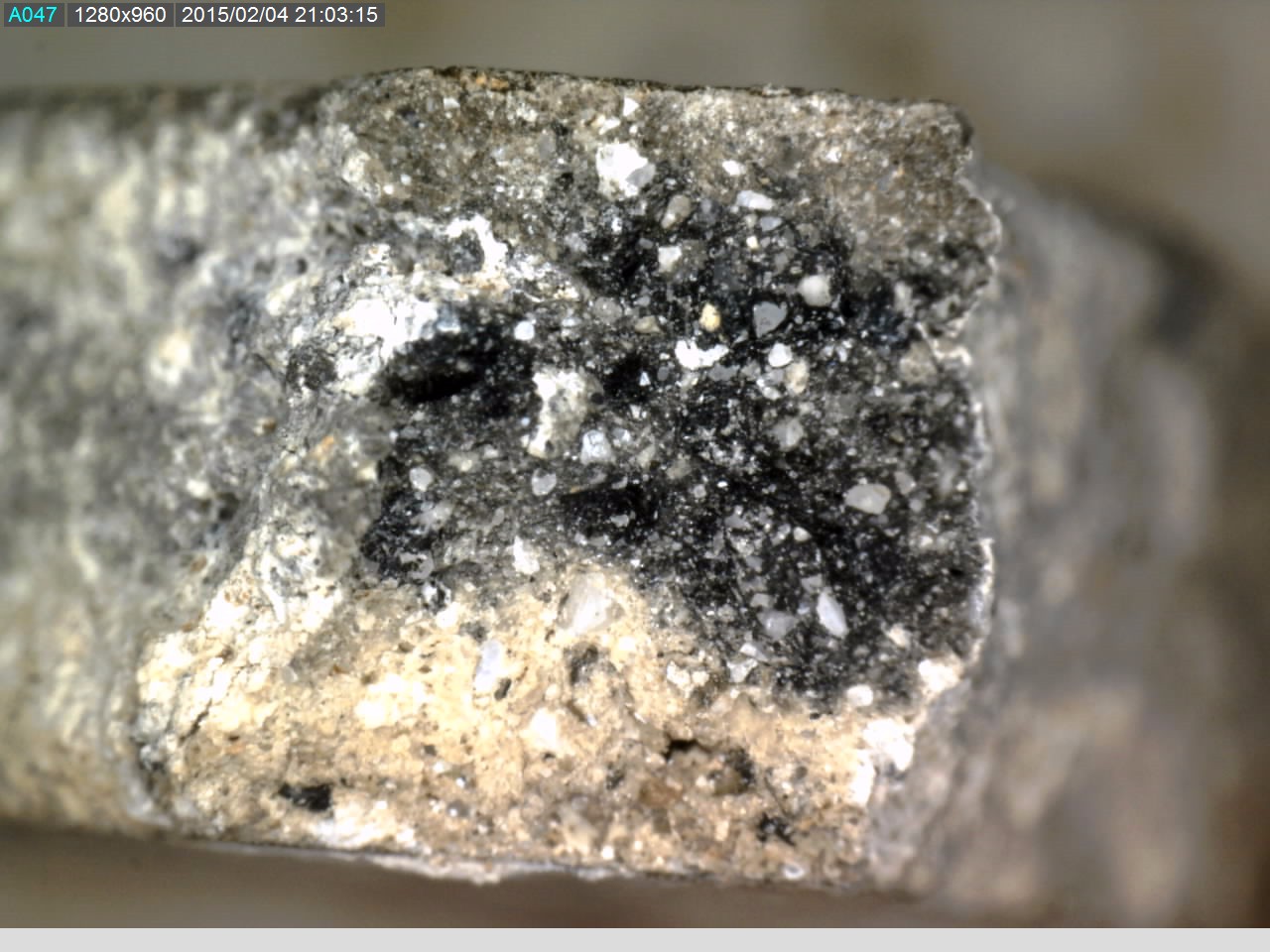

Supplement: Supplementary file 3 — Supplementary material [file mmc3.zip › Appendix A/HTN 23/HTN 23-50m-8.jpg]

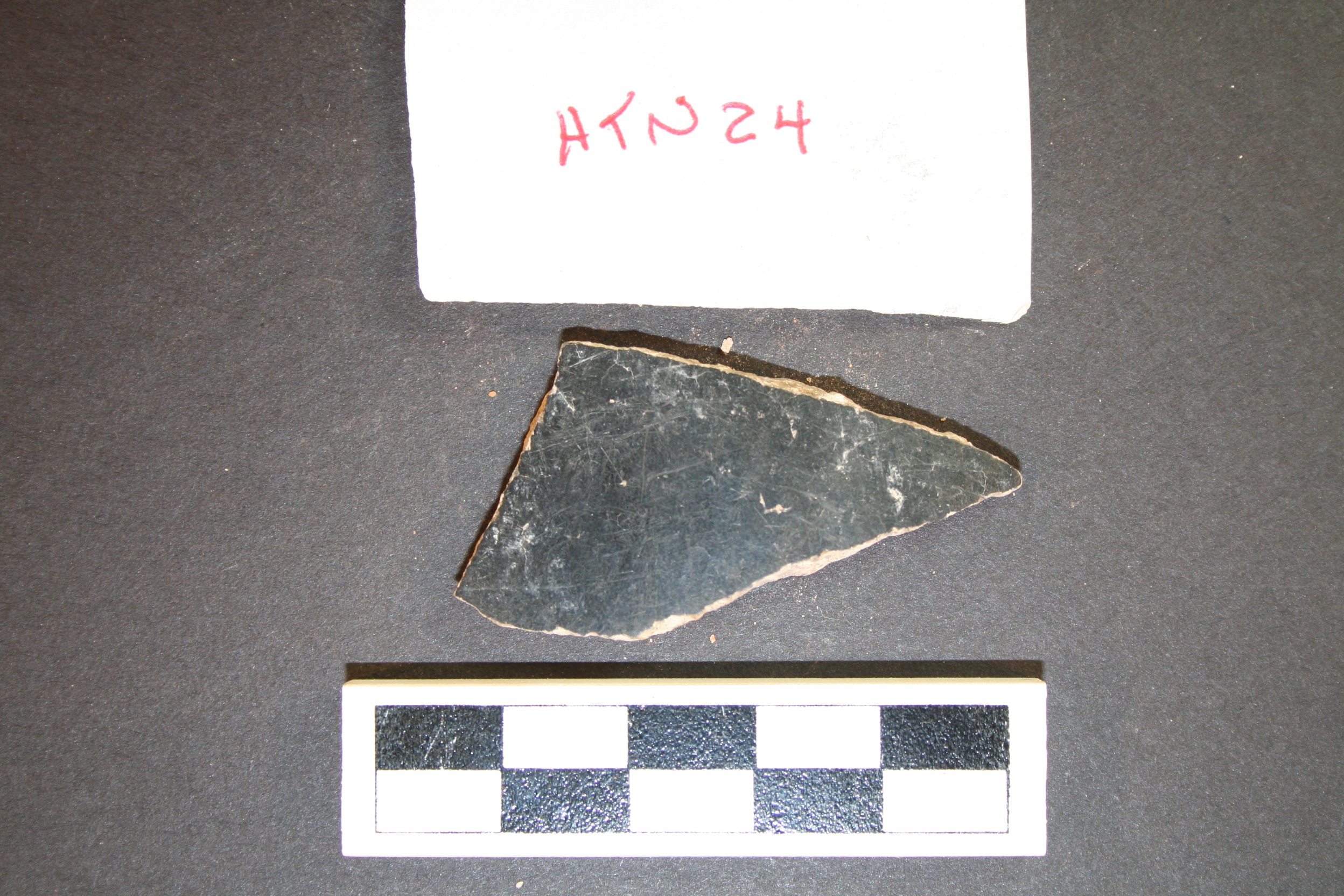

Supplement: Supplementary file 3 — Supplementary material [file mmc3.zip › Appendix A/HTN 24/24a.JPG]

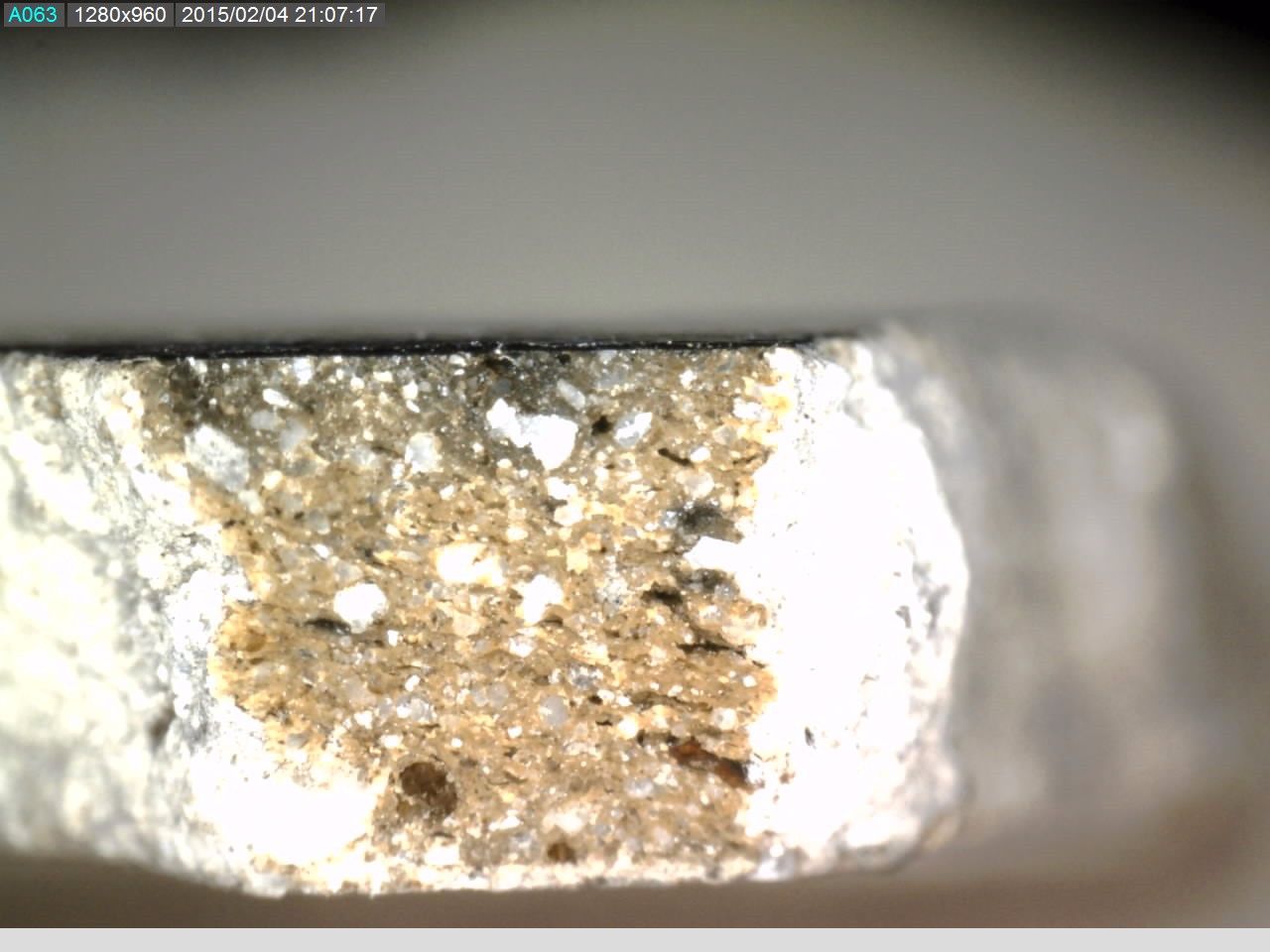

Supplement: Supplementary file 3 — Supplementary material [file mmc3.zip › Appendix A/HTN 24/HTN 24-50m-5.jpg]

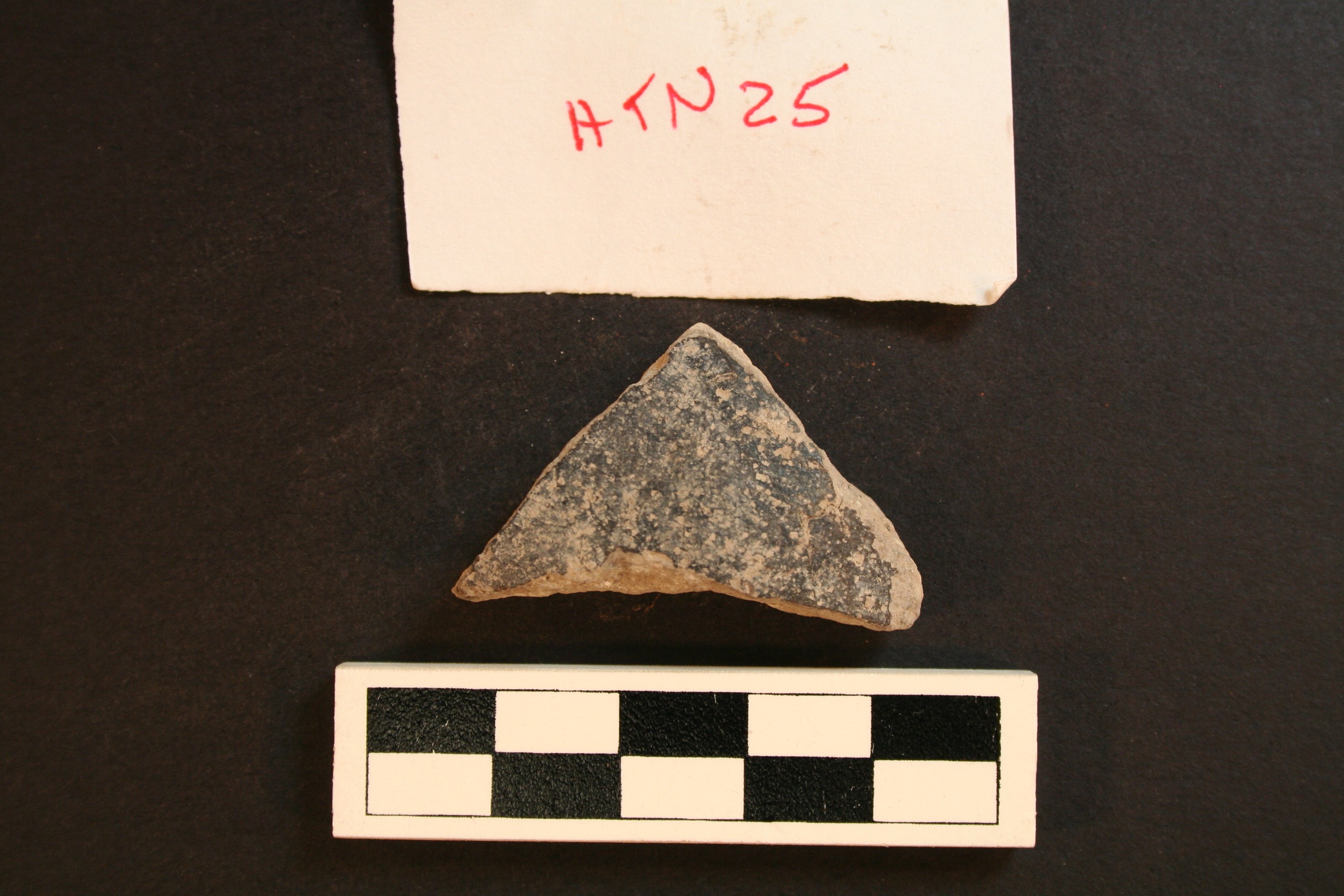

Supplement: Supplementary file 3 — Supplementary material [file mmc3.zip › Appendix A/HTN 25/25a.JPG]

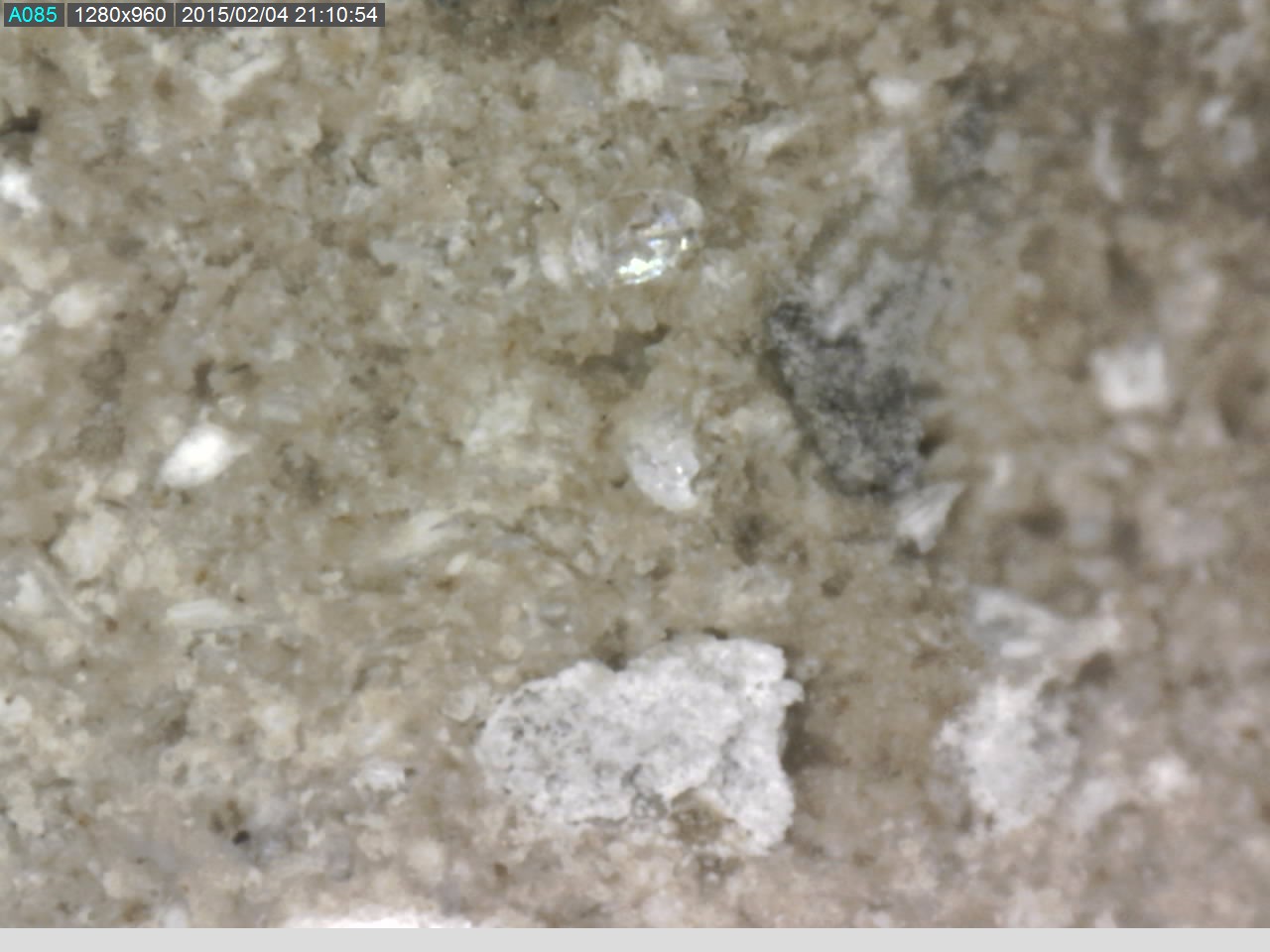

Supplement: Supplementary file 3 — Supplementary material [file mmc3.zip › Appendix A/HTN 25/HTN 25-250m-7.jpg]

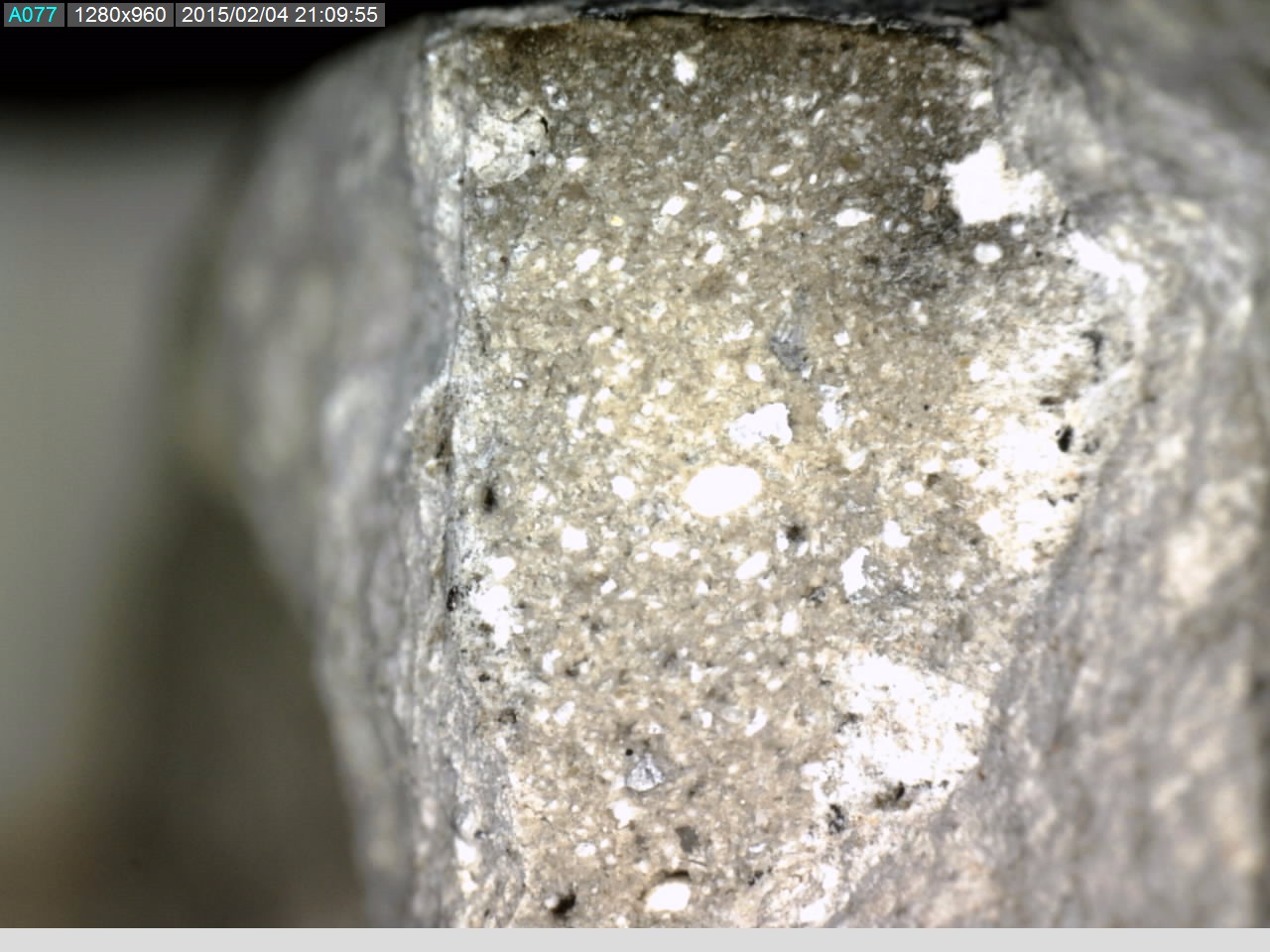

Supplement: Supplementary file 3 — Supplementary material [file mmc3.zip › Appendix A/HTN 25/HTN 25-50m-6.jpg]

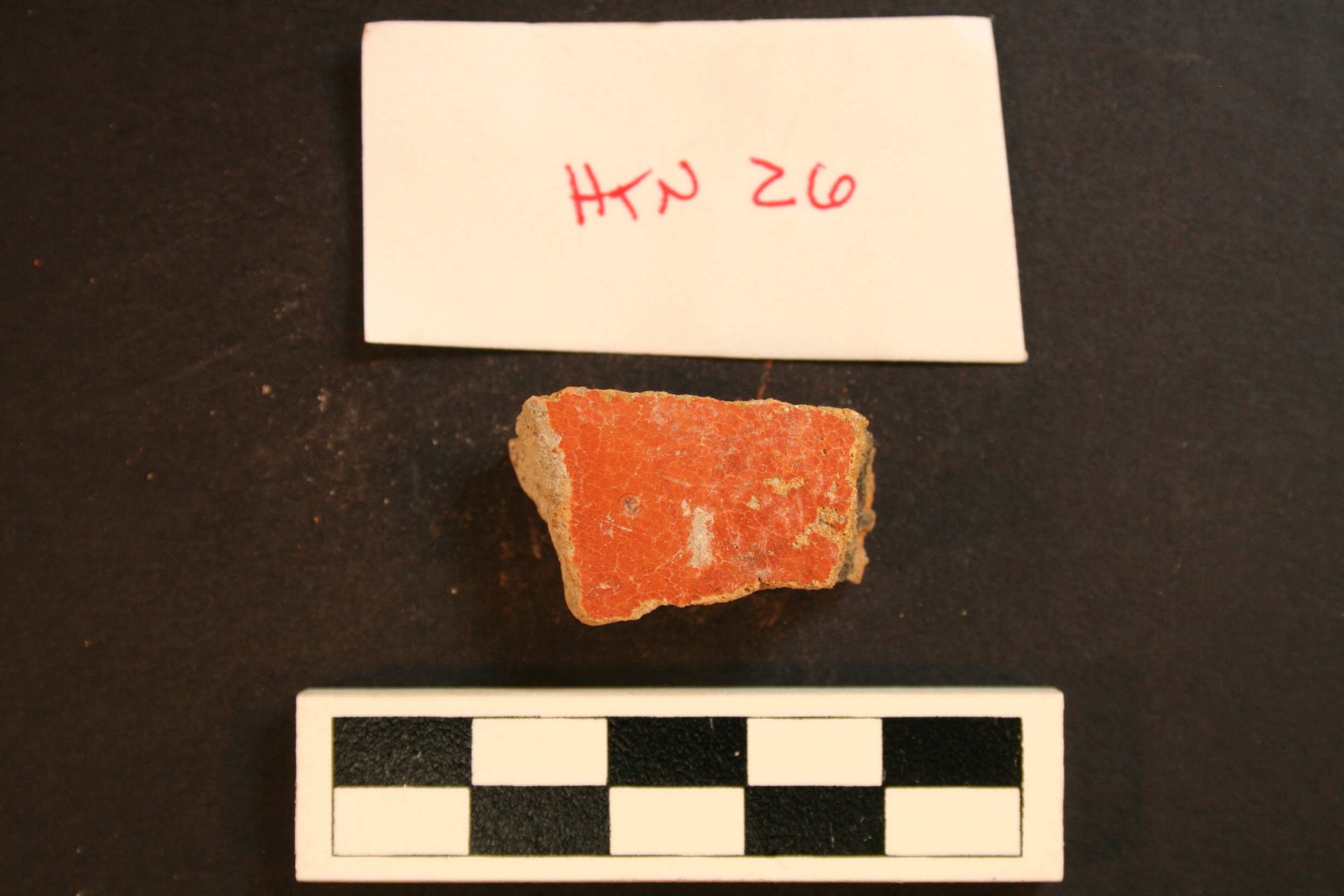

Supplement: Supplementary file 3 — Supplementary material [file mmc3.zip › Appendix A/HTN 26/26a.JPG]

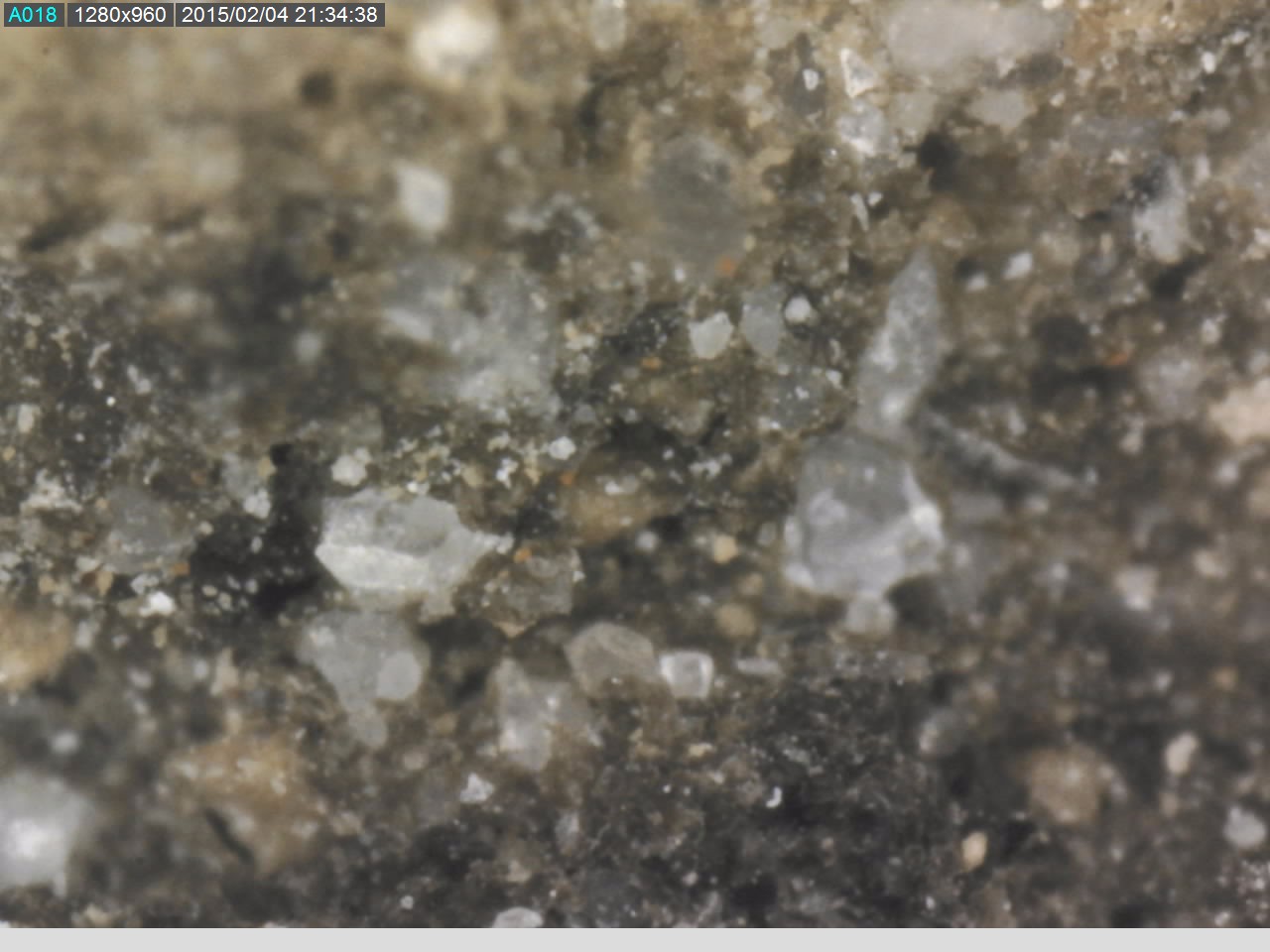

Supplement: Supplementary file 3 — Supplementary material [file mmc3.zip › Appendix A/HTN 26/HTN 26-250m-8.jpg]

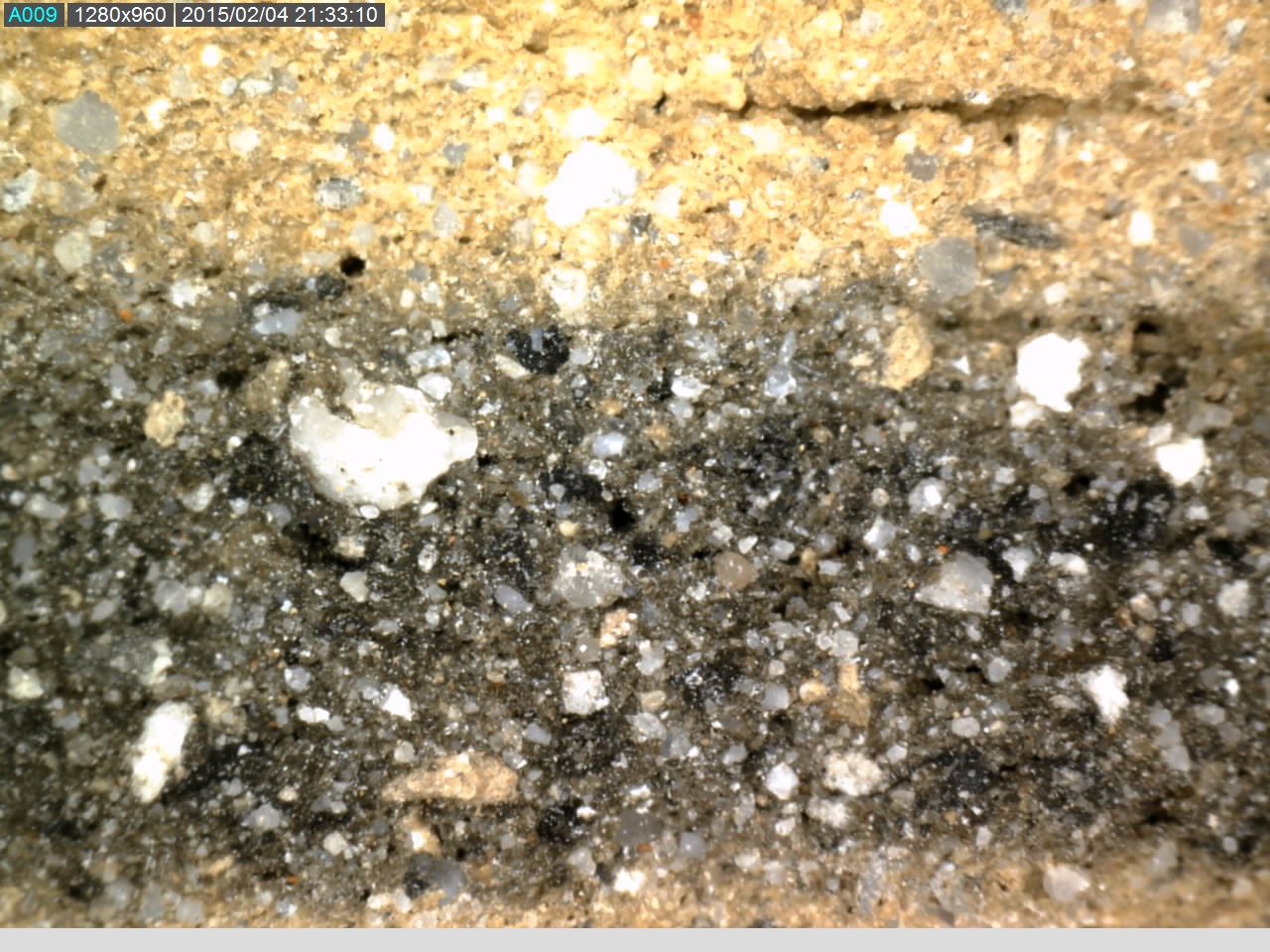

Supplement: Supplementary file 3 — Supplementary material [file mmc3.zip › Appendix A/HTN 26/HTN 26-50m-8.jpg]

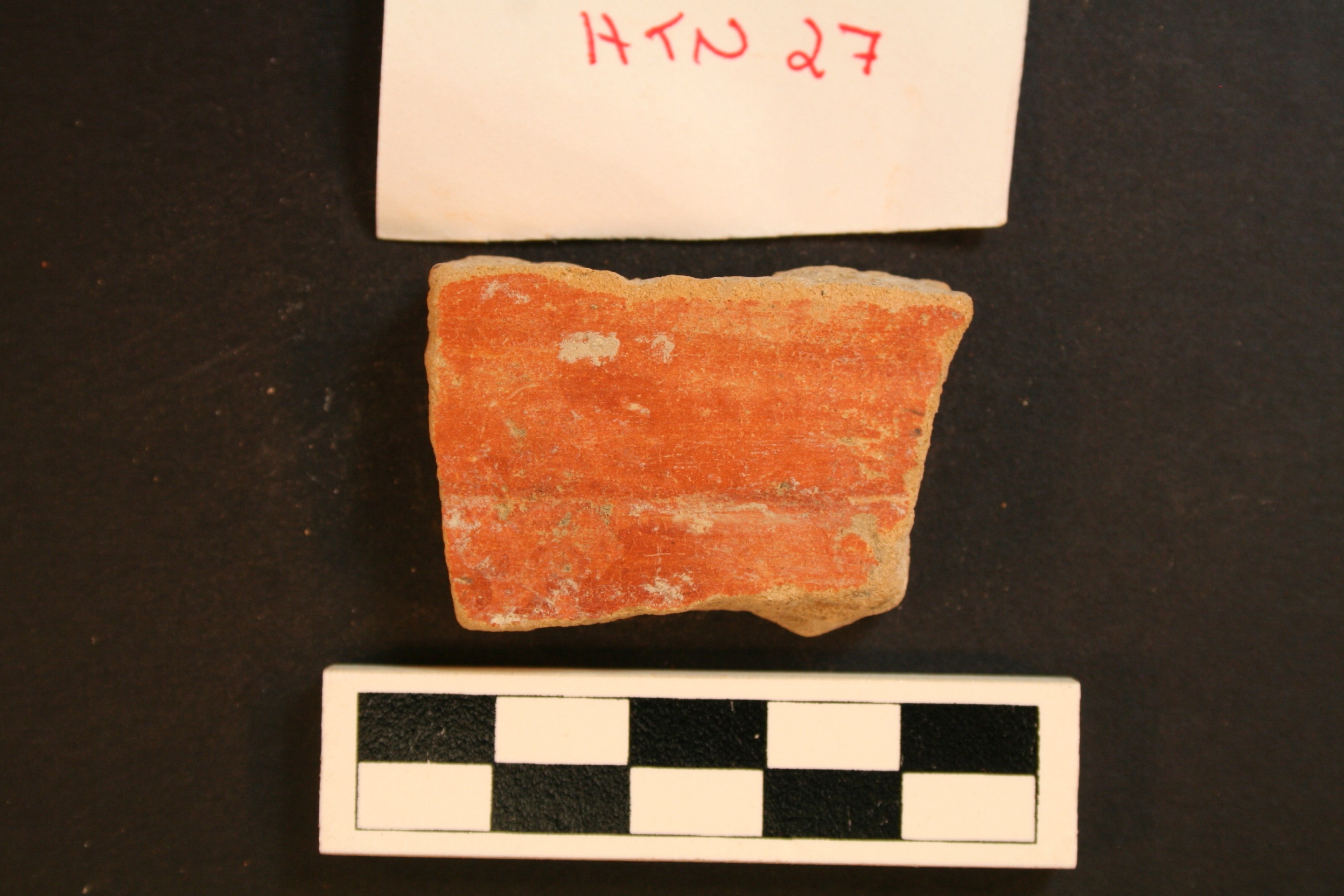

Supplement: Supplementary file 3 — Supplementary material [file mmc3.zip › Appendix A/HTN 27/27a.JPG]

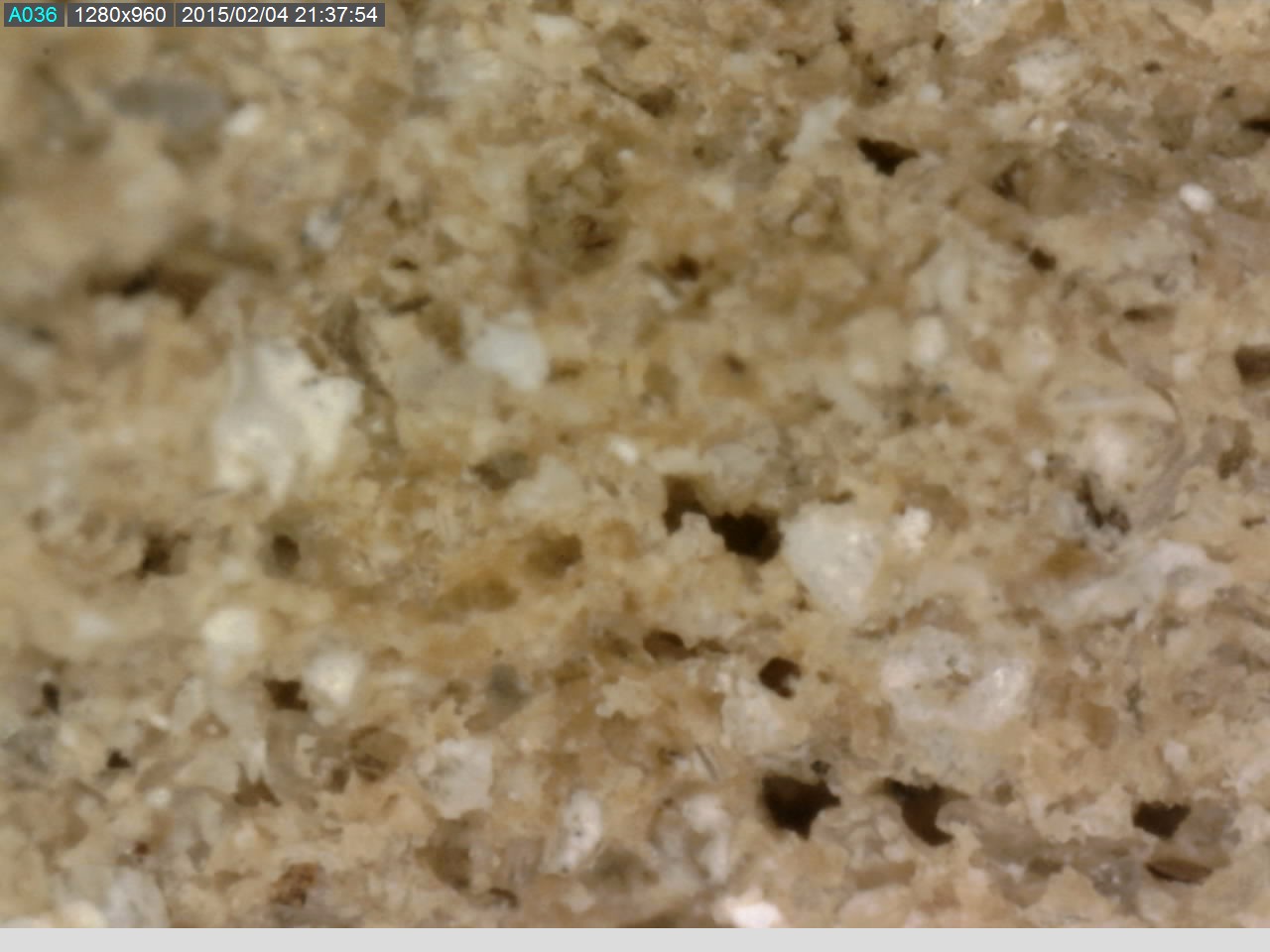

Supplement: Supplementary file 3 — Supplementary material [file mmc3.zip › Appendix A/HTN 27/HTN 27-250m-7.jpg]

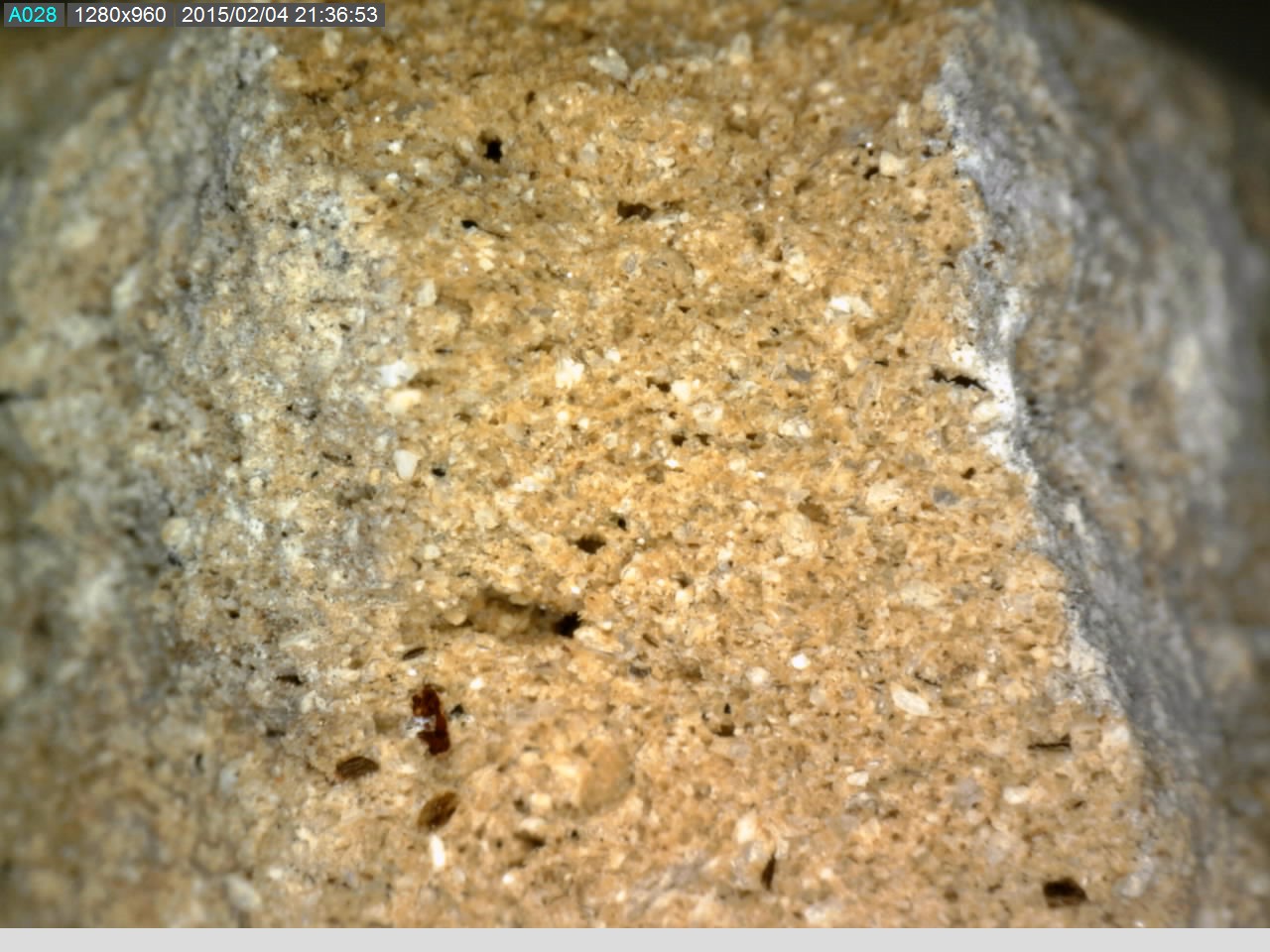

Supplement: Supplementary file 3 — Supplementary material [file mmc3.zip › Appendix A/HTN 27/HTN 27-50m-5.jpg]

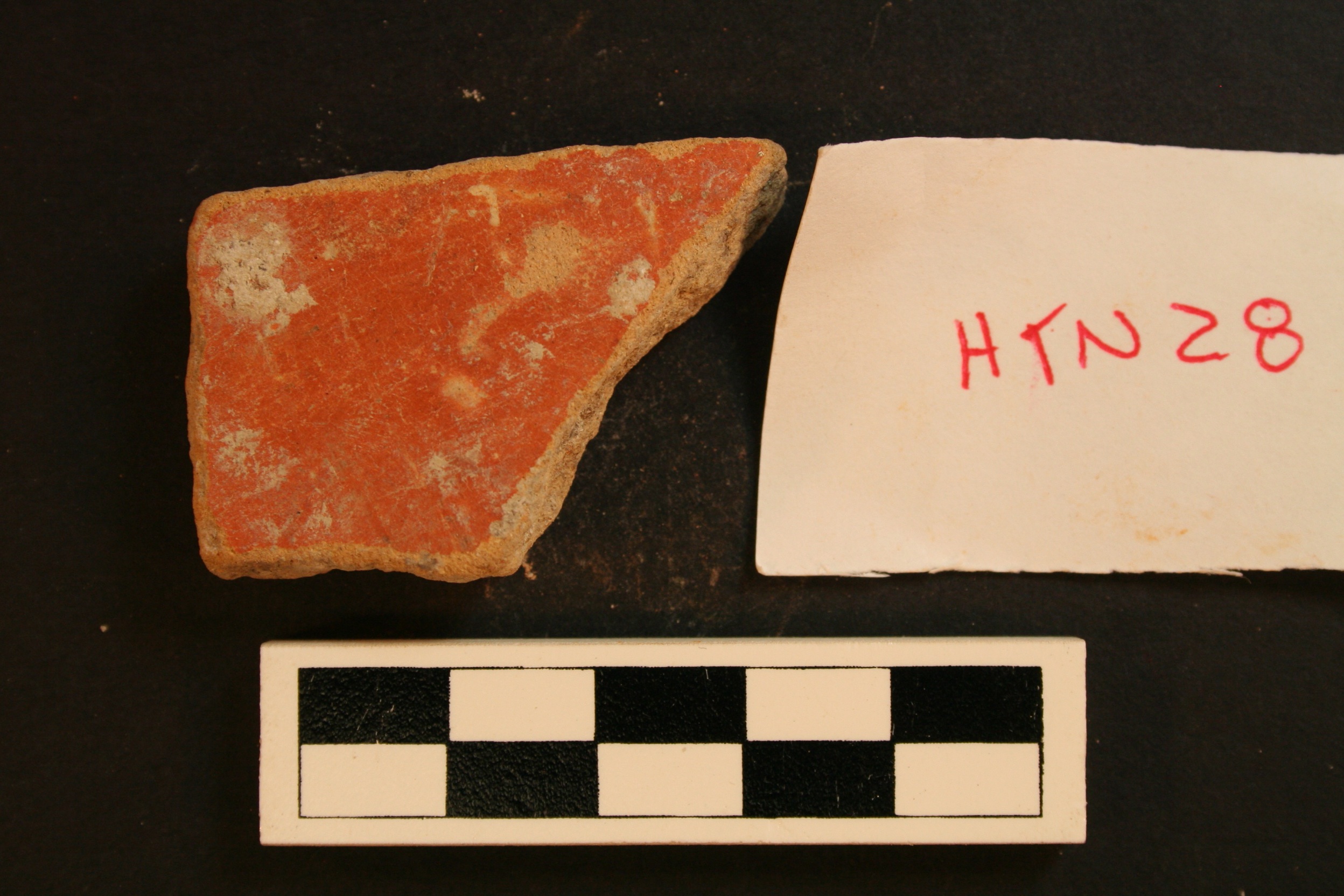

Supplement: Supplementary file 3 — Supplementary material [file mmc3.zip › Appendix A/HTN 28/28a.JPG]

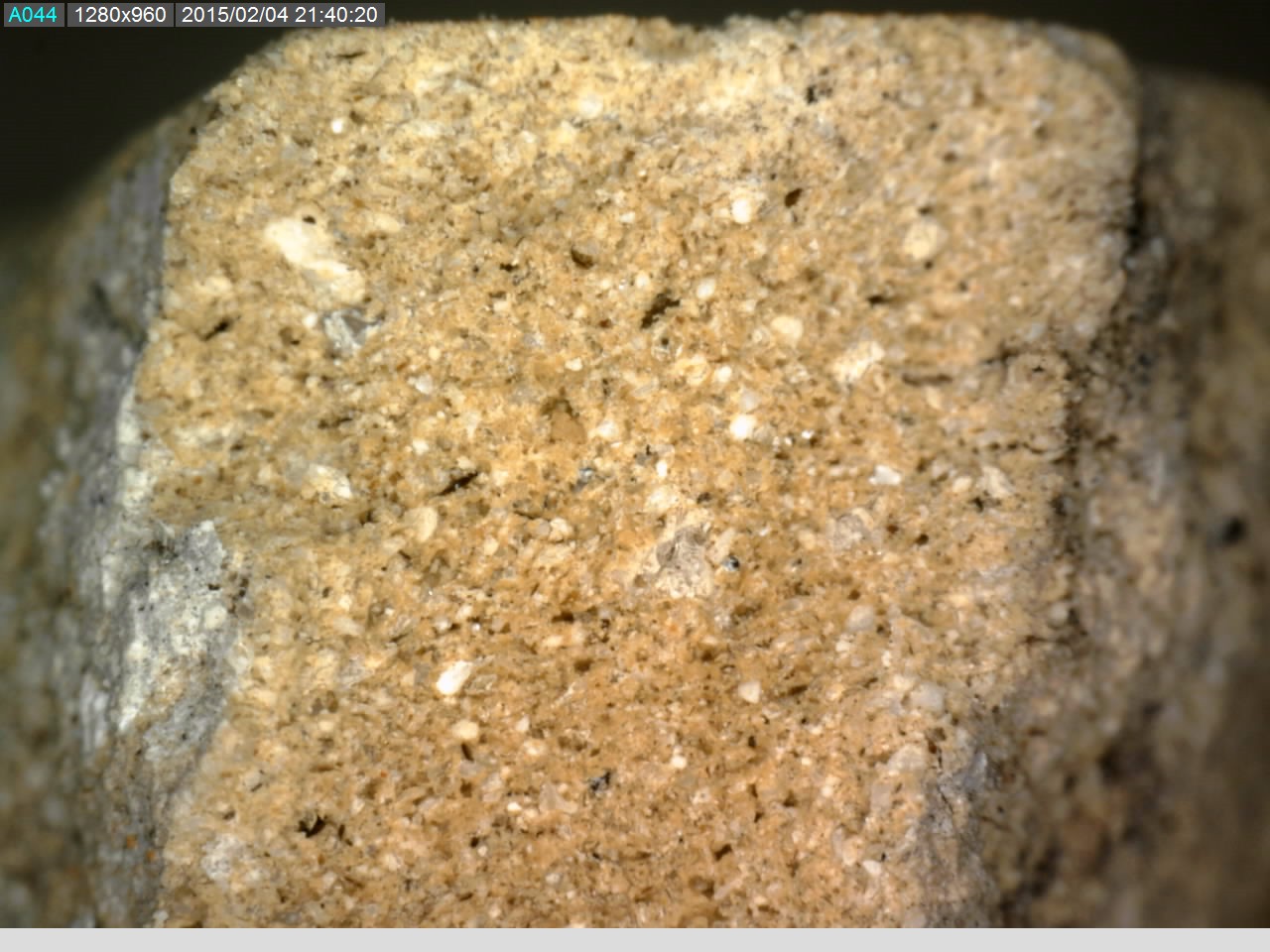

Supplement: Supplementary file 3 — Supplementary material [file mmc3.zip › Appendix A/HTN 28/HTN 28-50m-1.jpg]

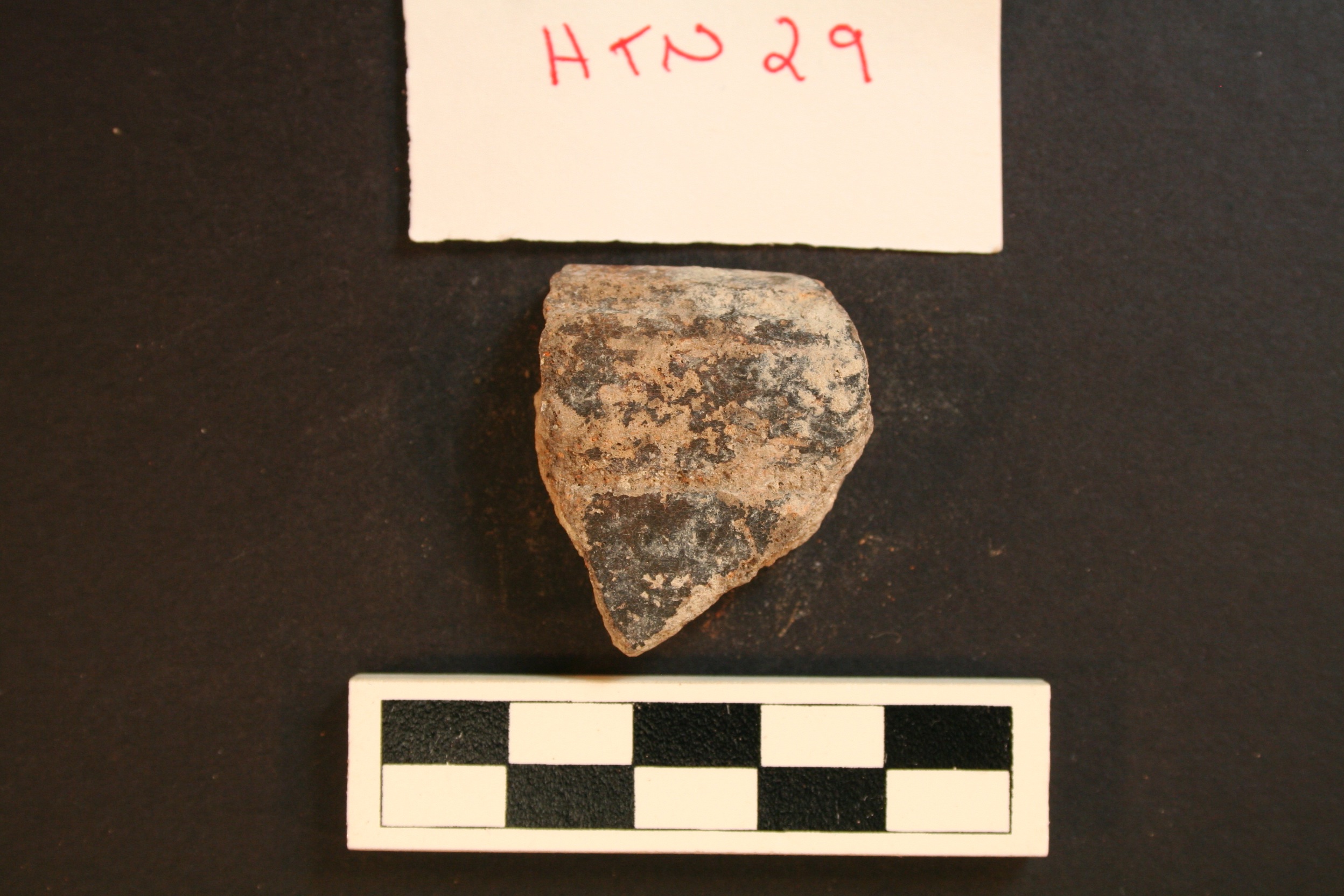

Supplement: Supplementary file 3 — Supplementary material [file mmc3.zip › Appendix A/HTN 29/29a.JPG]

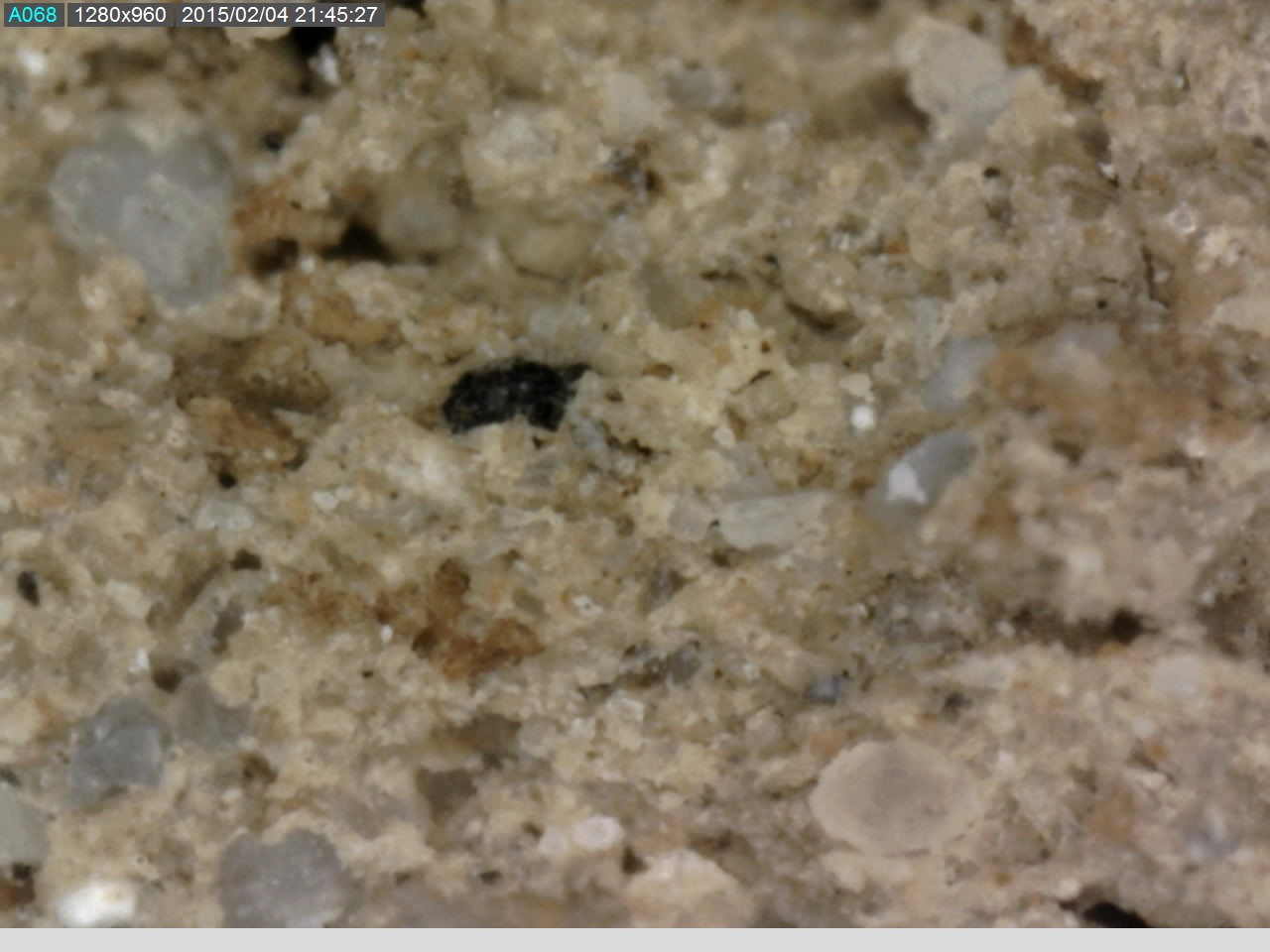

Supplement: Supplementary file 3 — Supplementary material [file mmc3.zip › Appendix A/HTN 29/HTN 29-250m-2.jpg]

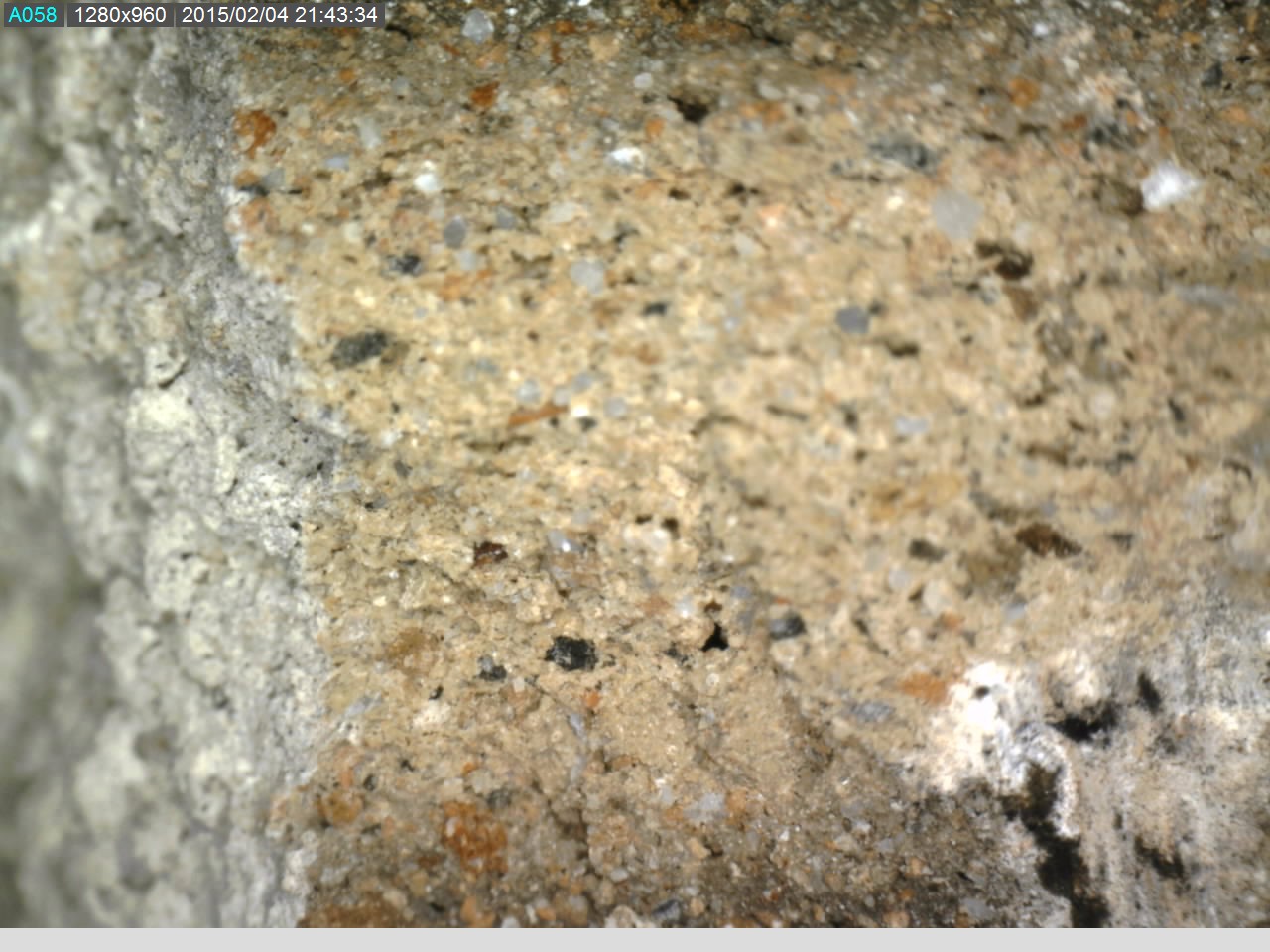

Supplement: Supplementary file 3 — Supplementary material [file mmc3.zip › Appendix A/HTN 29/HTN 29-50m-0.jpg]

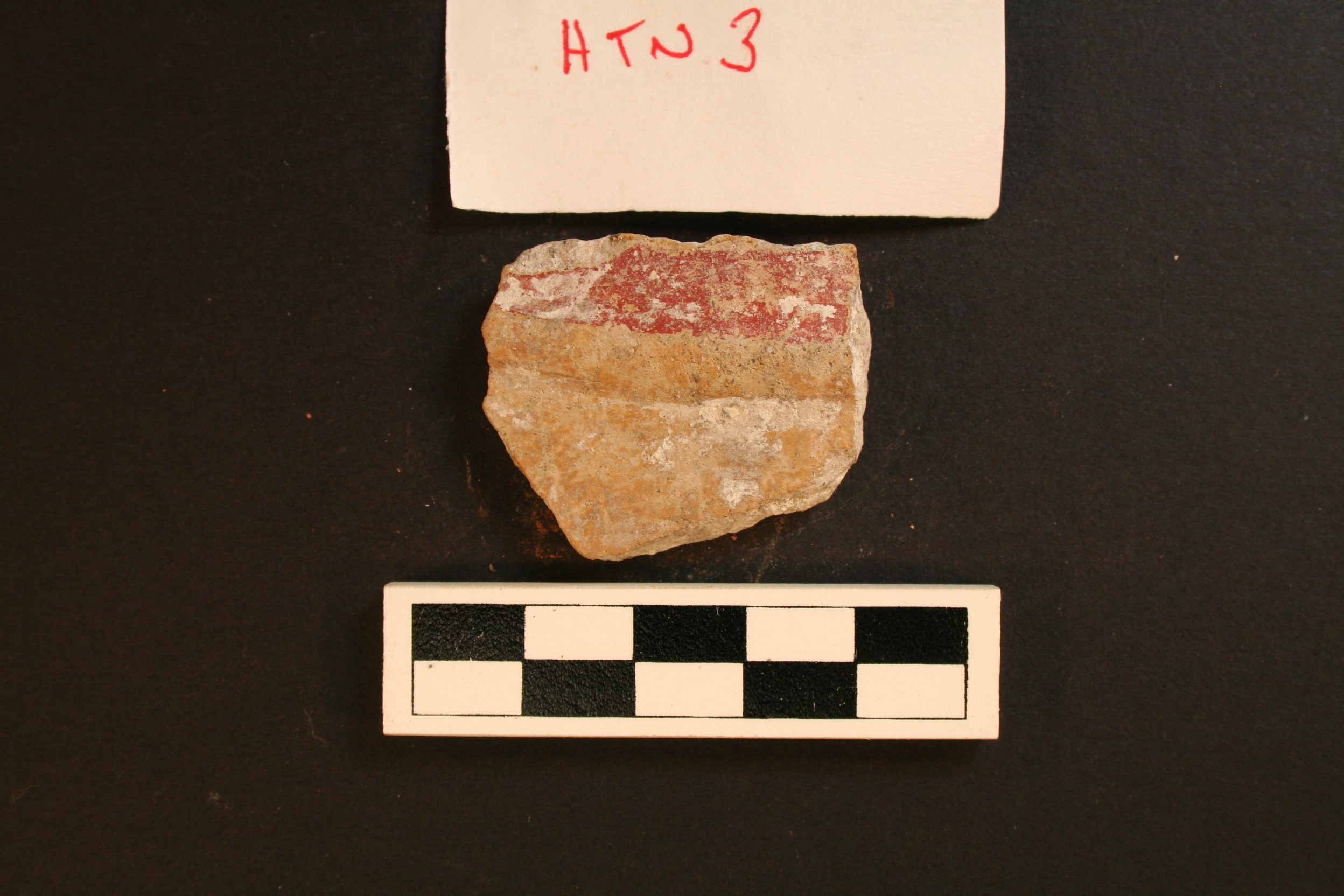

Supplement: Supplementary file 3 — Supplementary material [file mmc3.zip › Appendix A/HTN 3/3a.JPG]

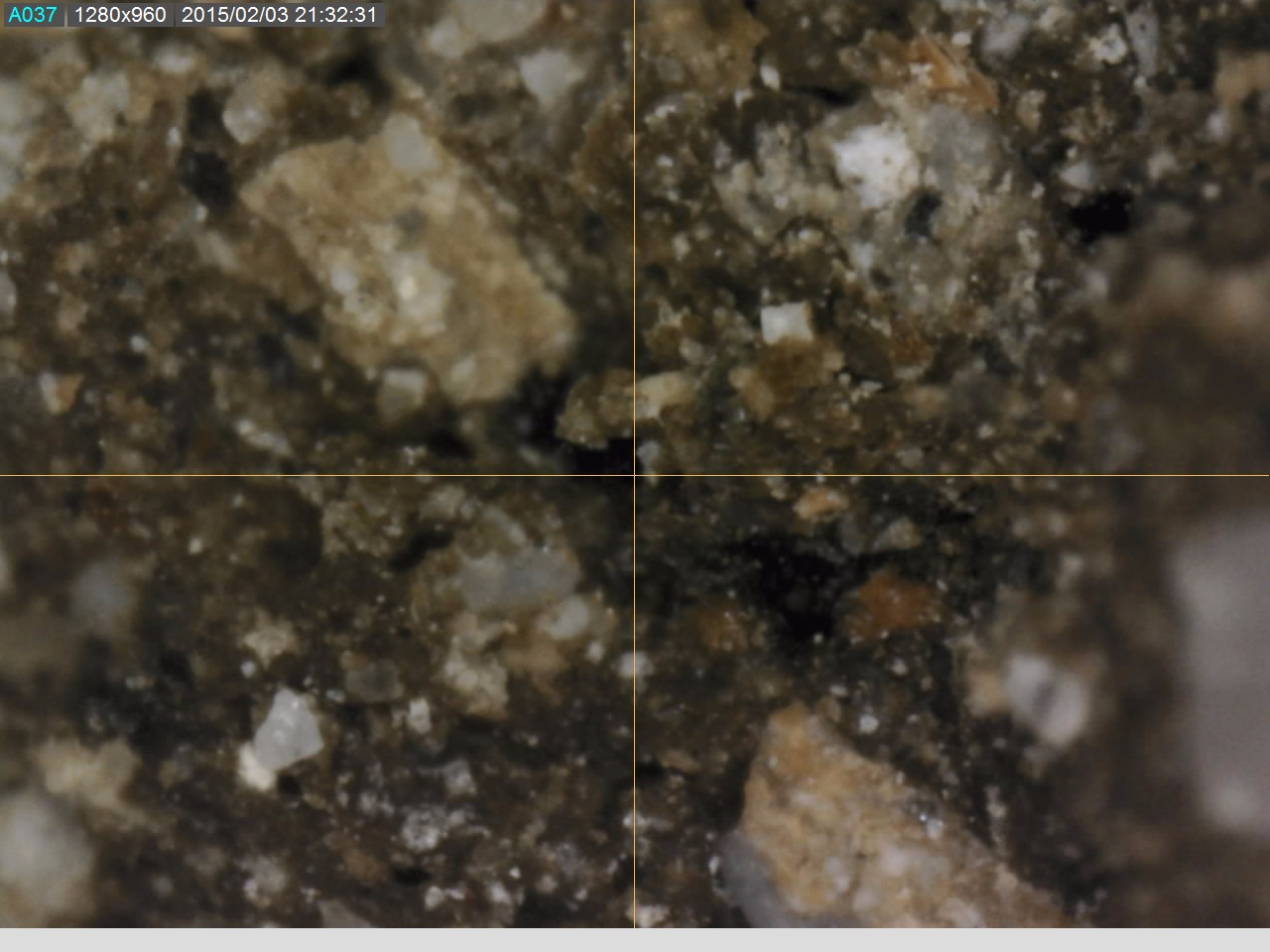

Supplement: Supplementary file 3 — Supplementary material [file mmc3.zip › Appendix A/HTN 3/HTN 3-250m-1.jpg]

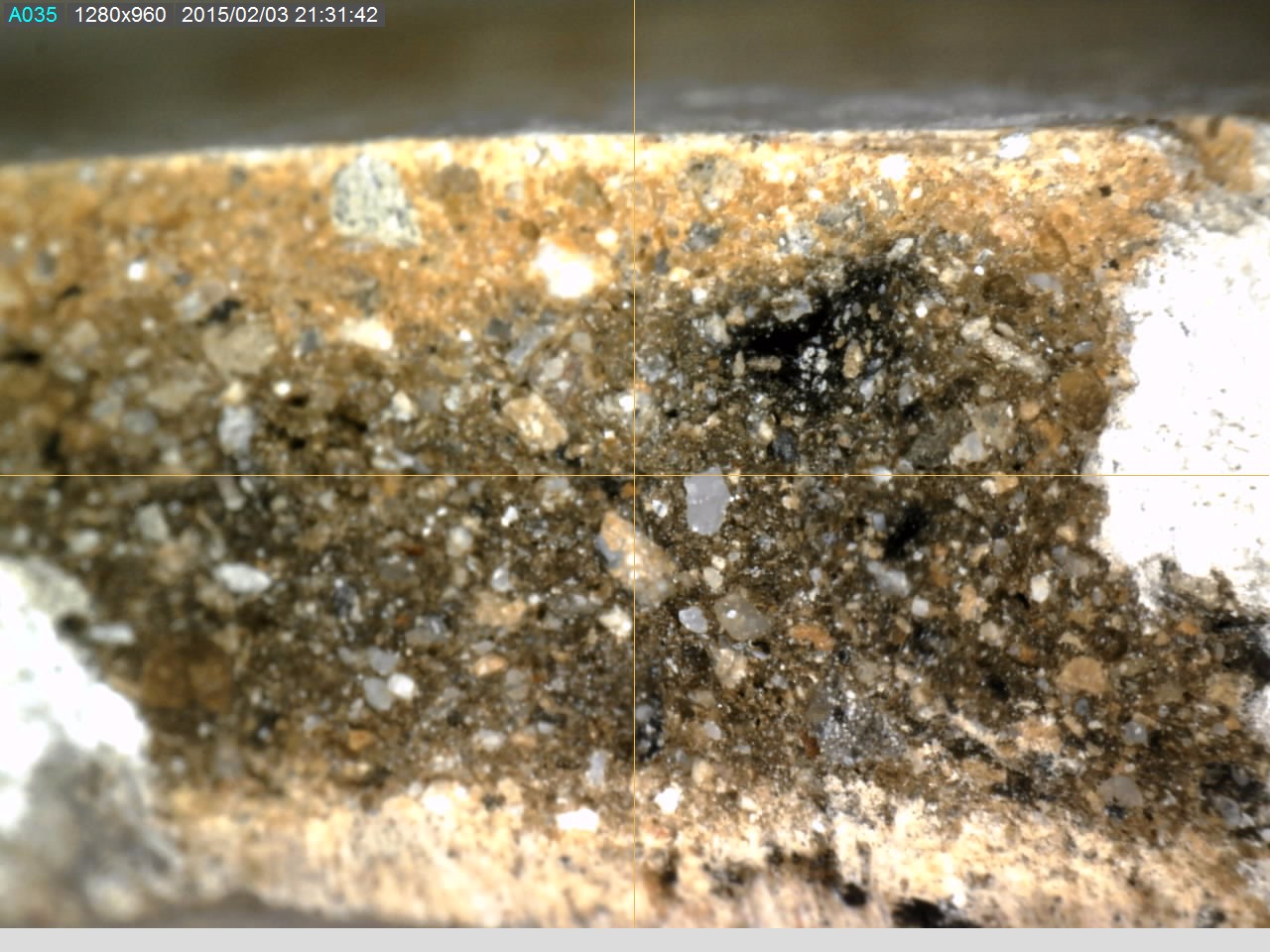

Supplement: Supplementary file 3 — Supplementary material [file mmc3.zip › Appendix A/HTN 3/HTN 3-50m-4.jpg]

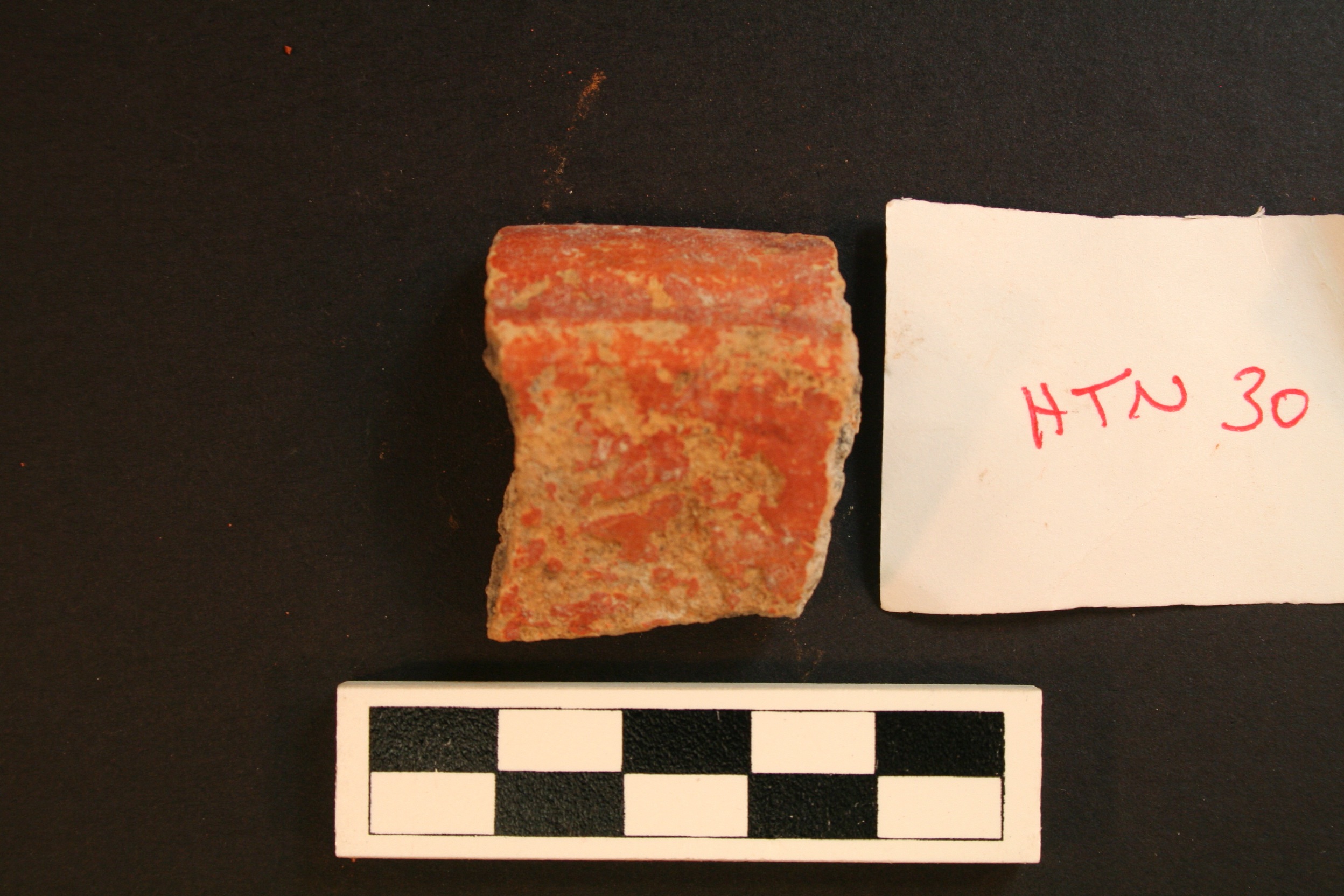

Supplement: Supplementary file 3 — Supplementary material [file mmc3.zip › Appendix A/HTN 30/30a.JPG]

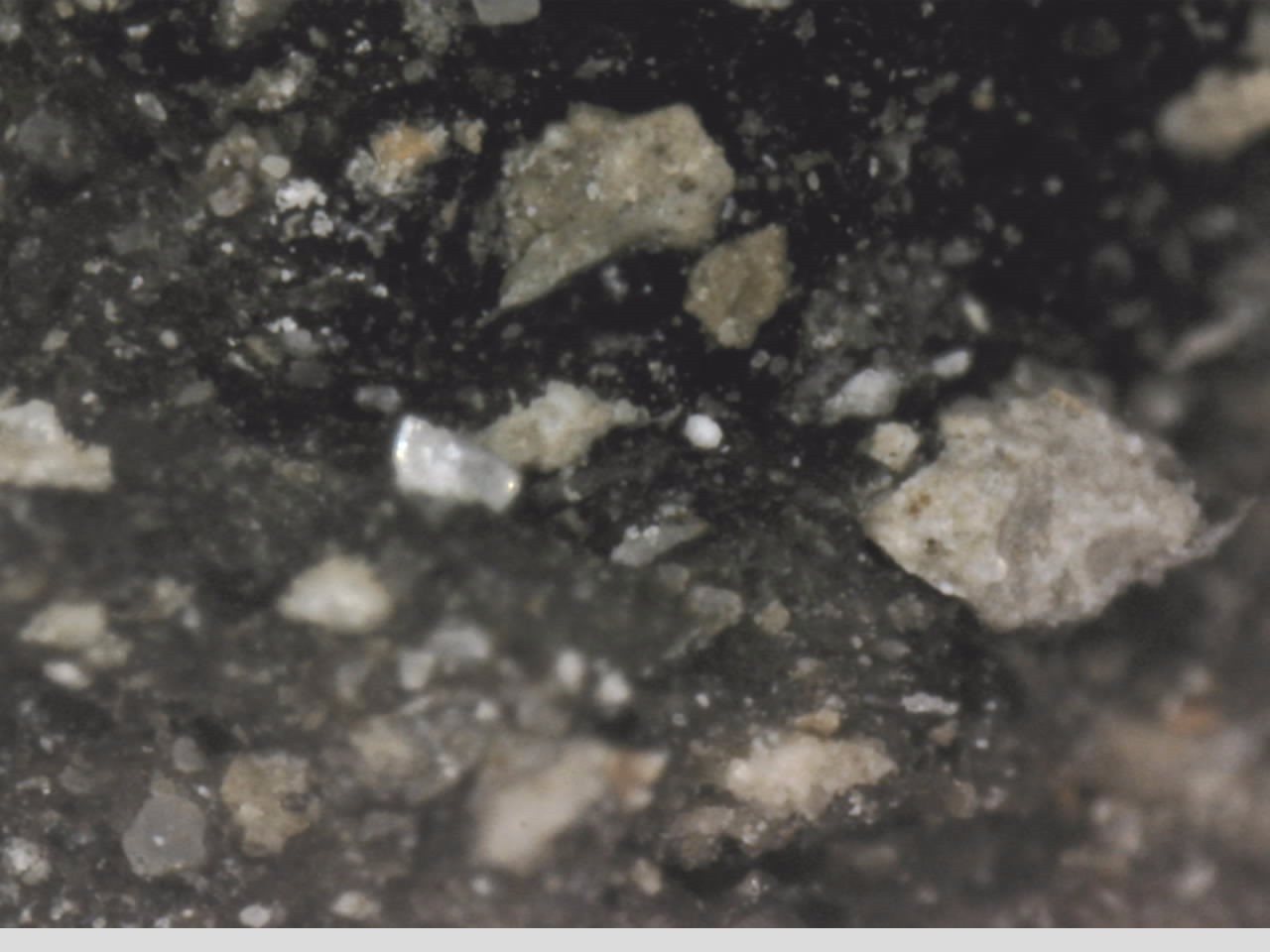

Supplement: Supplementary file 3 — Supplementary material [file mmc3.zip › Appendix A/HTN 30/HTN 30-250m-9.jpg]

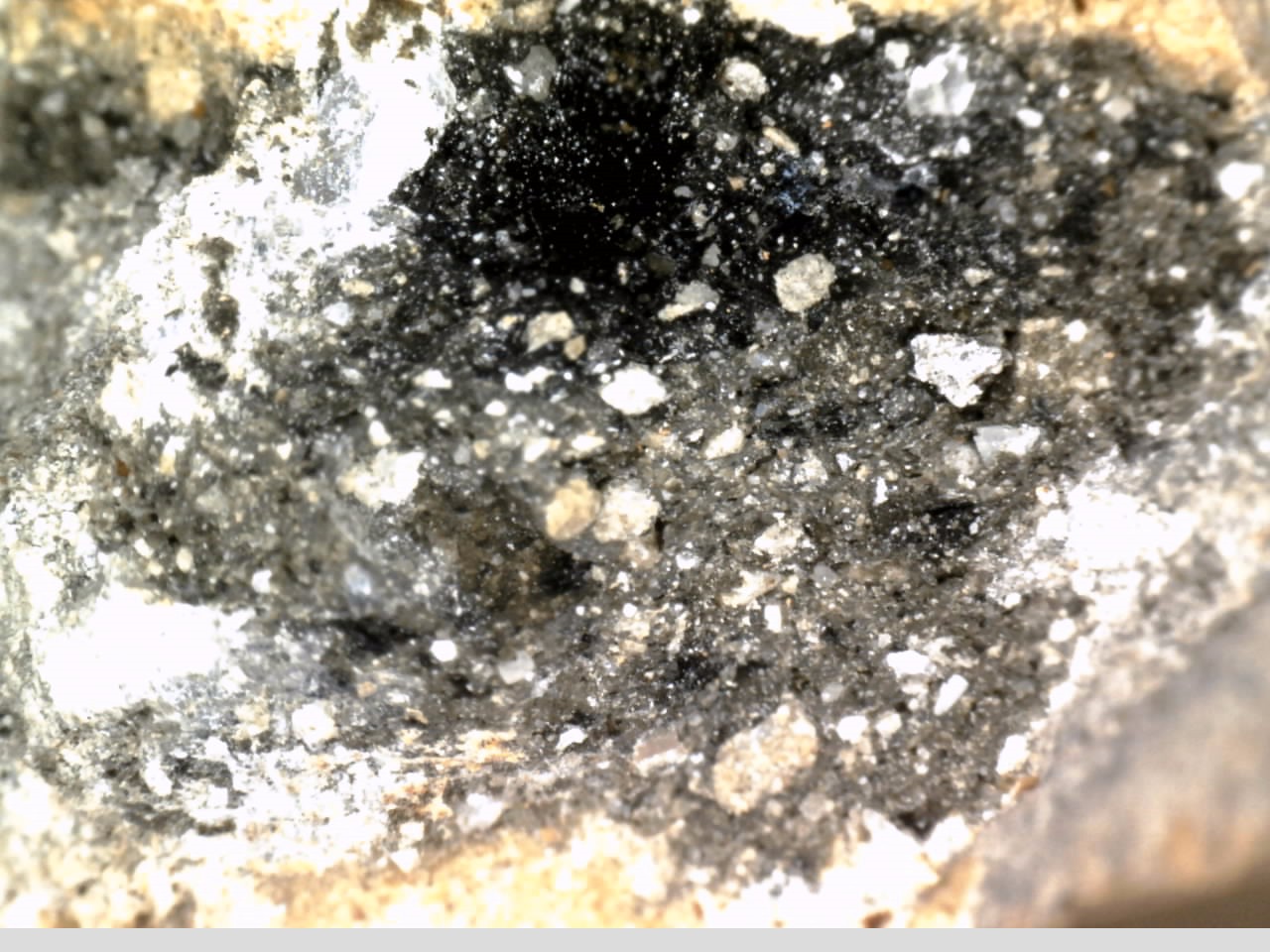

Supplement: Supplementary file 3 — Supplementary material [file mmc3.zip › Appendix A/HTN 30/HTN 30-50m-6.jpg]

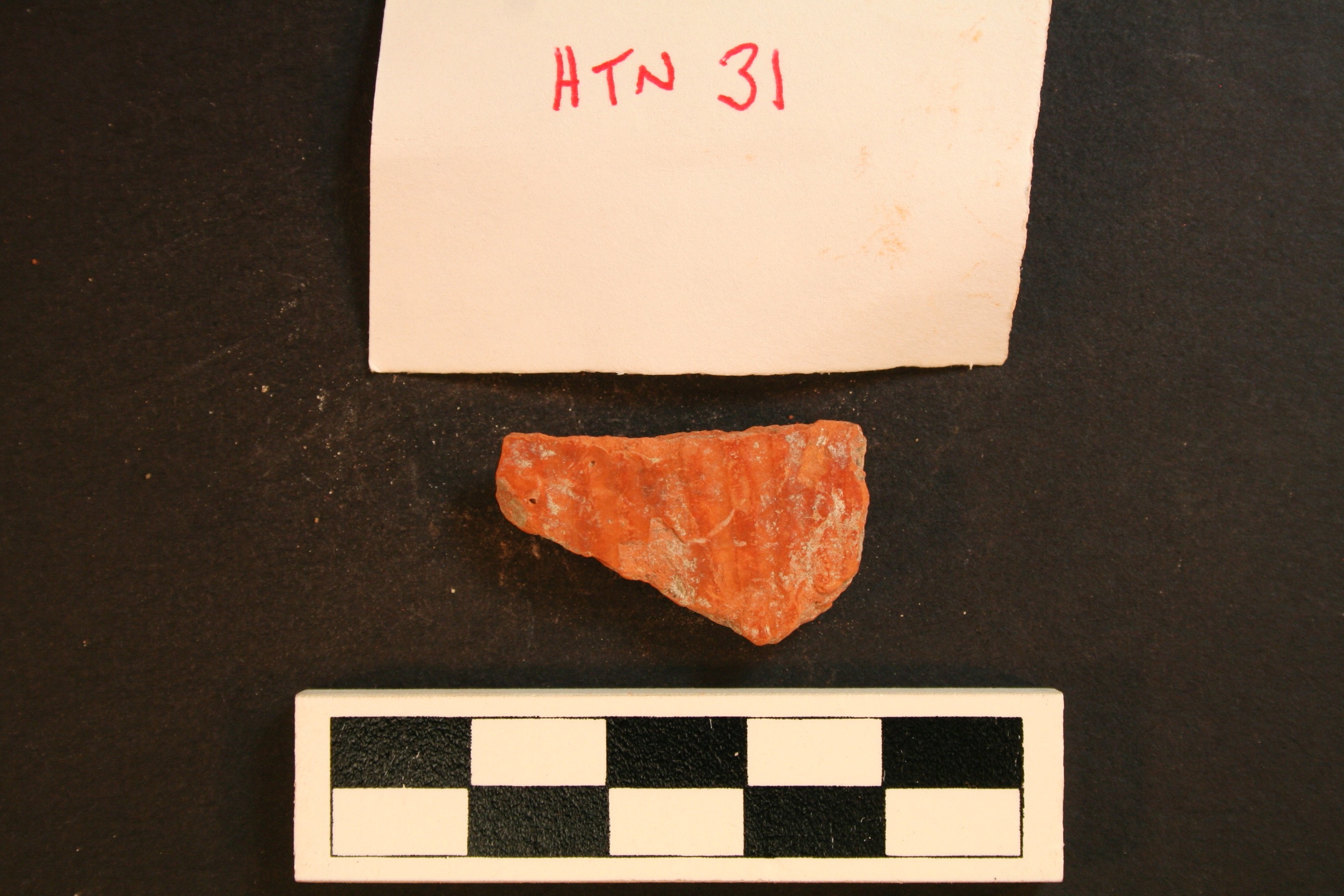

Supplement: Supplementary file 3 — Supplementary material [file mmc3.zip › Appendix A/HTN 31/31a.JPG]

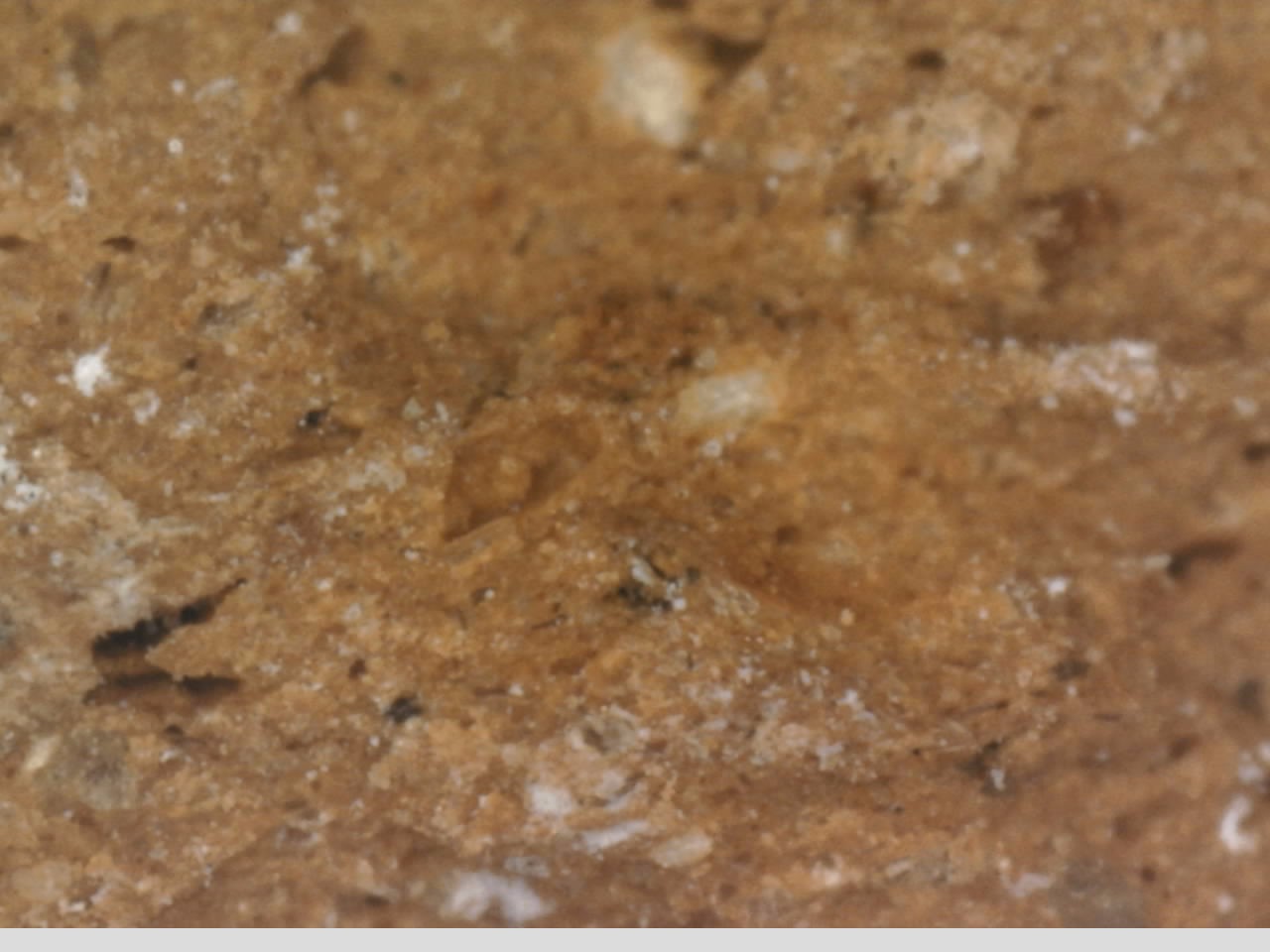

Supplement: Supplementary file 3 — Supplementary material [file mmc3.zip › Appendix A/HTN 31/HTN 31-250m-4.jpg]

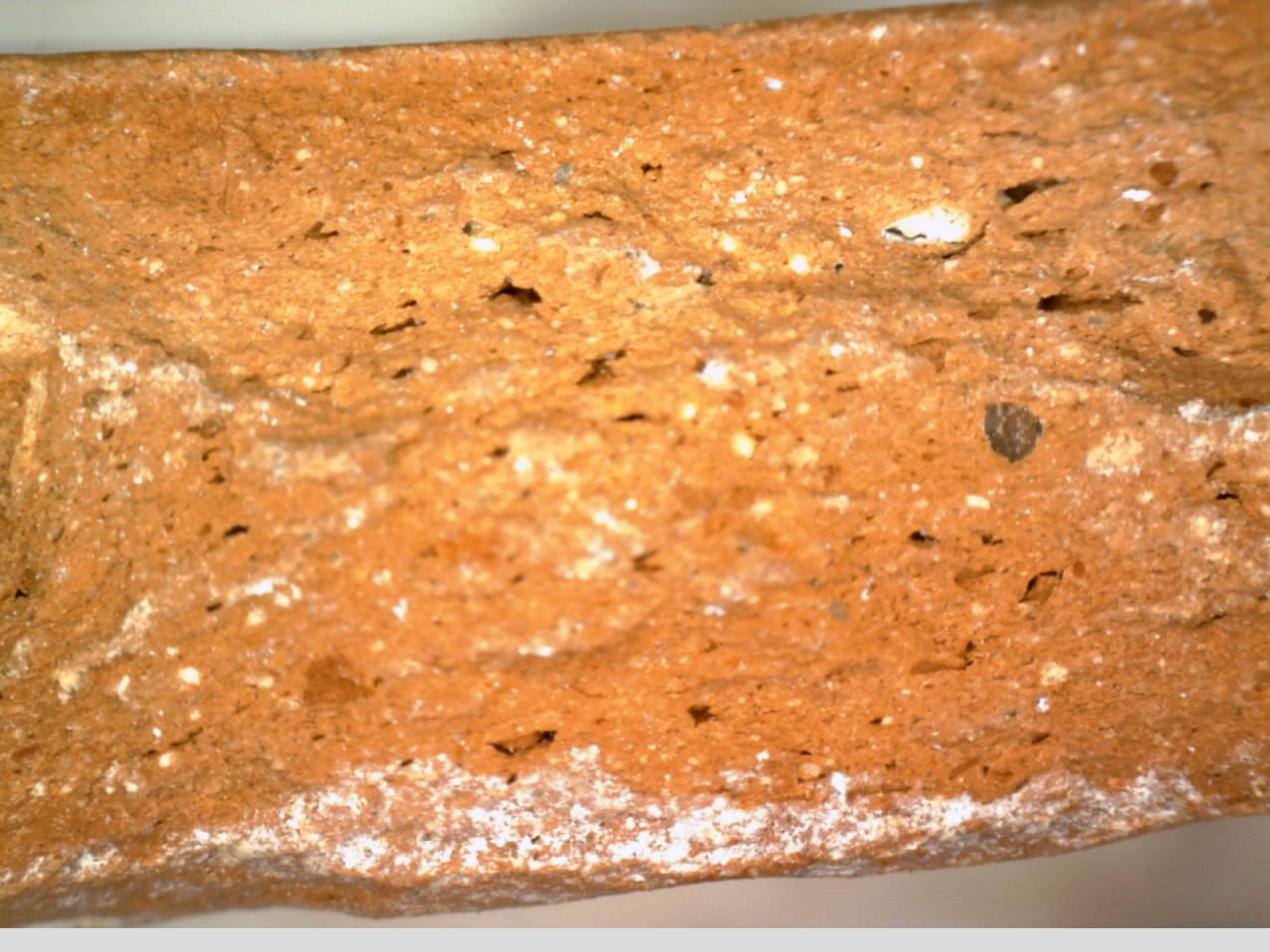

Supplement: Supplementary file 3 — Supplementary material [file mmc3.zip › Appendix A/HTN 31/HTN 31-50m-1.jpg]

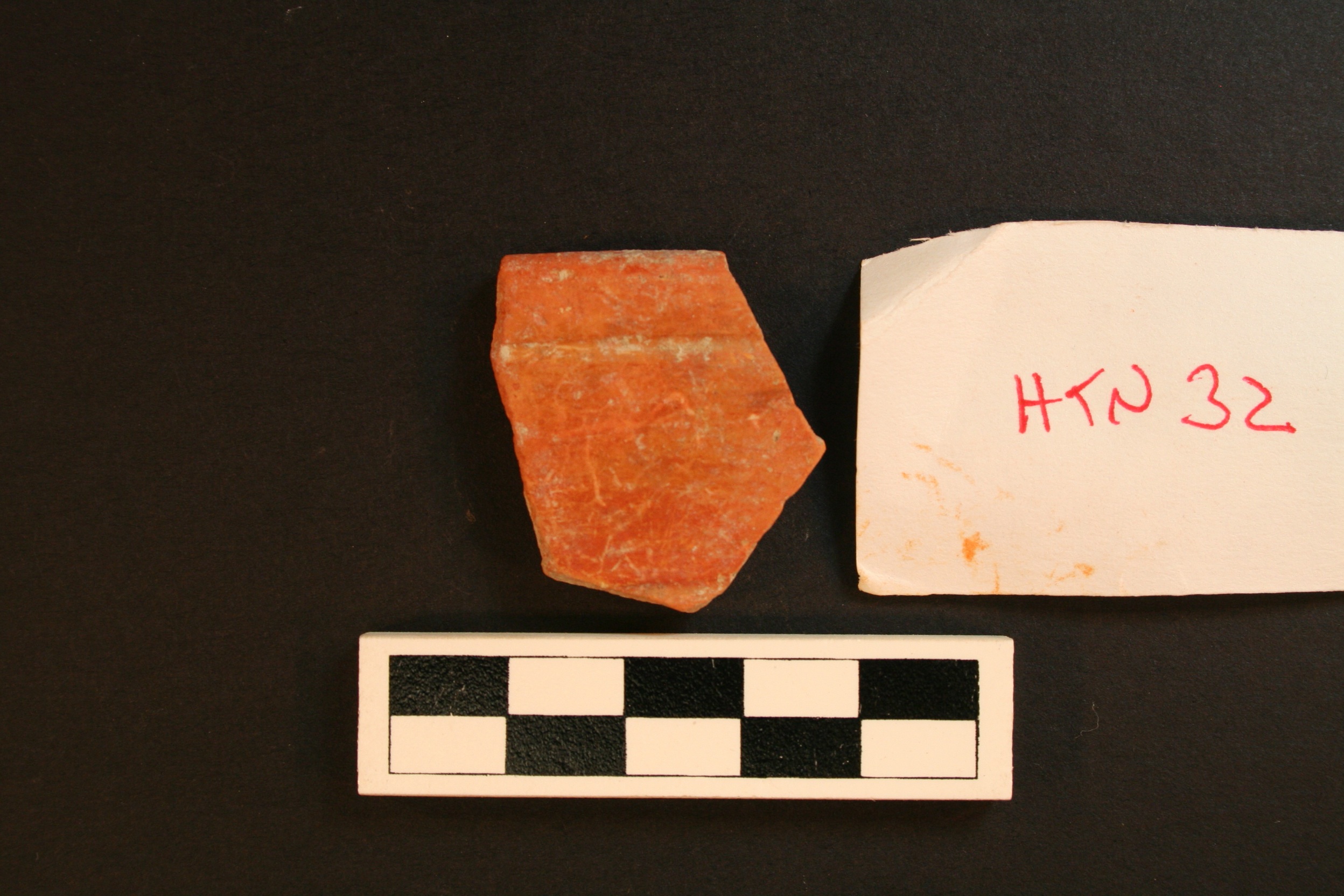

Supplement: Supplementary file 3 — Supplementary material [file mmc3.zip › Appendix A/HTN 32/32a.JPG]

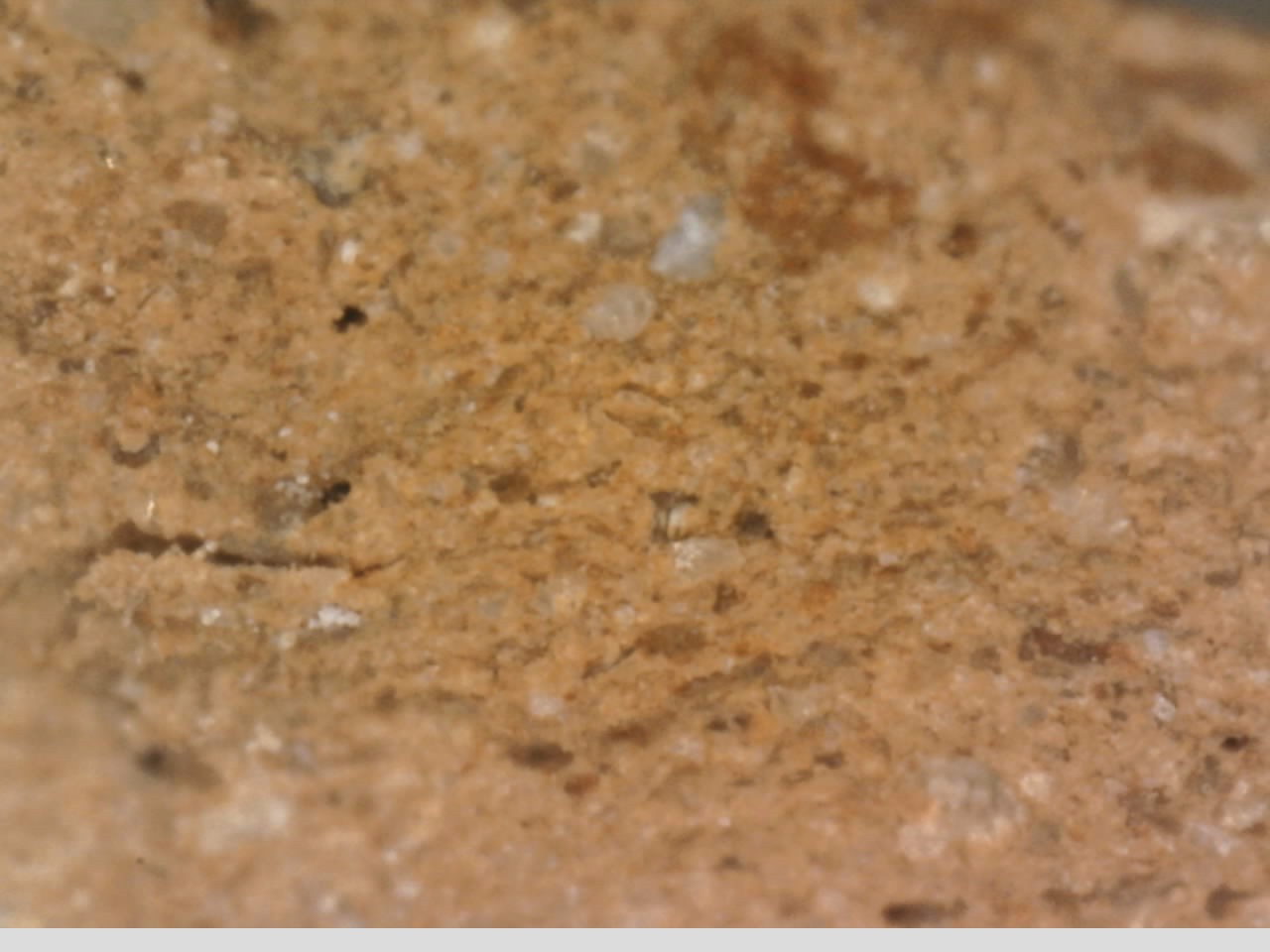

Supplement: Supplementary file 3 — Supplementary material [file mmc3.zip › Appendix A/HTN 32/HTN 32-250m-4.jpg]

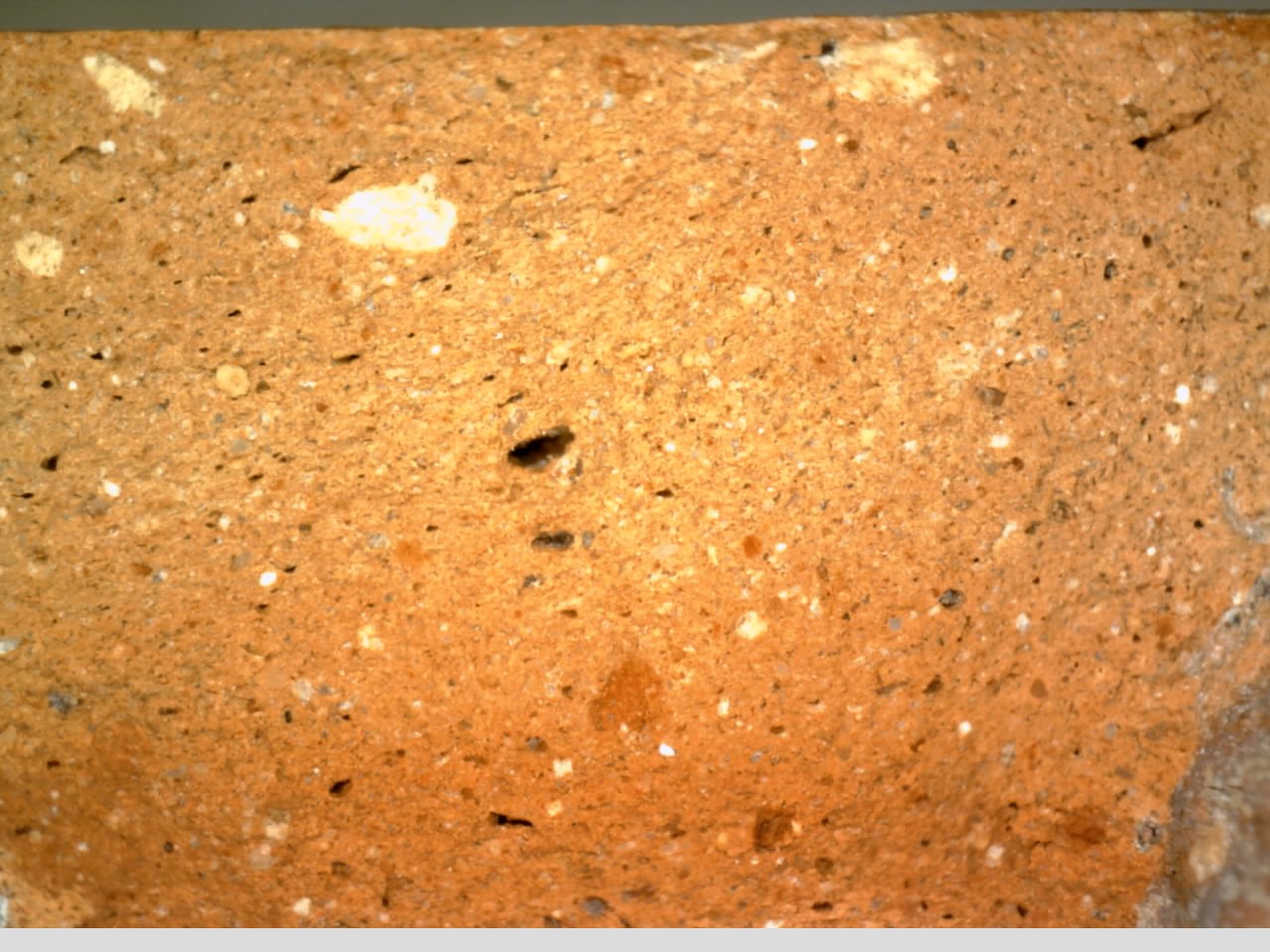

Supplement: Supplementary file 3 — Supplementary material [file mmc3.zip › Appendix A/HTN 32/HTN 32-50m-3.jpg]

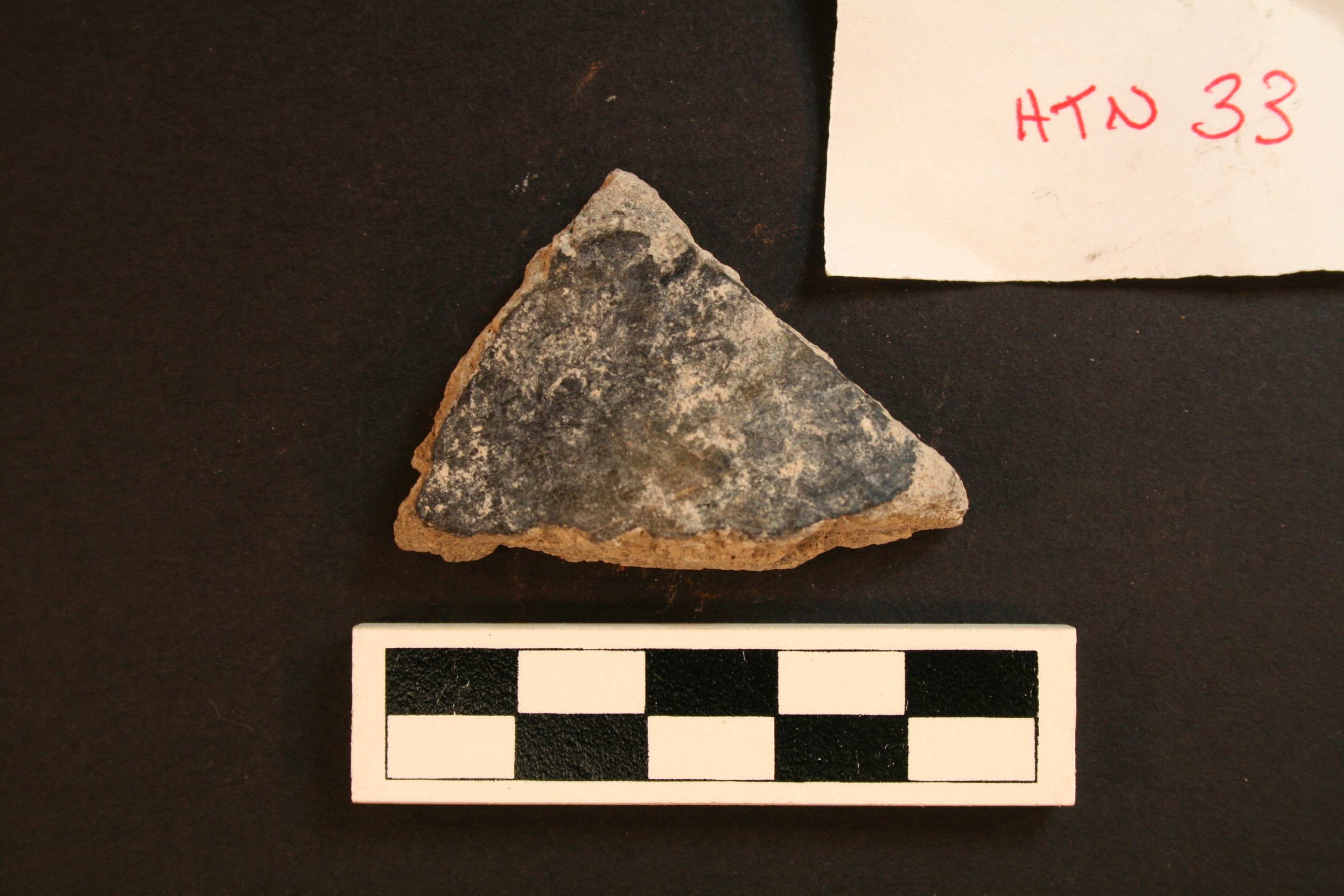

Supplement: Supplementary file 3 — Supplementary material [file mmc3.zip › Appendix A/HTN 33/33a.JPG]

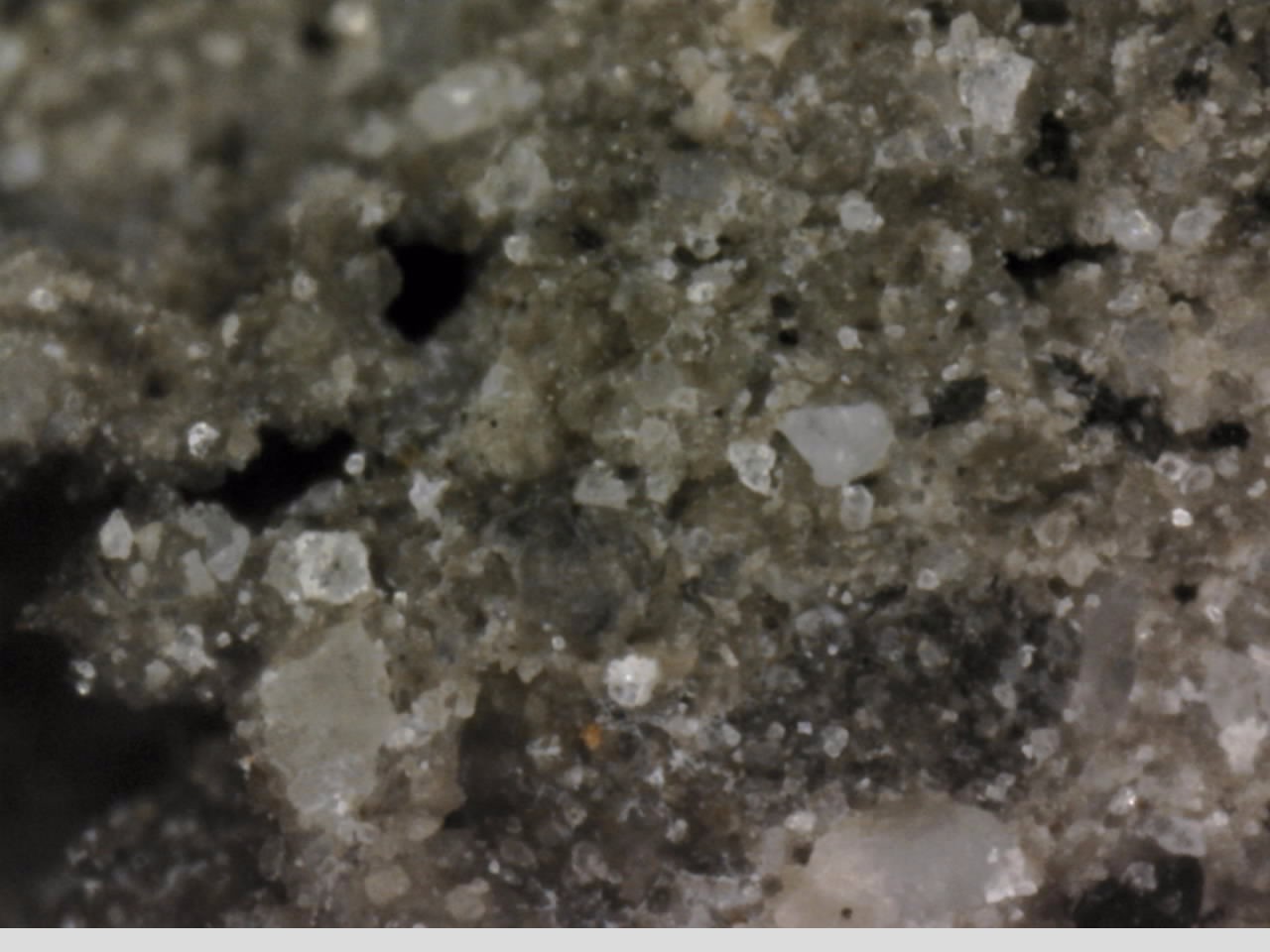

Supplement: Supplementary file 3 — Supplementary material [file mmc3.zip › Appendix A/HTN 33/HTN 33-250m-0.jpg]

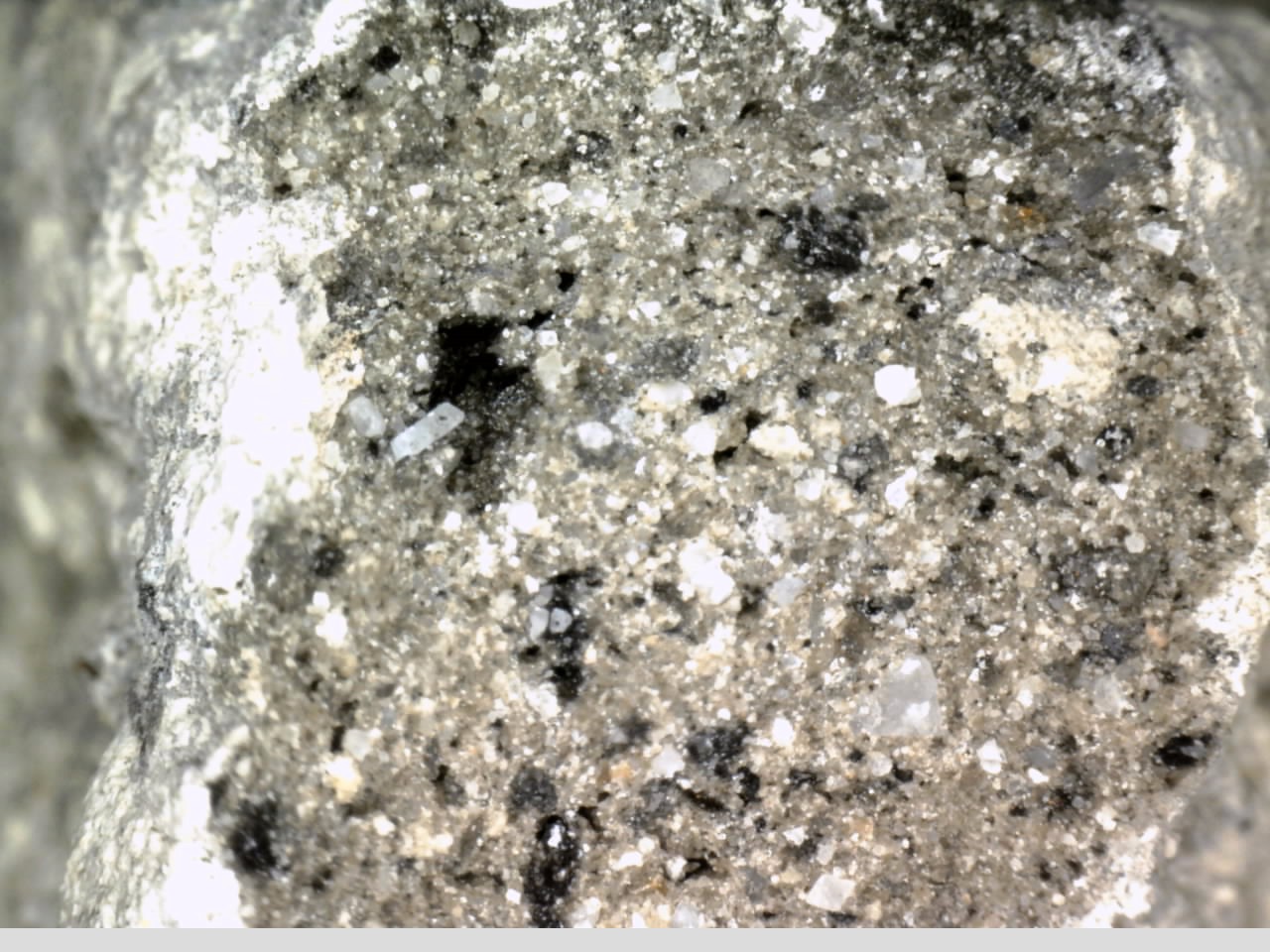

Supplement: Supplementary file 3 — Supplementary material [file mmc3.zip › Appendix A/HTN 33/HTN 33-50m-3.jpg]

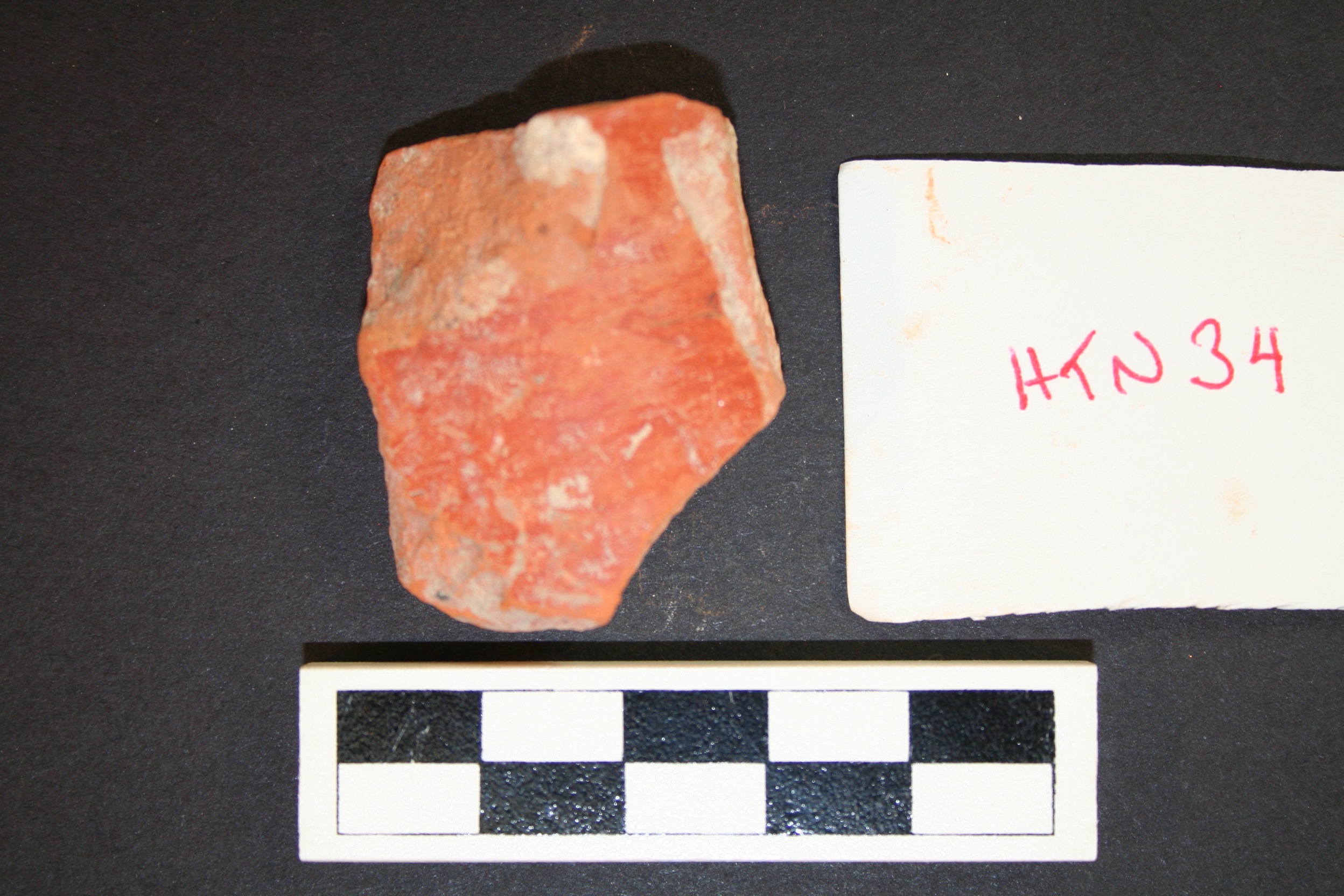

Supplement: Supplementary file 3 — Supplementary material [file mmc3.zip › Appendix A/HTN 34/34a.JPG]

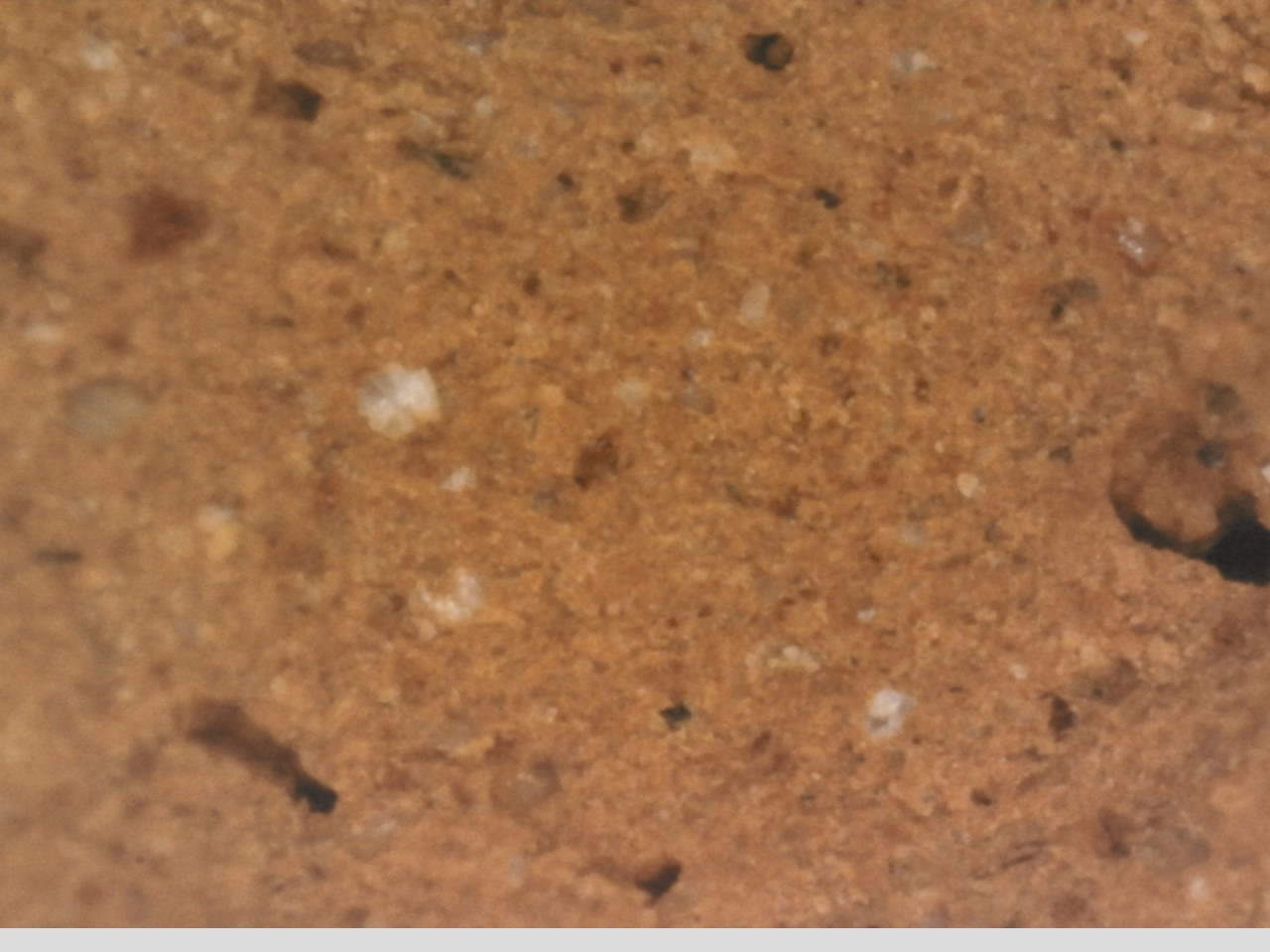

Supplement: Supplementary file 3 — Supplementary material [file mmc3.zip › Appendix A/HTN 34/HTN 34-250m-3.jpg]

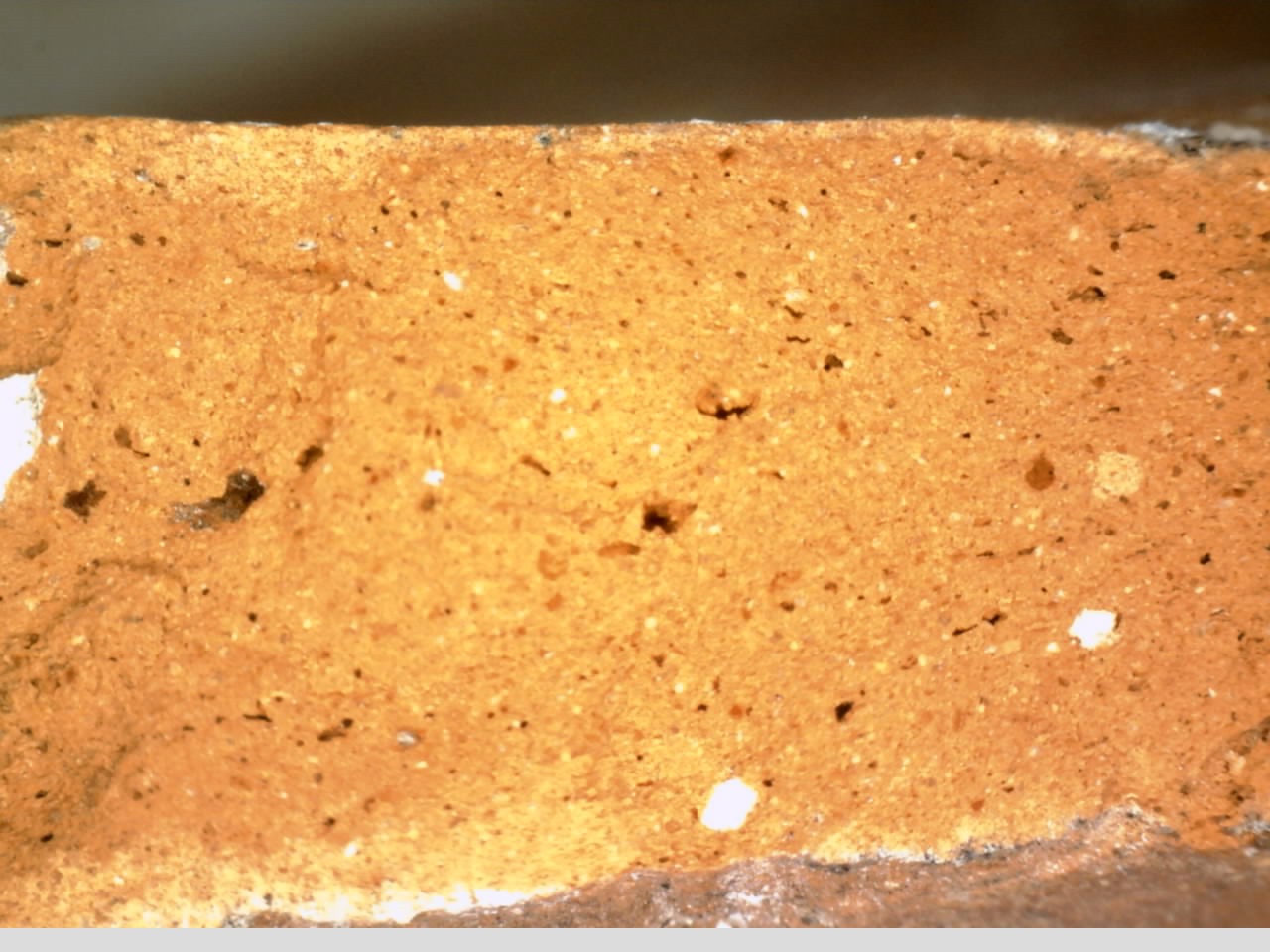

Supplement: Supplementary file 3 — Supplementary material [file mmc3.zip › Appendix A/HTN 34/HTN 34-50m-2.jpg]

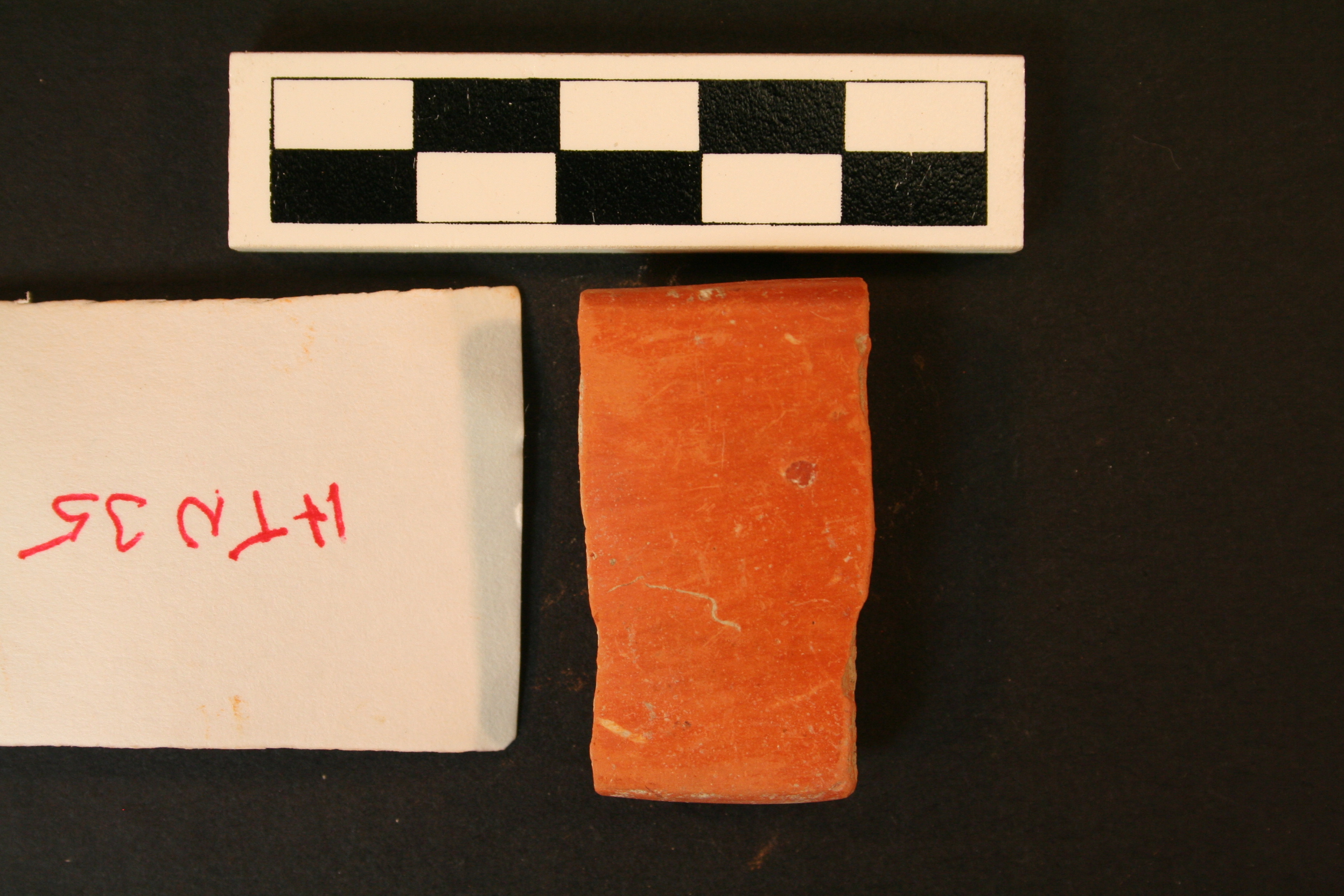

Supplement: Supplementary file 3 — Supplementary material [file mmc3.zip › Appendix A/HTN 35/35a.JPG]

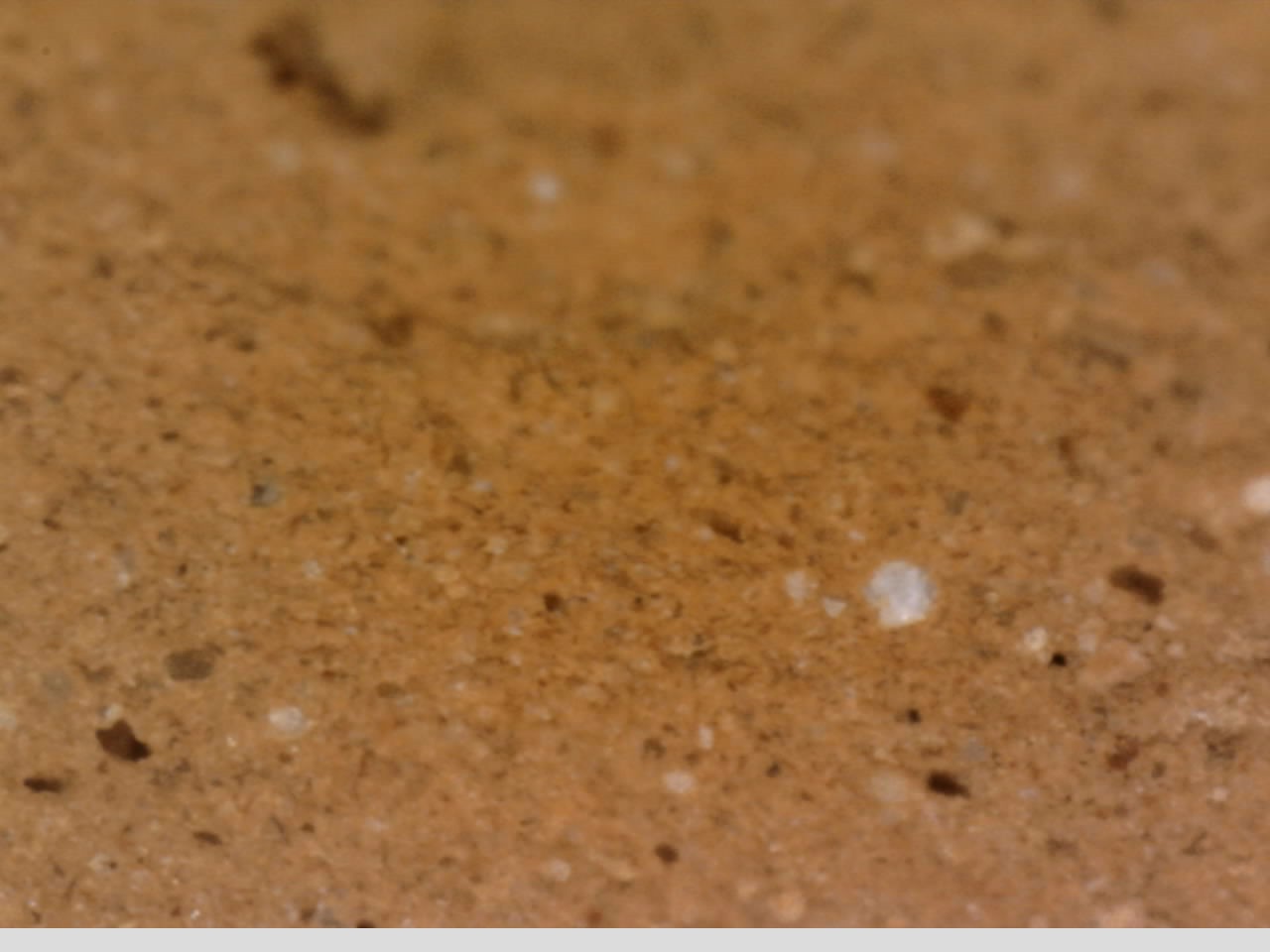

Supplement: Supplementary file 3 — Supplementary material [file mmc3.zip › Appendix A/HTN 35/HTN 35-250m-0.jpg]

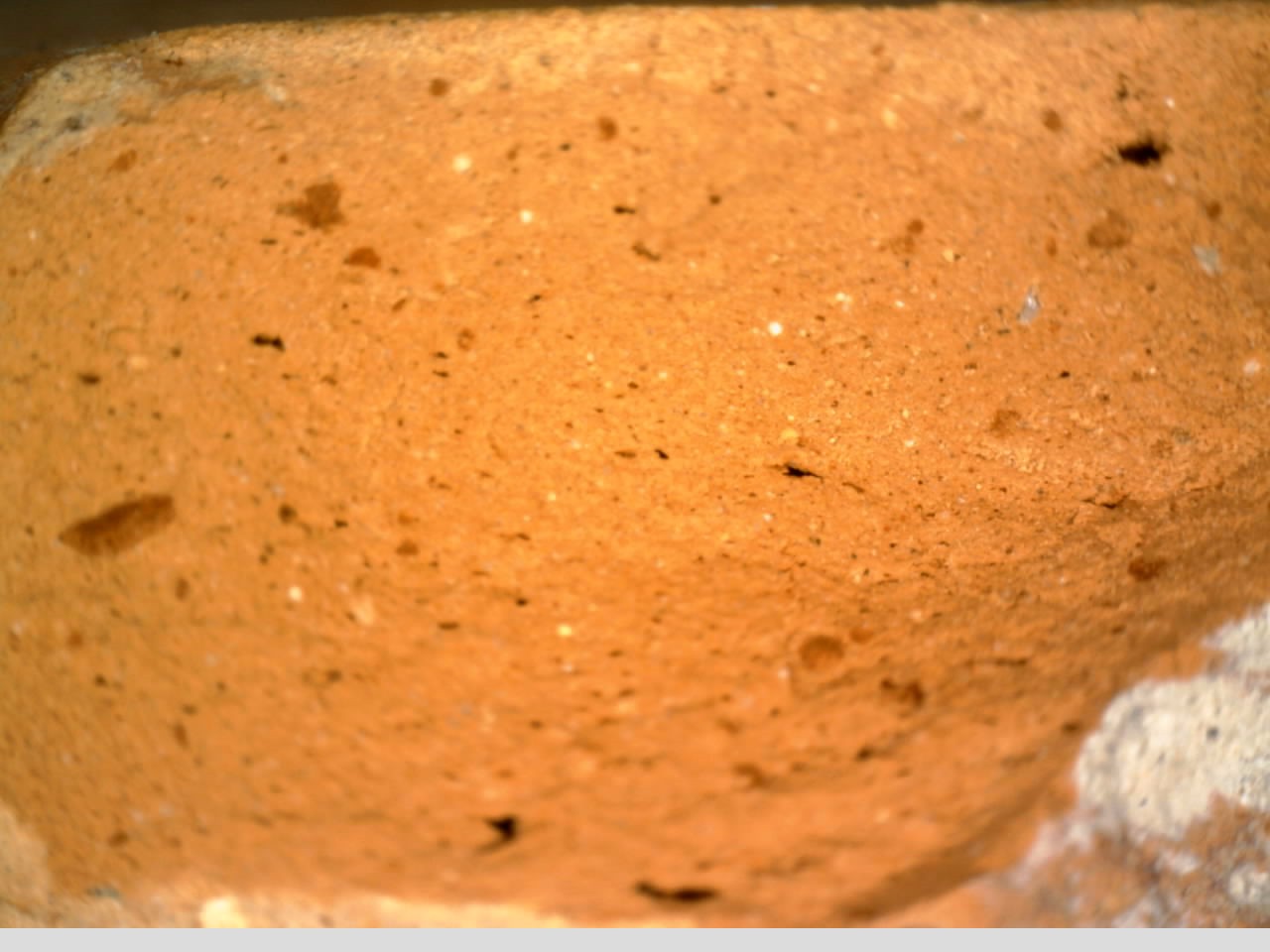

Supplement: Supplementary file 3 — Supplementary material [file mmc3.zip › Appendix A/HTN 35/HTN 35-50m-3.jpg]

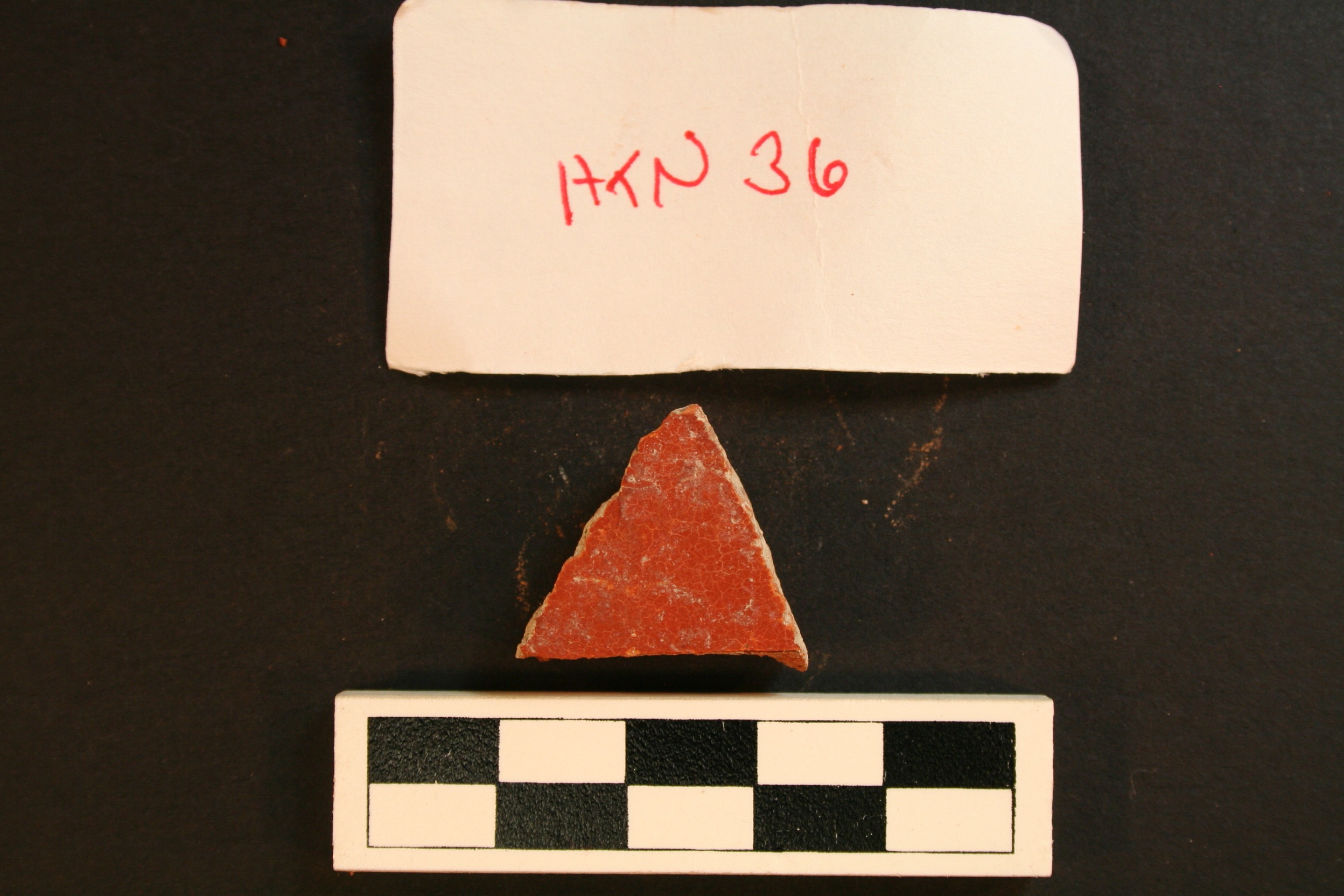

Supplement: Supplementary file 3 — Supplementary material [file mmc3.zip › Appendix A/HTN 36/36a.JPG]

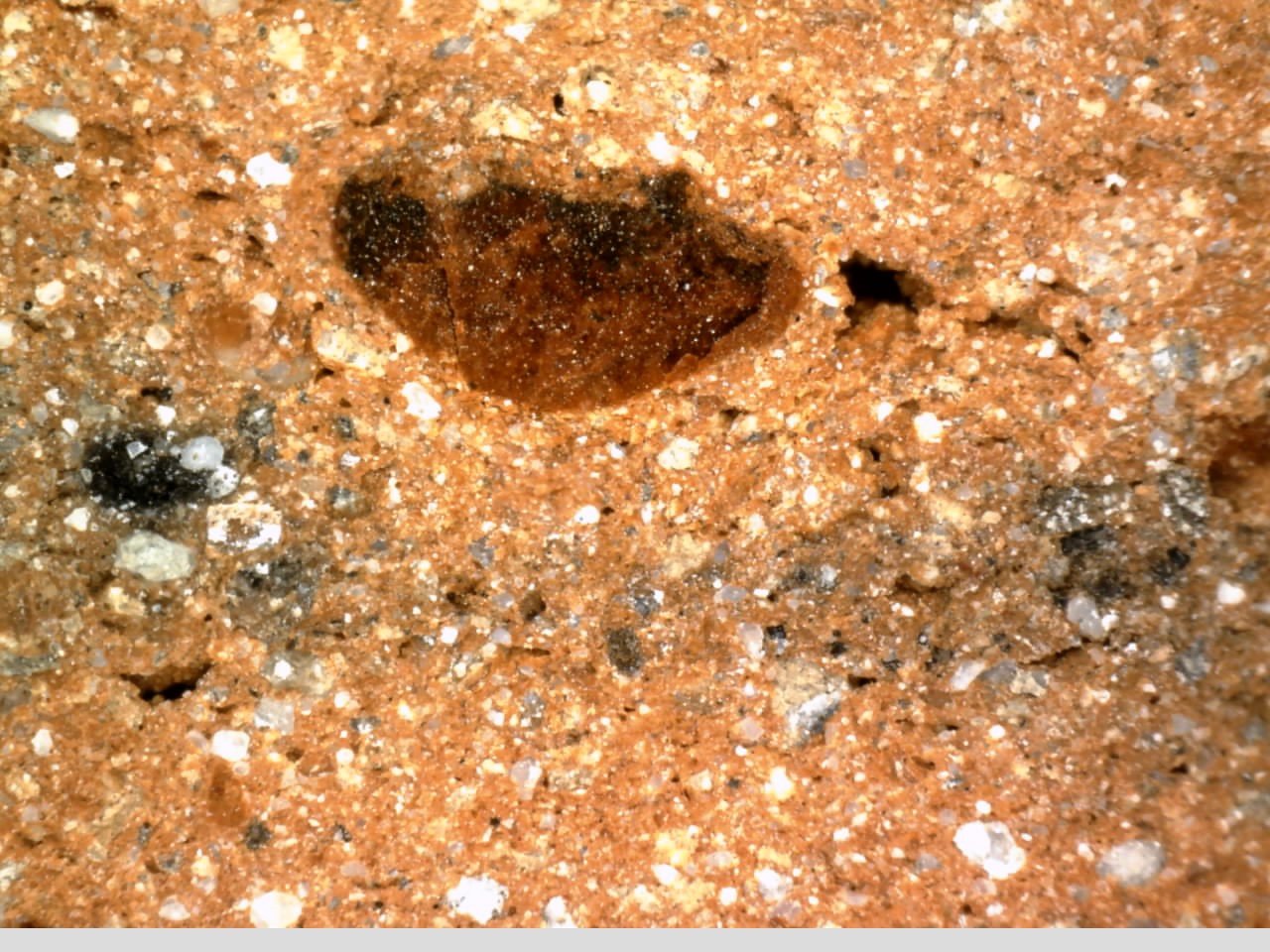

Supplement: Supplementary file 3 — Supplementary material [file mmc3.zip › Appendix A/HTN 36/HTN 36-50m-3.jpg]

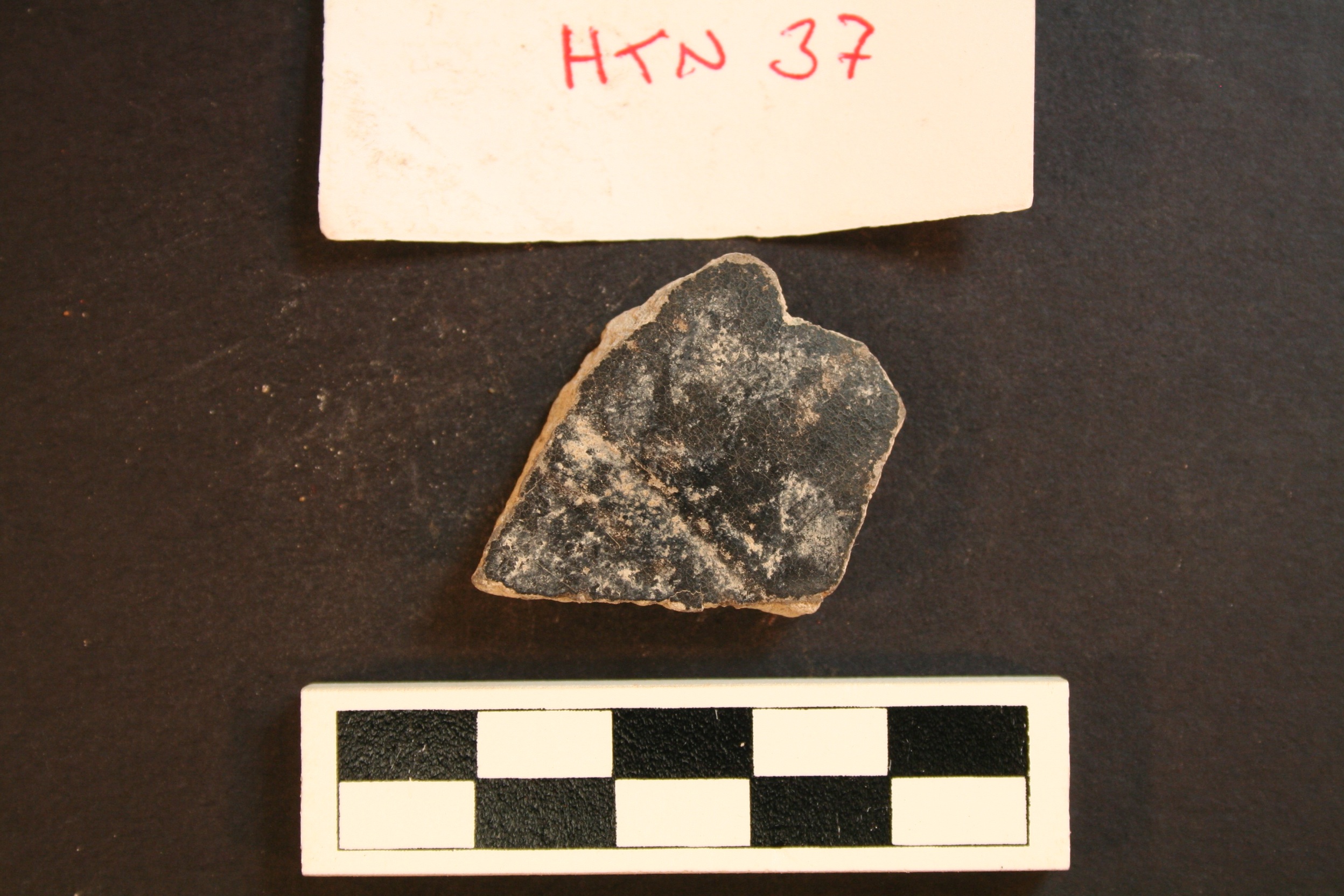

Supplement: Supplementary file 3 — Supplementary material [file mmc3.zip › Appendix A/HTN 37/37a.JPG]

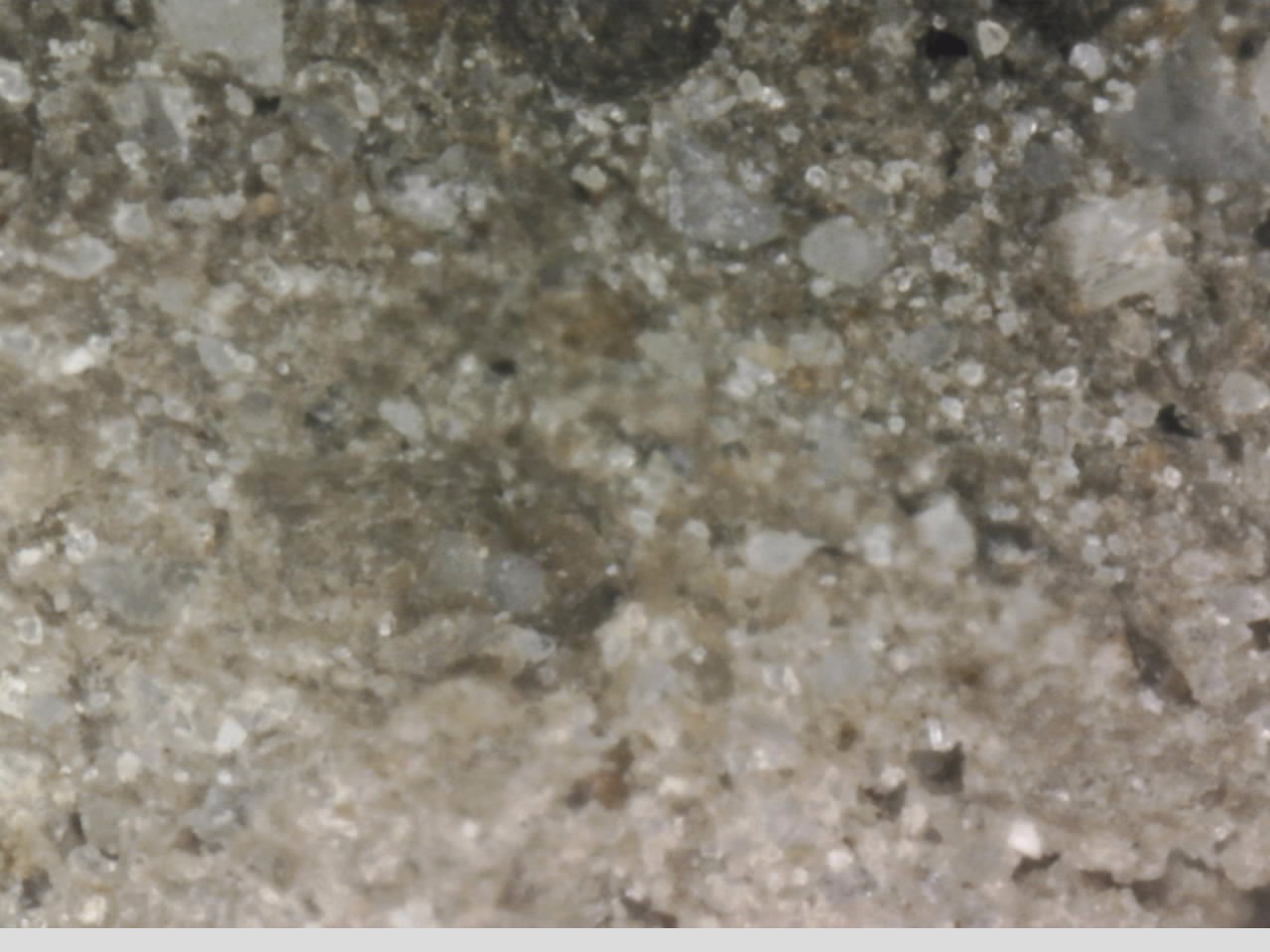

Supplement: Supplementary file 3 — Supplementary material [file mmc3.zip › Appendix A/HTN 37/HTN 37-250m-3.jpg]

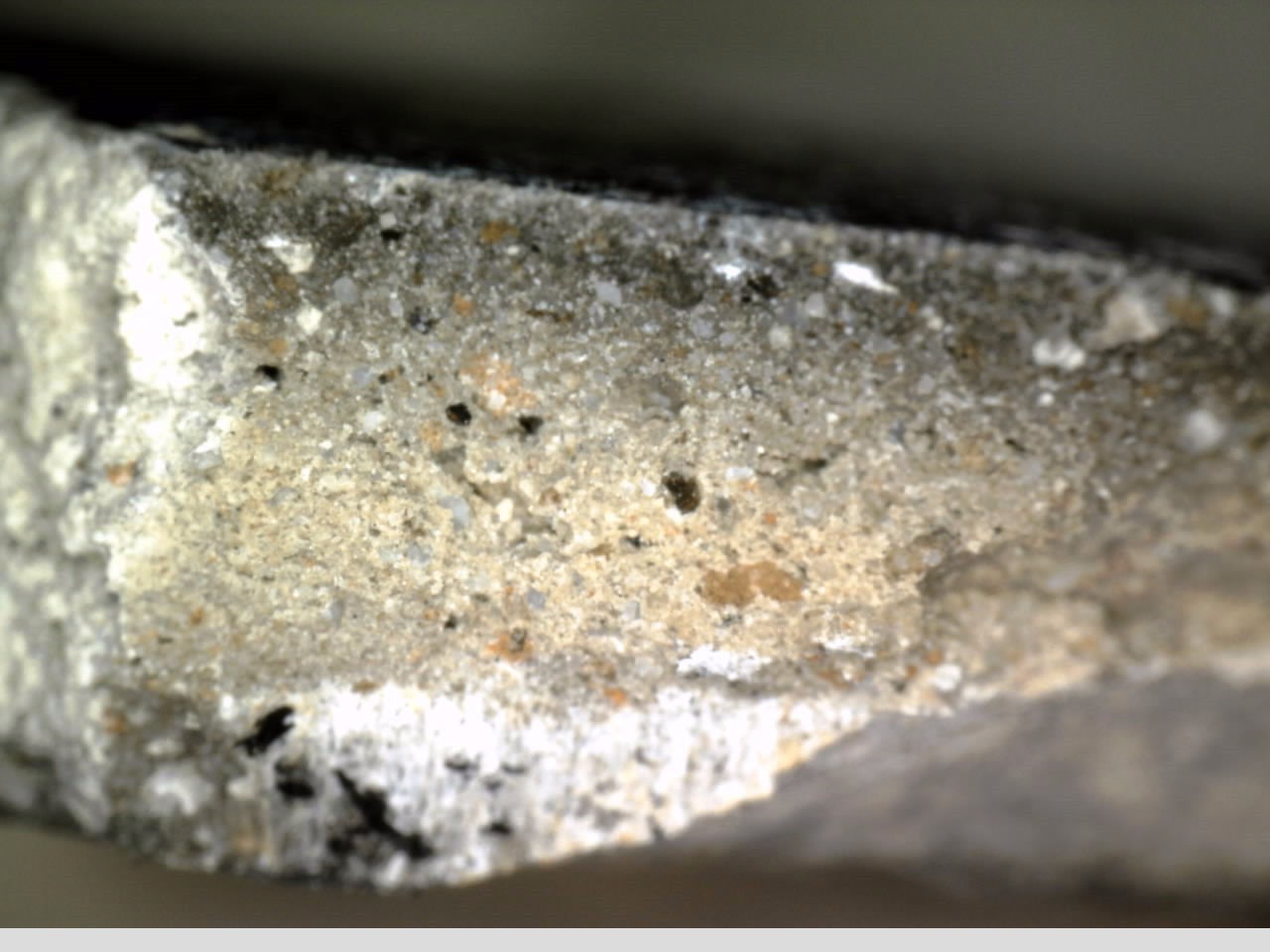

Supplement: Supplementary file 3 — Supplementary material [file mmc3.zip › Appendix A/HTN 37/HTN 37-50m-5.jpg]

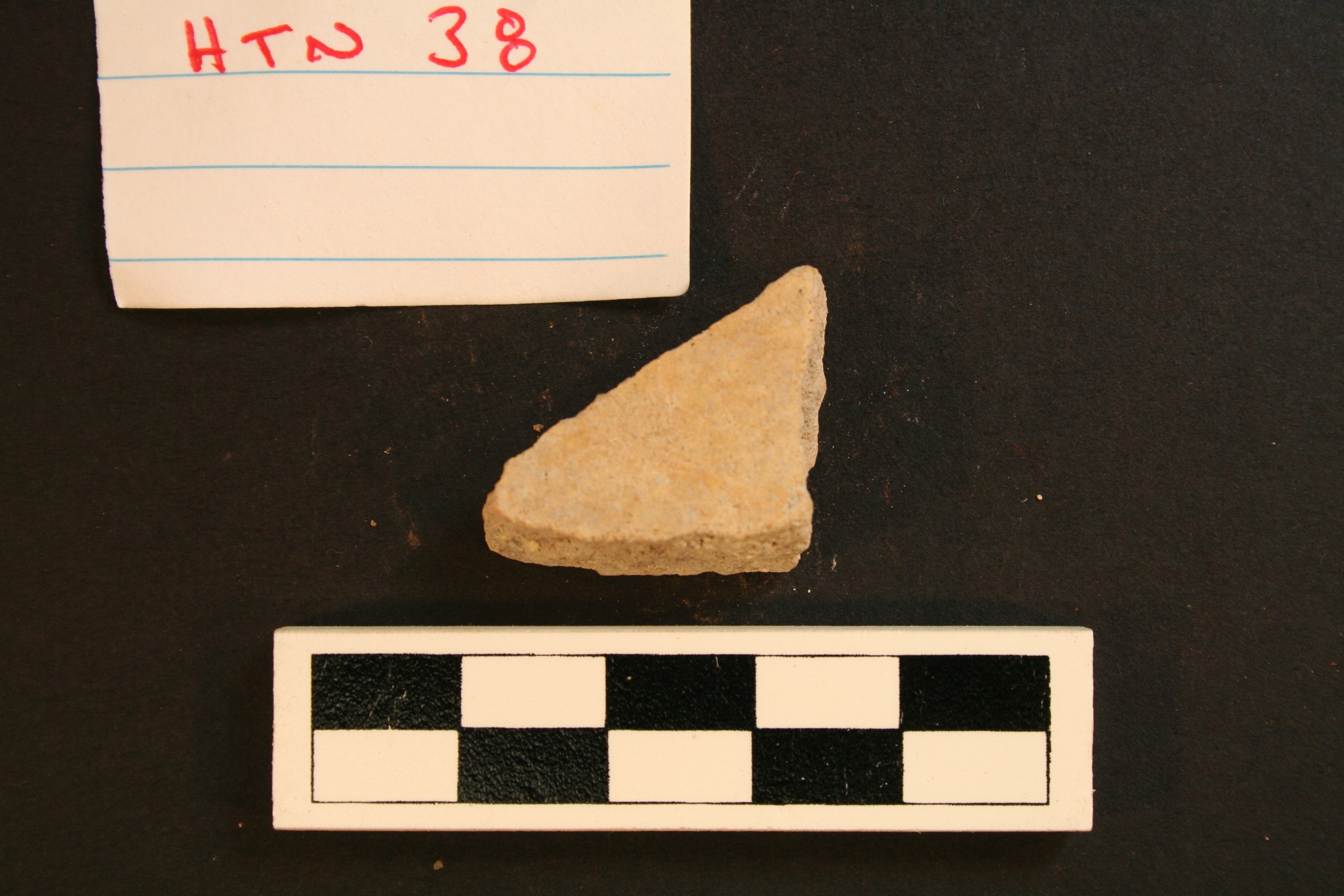

Supplement: Supplementary file 3 — Supplementary material [file mmc3.zip › Appendix A/HTN 38/38a.JPG]

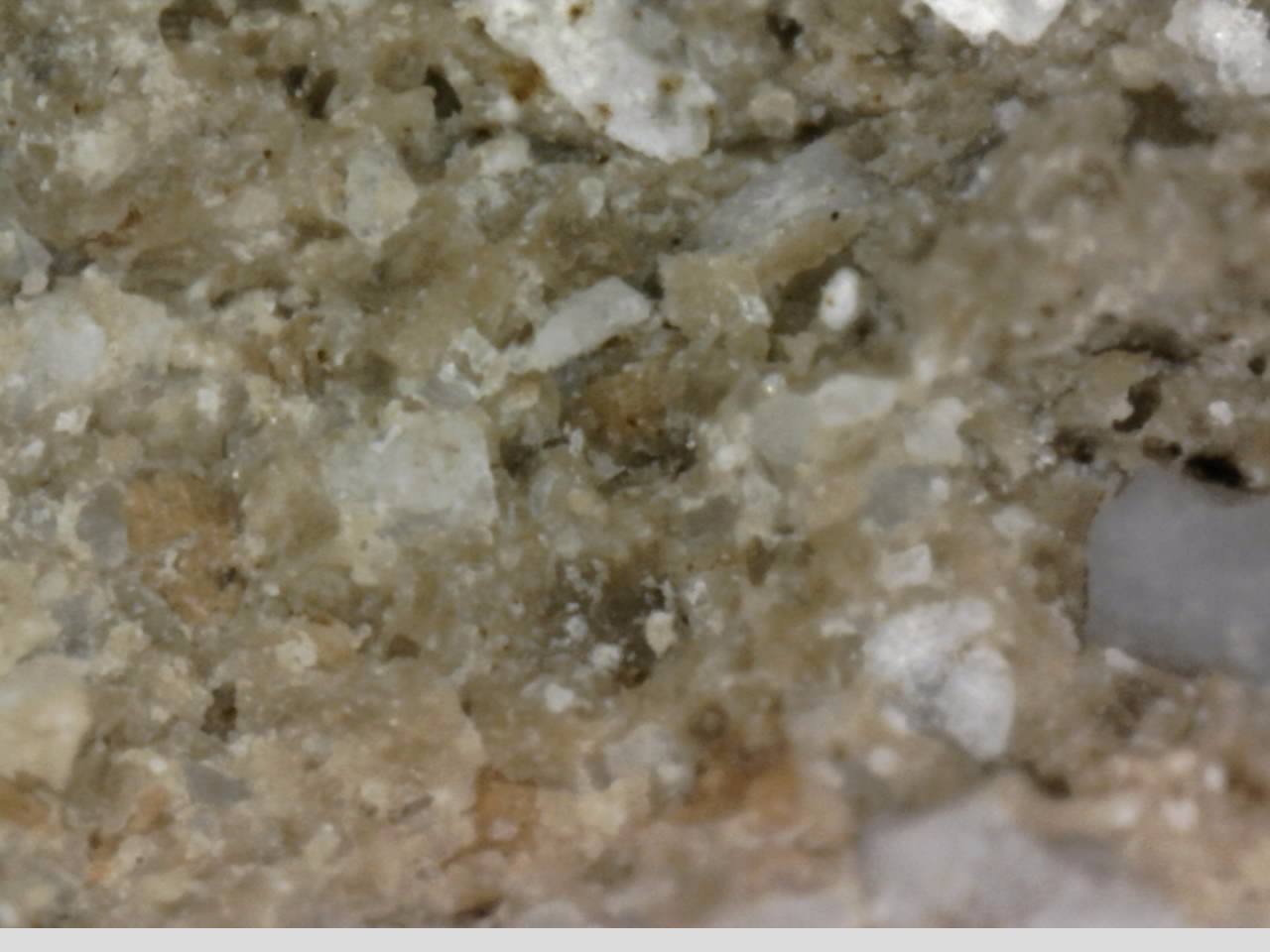

Supplement: Supplementary file 3 — Supplementary material [file mmc3.zip › Appendix A/HTN 38/HTN 38-250m-0.jpg]

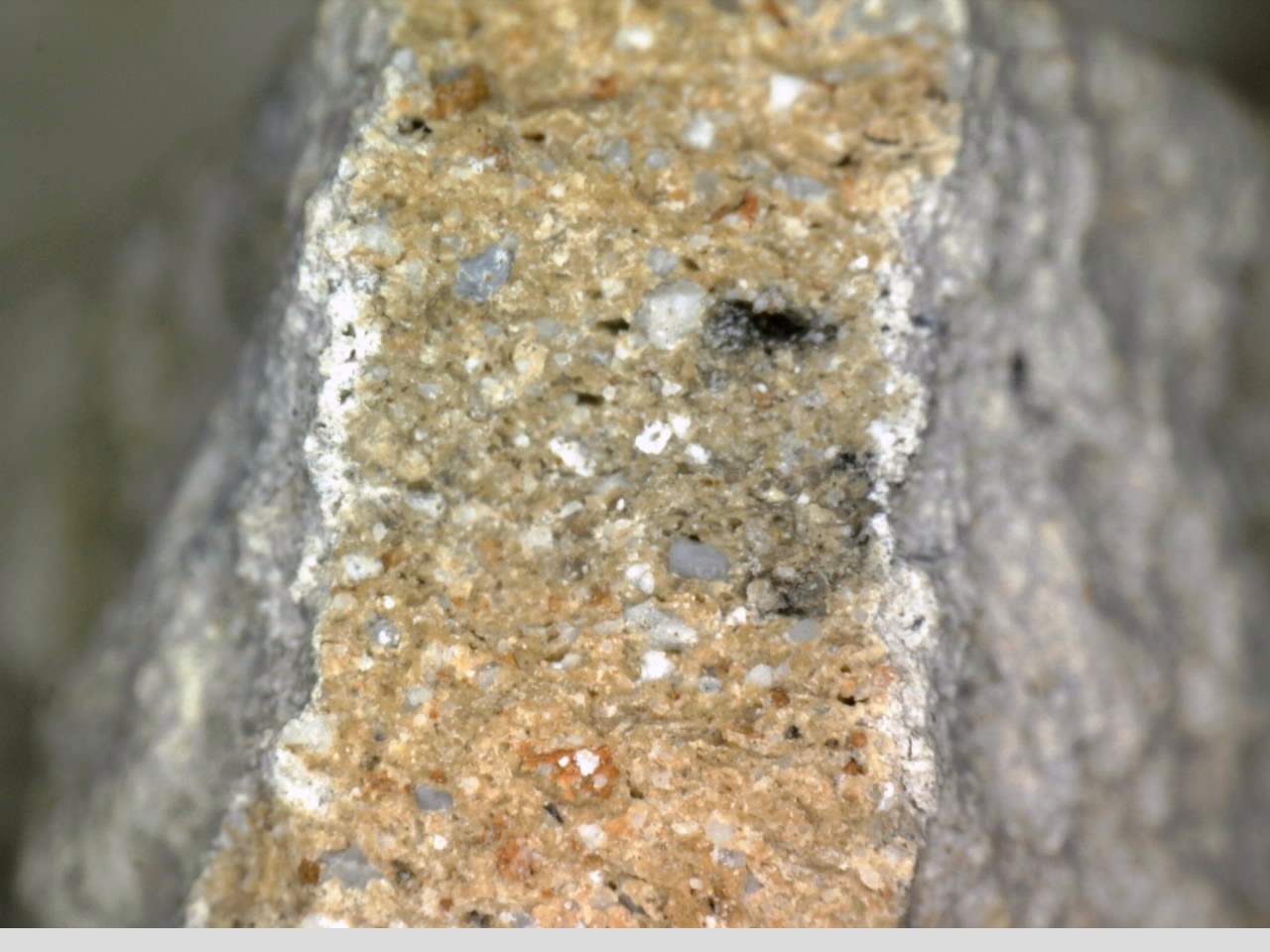

Supplement: Supplementary file 3 — Supplementary material [file mmc3.zip › Appendix A/HTN 38/HTN 38-50m-5.jpg]

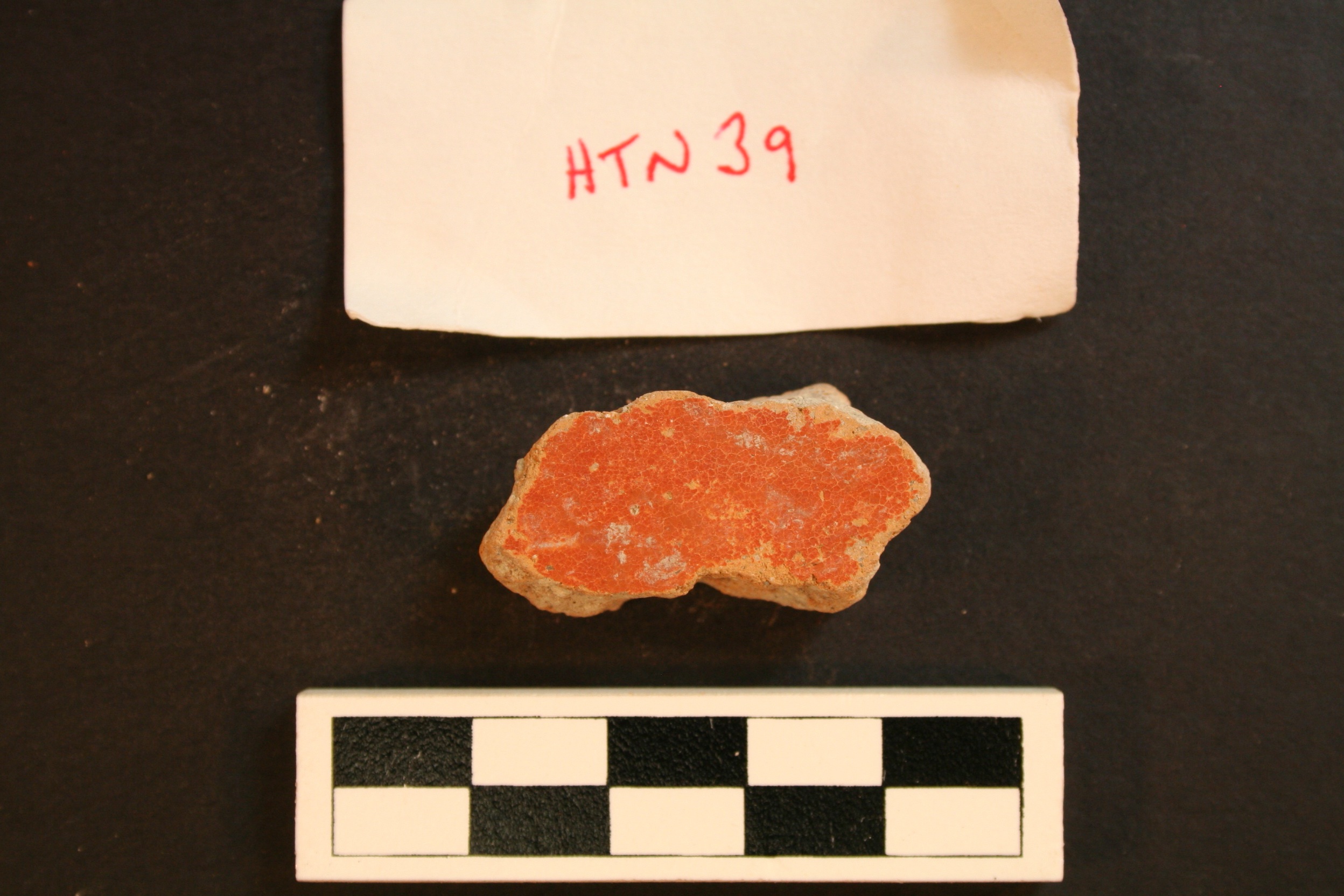

Supplement: Supplementary file 3 — Supplementary material [file mmc3.zip › Appendix A/HTN 39/39a.JPG]

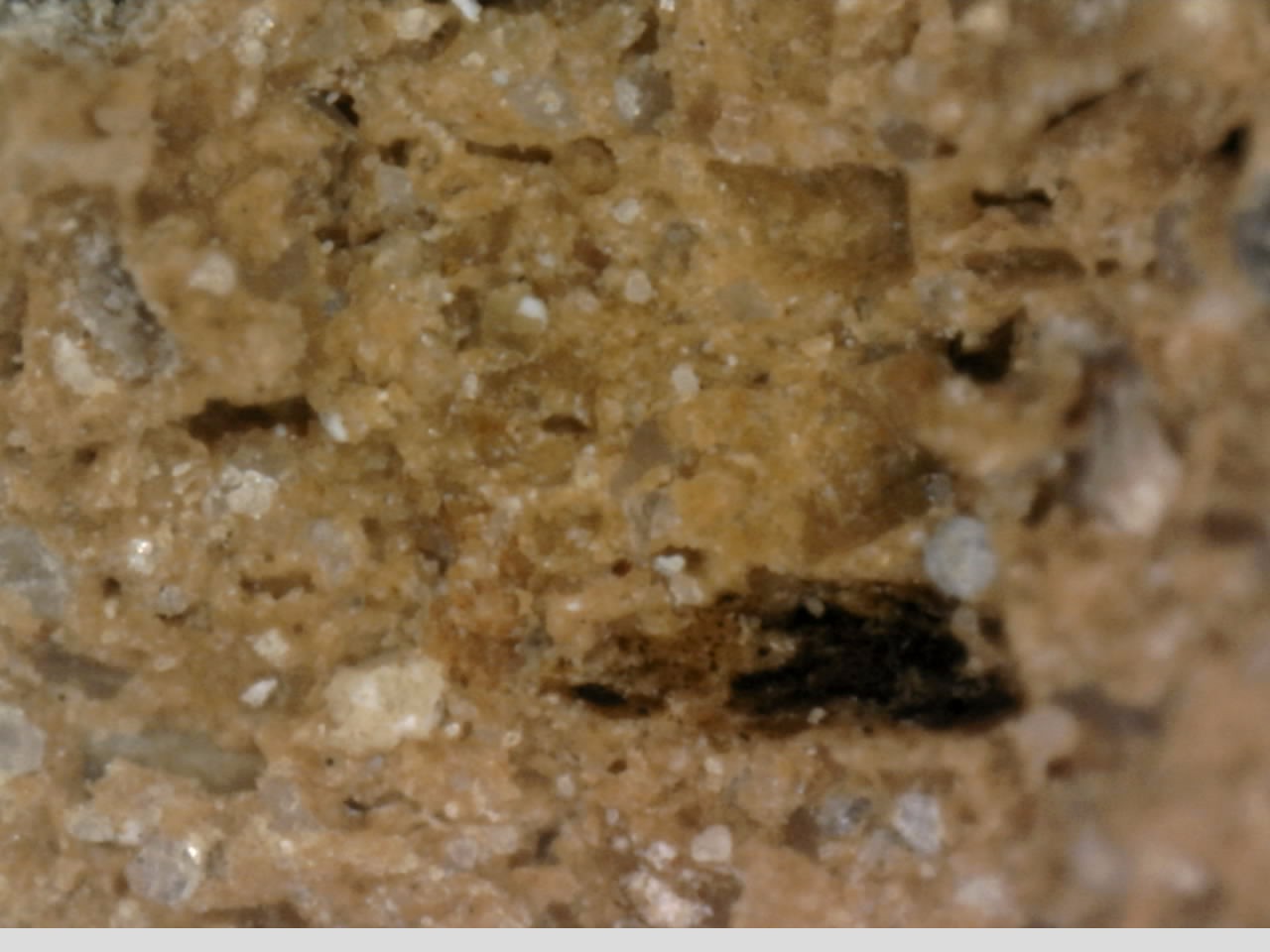

Supplement: Supplementary file 3 — Supplementary material [file mmc3.zip › Appendix A/HTN 39/HTN 39-250m-0.jpg]

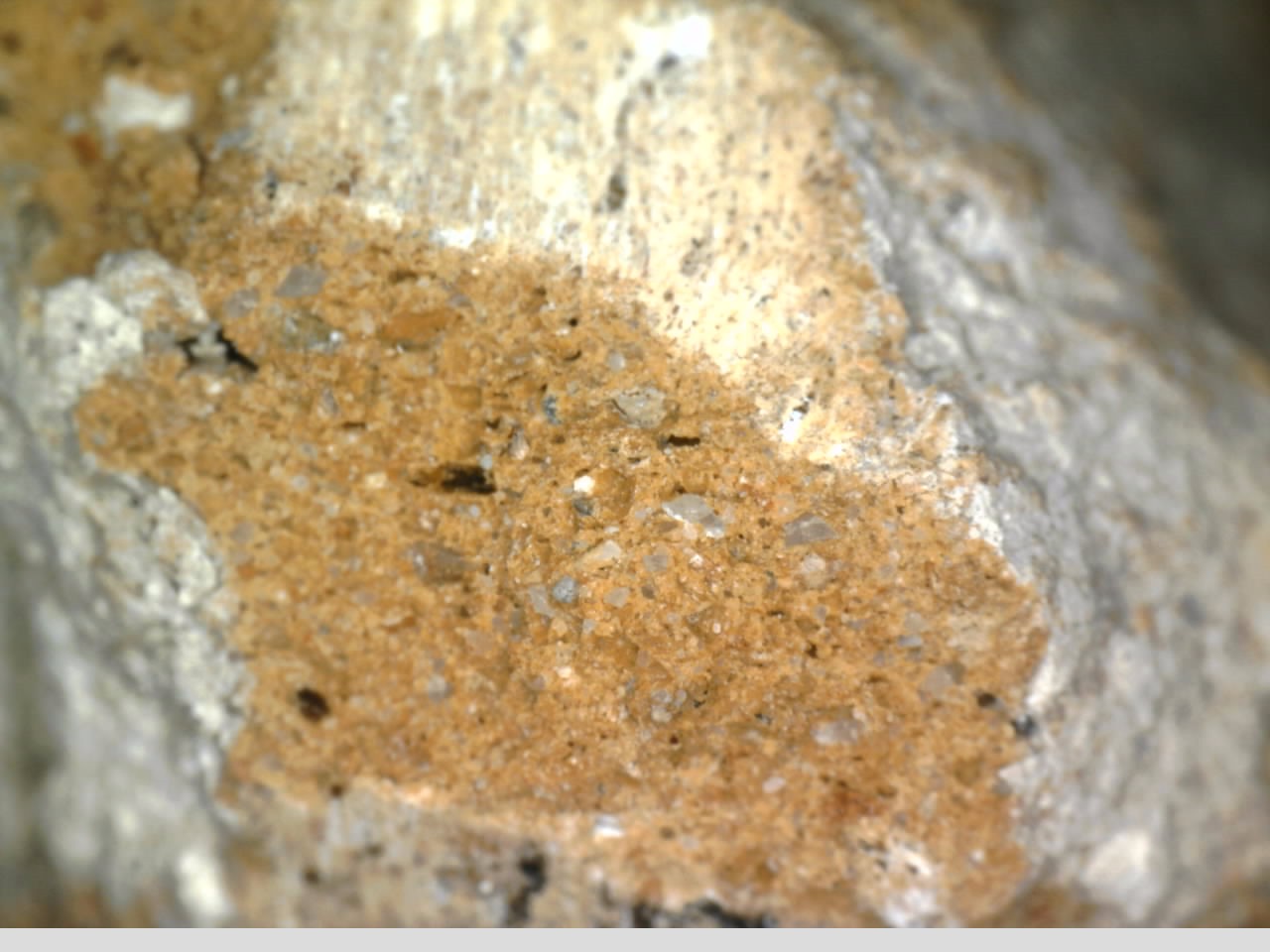

Supplement: Supplementary file 3 — Supplementary material [file mmc3.zip › Appendix A/HTN 39/HTN 39-50m-2.jpg]

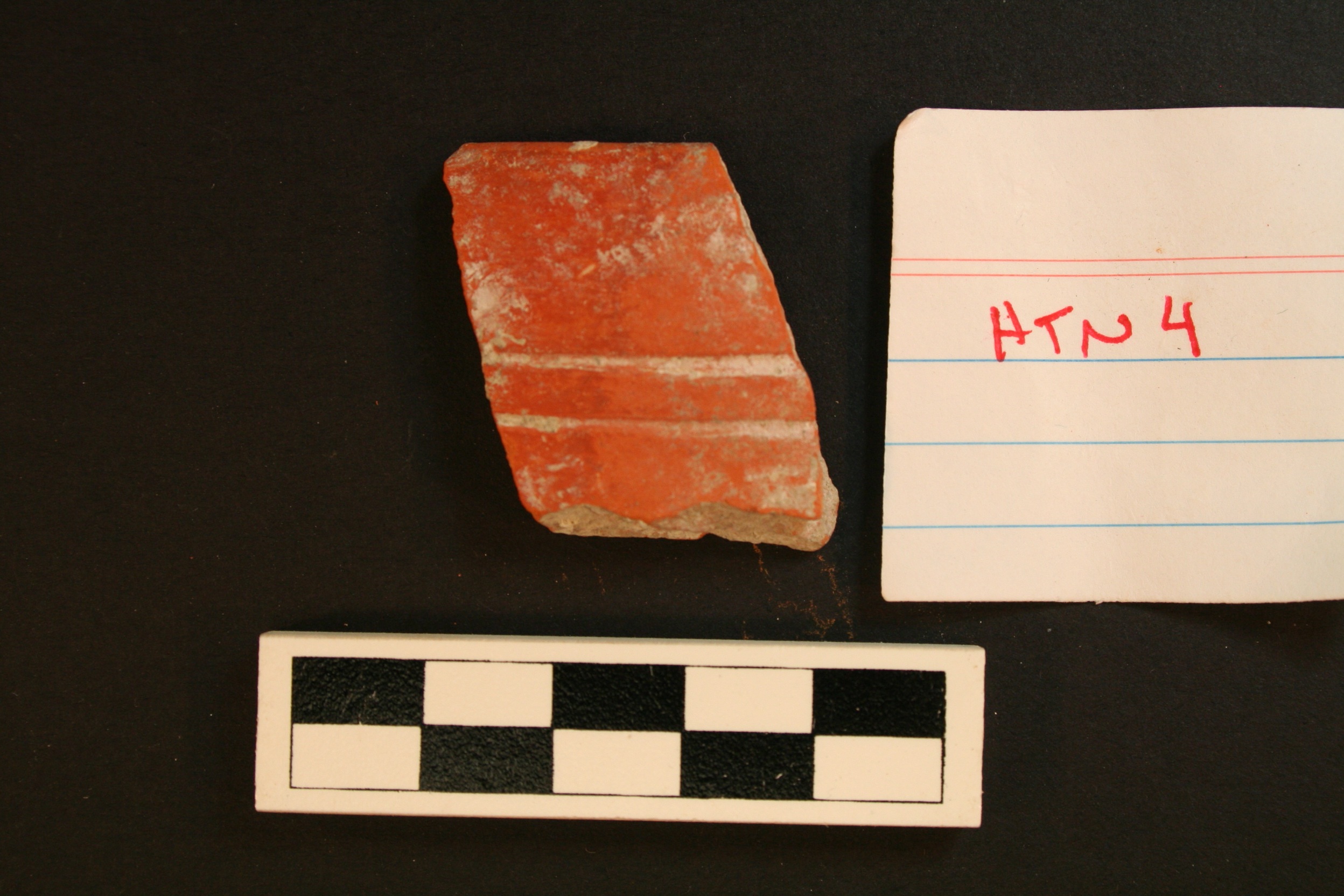

Supplement: Supplementary file 3 — Supplementary material [file mmc3.zip › Appendix A/HTN 4/4b.JPG]

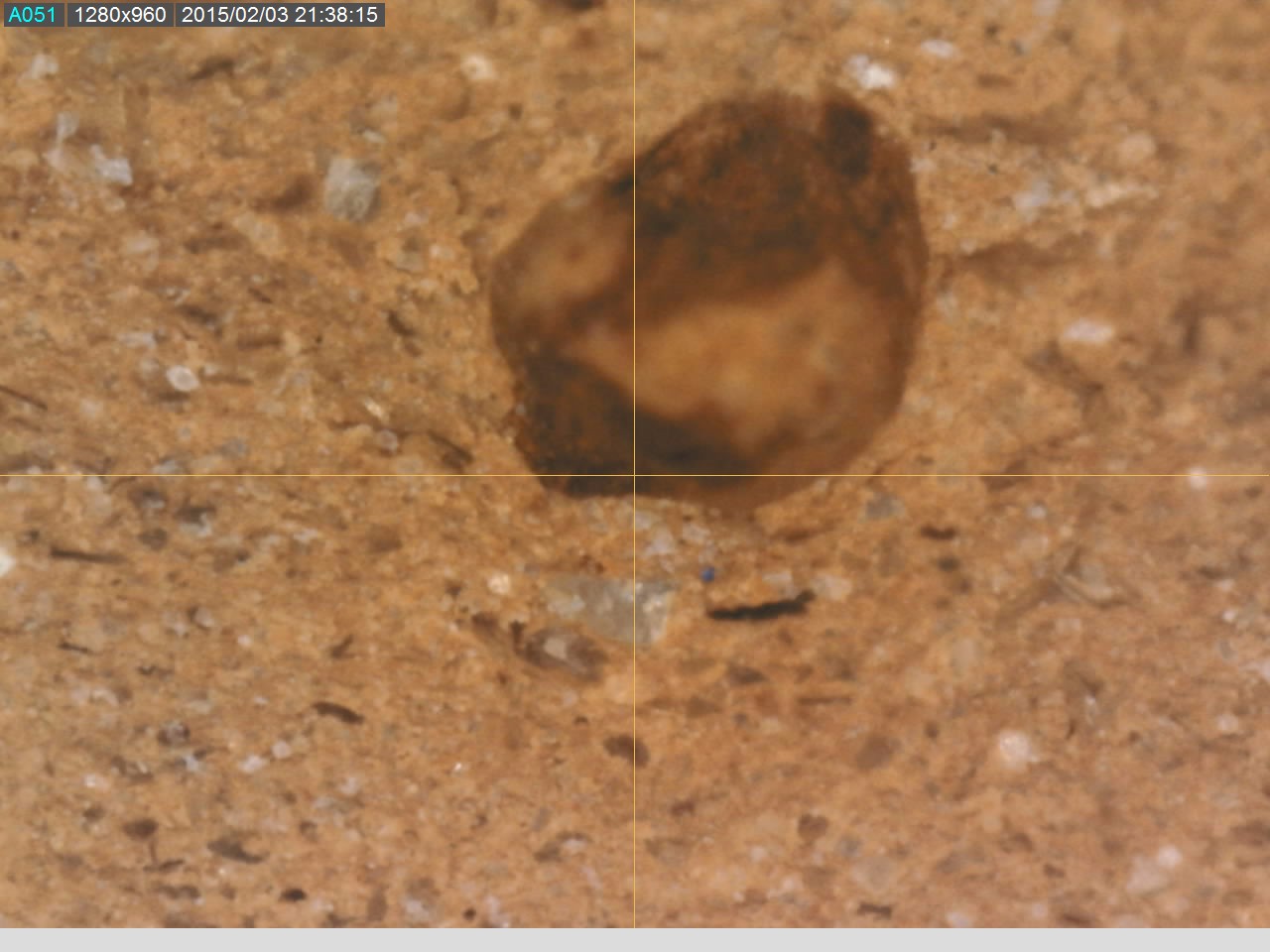

Supplement: Supplementary file 3 — Supplementary material [file mmc3.zip › Appendix A/HTN 4/HTN 4-250m-1.jpg]

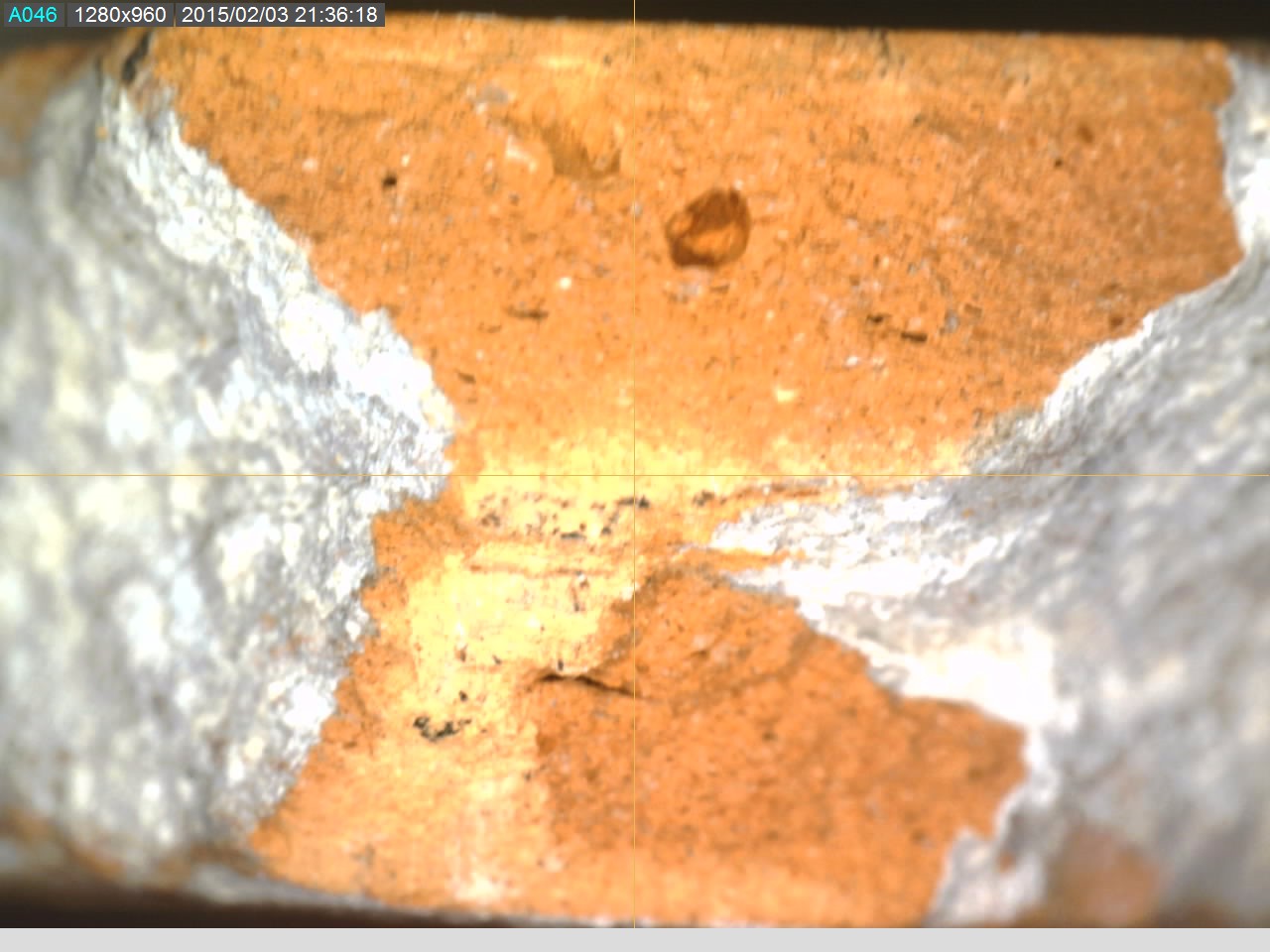

Supplement: Supplementary file 3 — Supplementary material [file mmc3.zip › Appendix A/HTN 4/HTN 4-50m-1.jpg]

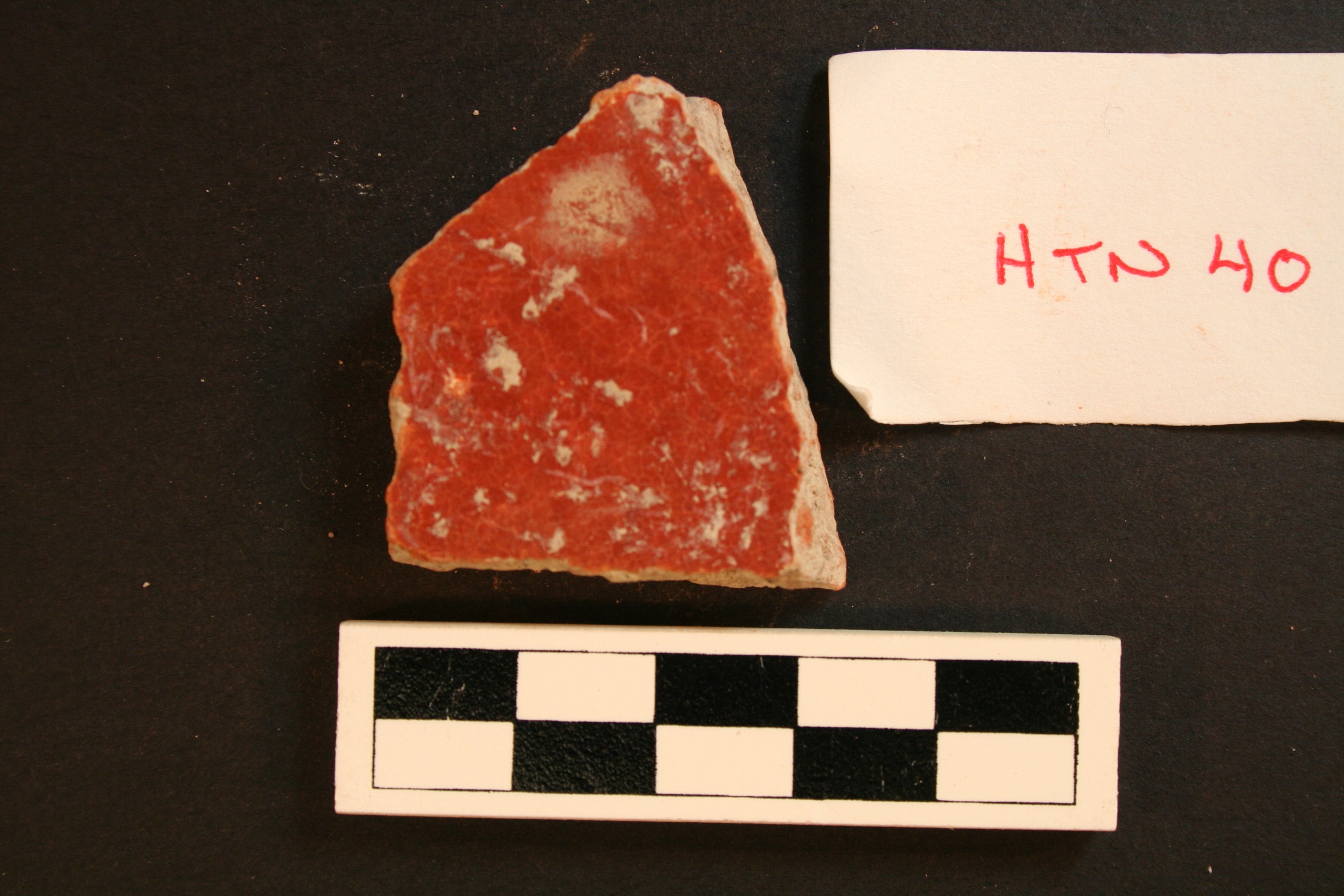

Supplement: Supplementary file 3 — Supplementary material [file mmc3.zip › Appendix A/HTN 40/40a.JPG]
