# Supplementary material for: An atlas of paste fabrics and supplemental paste compositional data from late middle preclassic-period ceramics at the Maya site of Holtun, Guatemala
Source: Data Brief. 2017 Mar 19;12:55–67. doi: 10.1016/j.dib.2017.03.024 (PMC5376253; doi:10.1016/j.dib.2017.03.024)
Supplement: Supplementary file 4 — Supplementary material [file mmc4.pdf]

SUPPLEMENTAL TABLE 4*
Chi-square Tests of Association

*It is important to note that microscopic paste examination and NAA revealed the presence of 5 ambiguous paste samples. The samples are HTN 049, HTN 082g, HTN 082r, HTN 096, and HTN 097. Based on form, HTN 049 belonged to a potential Pre-Mamom (i.e., early Middle Preclassic period) vessel. Its type: variety was undetermined, but it was included in the sample in order to create a baseline comparison for any future Pre-Mamom material. Because it is chronologically earlier and un-typed, HTN 049 has little bearing on this study and will not be included in this discussion. HTN 082g and HTN 082r come from what was believed to be a ball of Mars Orange Paste Ware clay found in HTN 3-1-6. However, after correlating microscopic paste examination with NAA findings, it appears this was not a ball of clay, but a bright orange piece of clay from paste that was used to fashion other Holtun slipped wares. In addition, because they lack a type: variety designation (much less group or ware designation), HTN 082g and HTN 082r have little to add to this data. In addition both samples came from the unassigned paste composition group, the inclusion of which in this discussion adds little to clarify any patterns that may have emerged. HTN 096 and HTN 097 were initially thought to be coarser-grained examples of Mars Orange Paste Ware that exhibited a thin milky cream wash and a thin black wash respectively. After microscopic paste analysis these classifications became suspect as both pastes contained large amounts of crystalline calcite, despite their bright orange color. NAA revealed these two samples do not exhibit the same composition as other Mars Orange Paste Ware sherds in this study and were assigned to Group 4. It is possible these sherds represent local production of Mars Orange Paste Ware, but it is also possible the two sherds represent differentially preserved weathered types of Flores Waxy Ware. Because of the inability to accurately classify them within the type: variety system, and their lack of corresponding Mars Orange Ware paste composition, we prefer to exclude them from these data.

Table 1. Type: Variety by Paste Group

Type: Variety	Group 1	Group 2	Group 3	Group 4	Unassigned	TOTAL
Achiotes Unslipped: Achiotes Variety	0	0	0	4	0	**4**
Centenario Fluted: Centenario Variety	0	0	2	0	0	**2**
Chunhinta Black: Chunhinta Variety	0	0	2	9	0	**11**
Deprecio Incised: Deprecio Variety	0	0	2	2	2	**6**
Guitara Incised: Guitara Variety	0	1	3	3	0	**7**
Jocote Orange-brown: Jocote Variety	0	11	0	1	0	**12**
Joventud Red: Joventud Variety	0	0	11	4	0	**15**
Muxanal Red-on-cream: Muxanal Variety	0	0	0	0	2	**2**
Muxanal Red-on-cream: Variety Unspecified	0	0	1	0	0	**1**
Paso Danto Incised: Paso Danto Variety	0	0	0	2	0	**2**
Pital Cream: Pital Variety	0	0	2	0	0	**2**
Reforma Incised: Mucnal Variety	7	0	0	0	0	**7**
Savana Orange: Rejolla Variety	13	0	0	0	1	**14**
Savana Orange: Savana Variety	2	0	0	0	0	**2**
Tierra Mojada Resist: Tierra Mojada Variety	0	0	1	2	1	**4**
Timax Incised: Timax Variety	0	0	1	1	0	**2**
**TOTAL**	**22**	**12**	**25**	**28**	**6**	**93**

Chi square = 241.3, df = 60, alpha = .05, critical value = 79.082

Table 2. Ceramic Group by Paste Group

Group	Group 1	Group 2	Group 3	Group 4	Unassigned	TOTAL
Achiotes	0	0	0	4	0	**4**
Chunhinta	0	0	6	11	2	**19**
Jocote	0	11	0	1	0	**12**
Joventud	0	1	14	7	0	**22**
Muxanal	0	0	1	0	2	**3**
Pital	0	0	2	2	0	**4**
Savana	22	0	0	0	1	**23**
Tierra Mojada	0	0	2	3	1	**6**
**TOTAL**	**22**	**12**	**25**	**28**	**6**	**93**

Chi square = 199.7, df = 28, alpha = .05,
critical value = 41.3372

Table 3. Ceramic Ware by Paste Group

Ware	Group 1	Group 2	Group 3	Group 4	Unassigned	TOTAL
Flores Waxy	0	1	25	23	5	54
Mars Orange Paste	22	0	0	0	1	23
Uaxactun Unslipped	0	11	0	5	0	16
**TOTAL**	22	12	25	28	6	93

Chi square = 143.3, df = 8, alpha = .05,
critical value = 15.5073

Table 4. Temper by Paste Group

Temper	Group 1	Group 2	Group 3	Group 4	Unassigned	TOTAL
Calcite	1	12	18	28	6	**65**
Volcanic ash	21		7			**28**
**TOTAL**	**22**	**12**	**25**	**28**	**6**	**93**

Chi square = 64.5, df = 4, alpha = .05,
critical value = 9.4877

Table 5. Decoration by Paste Group

Decoration	Group 1	Group 2	Group 3	Group 4	Unassigned	TOTAL
Dichrome	0	0	0	0	2	2
Incising	7	1	7	8	2	25
Plain slipped	13	0	15	13	1	42
Fluted	0	0	2	0	0	2
Resist	0	0	1	2	1	4
Unslipped	2	0	0	4	0	6
Unslipped applique	0	11	0	1	0	12
**TOTAL**	22	12	25	28	6	93

Chi square value = 121.2, df = 24, alpha = .05, critical value = 36.415

Table 6. Form by Paste Group

Form	Group 1	Group 2	Group 3	Group 4	Unassigned	TOTAL
Bowl	16	1	24	18	3	62
Jar	6	11	1	10	3	31
**TOTAL**	22	12	25	28	6	93

Chi square value = 29.2, df = 4, alpha = .05, critical value = 9.4877
