# Supplementary material for: An atlas of paste fabrics and supplemental paste compositional data from late middle preclassic-period ceramics at the Maya site of Holtun, Guatemala
Source: Data Brief. 2017 Mar 19;12:55–67. doi: 10.1016/j.dib.2017.03.024 (PMC5376253; doi:10.1016/j.dib.2017.03.024)
Supplement: Supplementary file 3 — Supplementary material [file mmc3.zip › Appendix A/Table A-1.docx]

**Table A-1. List of samples with context numbers, wares, type: variety, temper, and form**

* For source group, final source assignment, and closest matches in MURR database see Callaghan et al. 2017 Table 1

| **ANID** | **Context** | **Vessel Ware** | **Type:Variety** | **Temper** | **Vessel Form** |
| --- | --- | --- | --- | --- | --- |
| HTN001 | 1/1/10 | Flores Waxy | Chunhinta Black: Chunhinta Variety | Calcite | jar |
| HTN002 | 1/1/10 | Flores Waxy | Deprecio Incised: Deprecio Variety | Calcite | jar |
| HTN003 | 1/1/10 | Flores Waxy | Muxanal Red-on-cream: Muxanal Variety | Calcite | jar |
| HTN004 | 1/1/10 | Mars Orange | Reforma Incised: Mucnal Variety | Volcanic | bowl |
| HTN005 | 1/1/10 | Flores Waxy | Joventud Red: Joventud Variety | Calcite | bowl |
| HTN006 | 1/1/11 | Flores Waxy | Deprecio Incised: Deprecio Variety | Calcite | bowl |
| HTN007 | 1/1/11 | Flores Waxy | Chunhinta Black: Chunhinta Variety | Calcite | bowl |
| HTN008 | 1/1/11 | Flores Waxy | Joventud Red: Joventud Variety | Calcite | bowl |
| HTN009 | 1/1/11 | Flores Waxy | Joventud Red: Joventud Variety | Calcite | bowl |
| HTN010 | 1/1/11 | Flores Waxy | Tierra Mojada Resist: Tierra Mojada Variety | Calcite | bowl |
| HTN011 | 1/1/11 | Achiotes Unslipped | Achiotes Unslipped: Achiotes Variety | Calcite | jar |
| HTN012 | 1/1/12 | Achiotes Unslipped | Achiotes Unslipped: Achiotes Variety | Calcite | jar |
| HTN013 | 1/1/12 | Achiotes Unslipped | Achiotes Unslipped: Achiotes Variety | Calcite | jar |
| HTN014 | 1/1/12 | Flores Waxy | Chunhinta Black: Chunhinta Variety | Calcite | jar |
| HTN015 | 1/1/12 | Flores Waxy | Joventud Red: Joventud Variety | Calcite | bowl |
| HTN016 | 1/1/12 | Flores Waxy | Pital Cream: Pital Variety | Calcite | bowl |
| HTN017 | 1/1/12 | Flores Waxy | Guitara Incised: Guitara Variety | Calcite | bowl |
| HTN018 | 1/1/12 | Flores Waxy | Centenario Fluted: Centenario Variety | Calcite | bowl |
| HTN019 | 1/1/12 | Flores Waxy | Tierra Mojada Resist: Tierra Mojada Variety | Calcite | bowl |
| HTN020 | 1/1/12 | Mars Orange | Savana Orange: Rejolla Variety | Volcanic | bowl |
| HTN021 | 1/1/12 | Mars Orange | Savana Orange: Rejolla Variety | Volcanic | bowl |
| HTN022 | 1/1/12 | Mars Orange | Savana Orange: Savana Variety | Volcanic | bowl |
| HTN023 | 1/1/12 | Flores Waxy | Chunhinta Black: Chunhinta Variety | Calcite | bowl |
| HTN024 | 1/1/12 | Flores Waxy | Chunhinta Black: Chunhinta Variety | Calcite | jar |
| HTN025 | 1/1/12 | Flores Waxy | Chunhinta Black: Chunhinta Variety | Volcanic | bowl |
| HTN026 | 1/1/13 | Flores Waxy | Joventud Red: Joventud Variety | Calcite | bowl |
| HTN027 | 1/1/13 | Flores Waxy | Guitara Incised: Guitara Variety | Volcanic | bowl |
| HTN028 | 1/1/13 | Flores Waxy | Joventud Red: Joventud Variety | Volcanic | bowl |
| HTN029 | 1/1/13 | Flores Waxy | Deprecio Incised: Deprecio Variety | Calcite | bowl |
| HTN030 | 1/1/13 | Flores Waxy | Guitara Incised: Guitara Variety | Calcite | bowl |
| HTN031 | 1/1/13 | Mars Orange | Reforma Incised: Mucnal Variety | Volcanic | bowl |
| HTN032 | 1/1/13 | Mars Orange | Reforma Incised: Mucnal Variety | Volcanic | bowl |
| HTN033 | 1/1/13 | Flores Waxy | Chunhinta Black: Chunhinta Variety | Calcite | jar |
| HTN034 | 1/1/13 | Mars Orange | Savana Orange: Rejolla Variety | Volcanic | jar |
| HTN035 | 1/1/13 | Mars Orange | Savana Orange: Rejolla Variety | Volcanic | jar |
| HTN036 | 1/1/14 | Flores Waxy | Joventud Red: Joventud Variety | Calcite | bowl |
| HTN037 | 1/1/14 | Flores Waxy | Deprecio Incised: Deprecio Variety | Calcite | bowl |
| HTN038 | 1/1/14 | Flores Waxy | Muxanal Red-on-cream: Muxanal Variety | Calcite | bowl |
| HTN039 | 1/1/14 | Flores Waxy | Joventud Red: Joventud Variety | Calcite | bowl |
| HTN040 | 1/1/14 | Flores Waxy | Joventud Red: Joventud Variety | Calcite | bowl |
| HTN041 | 1/1/14 | Flores Waxy | Joventud Red: Joventud Variety | Calcite | bowl |
| HTN042 | 1/1/14 | Flores Waxy | Paso Danto Incised: Paso Danto Variety | Calcite | bowl |
| HTN043 | 1/1/14 | Flores Waxy | Chunhinta Black: Chunhinta Variety | Calcite | bowl |
| HTN044 | 1/1/14 | Flores Waxy | Guitara Incised: Guitara Variety | Calcite | jar |
| HTN045 | 1/1/14 | Flores Waxy | Timax Incised: Timax Variety | Calcite | bowl |
| HTN046 | 1/1/15 | Achiotes Unslipped | Jocote Orange-brown: Jocote Variety | Calcite | jar |
| HTN047 | 1/1/15 | Achiotes Unslipped | Jocote Orange-brown: Jocote Variety | Calcite | jar |
| HTN048 | 1/1/15 | Achiotes Unslipped | Achiotes Unslipped: Achiotes Variety | Calcite | jar |
| HTN049 | 1/1/15 | Unnamed | Unnamed Eroded (Ah Pahm Complex?) | Calcite | bowl |
| HTN050 | 1/1/15 | Flores Waxy | Paso Danto Incised: Paso Danto Variety | Calcite | bowl |
| HTN051 | 1/1/15 | Flores Waxy | Guitara Incised: Guitara Variety | Volcanic | bowl |
| HTN052 | 1/1/15 | Flores Waxy | Deprecio Incised: Deprecio Variety | Calcite | bowl |
| HTN053 | 1/1/15 | Flores Waxy | Guitara Incised: Guitara Variety | Volcanic | bowl |
| HTN054 | 1/1/15 | Flores Waxy | Timax Incised: Timax Variety | Calcite | bowl |
| HTN055 | 1/1/15 | Flores Waxy | Joventud Red: Joventud Variety | Calcite | bowl |
| HTN056 | 1/1/15 | Flores Waxy | Pital Cream: Pital Variety | Calcite | bowl |
| HTN057 | 1/1/15 | Flores Waxy | Muxanal Red-on-cream: Variety Unspecified | Volcanic | bowl |
| HTN058 | 1/1/15 | Mars Orange | Reforma Incised: Mucnal Variety | calcite | jar |
| HTN059 | 1/1/15 | Mars Orange | Savana Orange: Rejolla Variety | Volcanic | bowl |
| HTN060 | 1/1/15 | Flores Waxy | Tierra Mojada Resist: Tierra Mojada Variety | Calcite | bowl |
| HTN061 | 1/1/15 | Flores Waxy | Chunhinta Black: Chunhinta Variety | Calcite | bowl |
| HTN062 | 1/1/15 | Flores Waxy | Joventud Red: Joventud Variety | Calcite | bowl |
| HTN063 | 1/1/15 | Flores Waxy | Joventud Red: Joventud Variety | Calcite | bowl |
| HTN064 | 1/1/16 | Flores Waxy | Guitara Incised: Guitara Variety | Calcite | bowl |
| HTN065 | 1/1/16 | Flores Waxy | Chunhinta Black: Chunhinta Variety | Calcite | jar |
| HTN066 | 1/1/16 | Flores Waxy | Joventud Red: Joventud Variety | Calcite | bowl |
| HTN067 | 3/1/06 | Flores Waxy | Centenario Fluted: Centenario Variety | Volcanic | bowl |
| HTN068 | 3/1/06 | Flores Waxy | Tierra Mojada Resist: Tierra Mojada Variety | Calcite | bowl |
| HTN069 | 3/1/06 | Flores Waxy | Deprecio Incised: Deprecio Variety | Calcite | bowl |
| HTN070 | 3/1/06 | Flores Waxy | Joventud Red: Joventud Variety | Calcite | bowl |
| HTN071 | 3/1/06 | Flores Waxy | Chunhinta Black: Chunhinta Variety | Calcite | bowl |
| HTN072 | 3/1/06 | Achiotes Unslipped | Jocote Orange-brown: Jocote Variety | Calcite | jar |
| HTN073 | 3/1/06 | Achiotes Unslipped | Jocote Orange-brown: Jocote Variety | Calcite | jar |
| HTN074 | 3/1/06 | Achiotes Unslipped | Jocote Orange-brown: Jocote Variety | Calcite | jar |
| HTN075 | 3/1/06 | Achiotes Unslipped | Jocote Orange-brown: Jocote Variety | Calcite | jar |
| HTN076 | 3/1/06 | Achiotes Unslipped | Jocote Orange-brown: Jocote Variety | Calcite | jar |
| HTN077 | 3/1/06 | Achiotes Unslipped | Jocote Orange-brown: Jocote Variety | Calcite | jar |
| HTN078 | 3/1/06 | Achiotes Unslipped | Jocote Orange-brown: Jocote Variety | Calcite | jar |
| HTN079 | 3/1/06 | Achiotes Unslipped | Jocote Orange-brown: Jocote Variety | Calcite | jar |
| HTN080 | 3/1/06 | Achiotes Unslipped | Jocote Orange-brown: Jocote Variety | Calcite | jar |
| HTN081 | 3/1/06 | Achiotes Unslipped | Jocote Orange-brown: Jocote Variety | Calcite | jar |
| HTN082g | 3/1/06 | Mars Orange | Unnamed | indeterminate | indeterminate |
| HTN082r | 3/1/06 | Mars Orange | Unnamed | indeterminate | indeterminate |
| HTN083 | 3/1/06 | Mars Orange | Savana Orange: Savana Variety | Volcanic | bowl |
| HTN084 | 3/1/06 | Mars Orange | Savana Orange: Rejolla Variety | Volcanic | bowl |
| HTN085 | 3/1/06 | Mars Orange | Savana Orange: Rejolla Variety | Calcite | jar |
| HTN086 | 3/1/06 | Mars Orange | Savana Orange: Rejolla Variety | Volcanic | jar |
| HTN087 | 3/1/06 | Mars Orange | Savana Orange: Rejolla Variety | Volcanic | bowl |
| HTN088 | 3/1/06 | Mars Orange | Savana Orange: Rejolla Variety | Volcanic | bowl |
| HTN089 | 3/1/06 | Mars Orange | Savana Orange: Rejolla Variety | Volcanic | bowl |
| HTN090 | 3/1/06 | Mars Orange | Reforma Incised: Mucnal Variety | Volcanic | bowl |
| HTN091 | 3/1/06 | Mars Orange | Reforma Incised: Mucnal Variety | Volcanic | bowl |
| HTN092 | 3/1/06 | Mars Orange | Reforma Incised: Mucnal Variety | Volcanic | bowl |
| HTN093 | 3/1/06 | Mars Orange | Savana Orange: Rejolla Variety | Volcanic | jar |
| HTN094 | 3/1/06 | Mars Orange | Savana Orange: Rejolla Variety | Volcanic | jar |
| HTN095 | 3/1/06 | Mars Orange | Savana Orange: Rejolla Variety | Volcanic | bowl |
| HTN096 | 3/1/06 | Undetermined | Undetermined | Calcite | jar |
| HTN097 | 3/1/06 | Undetermined | Undetermined | Calcite | bowl |
